# Supplementary figures and images for: Nuclear PD-L1 triggers tumour-associated inflammation upon DNA damage (part 1 of 3)
Source: EMBO Rep. 2025 Jan 2;26(3):635–55. doi: 10.1038/s44319-024-00354-9 (PMC11811057; doi:10.1038/s44319-024-00354-9)

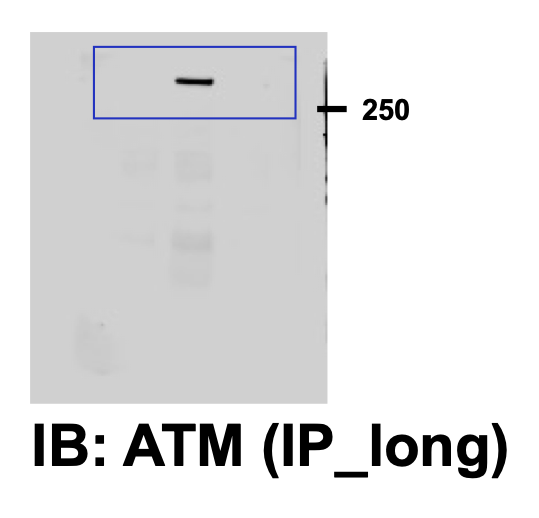

Supplement: Supplementary file 2 — Source data Fig. 1 [file 44319_2024_354_MOESM2_ESM.zip › Figure 1/1A/ATM_IP_long.tif]

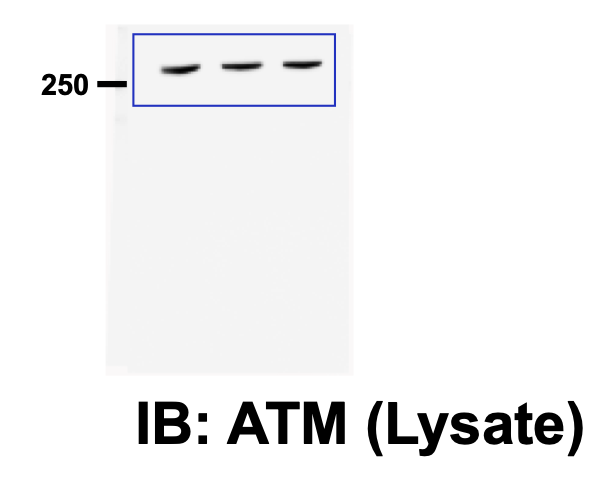

Supplement: Supplementary file 2 — Source data Fig. 1 [file 44319_2024_354_MOESM2_ESM.zip › Figure 1/1A/ATM_lysate.tif]

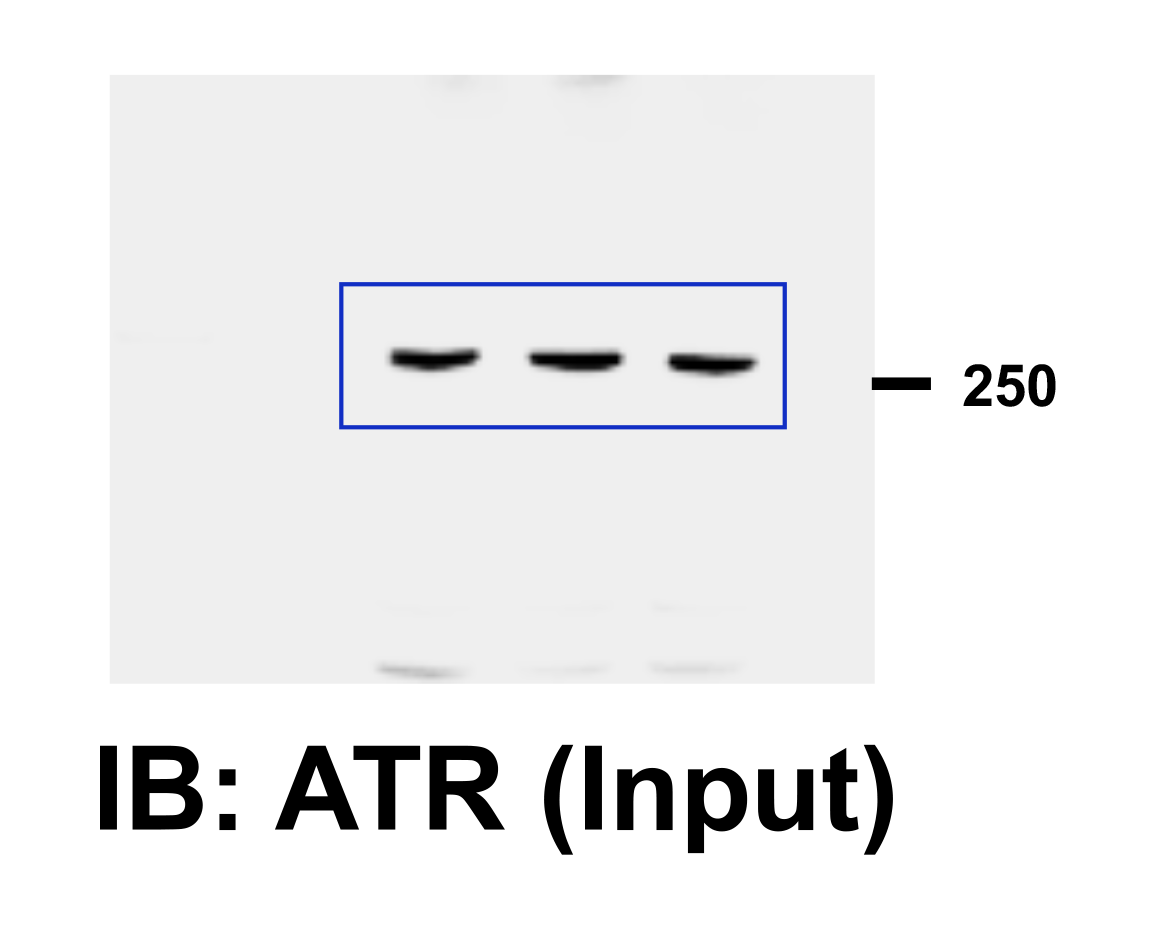

Supplement: Supplementary file 2 — Source data Fig. 1 [file 44319_2024_354_MOESM2_ESM.zip › Figure 1/1A/ATR_Input.tif]

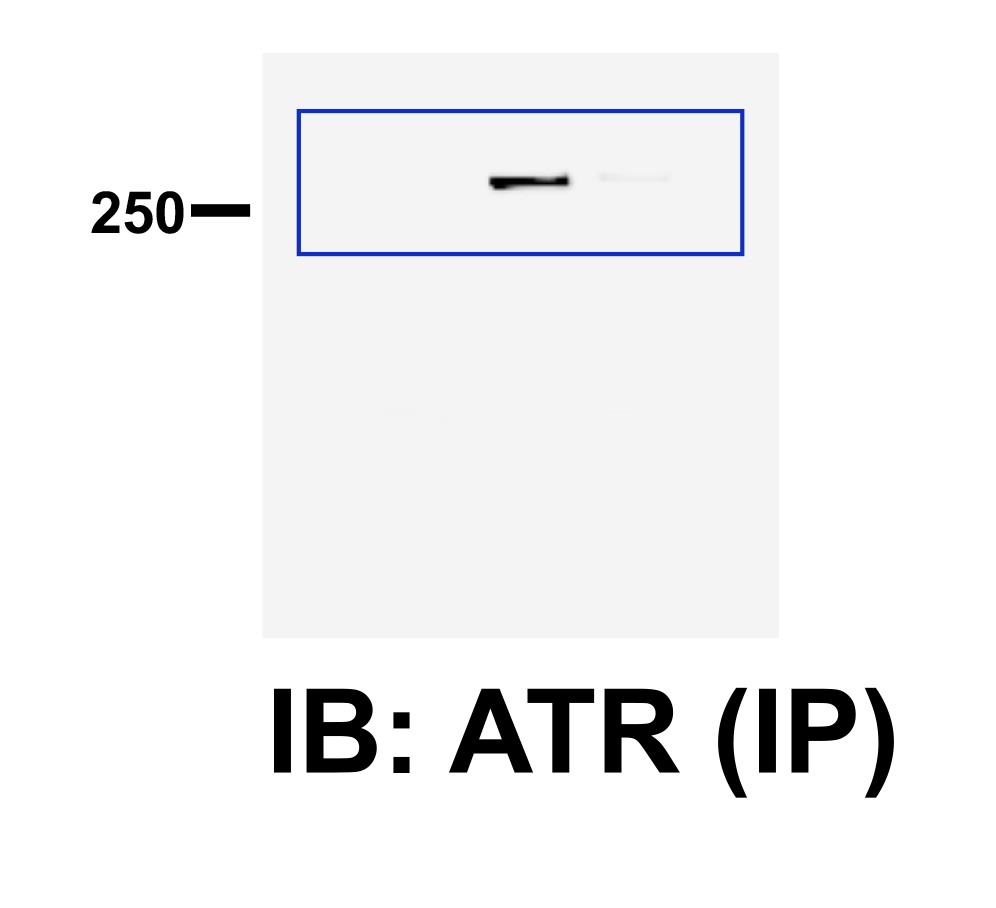

Supplement: Supplementary file 2 — Source data Fig. 1 [file 44319_2024_354_MOESM2_ESM.zip › Figure 1/1A/ATR_IP.tif]

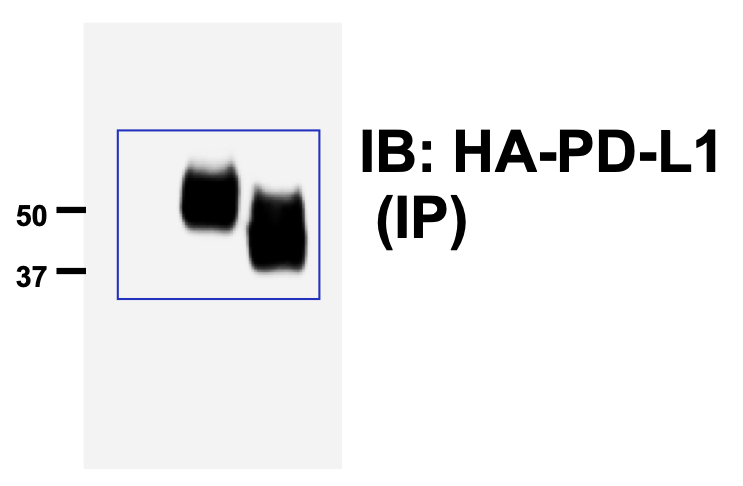

Supplement: Supplementary file 2 — Source data Fig. 1 [file 44319_2024_354_MOESM2_ESM.zip › Figure 1/1A/HA-PD-L1_IP.tif]

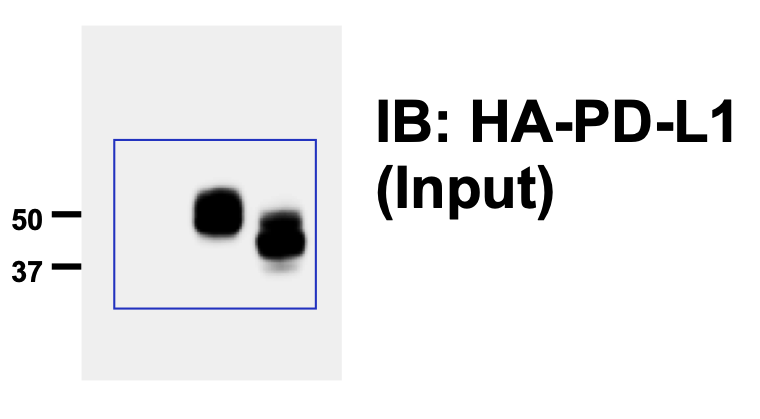

Supplement: Supplementary file 2 — Source data Fig. 1 [file 44319_2024_354_MOESM2_ESM.zip › Figure 1/1A/HA-PD-L1_lysate.tif]

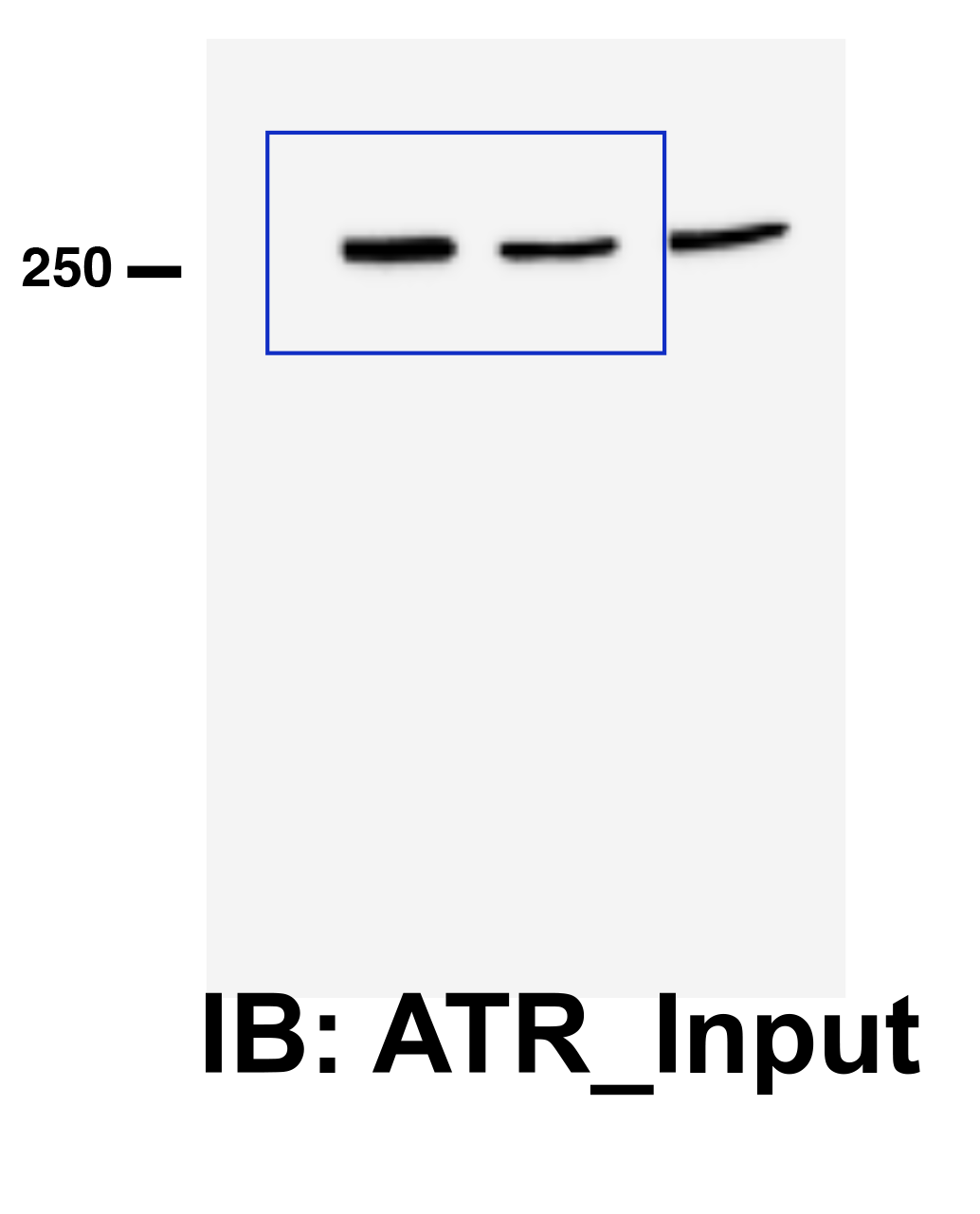

Supplement: Supplementary file 2 — Source data Fig. 1 [file 44319_2024_354_MOESM2_ESM.zip › Figure 1/1B/ATR_input.tif]

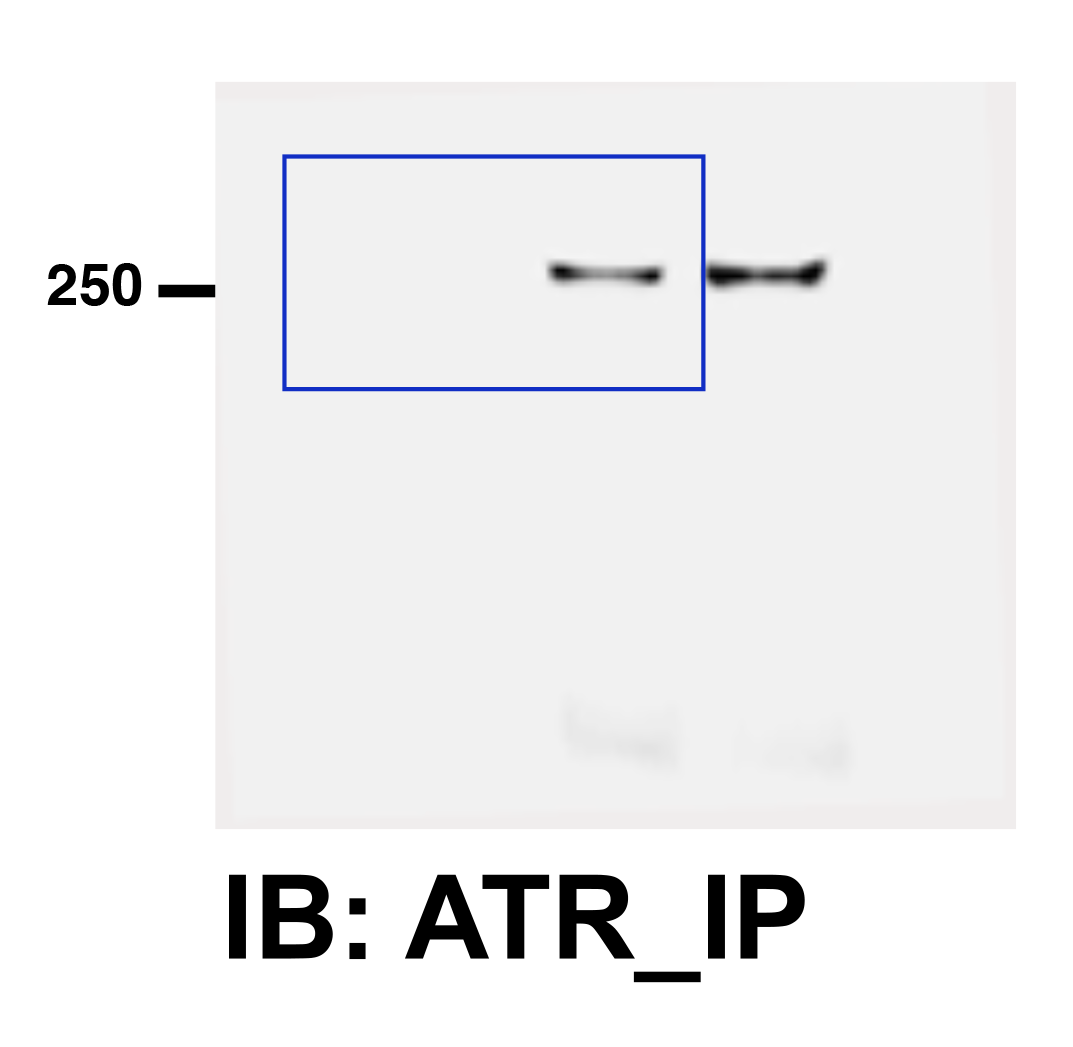

Supplement: Supplementary file 2 — Source data Fig. 1 [file 44319_2024_354_MOESM2_ESM.zip › Figure 1/1B/ATR_IP.tif]

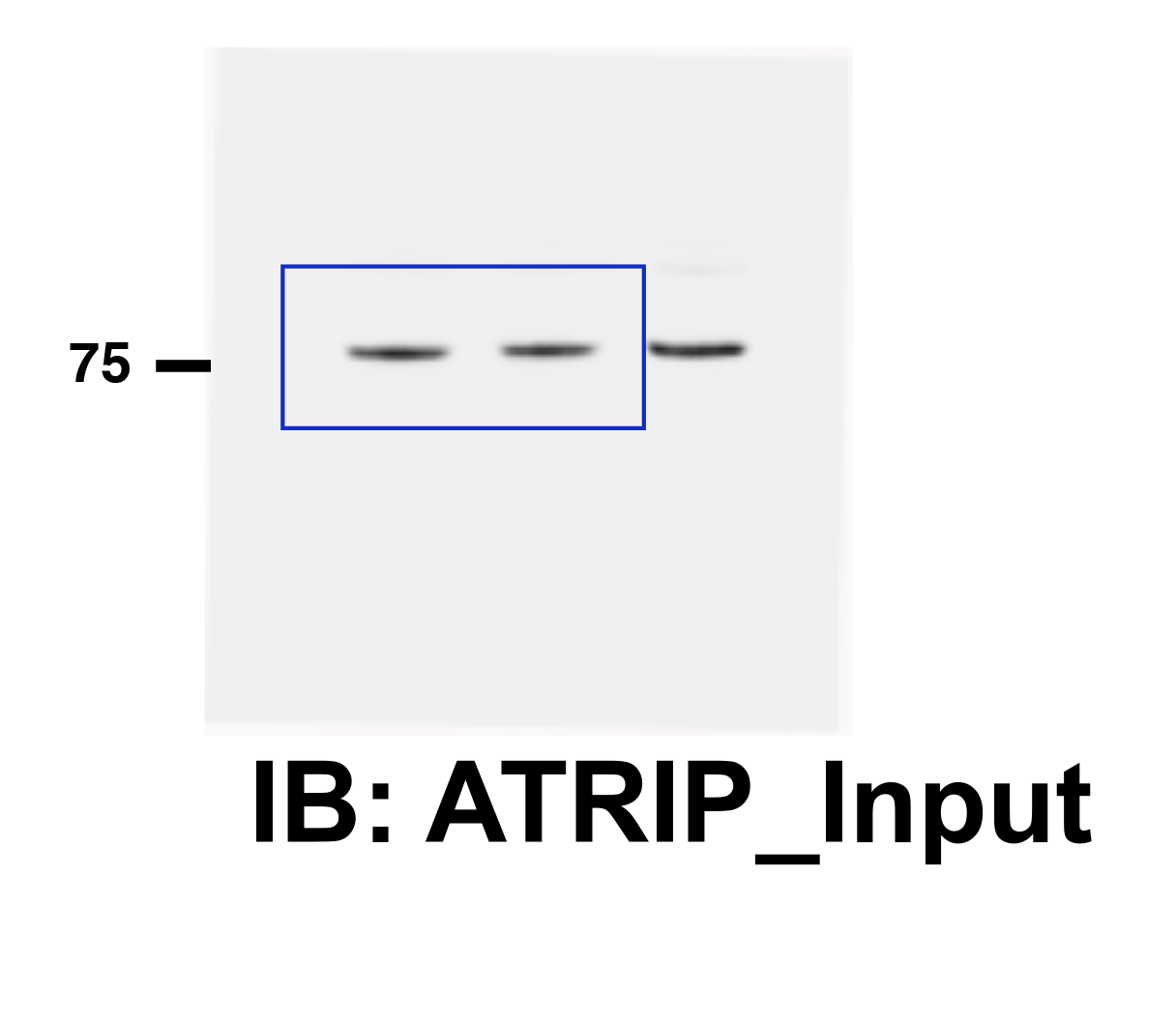

Supplement: Supplementary file 2 — Source data Fig. 1 [file 44319_2024_354_MOESM2_ESM.zip › Figure 1/1B/ATRIP_input.tif]

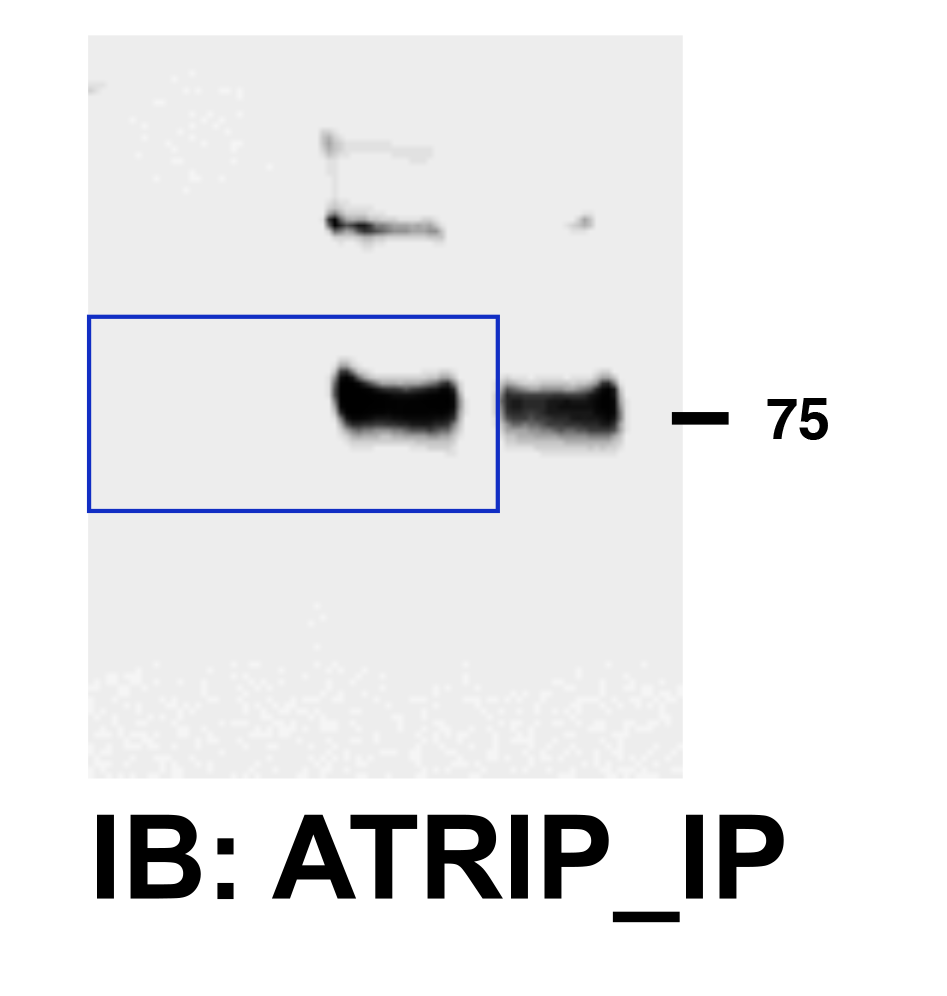

Supplement: Supplementary file 2 — Source data Fig. 1 [file 44319_2024_354_MOESM2_ESM.zip › Figure 1/1B/ATRIP_IP.tif]

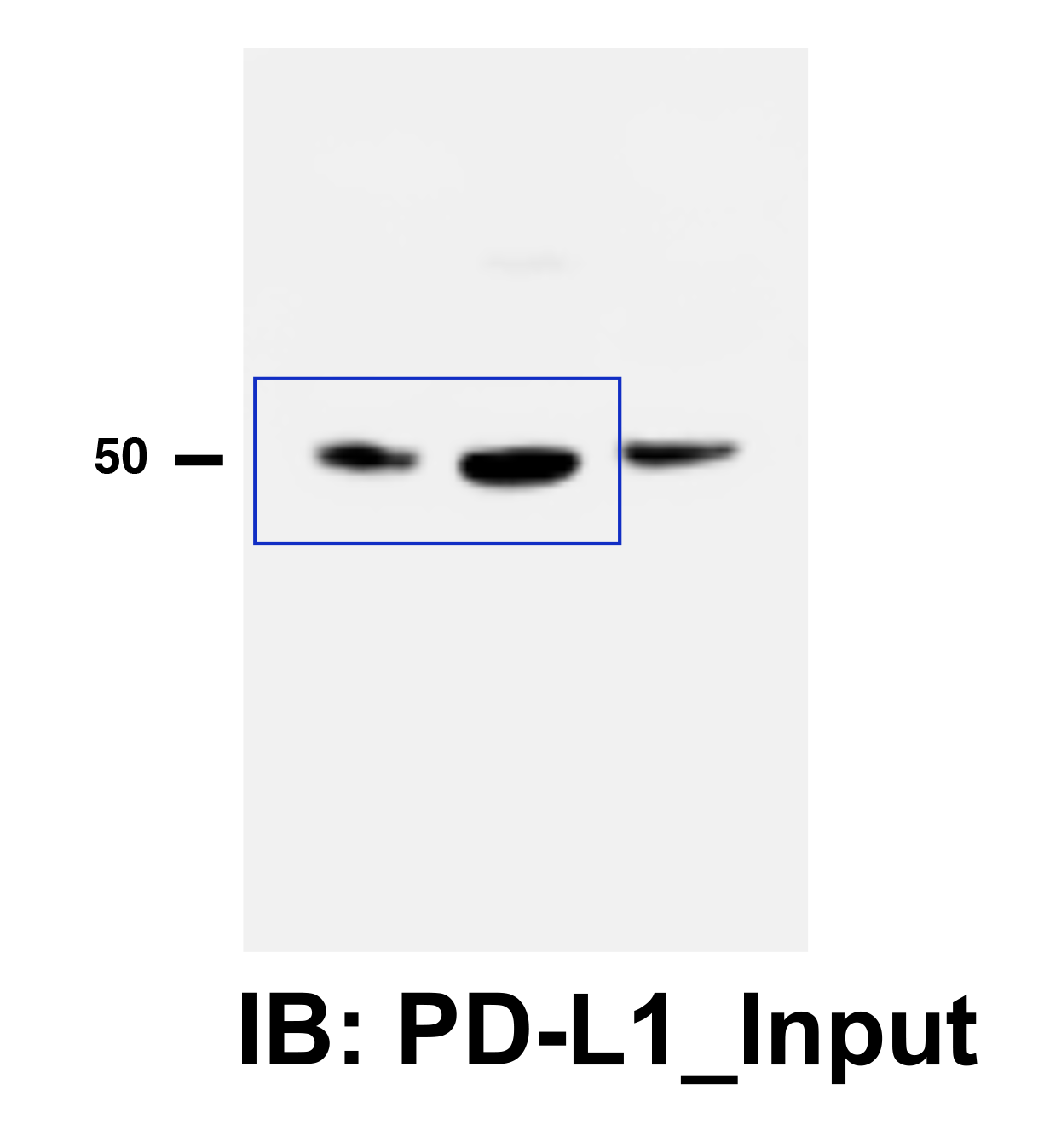

Supplement: Supplementary file 2 — Source data Fig. 1 [file 44319_2024_354_MOESM2_ESM.zip › Figure 1/1B/PD-L1_input.tif]

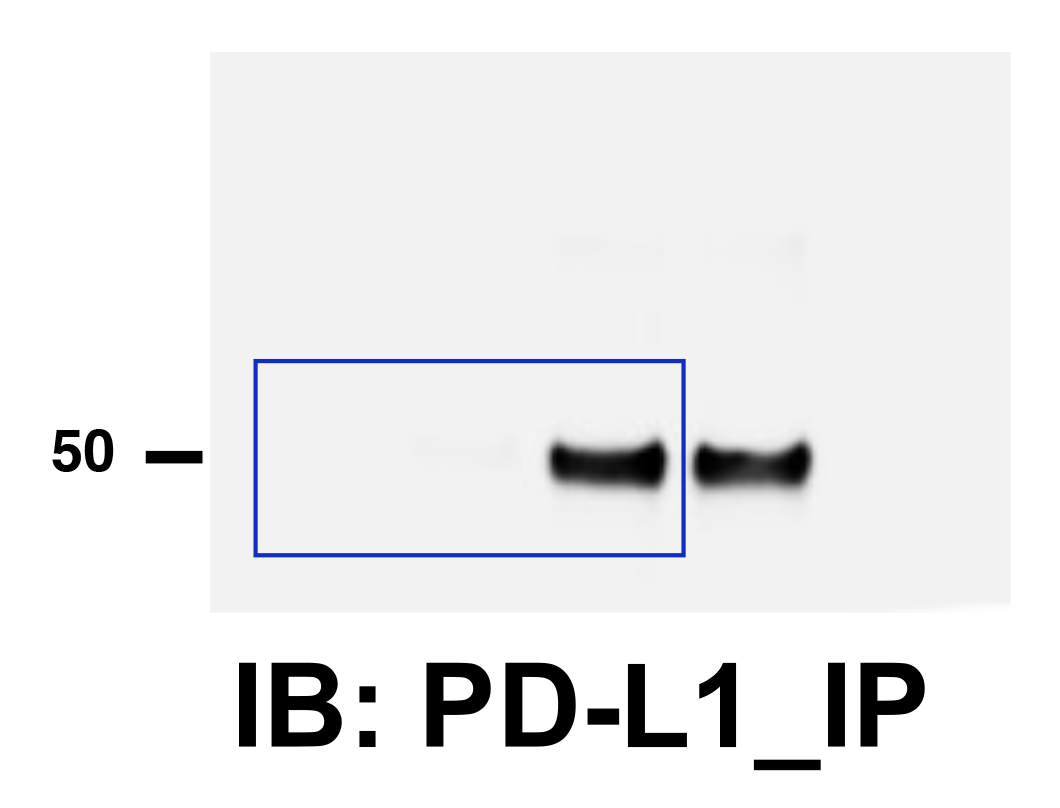

Supplement: Supplementary file 2 — Source data Fig. 1 [file 44319_2024_354_MOESM2_ESM.zip › Figure 1/1B/PD-L1_IP.tif]

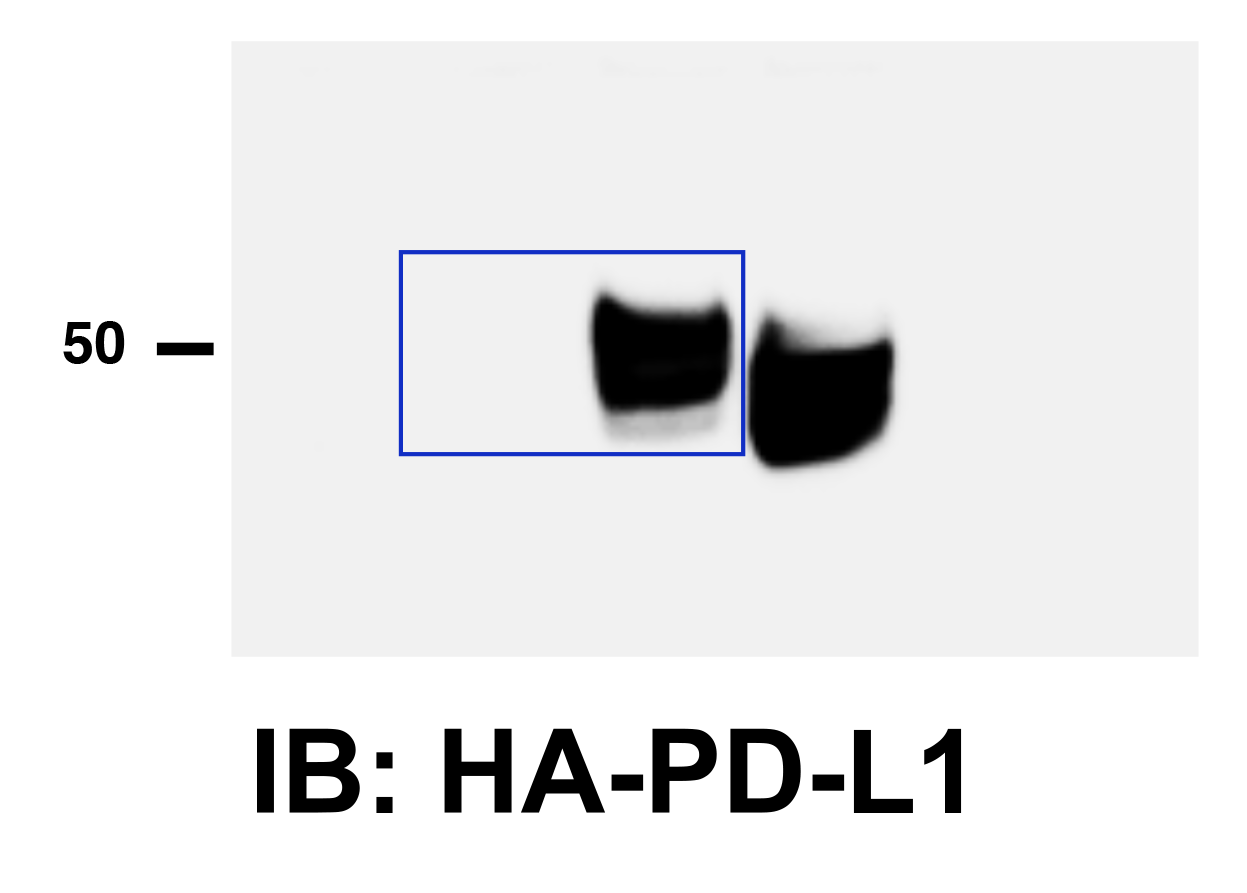

Supplement: Supplementary file 2 — Source data Fig. 1 [file 44319_2024_354_MOESM2_ESM.zip › Figure 1/1C/HA-PD-L1.tif]

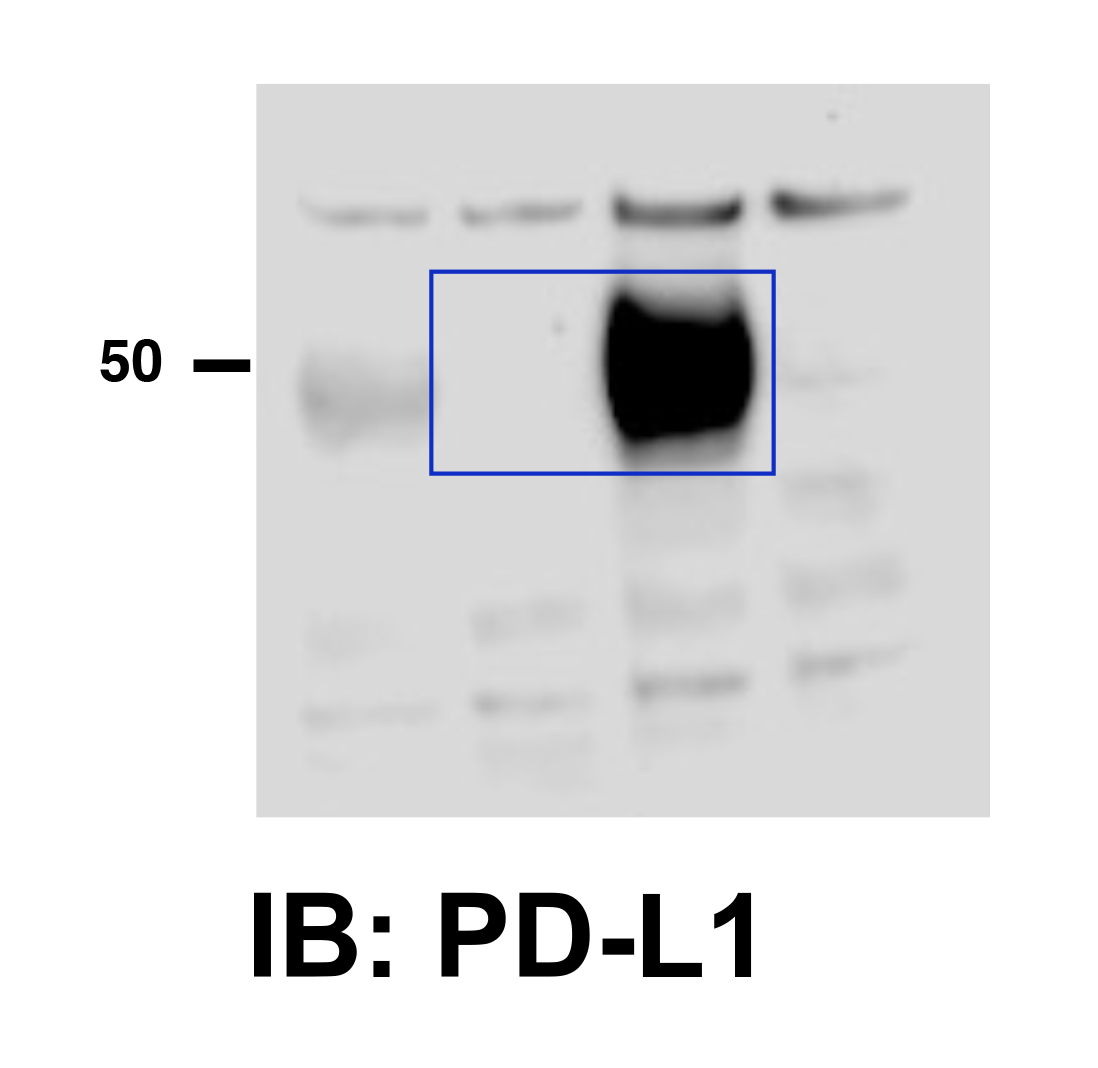

Supplement: Supplementary file 2 — Source data Fig. 1 [file 44319_2024_354_MOESM2_ESM.zip › Figure 1/1C/PD-L1.tif]

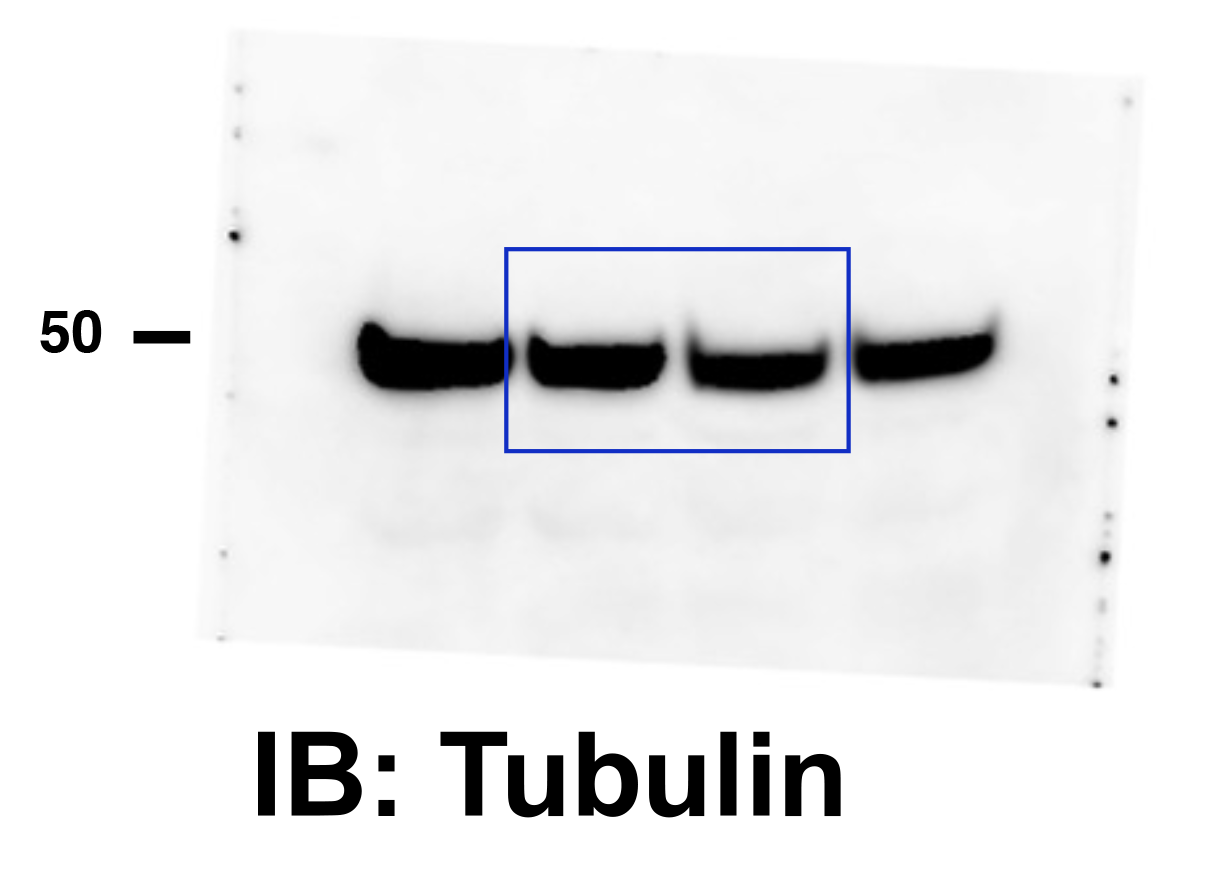

Supplement: Supplementary file 2 — Source data Fig. 1 [file 44319_2024_354_MOESM2_ESM.zip › Figure 1/1C/Tutulin.tif]

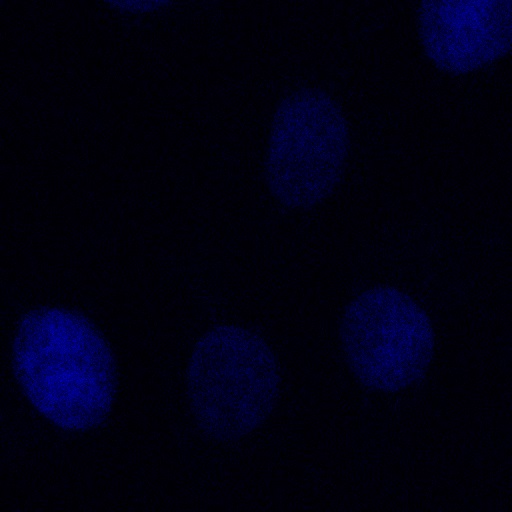

Supplement: Supplementary file 2 — Source data Fig. 1 [file 44319_2024_354_MOESM2_ESM.zip › Figure 1/1D/KO/DAPI.jpg]

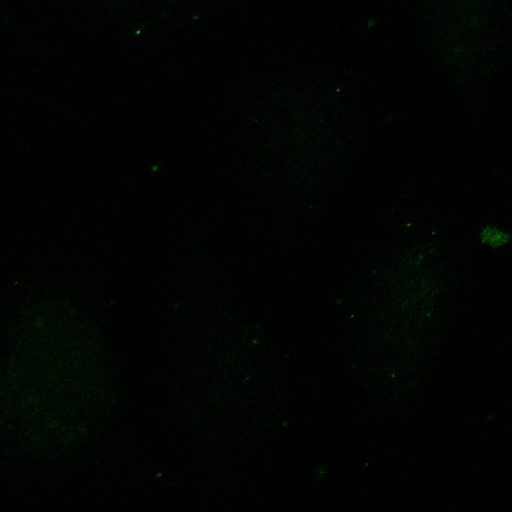

Supplement: Supplementary file 2 — Source data Fig. 1 [file 44319_2024_354_MOESM2_ESM.zip › Figure 1/1D/KO/HA.jpg]

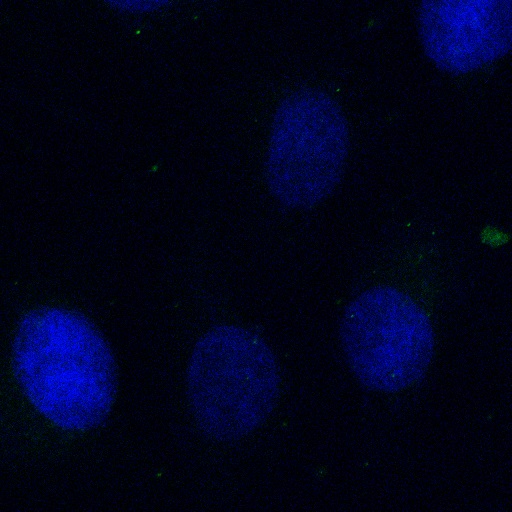

Supplement: Supplementary file 2 — Source data Fig. 1 [file 44319_2024_354_MOESM2_ESM.zip › Figure 1/1D/KO/Merge.jpg]

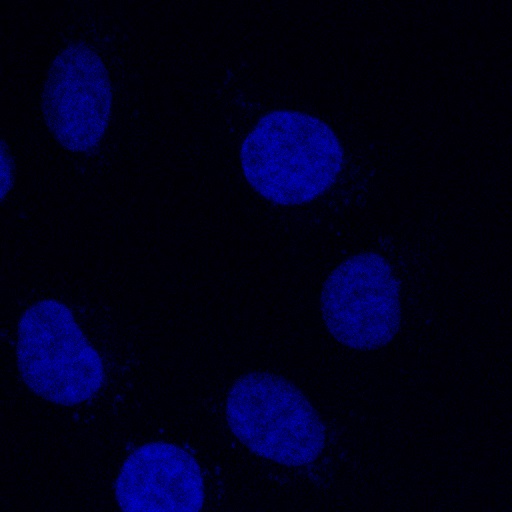

Supplement: Supplementary file 2 — Source data Fig. 1 [file 44319_2024_354_MOESM2_ESM.zip › Figure 1/1D/KO_Addback HA-PD-L1 FL/DAPI.jpg]

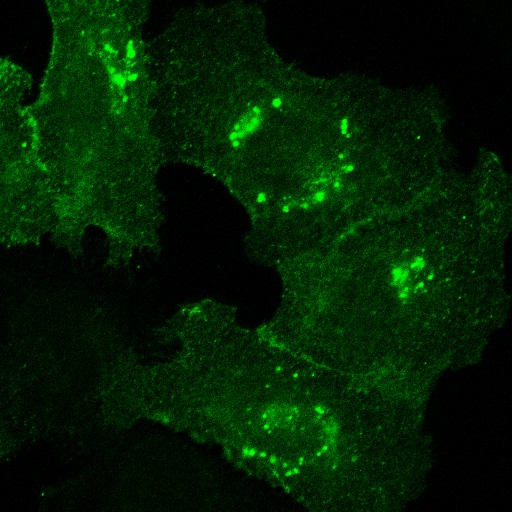

Supplement: Supplementary file 2 — Source data Fig. 1 [file 44319_2024_354_MOESM2_ESM.zip › Figure 1/1D/KO_Addback HA-PD-L1 FL/HA.jpg]

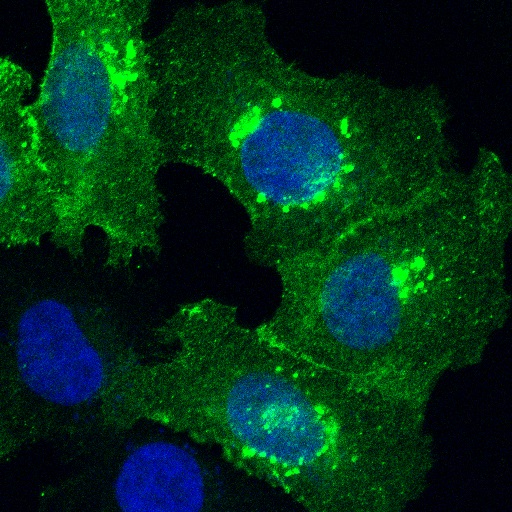

Supplement: Supplementary file 2 — Source data Fig. 1 [file 44319_2024_354_MOESM2_ESM.zip › Figure 1/1D/KO_Addback HA-PD-L1 FL/Merge.jpg]

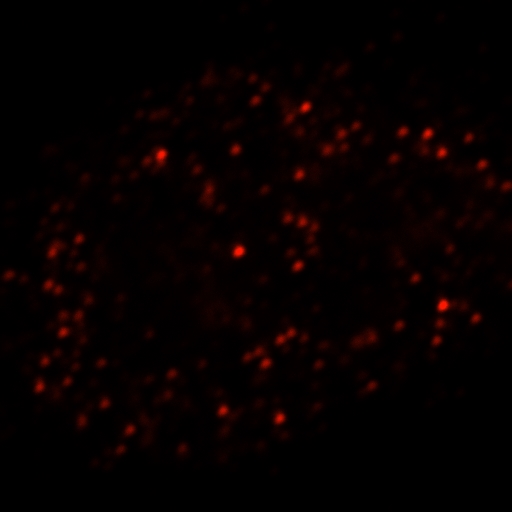

Supplement: Supplementary file 2 — Source data Fig. 1 [file 44319_2024_354_MOESM2_ESM.zip › Figure 1/1E/Enlarge_KO _addback HA-PD-L1_non-treatment/ATR.jpg]

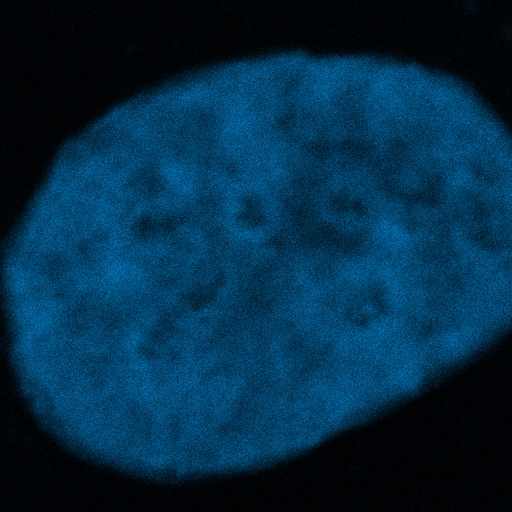

Supplement: Supplementary file 2 — Source data Fig. 1 [file 44319_2024_354_MOESM2_ESM.zip › Figure 1/1E/Enlarge_KO _addback HA-PD-L1_non-treatment/DAPI.jpg]

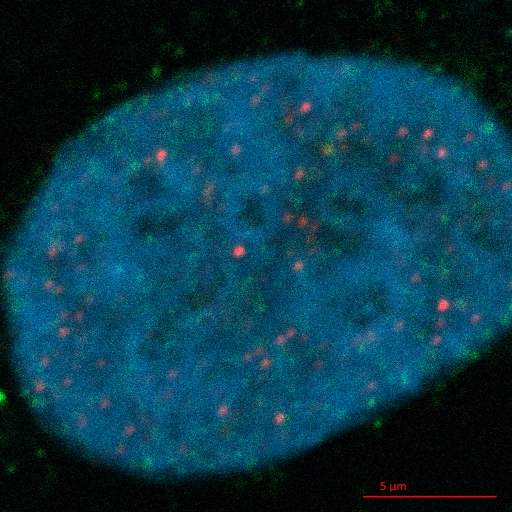

Supplement: Supplementary file 2 — Source data Fig. 1 [file 44319_2024_354_MOESM2_ESM.zip › Figure 1/1E/Enlarge_KO _addback HA-PD-L1_non-treatment/FL-non-HA(g)-ATR(r)-18-Scale/FL-non-HA(g)-ATR(r)-18_c1+2+3.jpg]

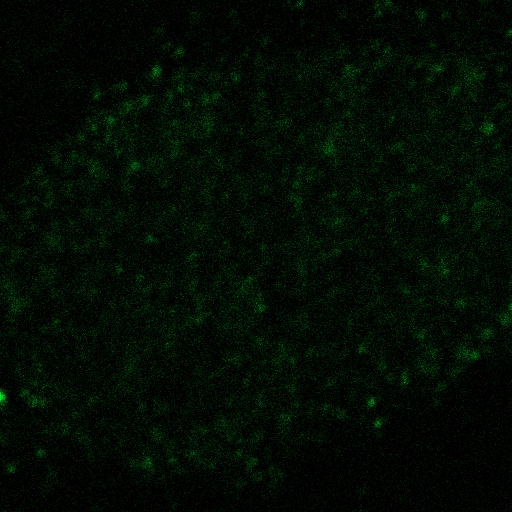

Supplement: Supplementary file 2 — Source data Fig. 1 [file 44319_2024_354_MOESM2_ESM.zip › Figure 1/1E/Enlarge_KO _addback HA-PD-L1_non-treatment/HA.jpg]

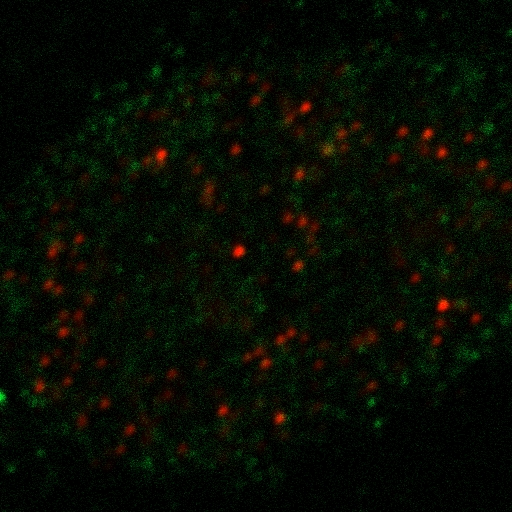

Supplement: Supplementary file 2 — Source data Fig. 1 [file 44319_2024_354_MOESM2_ESM.zip › Figure 1/1E/Enlarge_KO _addback HA-PD-L1_non-treatment/Merge.jpg]

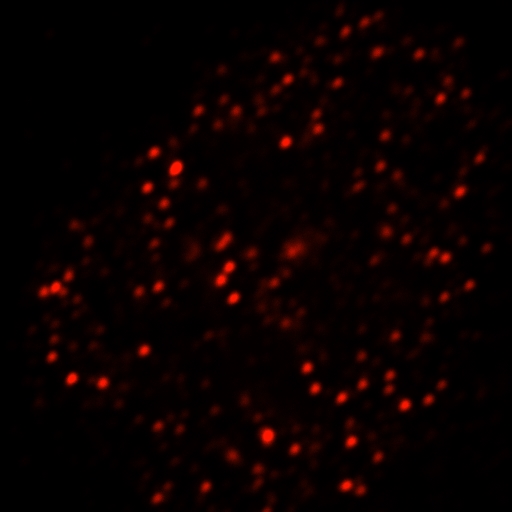

Supplement: Supplementary file 2 — Source data Fig. 1 [file 44319_2024_354_MOESM2_ESM.zip › Figure 1/1E/Enlarge_KO_addback HA-PD-L1_IR/ATR.jpg]

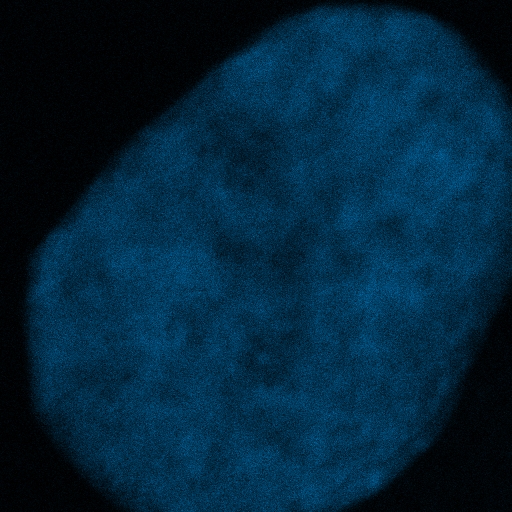

Supplement: Supplementary file 2 — Source data Fig. 1 [file 44319_2024_354_MOESM2_ESM.zip › Figure 1/1E/Enlarge_KO_addback HA-PD-L1_IR/DAPI.jpg]

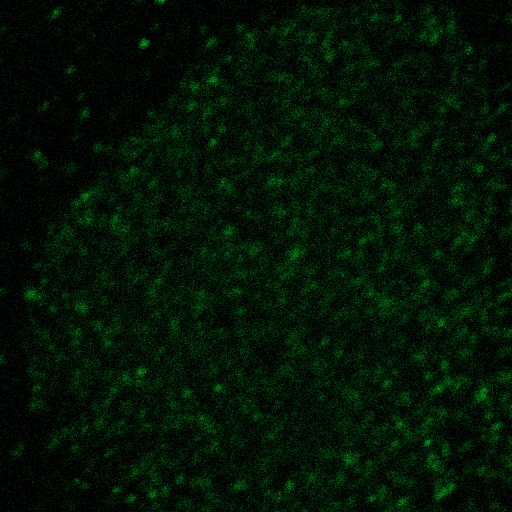

Supplement: Supplementary file 2 — Source data Fig. 1 [file 44319_2024_354_MOESM2_ESM.zip › Figure 1/1E/Enlarge_KO_addback HA-PD-L1_IR/HA.jpg]

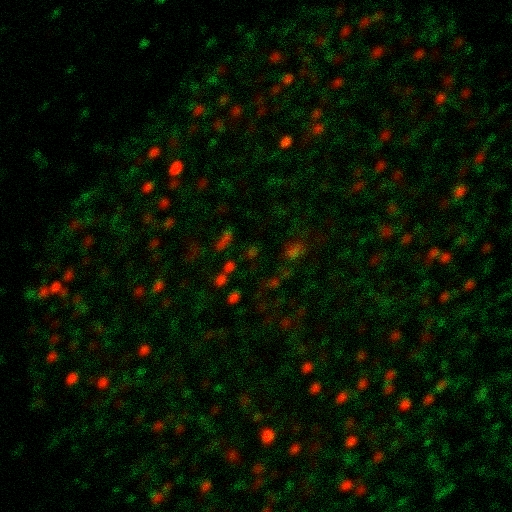

Supplement: Supplementary file 2 — Source data Fig. 1 [file 44319_2024_354_MOESM2_ESM.zip › Figure 1/1E/Enlarge_KO_addback HA-PD-L1_IR/Merge.jpg]

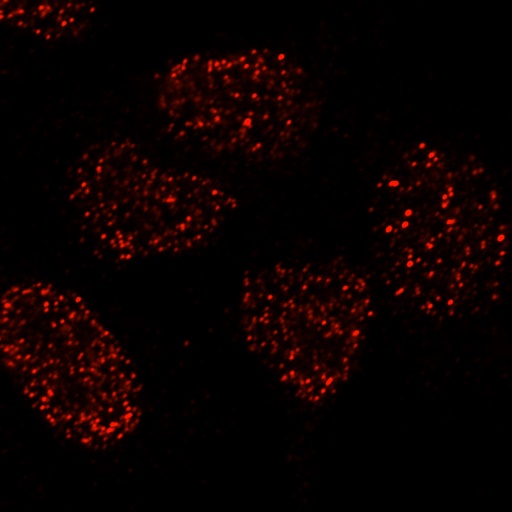

Supplement: Supplementary file 2 — Source data Fig. 1 [file 44319_2024_354_MOESM2_ESM.zip › Figure 1/1E/KO_Addback HA-PD-L1_IR/ATR.jpg]

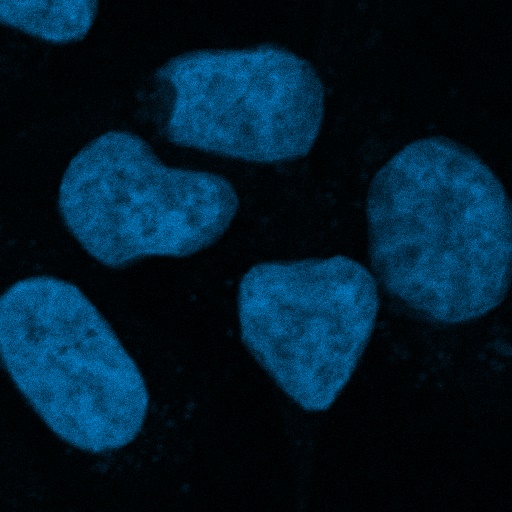

Supplement: Supplementary file 2 — Source data Fig. 1 [file 44319_2024_354_MOESM2_ESM.zip › Figure 1/1E/KO_Addback HA-PD-L1_IR/DAPI.jpg]

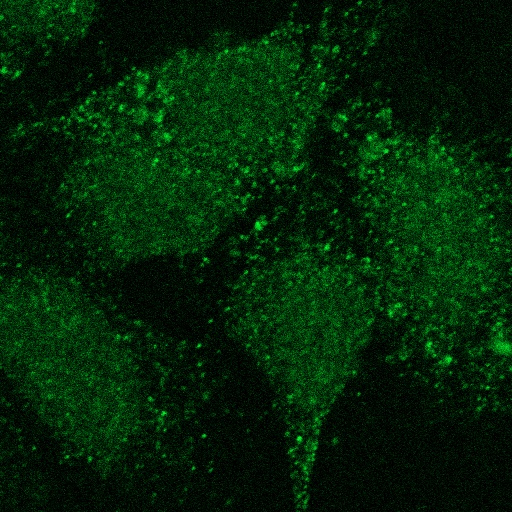

Supplement: Supplementary file 2 — Source data Fig. 1 [file 44319_2024_354_MOESM2_ESM.zip › Figure 1/1E/KO_Addback HA-PD-L1_IR/HA.jpg]

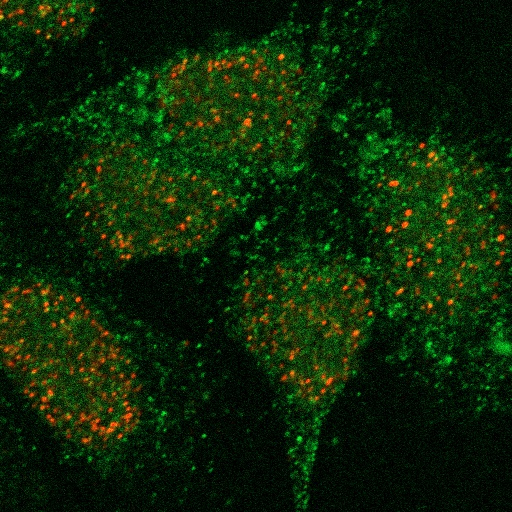

Supplement: Supplementary file 2 — Source data Fig. 1 [file 44319_2024_354_MOESM2_ESM.zip › Figure 1/1E/KO_Addback HA-PD-L1_IR/Merge.jpg]

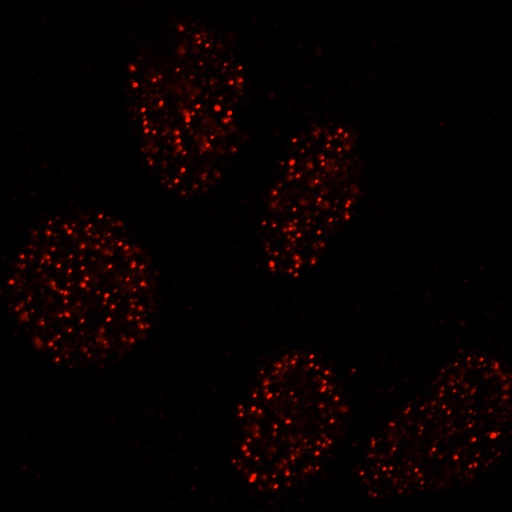

Supplement: Supplementary file 2 — Source data Fig. 1 [file 44319_2024_354_MOESM2_ESM.zip › Figure 1/1E/KO_Addback HA-PD-L1_non-treatment/ATR.jpg]

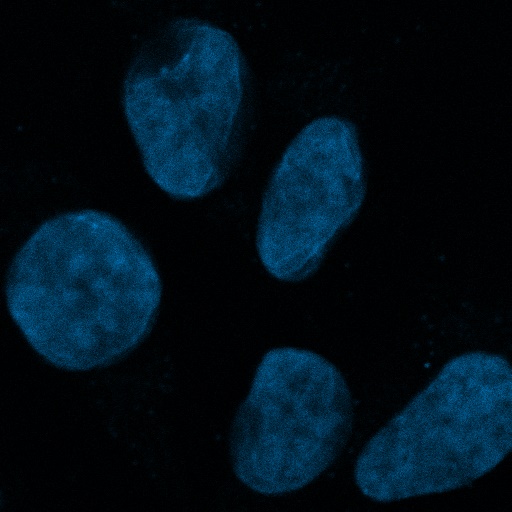

Supplement: Supplementary file 2 — Source data Fig. 1 [file 44319_2024_354_MOESM2_ESM.zip › Figure 1/1E/KO_Addback HA-PD-L1_non-treatment/DAPI.jpg]

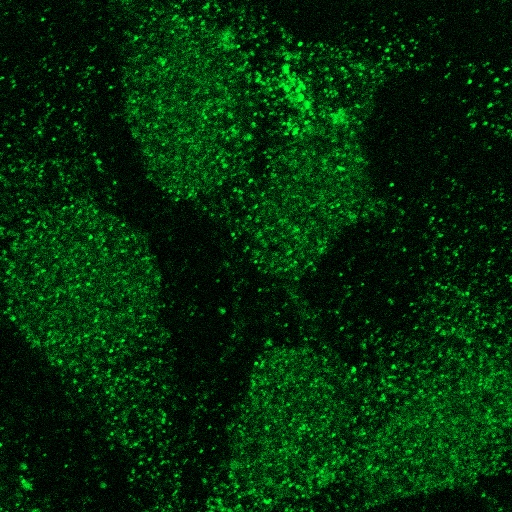

Supplement: Supplementary file 2 — Source data Fig. 1 [file 44319_2024_354_MOESM2_ESM.zip › Figure 1/1E/KO_Addback HA-PD-L1_non-treatment/HA.jpg]

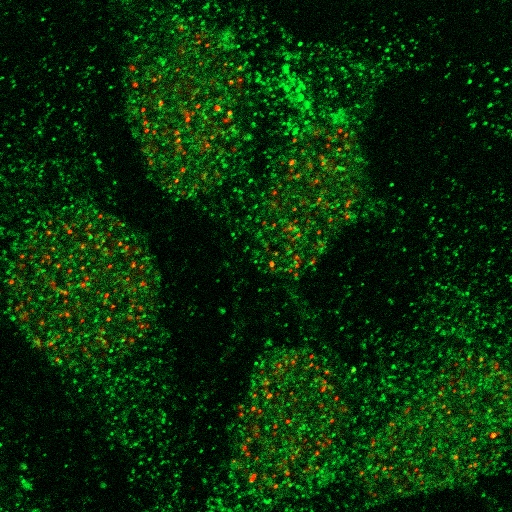

Supplement: Supplementary file 2 — Source data Fig. 1 [file 44319_2024_354_MOESM2_ESM.zip › Figure 1/1E/KO_Addback HA-PD-L1_non-treatment/Merge.jpg]

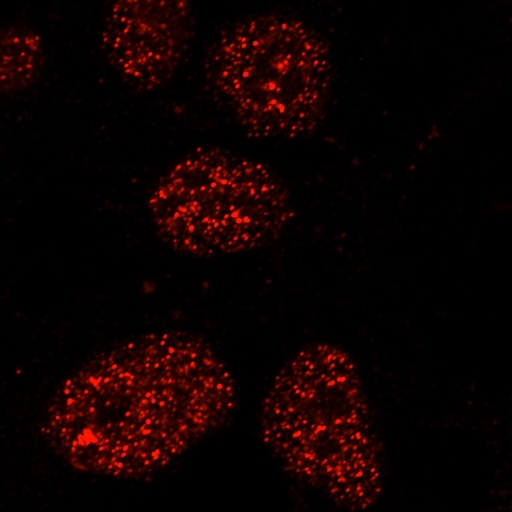

Supplement: Supplementary file 2 — Source data Fig. 1 [file 44319_2024_354_MOESM2_ESM.zip › Figure 1/1E/KO_non-treatment/ATR.jpg]

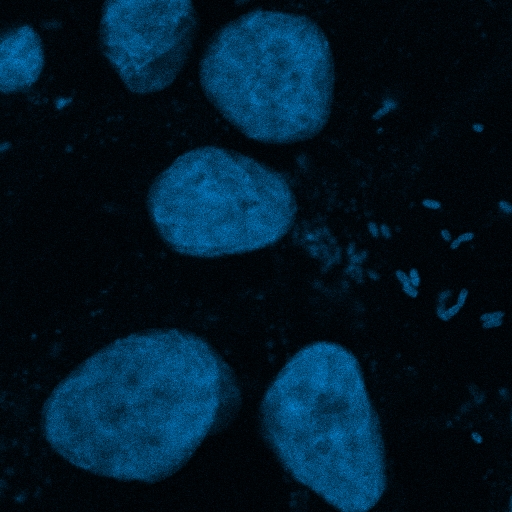

Supplement: Supplementary file 2 — Source data Fig. 1 [file 44319_2024_354_MOESM2_ESM.zip › Figure 1/1E/KO_non-treatment/DAPI.jpg]

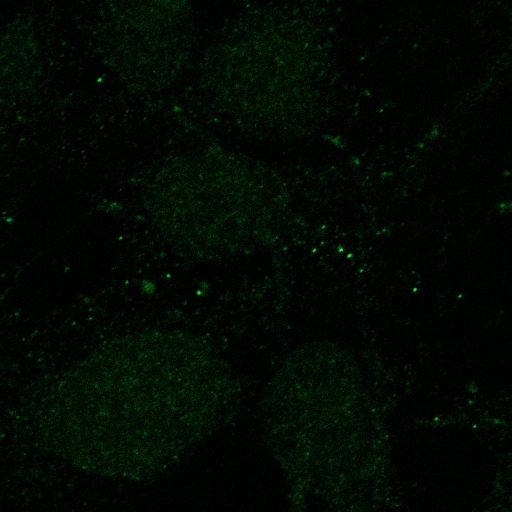

Supplement: Supplementary file 2 — Source data Fig. 1 [file 44319_2024_354_MOESM2_ESM.zip › Figure 1/1E/KO_non-treatment/HA.jpg]

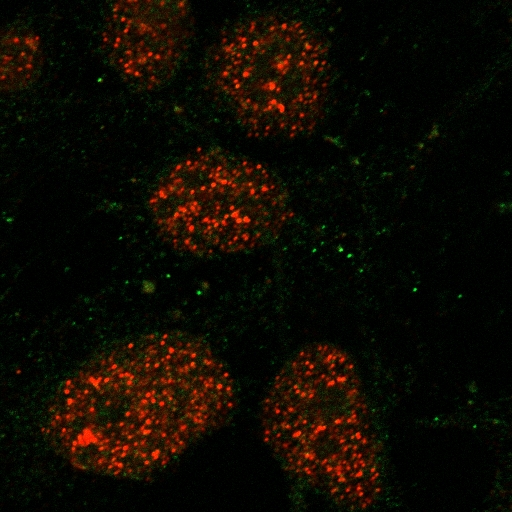

Supplement: Supplementary file 2 — Source data Fig. 1 [file 44319_2024_354_MOESM2_ESM.zip › Figure 1/1E/KO_non-treatment/Merge.jpg]

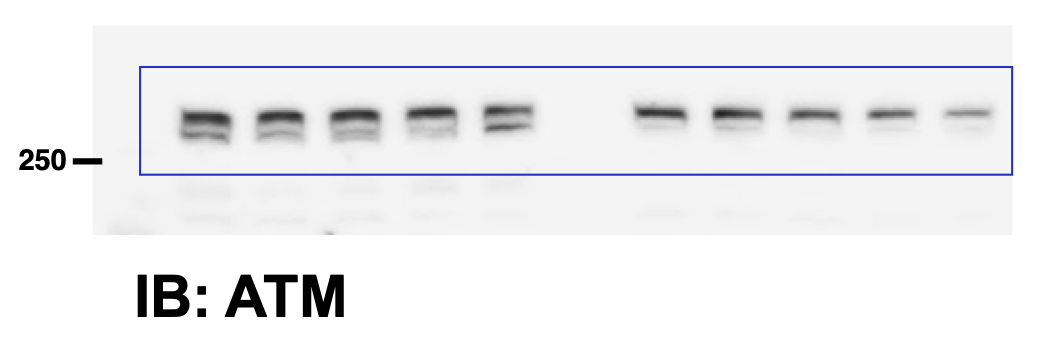

Supplement: Supplementary file 3 — Source data Fig. 2 [file 44319_2024_354_MOESM3_ESM.zip › Figure 2/2A/ATM.tif]

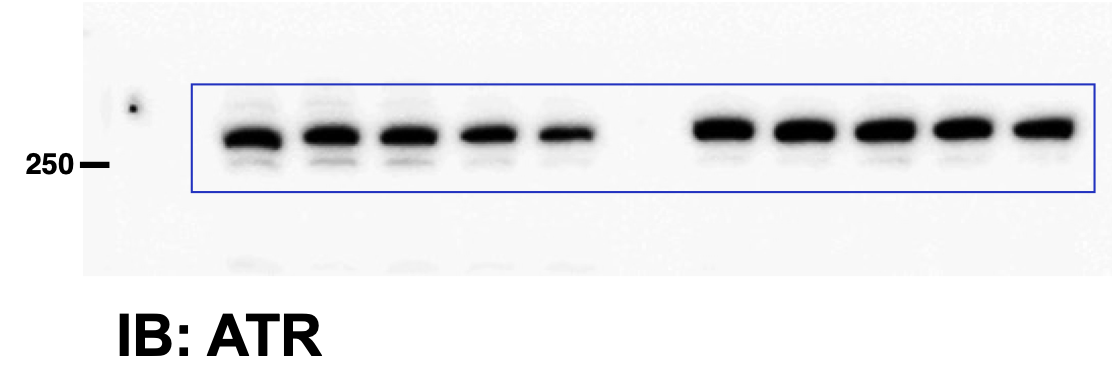

Supplement: Supplementary file 3 — Source data Fig. 2 [file 44319_2024_354_MOESM3_ESM.zip › Figure 2/2A/ATR.tif]

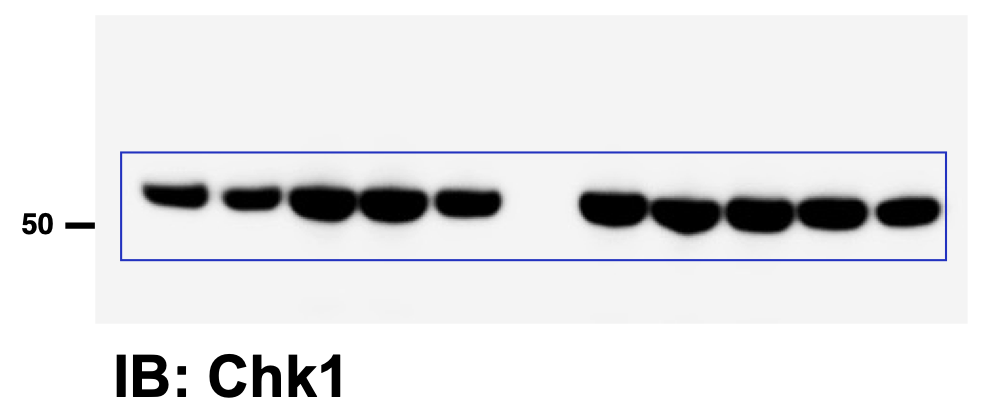

Supplement: Supplementary file 3 — Source data Fig. 2 [file 44319_2024_354_MOESM3_ESM.zip › Figure 2/2A/Chk1.tif]

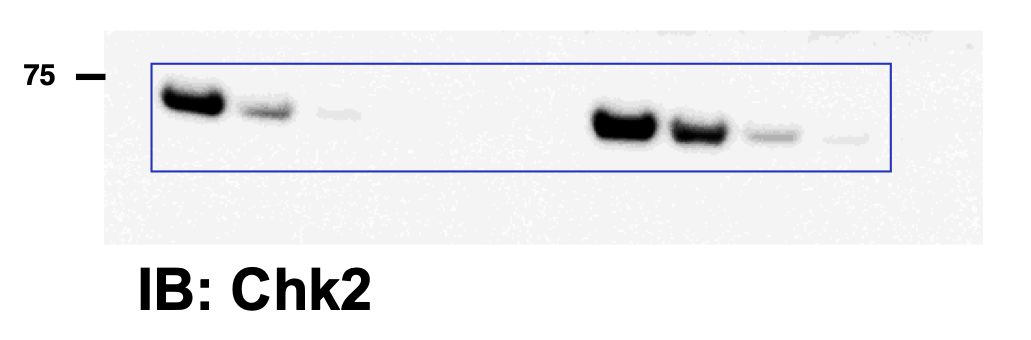

Supplement: Supplementary file 3 — Source data Fig. 2 [file 44319_2024_354_MOESM3_ESM.zip › Figure 2/2A/Chk2.tif]

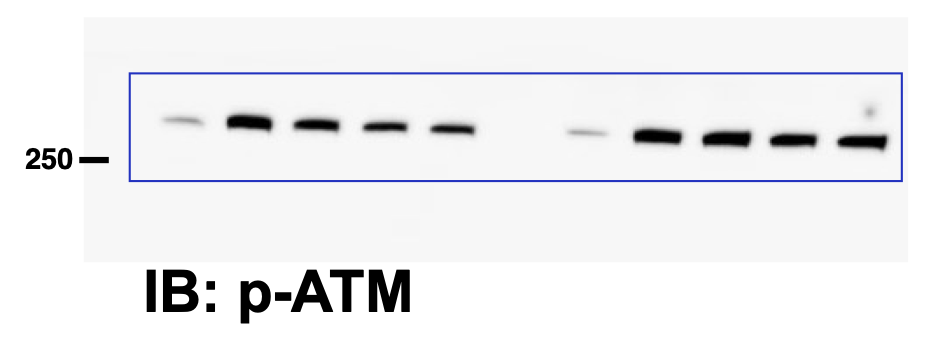

Supplement: Supplementary file 3 — Source data Fig. 2 [file 44319_2024_354_MOESM3_ESM.zip › Figure 2/2A/pATM.tif]

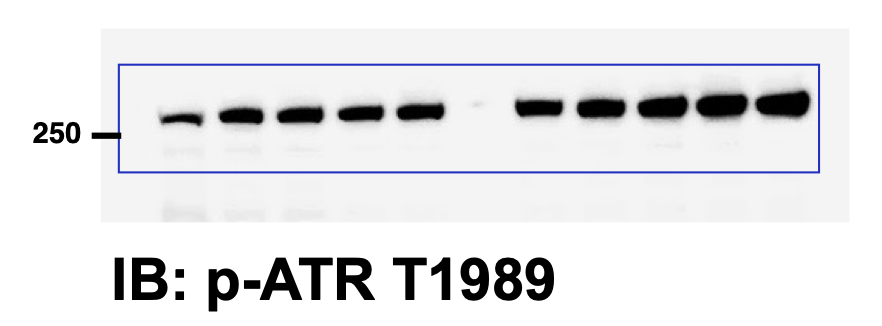

Supplement: Supplementary file 3 — Source data Fig. 2 [file 44319_2024_354_MOESM3_ESM.zip › Figure 2/2A/pATR.tif]

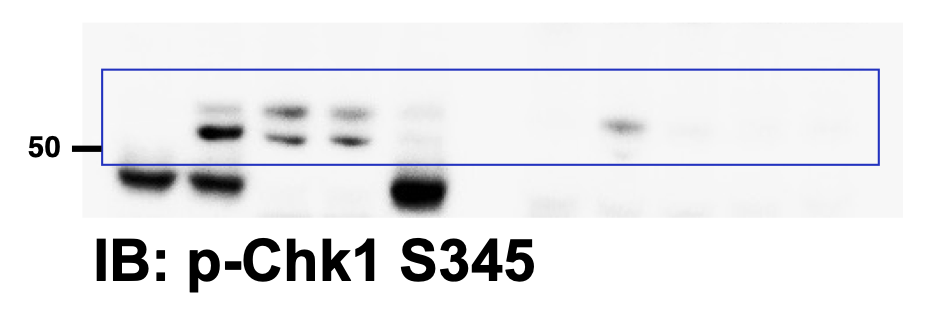

Supplement: Supplementary file 3 — Source data Fig. 2 [file 44319_2024_354_MOESM3_ESM.zip › Figure 2/2A/pChk1.tif]

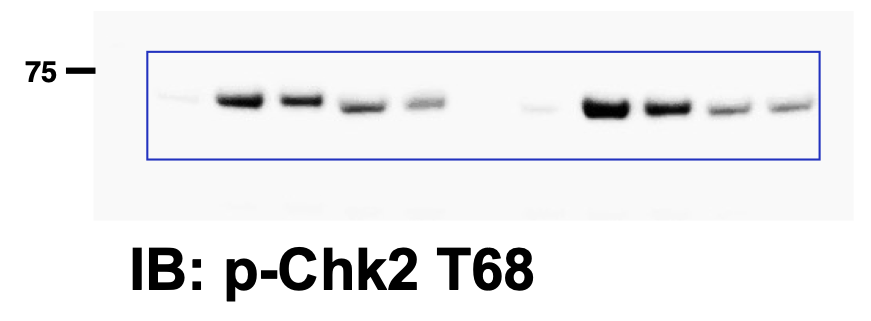

Supplement: Supplementary file 3 — Source data Fig. 2 [file 44319_2024_354_MOESM3_ESM.zip › Figure 2/2A/pChk2.tif]

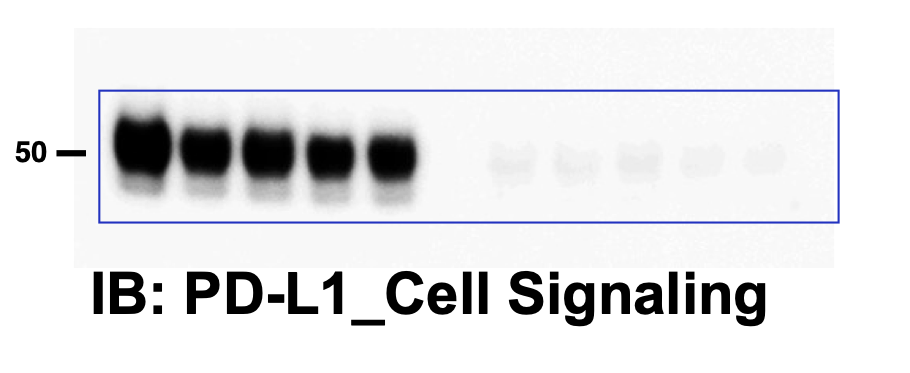

Supplement: Supplementary file 3 — Source data Fig. 2 [file 44319_2024_354_MOESM3_ESM.zip › Figure 2/2A/PD-L1_CST.tif]

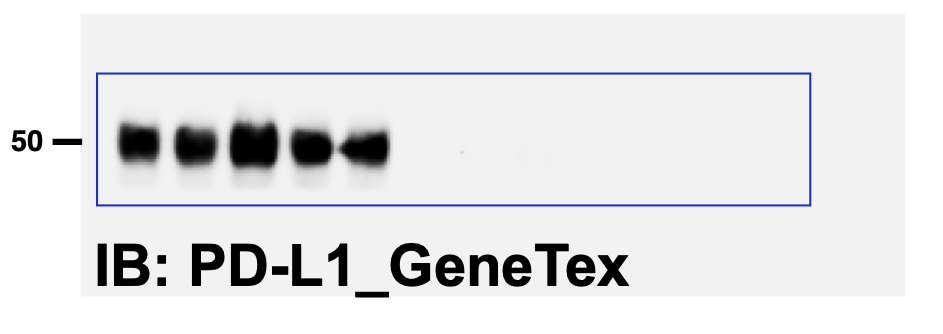

Supplement: Supplementary file 3 — Source data Fig. 2 [file 44319_2024_354_MOESM3_ESM.zip › Figure 2/2A/PD-L1_GeneTex.tif]

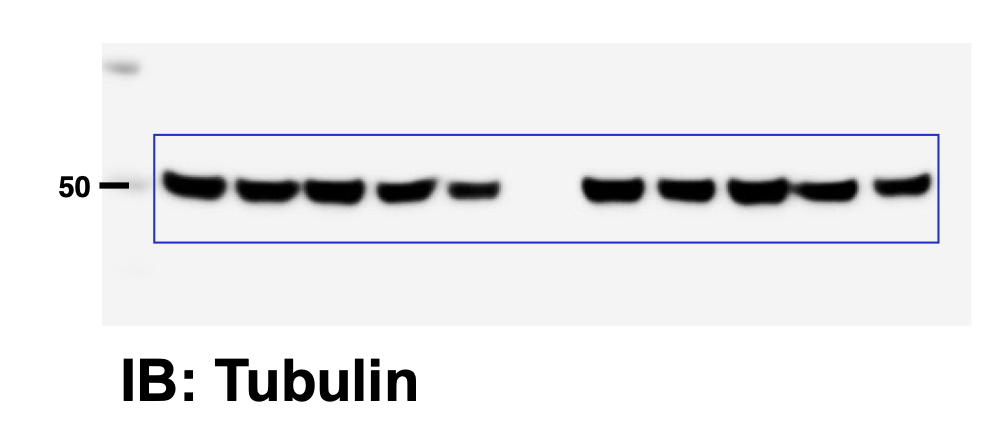

Supplement: Supplementary file 3 — Source data Fig. 2 [file 44319_2024_354_MOESM3_ESM.zip › Figure 2/2A/Tubulin.tif]

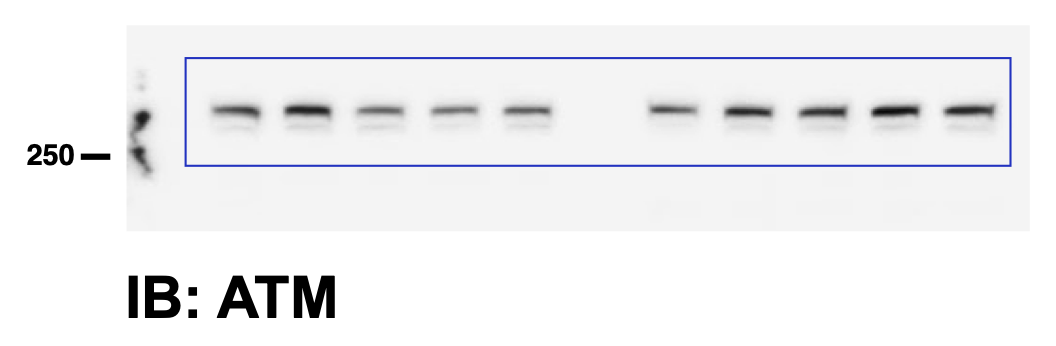

Supplement: Supplementary file 3 — Source data Fig. 2 [file 44319_2024_354_MOESM3_ESM.zip › Figure 2/2B/ATM.tif]

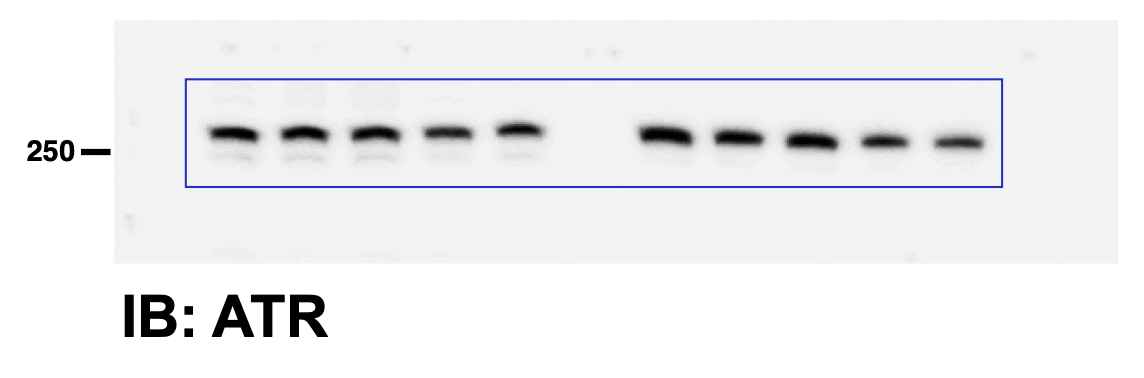

Supplement: Supplementary file 3 — Source data Fig. 2 [file 44319_2024_354_MOESM3_ESM.zip › Figure 2/2B/ATR.tif]

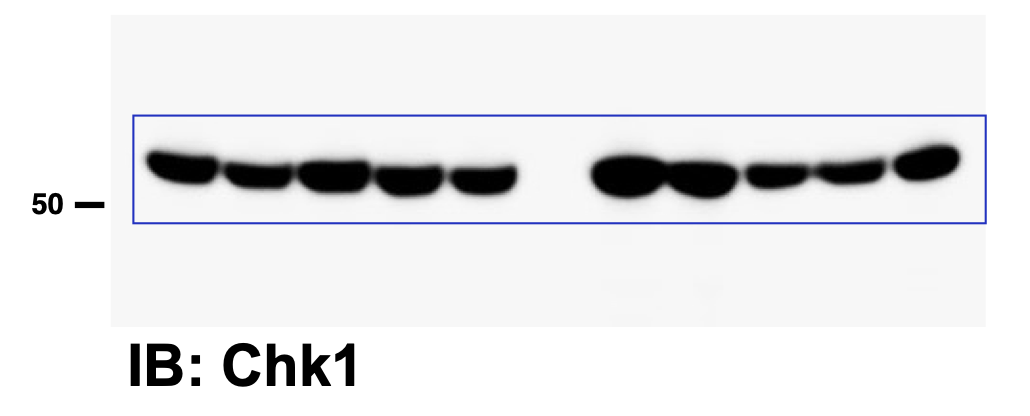

Supplement: Supplementary file 3 — Source data Fig. 2 [file 44319_2024_354_MOESM3_ESM.zip › Figure 2/2B/Chk1.tif]

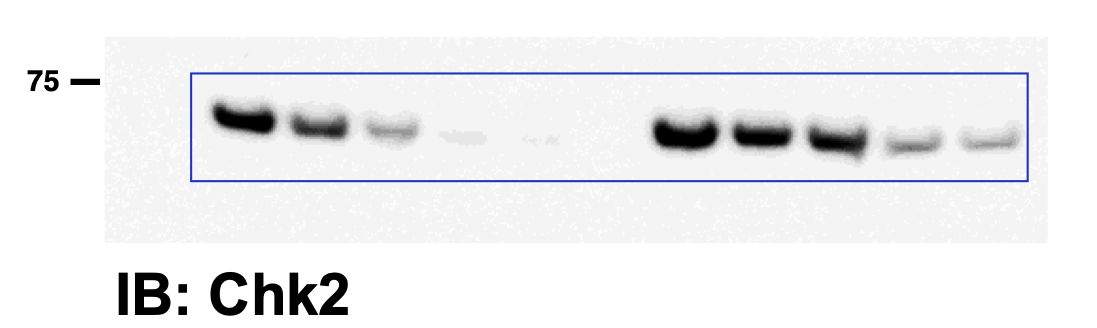

Supplement: Supplementary file 3 — Source data Fig. 2 [file 44319_2024_354_MOESM3_ESM.zip › Figure 2/2B/Chk2.tif]

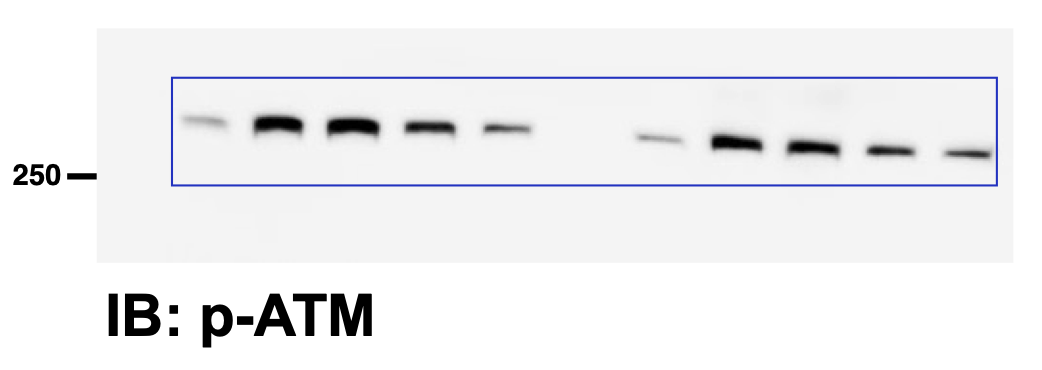

Supplement: Supplementary file 3 — Source data Fig. 2 [file 44319_2024_354_MOESM3_ESM.zip › Figure 2/2B/pATM.tif]

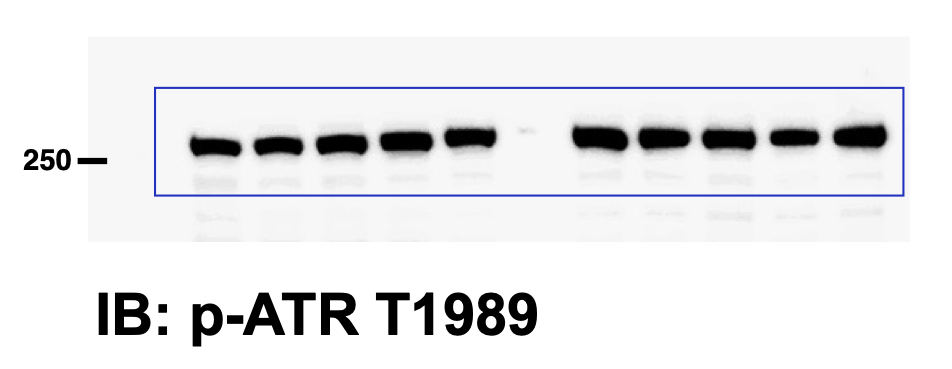

Supplement: Supplementary file 3 — Source data Fig. 2 [file 44319_2024_354_MOESM3_ESM.zip › Figure 2/2B/pATR.tif]

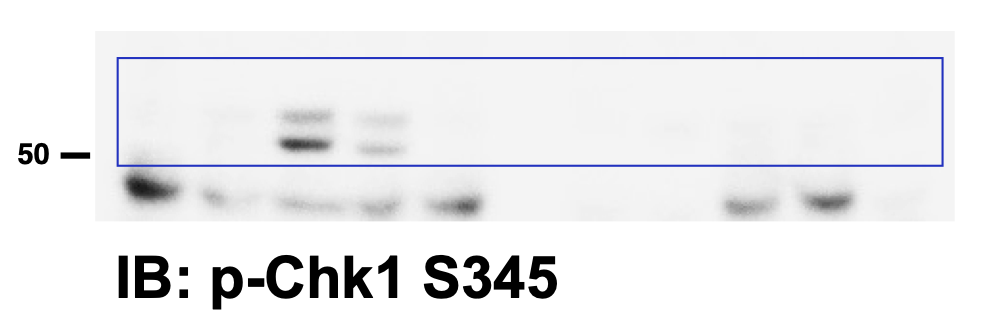

Supplement: Supplementary file 3 — Source data Fig. 2 [file 44319_2024_354_MOESM3_ESM.zip › Figure 2/2B/pChk1.tif]

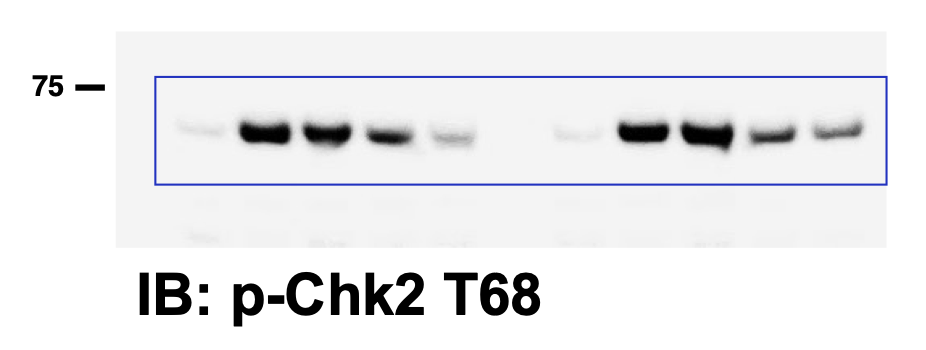

Supplement: Supplementary file 3 — Source data Fig. 2 [file 44319_2024_354_MOESM3_ESM.zip › Figure 2/2B/pChk2.tif]

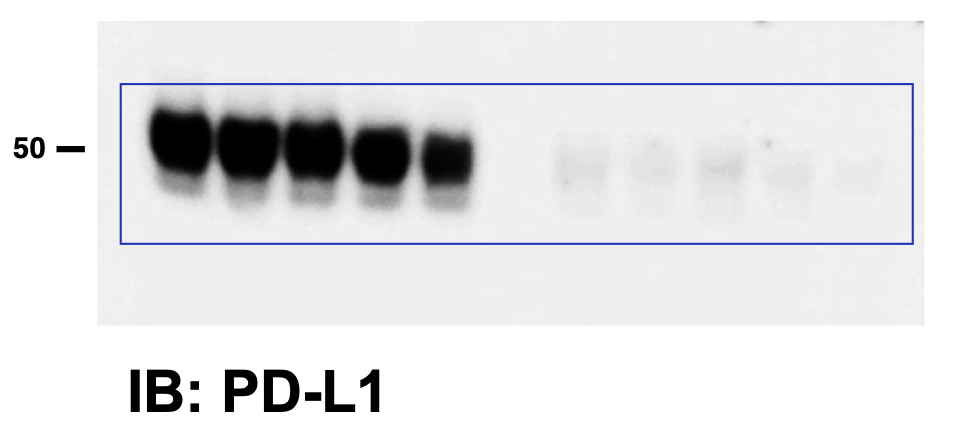

Supplement: Supplementary file 3 — Source data Fig. 2 [file 44319_2024_354_MOESM3_ESM.zip › Figure 2/2B/PD-L1.tif]

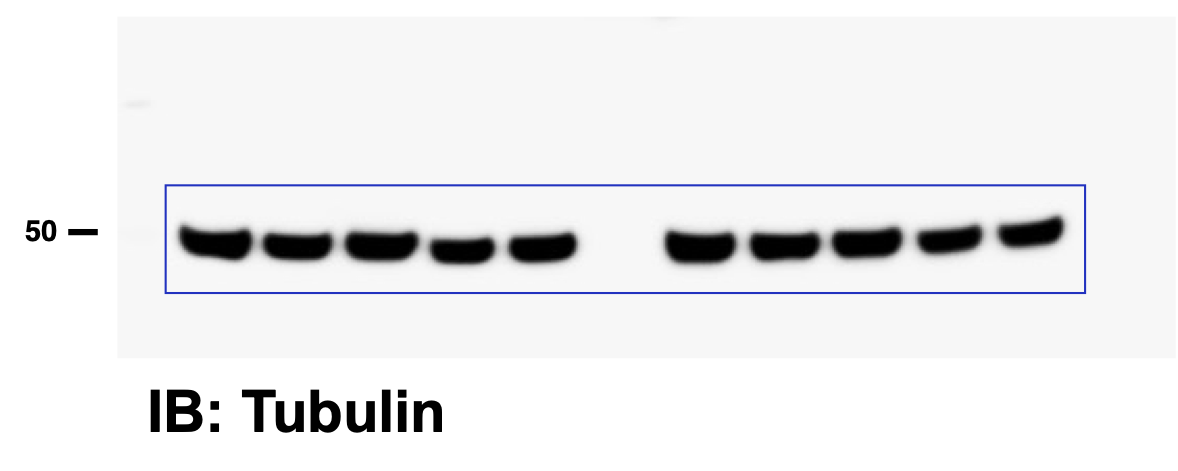

Supplement: Supplementary file 3 — Source data Fig. 2 [file 44319_2024_354_MOESM3_ESM.zip › Figure 2/2B/Tubulin.tif]

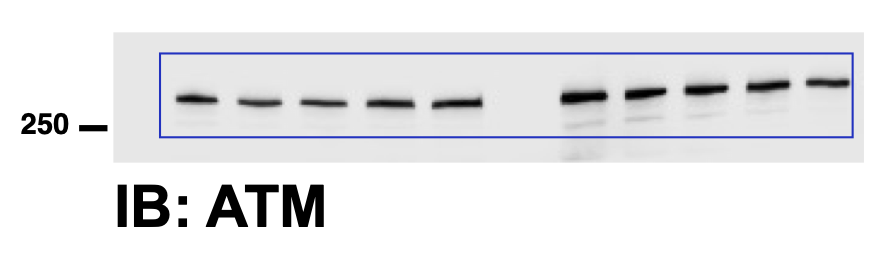

Supplement: Supplementary file 3 — Source data Fig. 2 [file 44319_2024_354_MOESM3_ESM.zip › Figure 2/2C/ATM.tif]

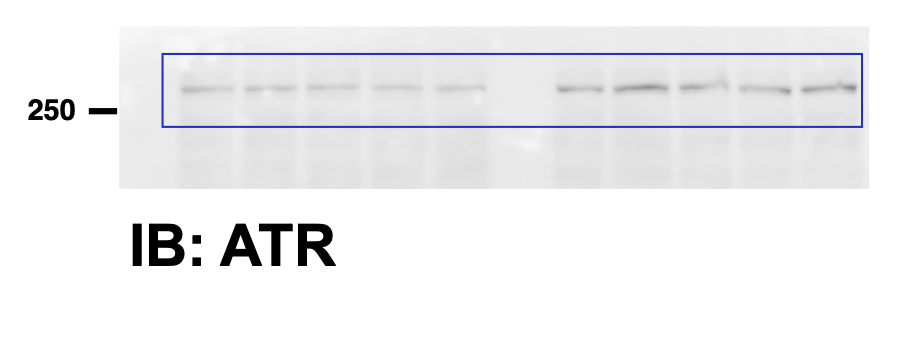

Supplement: Supplementary file 3 — Source data Fig. 2 [file 44319_2024_354_MOESM3_ESM.zip › Figure 2/2C/ATR.tif]

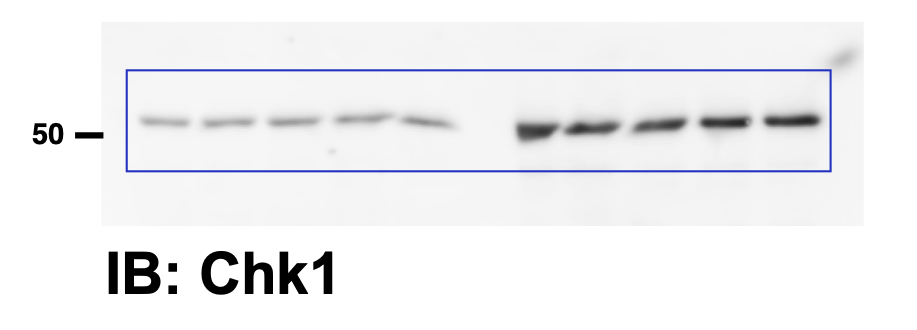

Supplement: Supplementary file 3 — Source data Fig. 2 [file 44319_2024_354_MOESM3_ESM.zip › Figure 2/2C/Chk1.tif]

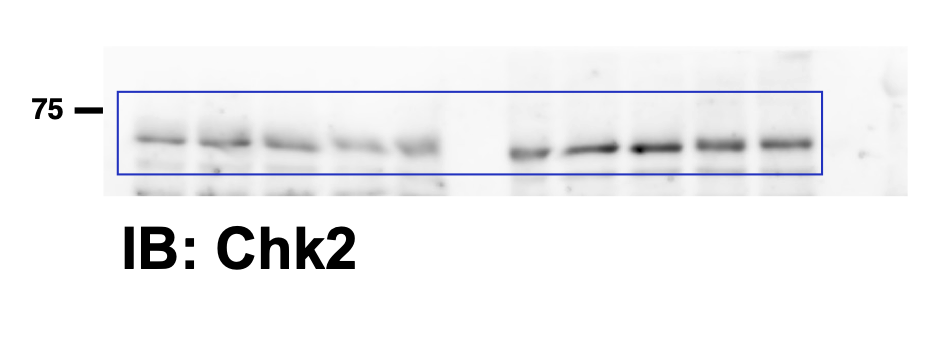

Supplement: Supplementary file 3 — Source data Fig. 2 [file 44319_2024_354_MOESM3_ESM.zip › Figure 2/2C/Chk2.tif]

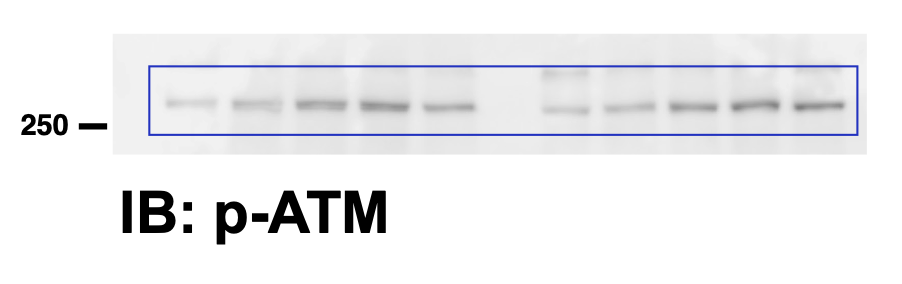

Supplement: Supplementary file 3 — Source data Fig. 2 [file 44319_2024_354_MOESM3_ESM.zip › Figure 2/2C/pATM.tif]

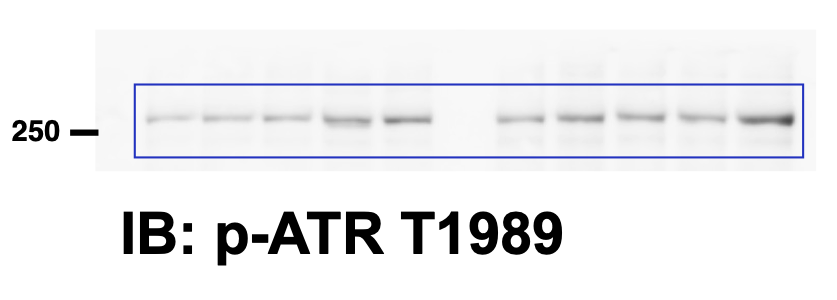

Supplement: Supplementary file 3 — Source data Fig. 2 [file 44319_2024_354_MOESM3_ESM.zip › Figure 2/2C/pATR.tif]

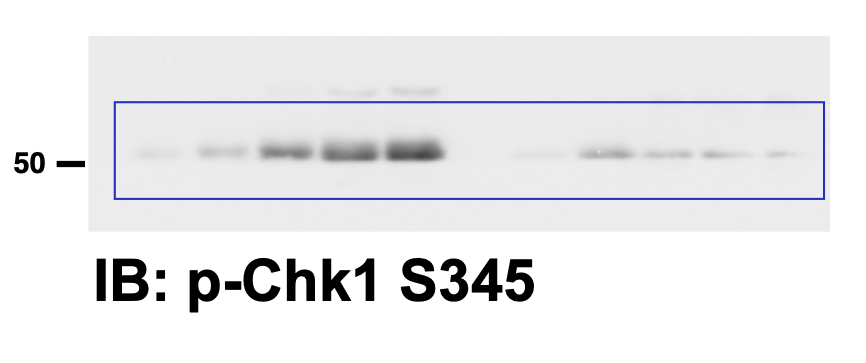

Supplement: Supplementary file 3 — Source data Fig. 2 [file 44319_2024_354_MOESM3_ESM.zip › Figure 2/2C/pChk1.tif]

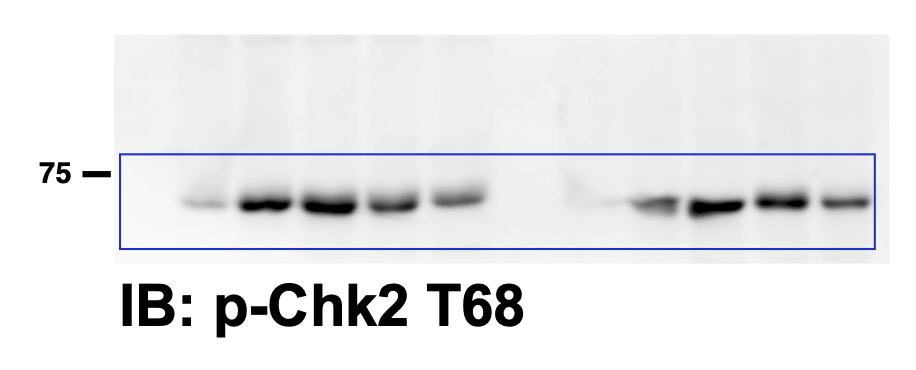

Supplement: Supplementary file 3 — Source data Fig. 2 [file 44319_2024_354_MOESM3_ESM.zip › Figure 2/2C/pChk2.tif]

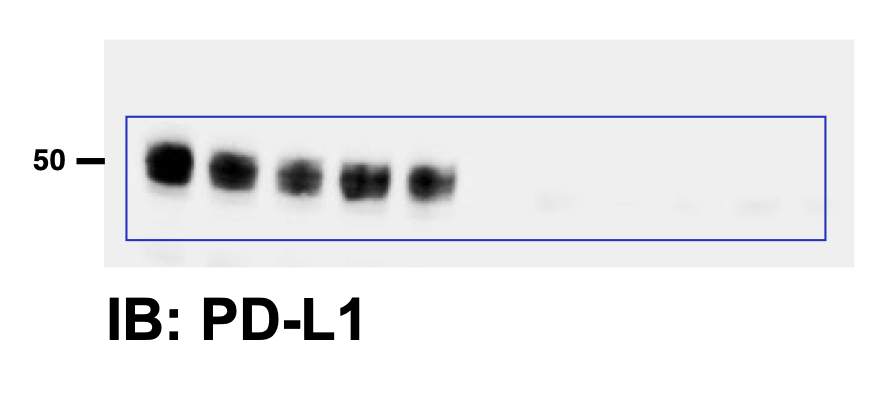

Supplement: Supplementary file 3 — Source data Fig. 2 [file 44319_2024_354_MOESM3_ESM.zip › Figure 2/2C/PD-L1.tif]

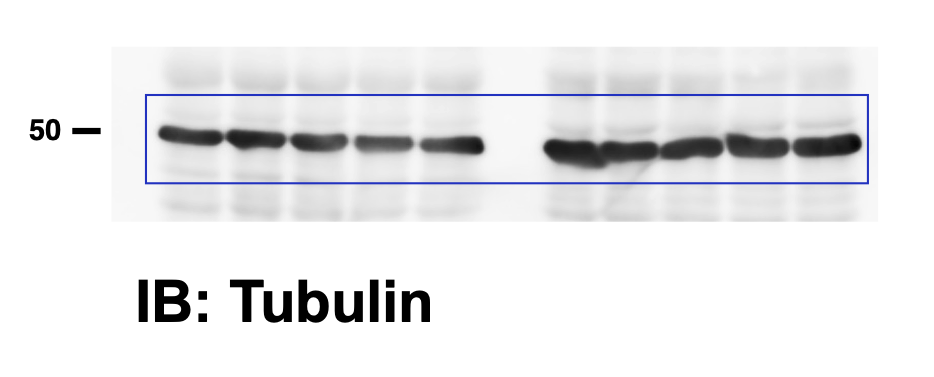

Supplement: Supplementary file 3 — Source data Fig. 2 [file 44319_2024_354_MOESM3_ESM.zip › Figure 2/2C/Tubulin.tif]

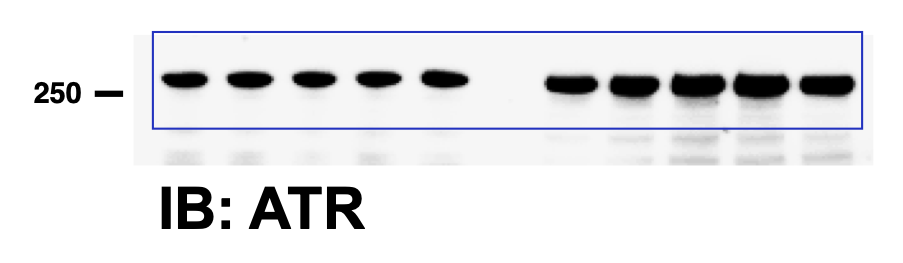

Supplement: Supplementary file 3 — Source data Fig. 2 [file 44319_2024_354_MOESM3_ESM.zip › Figure 2/2D/ATR.tif]

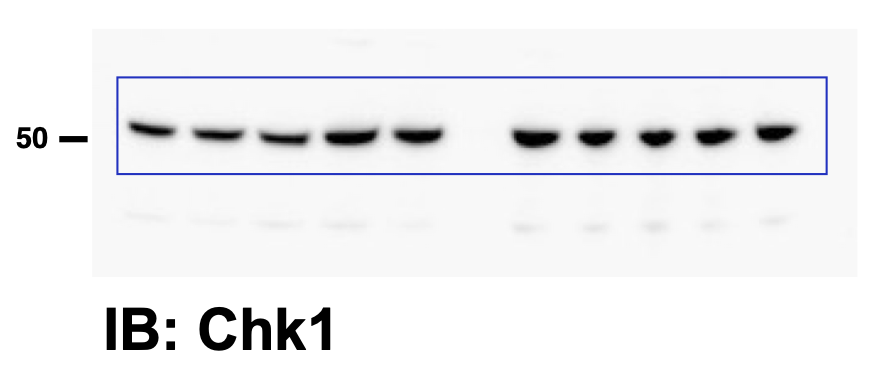

Supplement: Supplementary file 3 — Source data Fig. 2 [file 44319_2024_354_MOESM3_ESM.zip › Figure 2/2D/Chk1.tif]

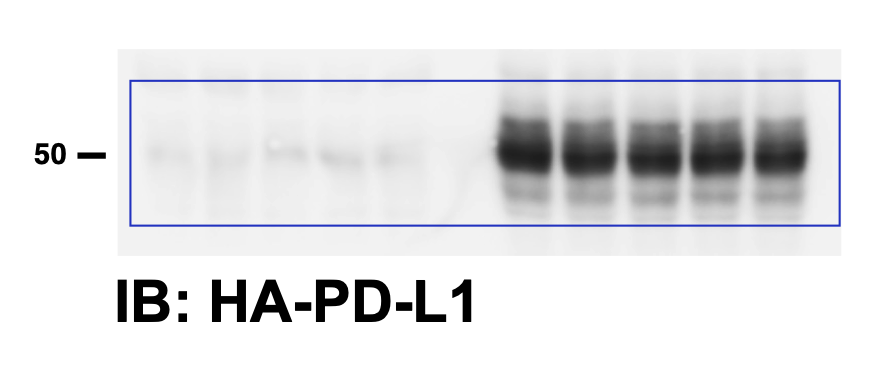

Supplement: Supplementary file 3 — Source data Fig. 2 [file 44319_2024_354_MOESM3_ESM.zip › Figure 2/2D/HA-PD-L1.tif]

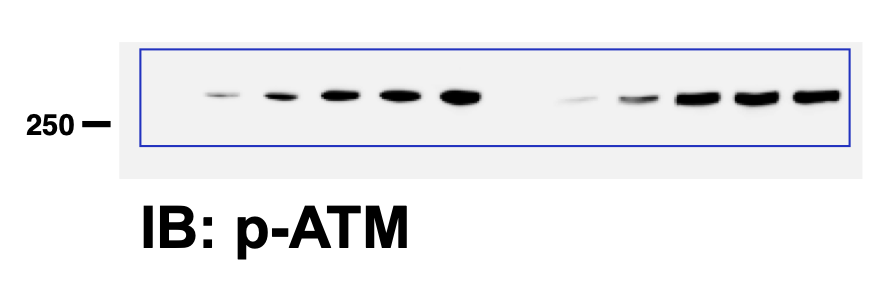

Supplement: Supplementary file 3 — Source data Fig. 2 [file 44319_2024_354_MOESM3_ESM.zip › Figure 2/2D/pATM.tif]

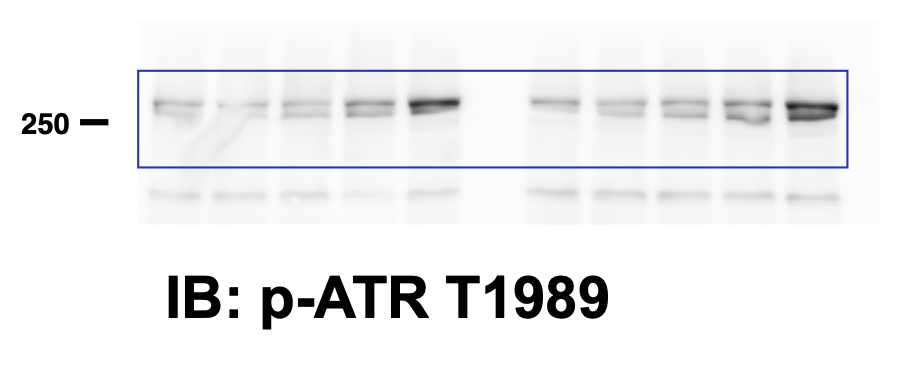

Supplement: Supplementary file 3 — Source data Fig. 2 [file 44319_2024_354_MOESM3_ESM.zip › Figure 2/2D/pATR.tif]

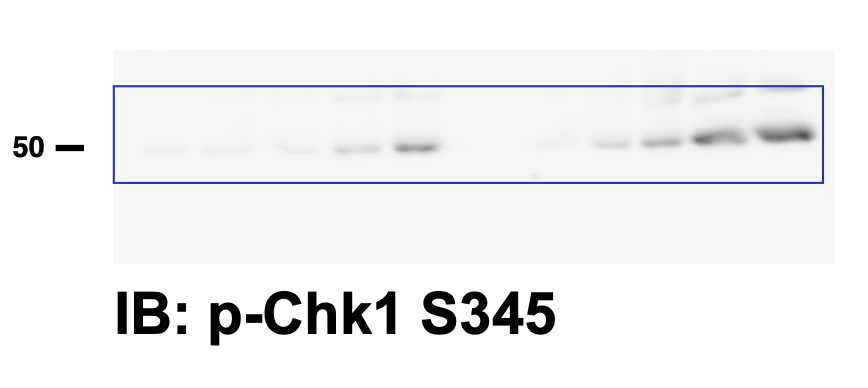

Supplement: Supplementary file 3 — Source data Fig. 2 [file 44319_2024_354_MOESM3_ESM.zip › Figure 2/2D/pChk1.tif]

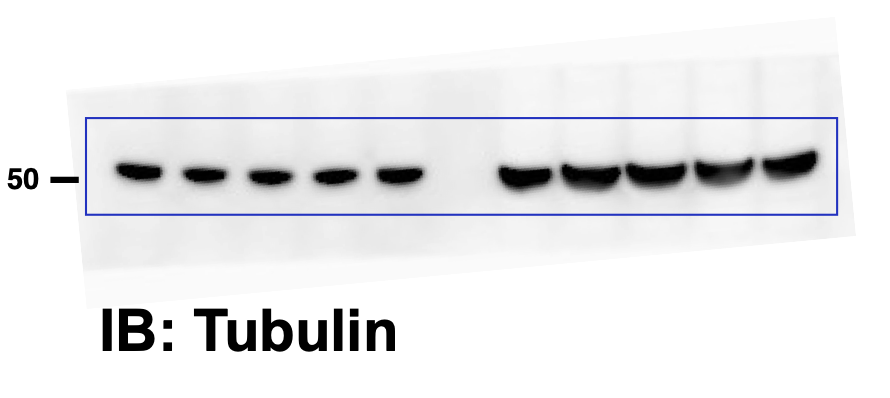

Supplement: Supplementary file 3 — Source data Fig. 2 [file 44319_2024_354_MOESM3_ESM.zip › Figure 2/2D/Tubulin.tif]

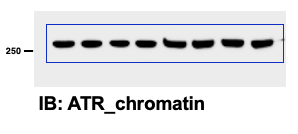

Supplement: Supplementary file 3 — Source data Fig. 2 [file 44319_2024_354_MOESM3_ESM.zip › Figure 2/2F/ATR_chromatin.tif]

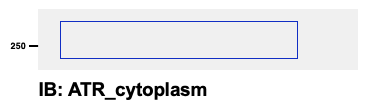

Supplement: Supplementary file 3 — Source data Fig. 2 [file 44319_2024_354_MOESM3_ESM.zip › Figure 2/2F/ATR_cytoplasm.tif]

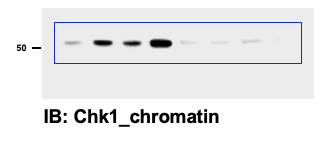

Supplement: Supplementary file 3 — Source data Fig. 2 [file 44319_2024_354_MOESM3_ESM.zip › Figure 2/2F/Chk1_Chromatin.tif]

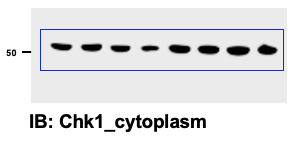

Supplement: Supplementary file 3 — Source data Fig. 2 [file 44319_2024_354_MOESM3_ESM.zip › Figure 2/2F/Chk1_cytoplasm.tif]

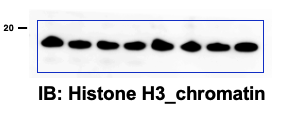

Supplement: Supplementary file 3 — Source data Fig. 2 [file 44319_2024_354_MOESM3_ESM.zip › Figure 2/2F/Histone H3_chromatin.tif]

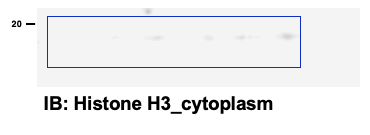

Supplement: Supplementary file 3 — Source data Fig. 2 [file 44319_2024_354_MOESM3_ESM.zip › Figure 2/2F/Histone H3_cytoplasm.tif]

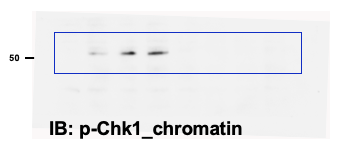

Supplement: Supplementary file 3 — Source data Fig. 2 [file 44319_2024_354_MOESM3_ESM.zip › Figure 2/2F/pChk1_chromatin.tif]

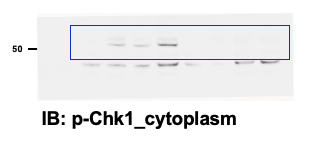

Supplement: Supplementary file 3 — Source data Fig. 2 [file 44319_2024_354_MOESM3_ESM.zip › Figure 2/2F/pChk1_cytoplasm.tif]

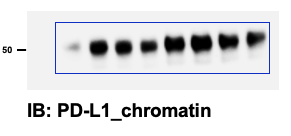

Supplement: Supplementary file 3 — Source data Fig. 2 [file 44319_2024_354_MOESM3_ESM.zip › Figure 2/2F/PD-L1_chromatin.tif]

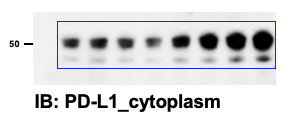

Supplement: Supplementary file 3 — Source data Fig. 2 [file 44319_2024_354_MOESM3_ESM.zip › Figure 2/2F/PD-L1_cytoplasm.tif]

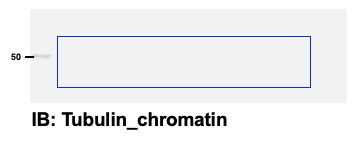

Supplement: Supplementary file 3 — Source data Fig. 2 [file 44319_2024_354_MOESM3_ESM.zip › Figure 2/2F/Tubulin_chromatin.tif]

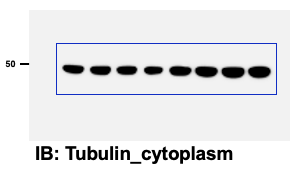

Supplement: Supplementary file 3 — Source data Fig. 2 [file 44319_2024_354_MOESM3_ESM.zip › Figure 2/2F/Tubulin_cytolasm.tif]

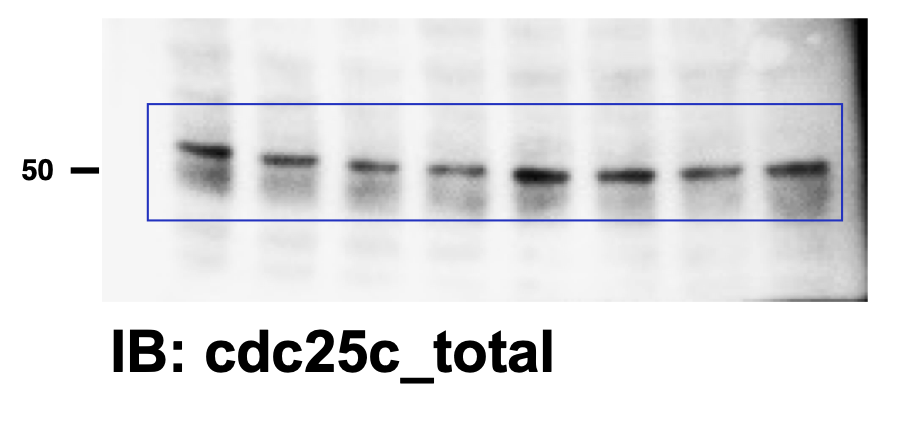

Supplement: Supplementary file 3 — Source data Fig. 2 [file 44319_2024_354_MOESM3_ESM.zip › Figure 2/2G/cdc25_total lysate.tif]

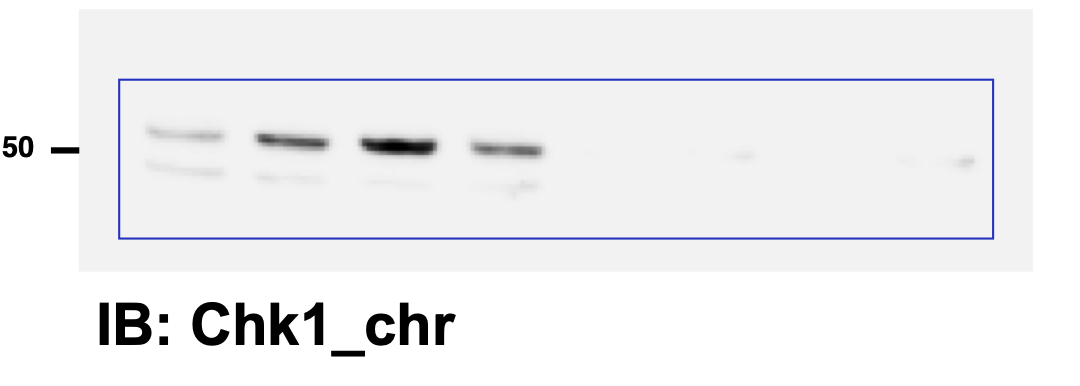

Supplement: Supplementary file 3 — Source data Fig. 2 [file 44319_2024_354_MOESM3_ESM.zip › Figure 2/2G/Chk1_chromatin.tif]

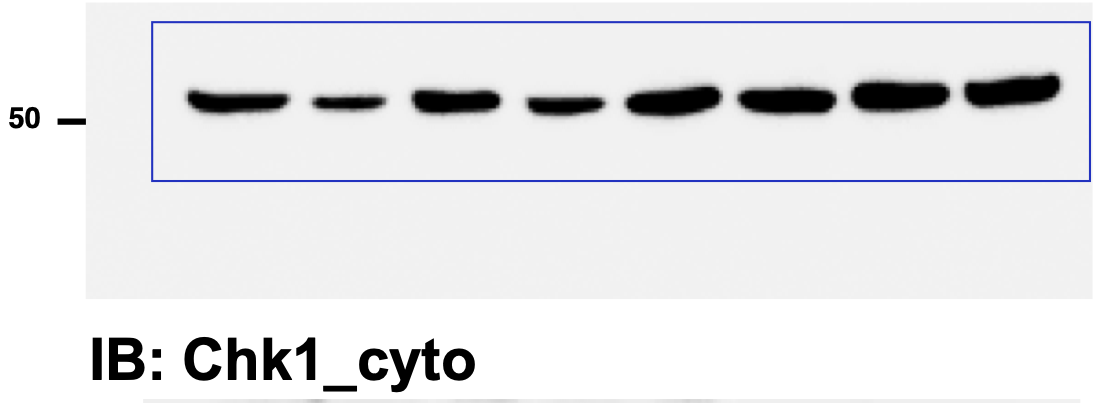

Supplement: Supplementary file 3 — Source data Fig. 2 [file 44319_2024_354_MOESM3_ESM.zip › Figure 2/2G/Chk1_cytoplasm.tif]

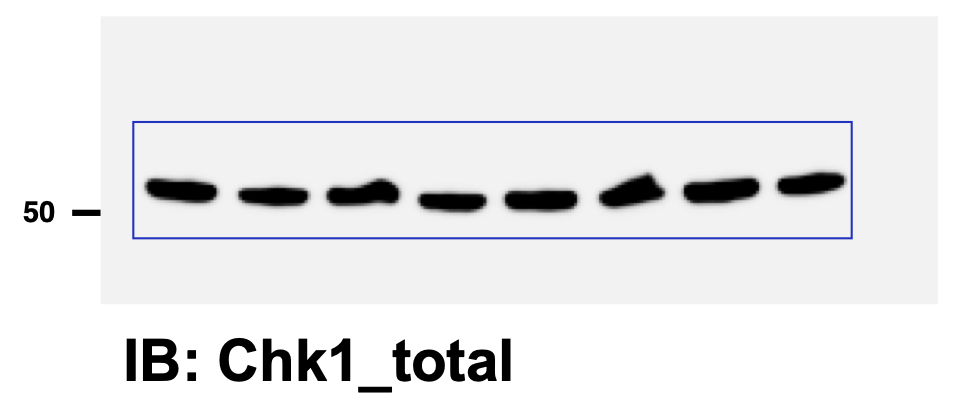

Supplement: Supplementary file 3 — Source data Fig. 2 [file 44319_2024_354_MOESM3_ESM.zip › Figure 2/2G/Chk1_total lysate.tif]

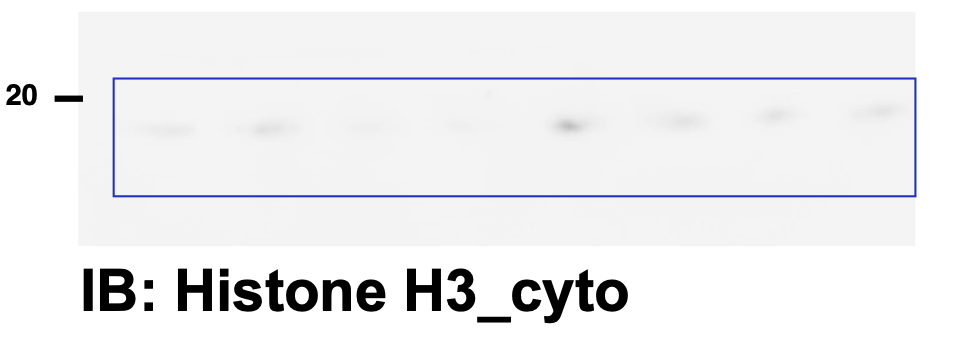

Supplement: Supplementary file 3 — Source data Fig. 2 [file 44319_2024_354_MOESM3_ESM.zip › Figure 2/2G/Hisotone H3_cytoplasm.tif]

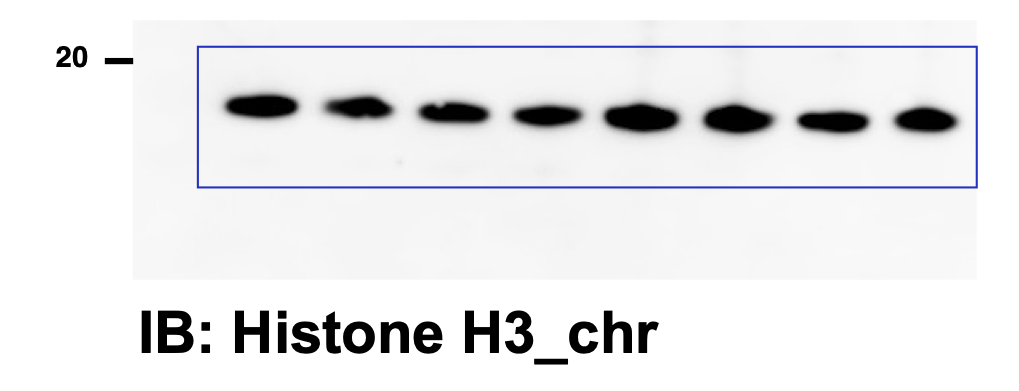

Supplement: Supplementary file 3 — Source data Fig. 2 [file 44319_2024_354_MOESM3_ESM.zip › Figure 2/2G/Histone H3_chromatin.tif]

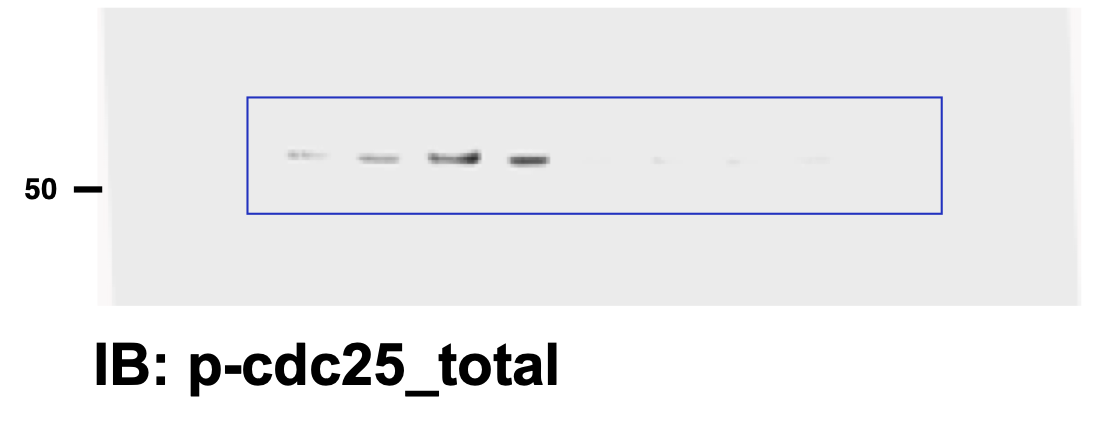

Supplement: Supplementary file 3 — Source data Fig. 2 [file 44319_2024_354_MOESM3_ESM.zip › Figure 2/2G/p-cdc25_total lysate.tif]

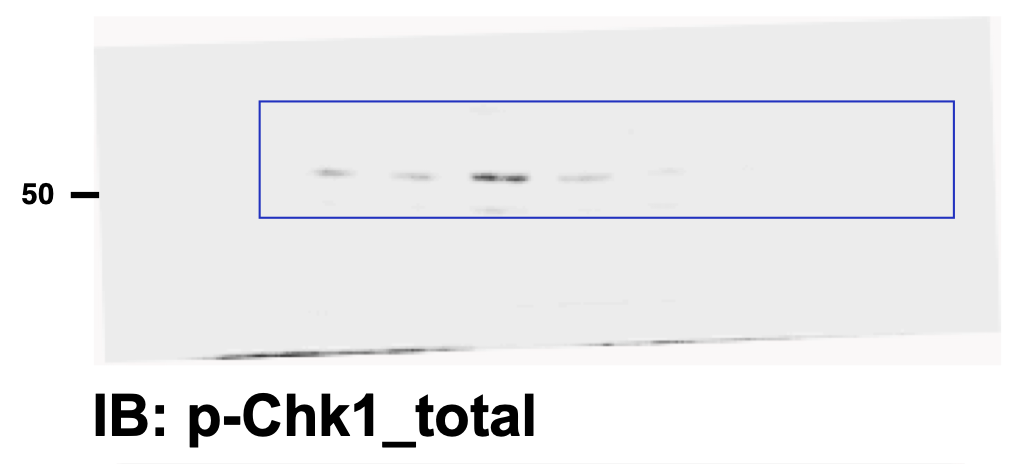

Supplement: Supplementary file 3 — Source data Fig. 2 [file 44319_2024_354_MOESM3_ESM.zip › Figure 2/2G/pChk1_total lysate.tif]
