# Supplementary figures and images for: Nuclear PD-L1 triggers tumour-associated inflammation upon DNA damage (part 2 of 3)
Source: EMBO Rep. 2025 Jan 2;26(3):635–55. doi: 10.1038/s44319-024-00354-9 (PMC11811057; doi:10.1038/s44319-024-00354-9)

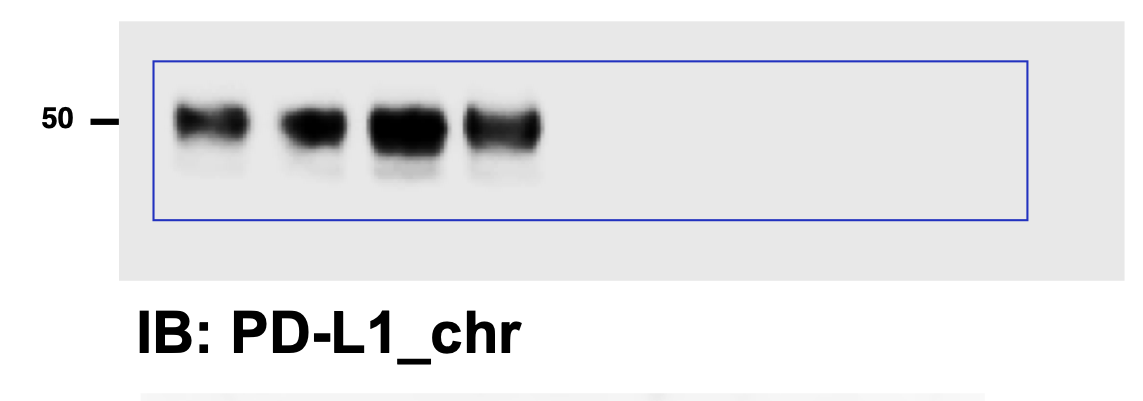

Supplement: Supplementary file 3 — Source data Fig. 2 [file 44319_2024_354_MOESM3_ESM.zip › Figure 2/2G/PD-L1_chromatin.tif]

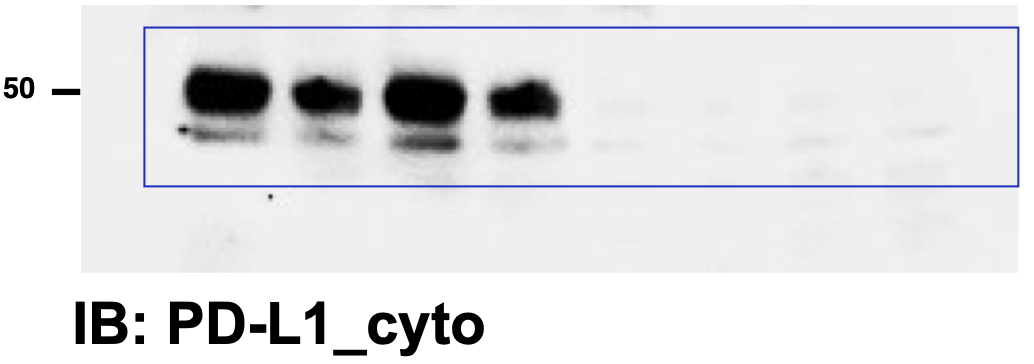

Supplement: Supplementary file 3 — Source data Fig. 2 [file 44319_2024_354_MOESM3_ESM.zip › Figure 2/2G/PD-L1_cytoplasm.tif]

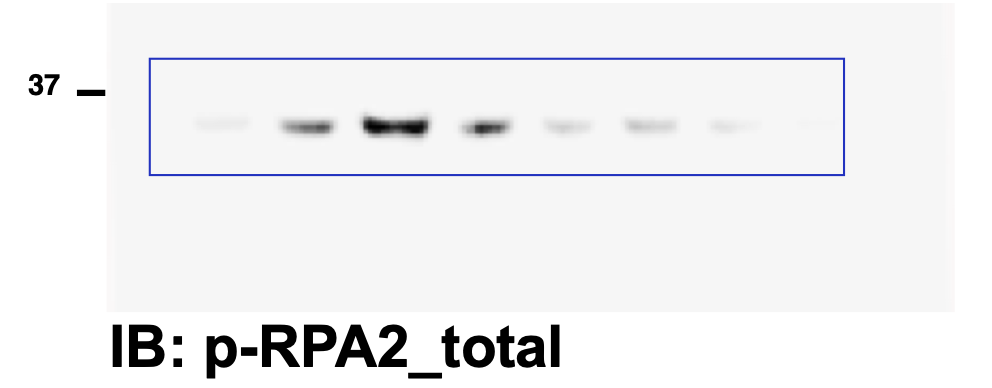

Supplement: Supplementary file 3 — Source data Fig. 2 [file 44319_2024_354_MOESM3_ESM.zip › Figure 2/2G/pRPA2_total lysate.tif]

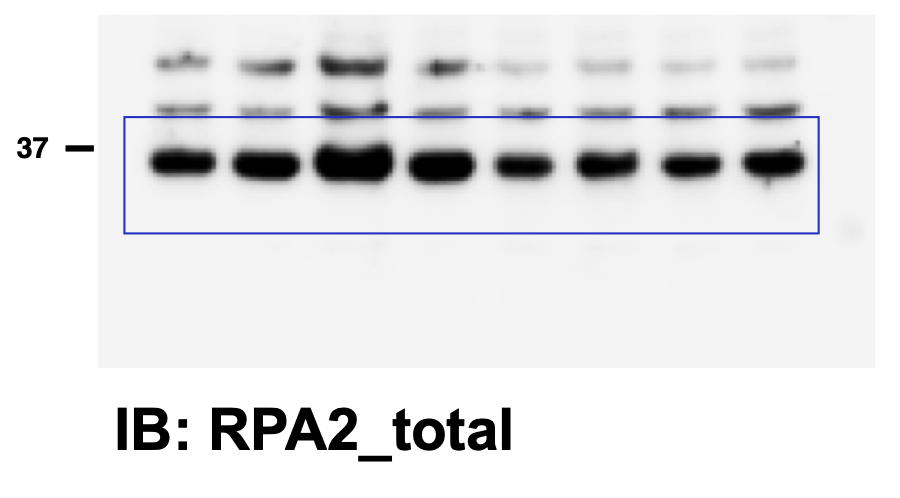

Supplement: Supplementary file 3 — Source data Fig. 2 [file 44319_2024_354_MOESM3_ESM.zip › Figure 2/2G/RPA2_total lysate.tif]

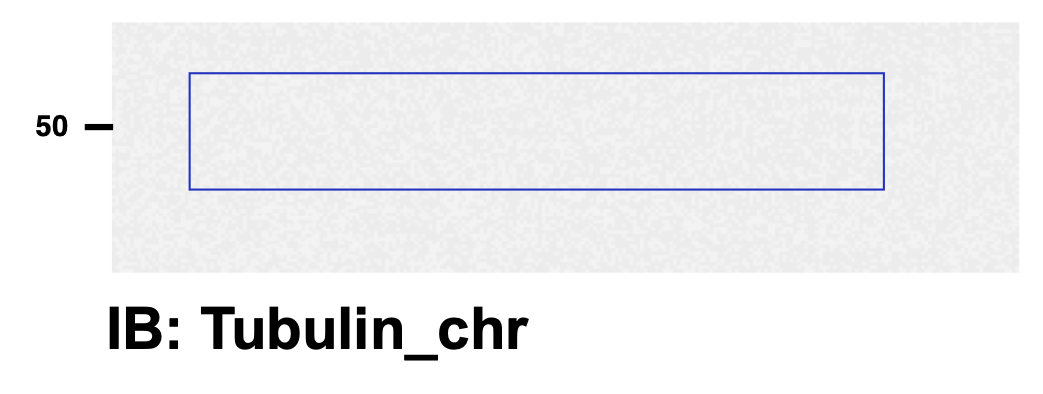

Supplement: Supplementary file 3 — Source data Fig. 2 [file 44319_2024_354_MOESM3_ESM.zip › Figure 2/2G/Tubulin_chromatin.tif]

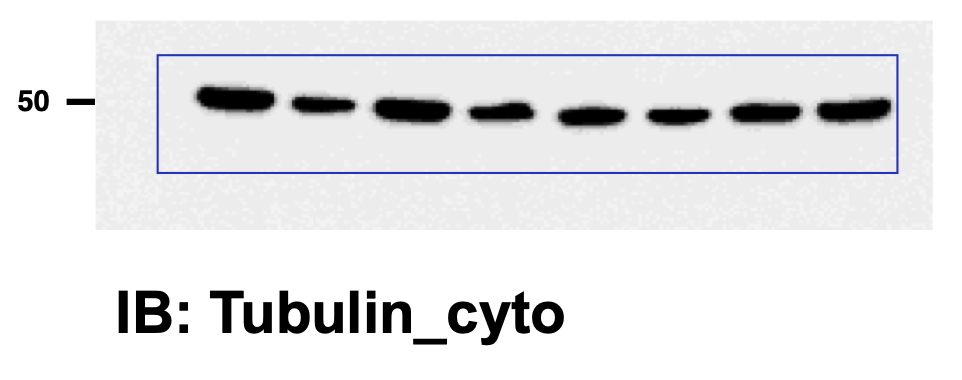

Supplement: Supplementary file 3 — Source data Fig. 2 [file 44319_2024_354_MOESM3_ESM.zip › Figure 2/2G/Tubulin_cytoplasm.tif]

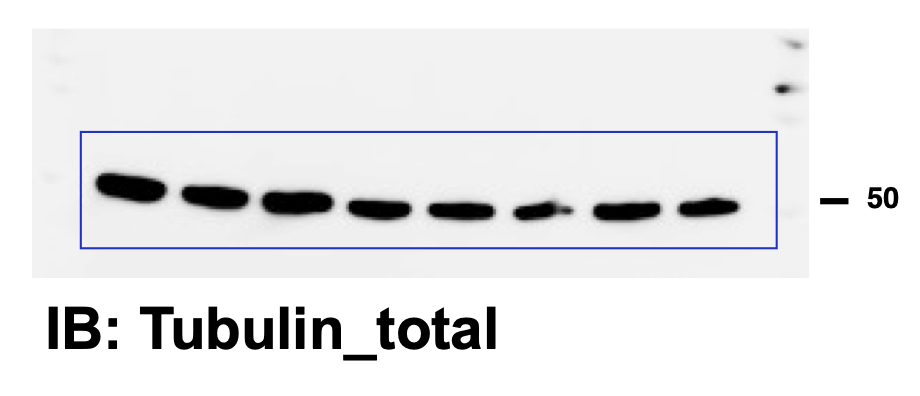

Supplement: Supplementary file 3 — Source data Fig. 2 [file 44319_2024_354_MOESM3_ESM.zip › Figure 2/2G/Tubulin_total lysate.tif]

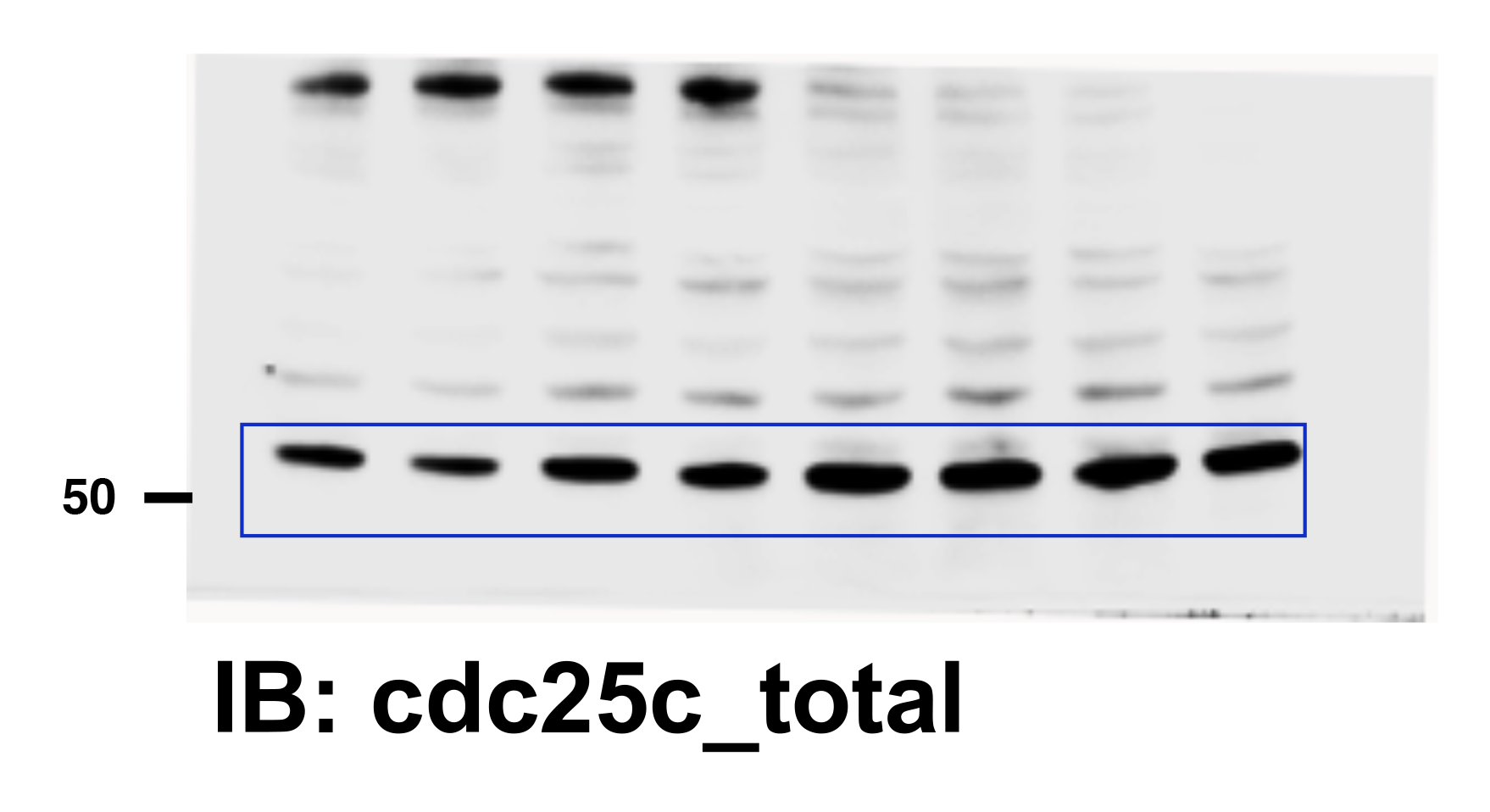

Supplement: Supplementary file 3 — Source data Fig. 2 [file 44319_2024_354_MOESM3_ESM.zip › Figure 2/2H/cdc25_total lysate.tif]

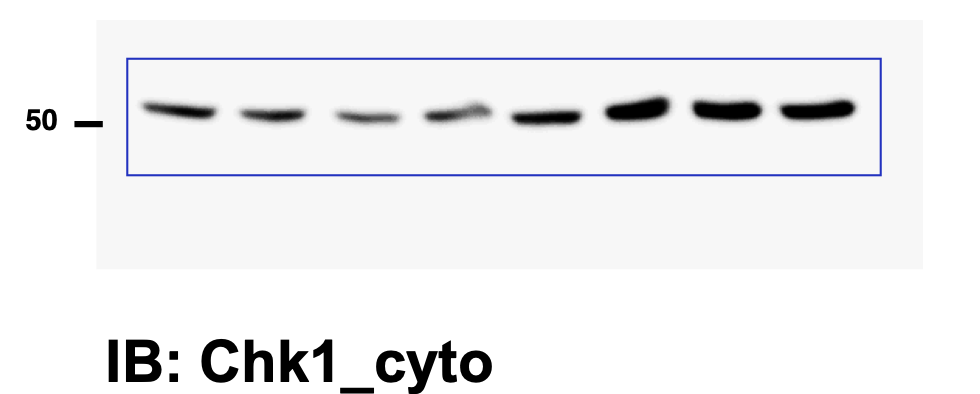

Supplement: Supplementary file 3 — Source data Fig. 2 [file 44319_2024_354_MOESM3_ESM.zip › Figure 2/2H/Chk1_cytoplasm.tif]

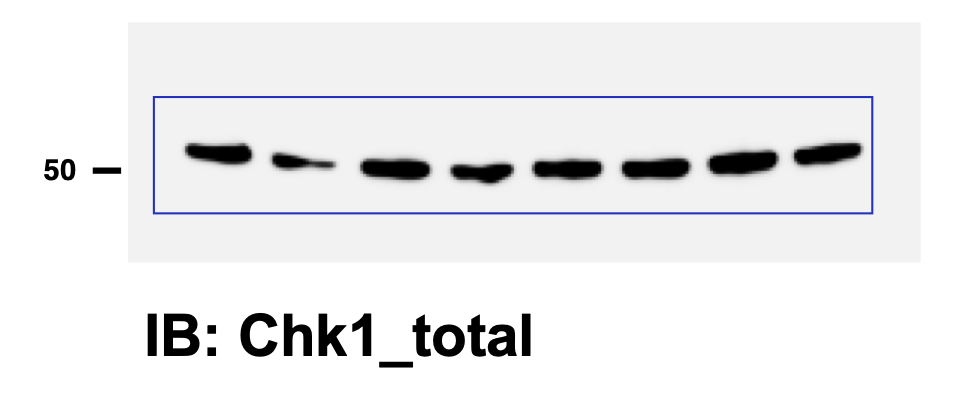

Supplement: Supplementary file 3 — Source data Fig. 2 [file 44319_2024_354_MOESM3_ESM.zip › Figure 2/2H/Chk1_total lysate.tif]

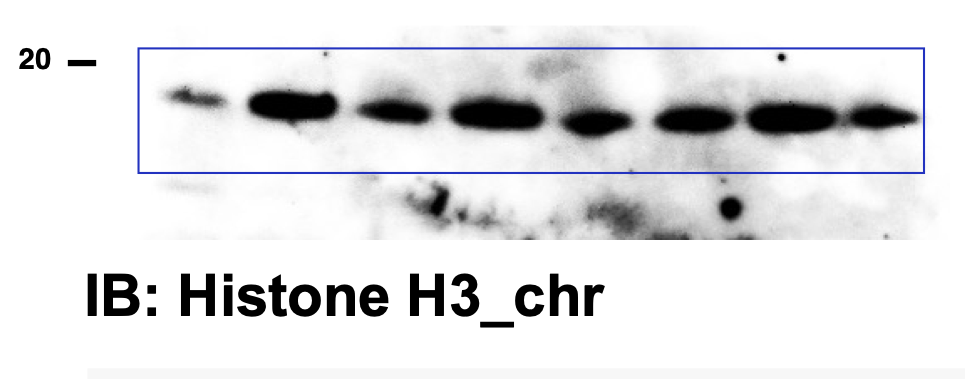

Supplement: Supplementary file 3 — Source data Fig. 2 [file 44319_2024_354_MOESM3_ESM.zip › Figure 2/2H/Histone H3_chromatin.tif]

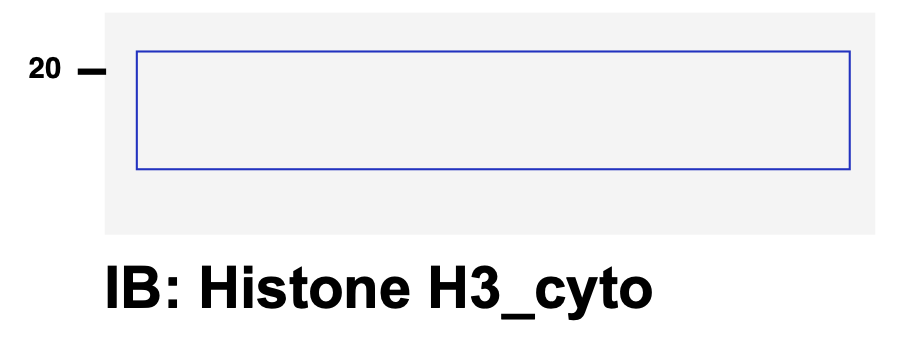

Supplement: Supplementary file 3 — Source data Fig. 2 [file 44319_2024_354_MOESM3_ESM.zip › Figure 2/2H/Histone H3_cytoplasm.tif]

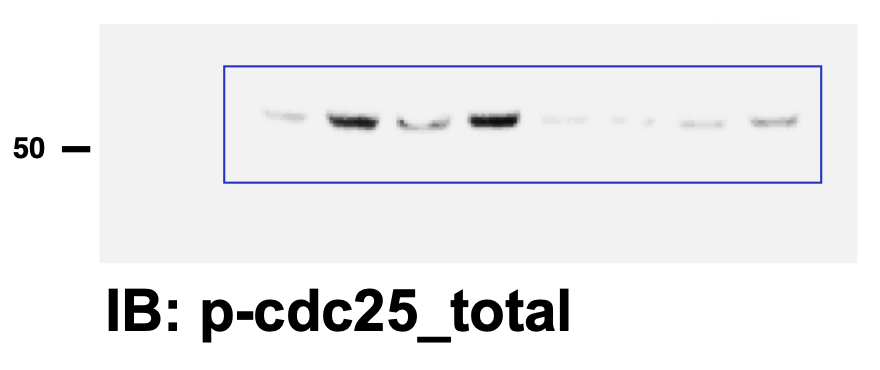

Supplement: Supplementary file 3 — Source data Fig. 2 [file 44319_2024_354_MOESM3_ESM.zip › Figure 2/2H/p-cdc25_total lysate.tif]

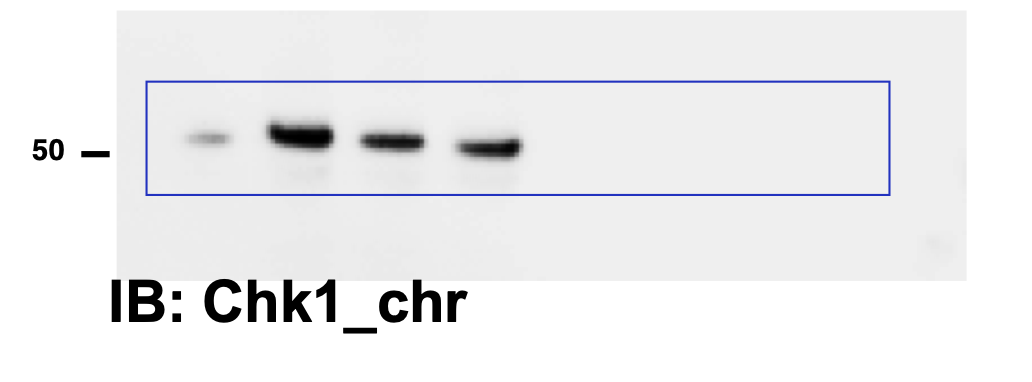

Supplement: Supplementary file 3 — Source data Fig. 2 [file 44319_2024_354_MOESM3_ESM.zip › Figure 2/2H/pChk1_chromatin.tif]

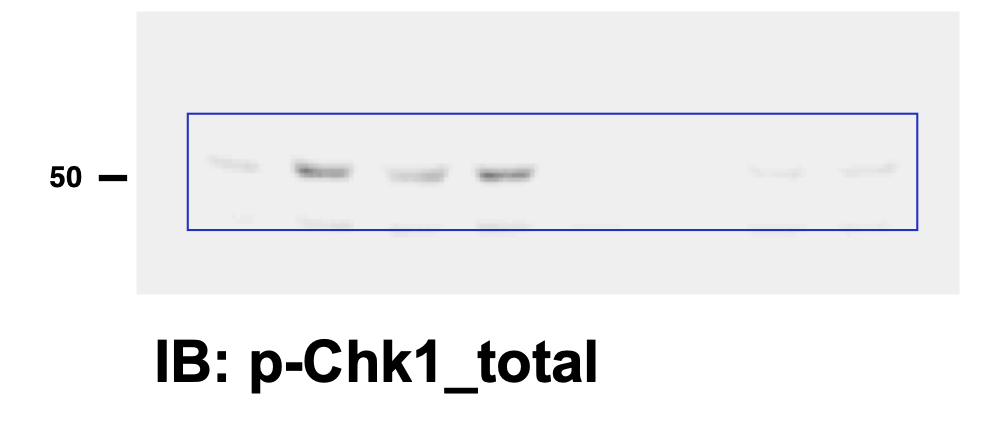

Supplement: Supplementary file 3 — Source data Fig. 2 [file 44319_2024_354_MOESM3_ESM.zip › Figure 2/2H/pChk1_total lysate.tif]

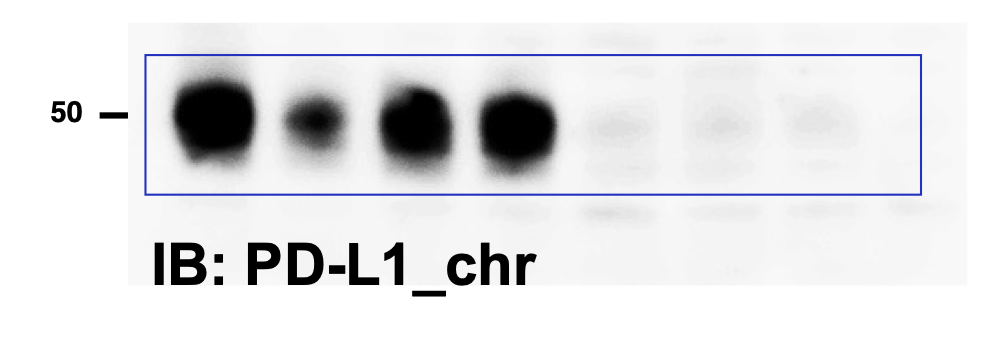

Supplement: Supplementary file 3 — Source data Fig. 2 [file 44319_2024_354_MOESM3_ESM.zip › Figure 2/2H/PD-L1_chromatin.tif]

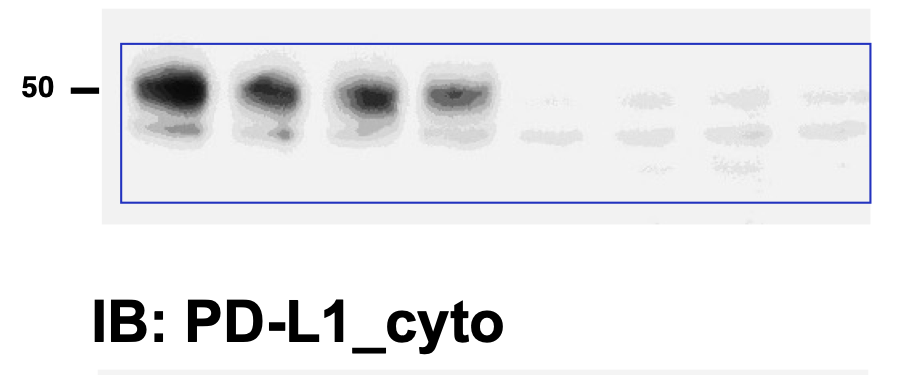

Supplement: Supplementary file 3 — Source data Fig. 2 [file 44319_2024_354_MOESM3_ESM.zip › Figure 2/2H/PD-L1_cytoplasm.tif]

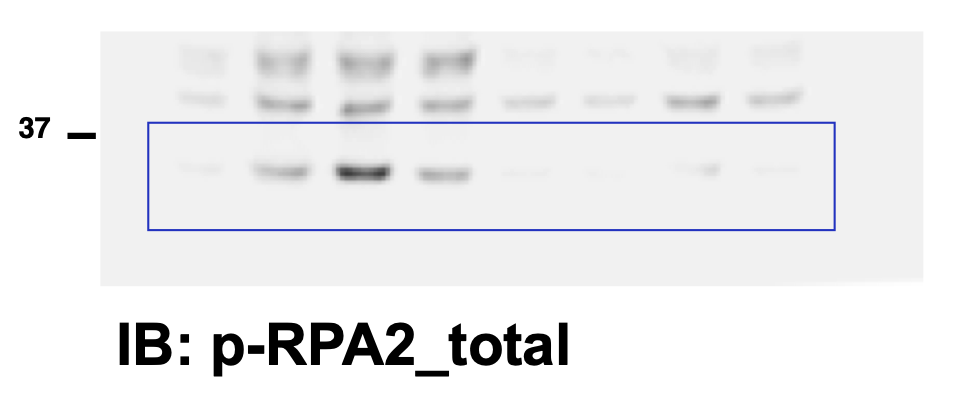

Supplement: Supplementary file 3 — Source data Fig. 2 [file 44319_2024_354_MOESM3_ESM.zip › Figure 2/2H/pRPA2_total lysate.tif]

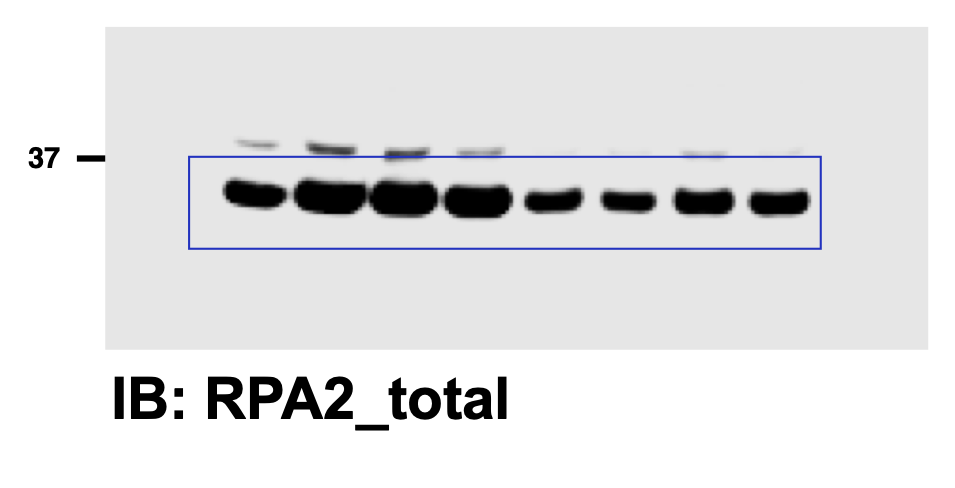

Supplement: Supplementary file 3 — Source data Fig. 2 [file 44319_2024_354_MOESM3_ESM.zip › Figure 2/2H/RPA2_total lysate.tif]

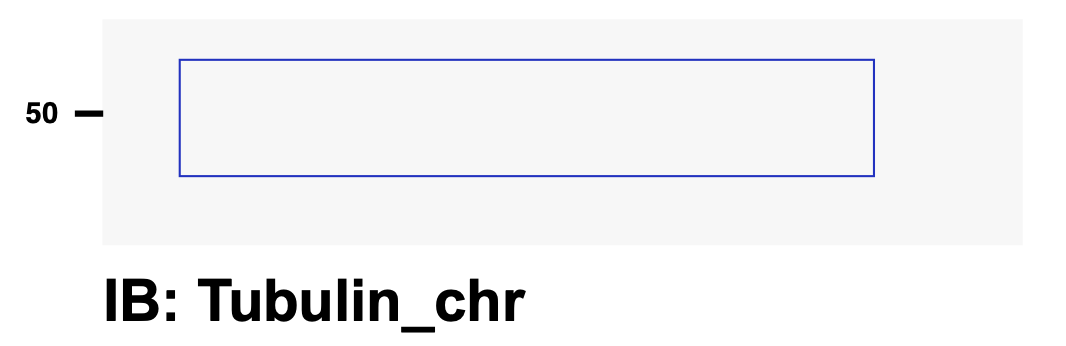

Supplement: Supplementary file 3 — Source data Fig. 2 [file 44319_2024_354_MOESM3_ESM.zip › Figure 2/2H/Tubulin_chromatin.tif]

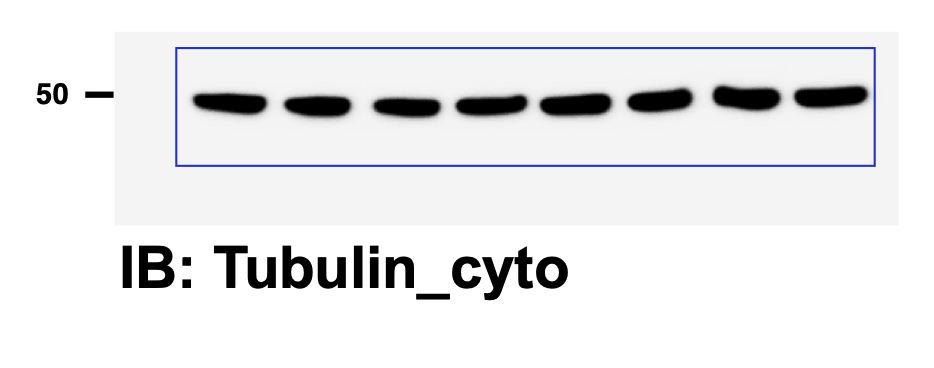

Supplement: Supplementary file 3 — Source data Fig. 2 [file 44319_2024_354_MOESM3_ESM.zip › Figure 2/2H/Tubulin_cytoplasm.tif]

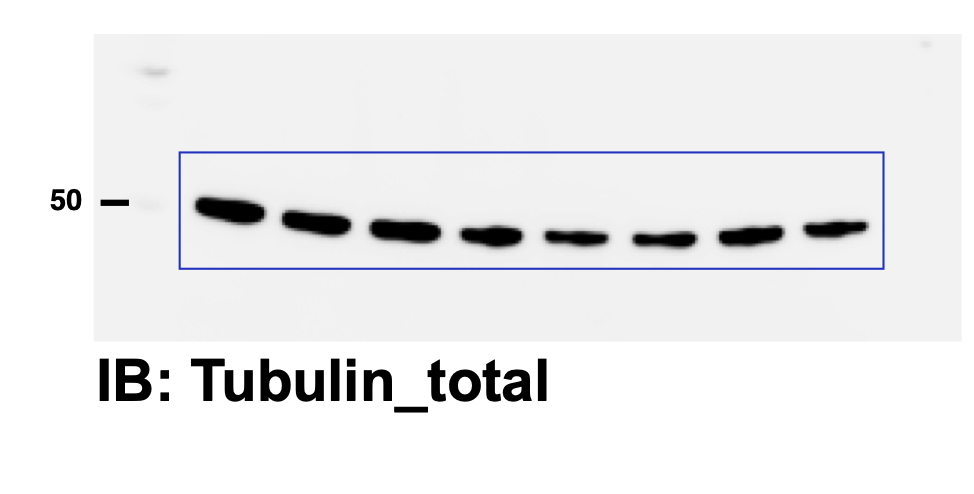

Supplement: Supplementary file 3 — Source data Fig. 2 [file 44319_2024_354_MOESM3_ESM.zip › Figure 2/2H/Tubulin_total lysate.tif]

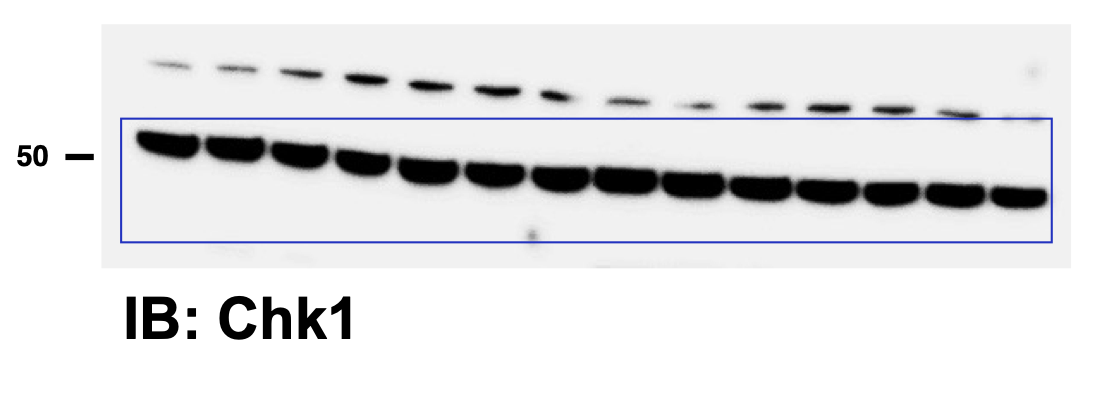

Supplement: Supplementary file 4 — Source data Fig. 3 [file 44319_2024_354_MOESM4_ESM.zip › Figure 3/3A/Chk1.tif]

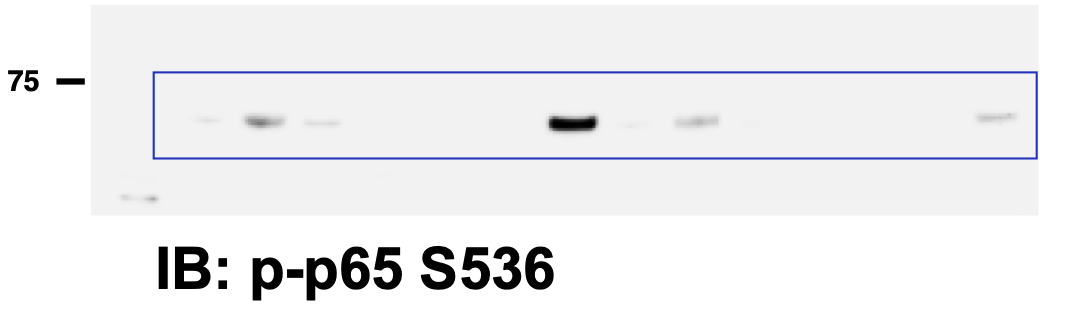

Supplement: Supplementary file 4 — Source data Fig. 3 [file 44319_2024_354_MOESM4_ESM.zip › Figure 3/3A/p-p65.tif]

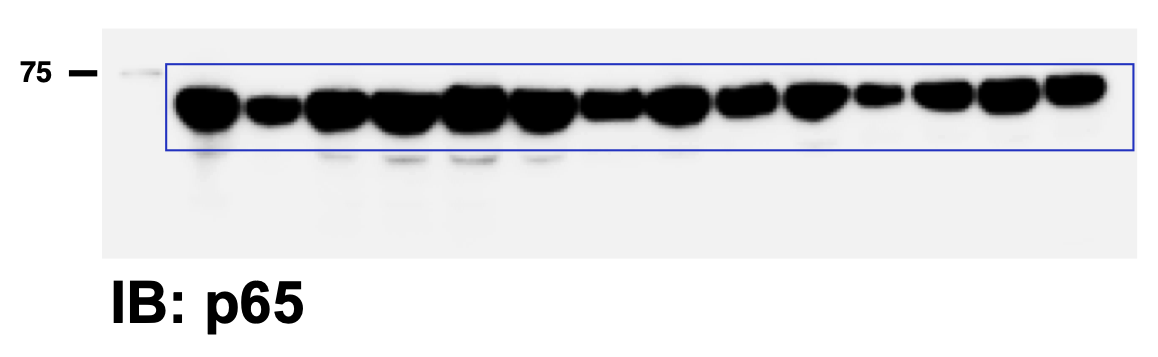

Supplement: Supplementary file 4 — Source data Fig. 3 [file 44319_2024_354_MOESM4_ESM.zip › Figure 3/3A/p65.tif]

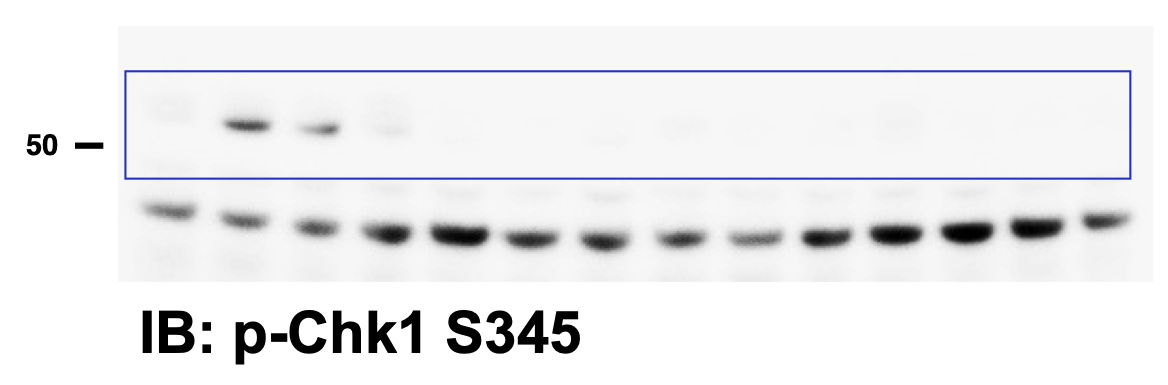

Supplement: Supplementary file 4 — Source data Fig. 3 [file 44319_2024_354_MOESM4_ESM.zip › Figure 3/3A/pChk1.tif]

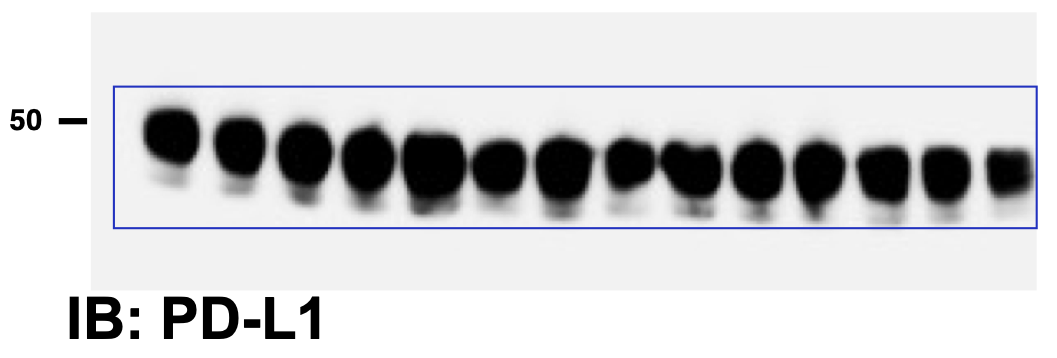

Supplement: Supplementary file 4 — Source data Fig. 3 [file 44319_2024_354_MOESM4_ESM.zip › Figure 3/3A/PD-L1.tif]

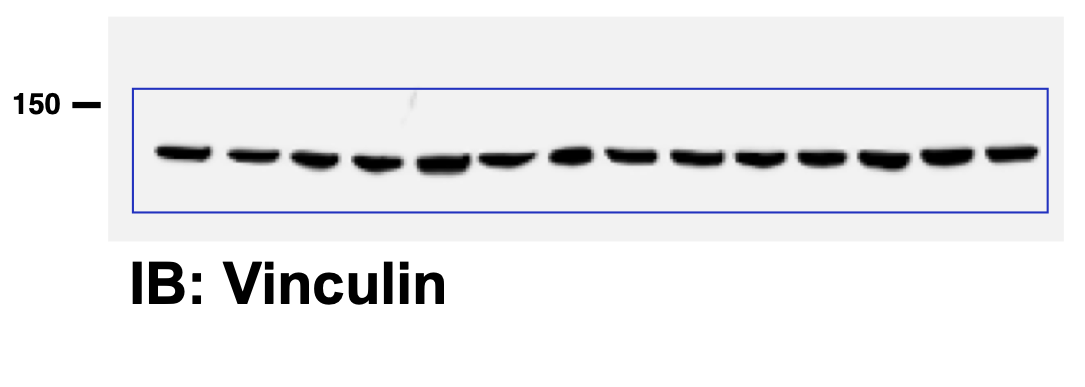

Supplement: Supplementary file 4 — Source data Fig. 3 [file 44319_2024_354_MOESM4_ESM.zip › Figure 3/3A/Vinculin.tif]

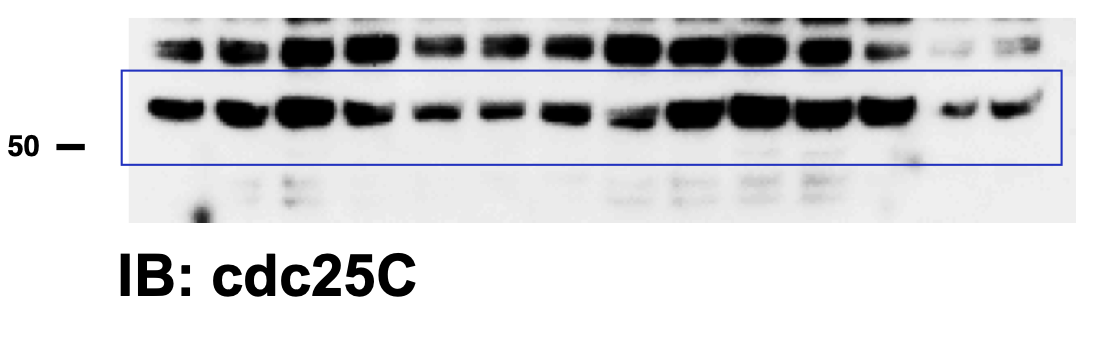

Supplement: Supplementary file 4 — Source data Fig. 3 [file 44319_2024_354_MOESM4_ESM.zip › Figure 3/3B/cdc25.tif]

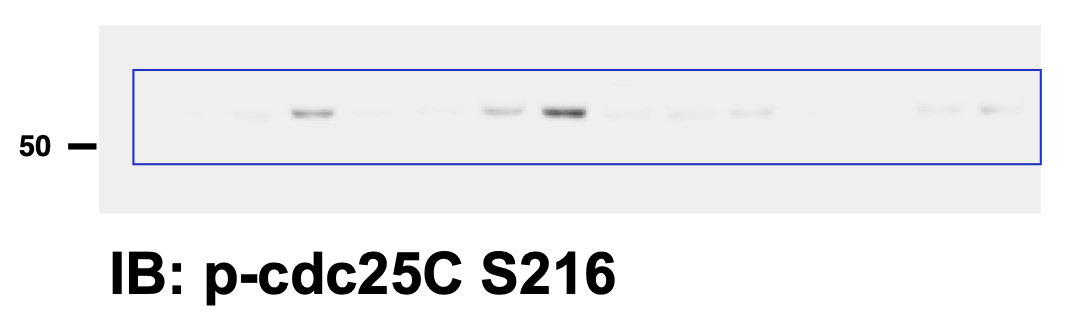

Supplement: Supplementary file 4 — Source data Fig. 3 [file 44319_2024_354_MOESM4_ESM.zip › Figure 3/3B/p-dc25.tif]

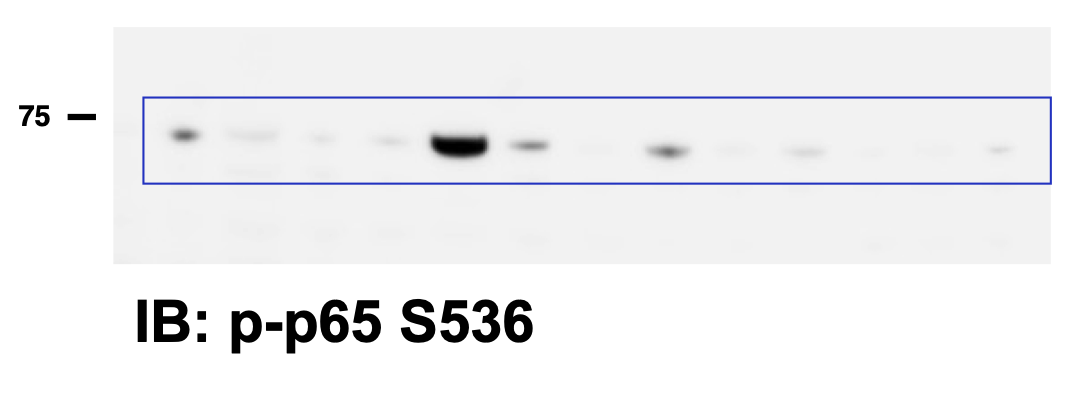

Supplement: Supplementary file 4 — Source data Fig. 3 [file 44319_2024_354_MOESM4_ESM.zip › Figure 3/3B/p-p65.tif]

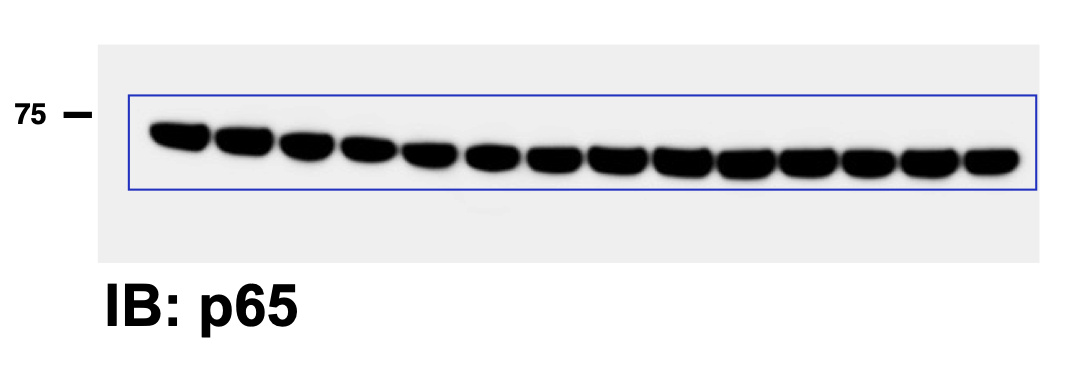

Supplement: Supplementary file 4 — Source data Fig. 3 [file 44319_2024_354_MOESM4_ESM.zip › Figure 3/3B/p65.tif]

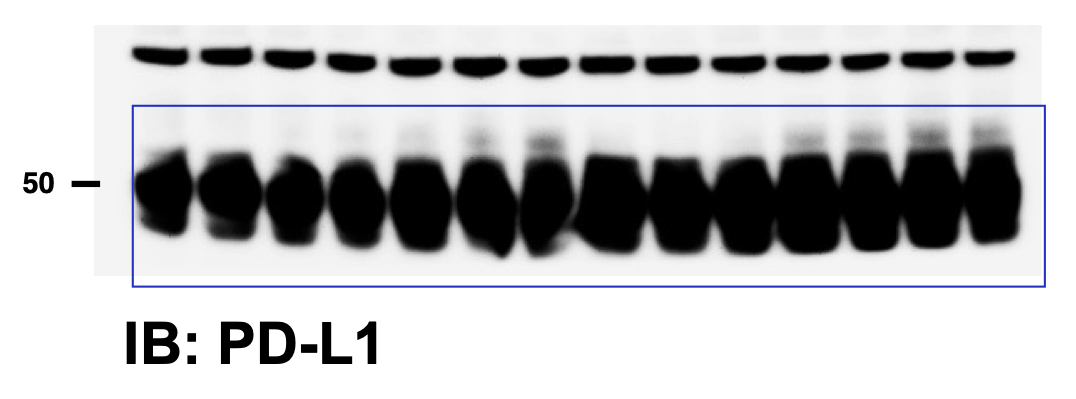

Supplement: Supplementary file 4 — Source data Fig. 3 [file 44319_2024_354_MOESM4_ESM.zip › Figure 3/3B/PD-L1.tif]

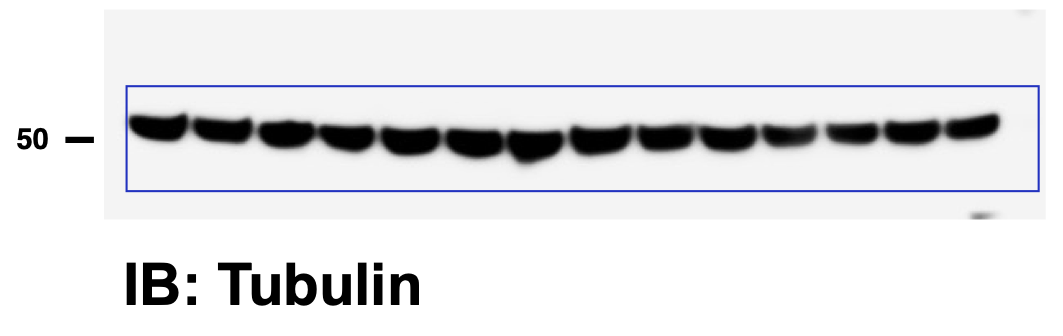

Supplement: Supplementary file 4 — Source data Fig. 3 [file 44319_2024_354_MOESM4_ESM.zip › Figure 3/3B/Tubulin.tif]

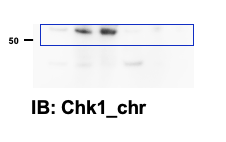

Supplement: Supplementary file 4 — Source data Fig. 3 [file 44319_2024_354_MOESM4_ESM.zip › Figure 3/3C/Chk1_chromatin.tif]

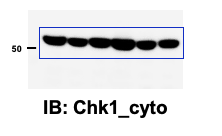

Supplement: Supplementary file 4 — Source data Fig. 3 [file 44319_2024_354_MOESM4_ESM.zip › Figure 3/3C/Chk1_cytplasm.tif]

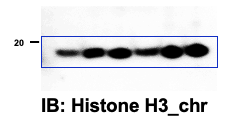

Supplement: Supplementary file 4 — Source data Fig. 3 [file 44319_2024_354_MOESM4_ESM.zip › Figure 3/3C/Histone H3_chromatin.tif]

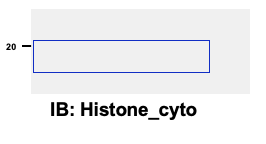

Supplement: Supplementary file 4 — Source data Fig. 3 [file 44319_2024_354_MOESM4_ESM.zip › Figure 3/3C/Histone H3_cytoplasm.tif]

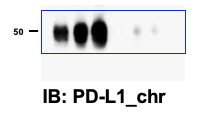

Supplement: Supplementary file 4 — Source data Fig. 3 [file 44319_2024_354_MOESM4_ESM.zip › Figure 3/3C/PD-L1_chromatin.tif]

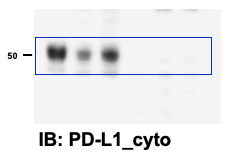

Supplement: Supplementary file 4 — Source data Fig. 3 [file 44319_2024_354_MOESM4_ESM.zip › Figure 3/3C/PD-L1_cytoplasm.tif]

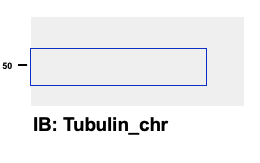

Supplement: Supplementary file 4 — Source data Fig. 3 [file 44319_2024_354_MOESM4_ESM.zip › Figure 3/3C/Tubulin_chromatin.tif]

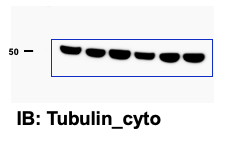

Supplement: Supplementary file 4 — Source data Fig. 3 [file 44319_2024_354_MOESM4_ESM.zip › Figure 3/3C/Tubulin_cytoplasm.tif]

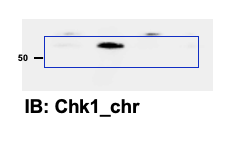

Supplement: Supplementary file 4 — Source data Fig. 3 [file 44319_2024_354_MOESM4_ESM.zip › Figure 3/3D/Chk1_chromatin.tif]

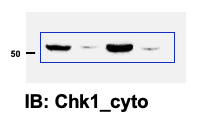

Supplement: Supplementary file 4 — Source data Fig. 3 [file 44319_2024_354_MOESM4_ESM.zip › Figure 3/3D/Chk1_cytoplasm.tif]

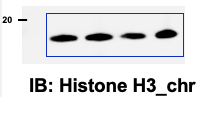

Supplement: Supplementary file 4 — Source data Fig. 3 [file 44319_2024_354_MOESM4_ESM.zip › Figure 3/3D/Histone H3_chromatin.tif]

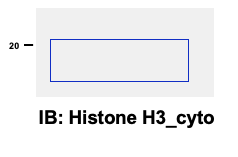

Supplement: Supplementary file 4 — Source data Fig. 3 [file 44319_2024_354_MOESM4_ESM.zip › Figure 3/3D/Histone H3_cytoplasm.tif]

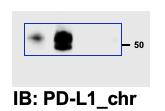

Supplement: Supplementary file 4 — Source data Fig. 3 [file 44319_2024_354_MOESM4_ESM.zip › Figure 3/3D/PD-L1_chromatin.tif]

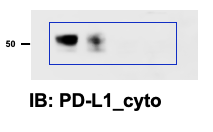

Supplement: Supplementary file 4 — Source data Fig. 3 [file 44319_2024_354_MOESM4_ESM.zip › Figure 3/3D/PD-L1_cytoplasm.tif]

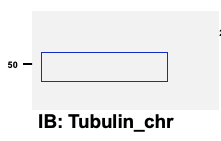

Supplement: Supplementary file 4 — Source data Fig. 3 [file 44319_2024_354_MOESM4_ESM.zip › Figure 3/3D/Tubulin_chromatin.tif]

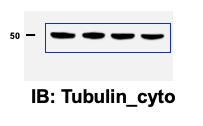

Supplement: Supplementary file 4 — Source data Fig. 3 [file 44319_2024_354_MOESM4_ESM.zip › Figure 3/3D/Tubulin_cytoplasm.tif]

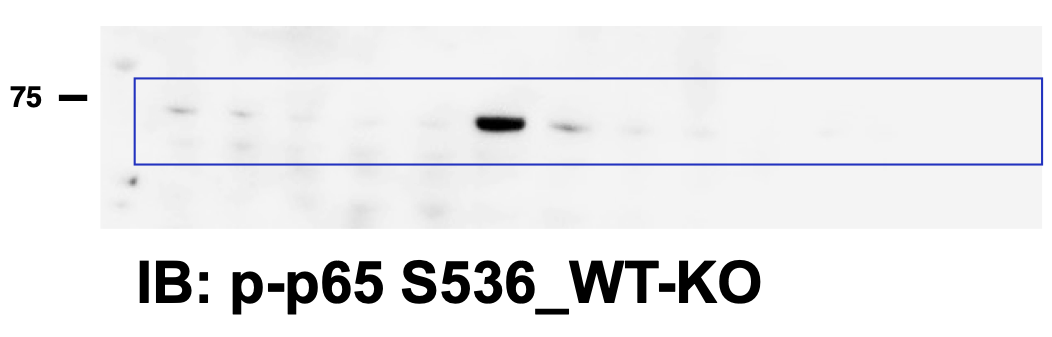

Supplement: Supplementary file 4 — Source data Fig. 3 [file 44319_2024_354_MOESM4_ESM.zip › Figure 3/3E/p-p65.tif]

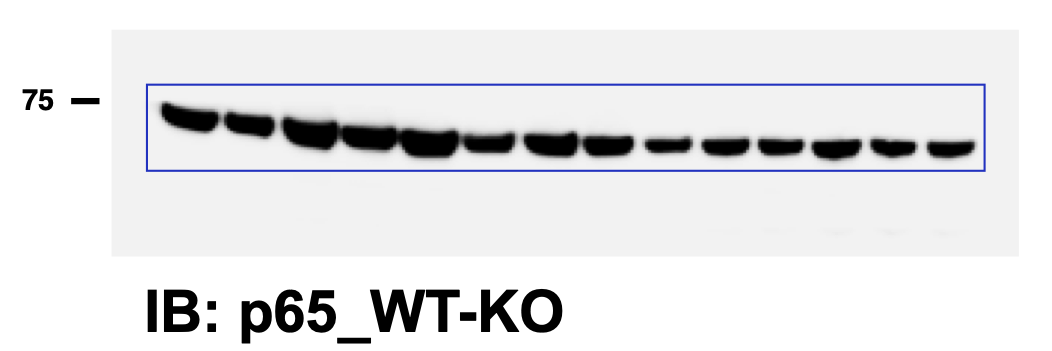

Supplement: Supplementary file 4 — Source data Fig. 3 [file 44319_2024_354_MOESM4_ESM.zip › Figure 3/3E/p65.tif]

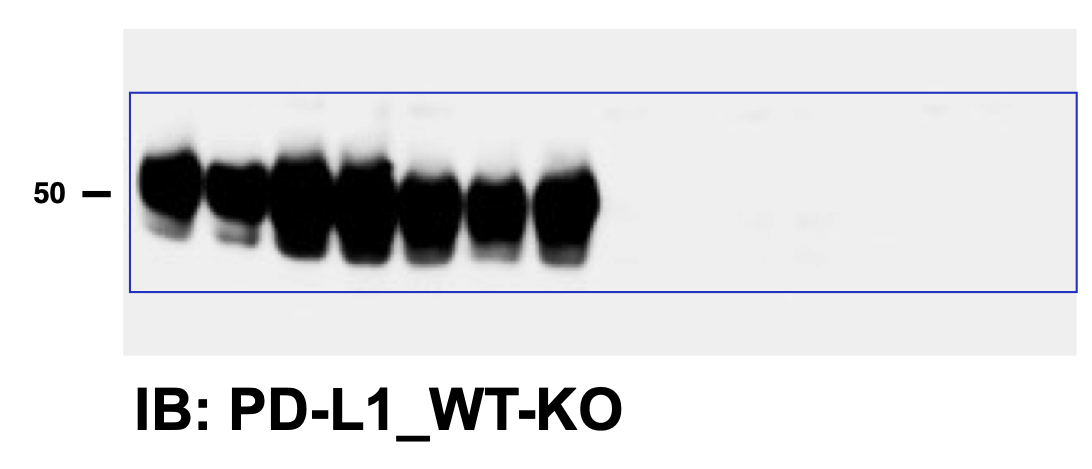

Supplement: Supplementary file 4 — Source data Fig. 3 [file 44319_2024_354_MOESM4_ESM.zip › Figure 3/3E/PD-L1.tif]

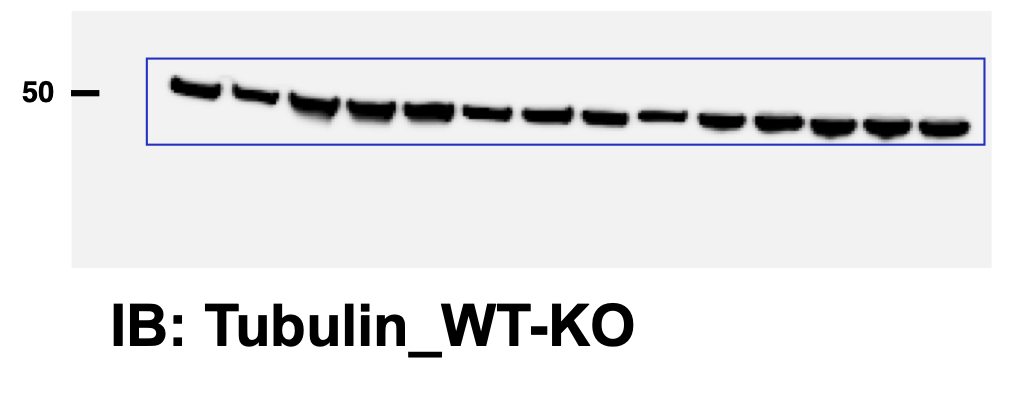

Supplement: Supplementary file 4 — Source data Fig. 3 [file 44319_2024_354_MOESM4_ESM.zip › Figure 3/3E/Tubulin.tif]

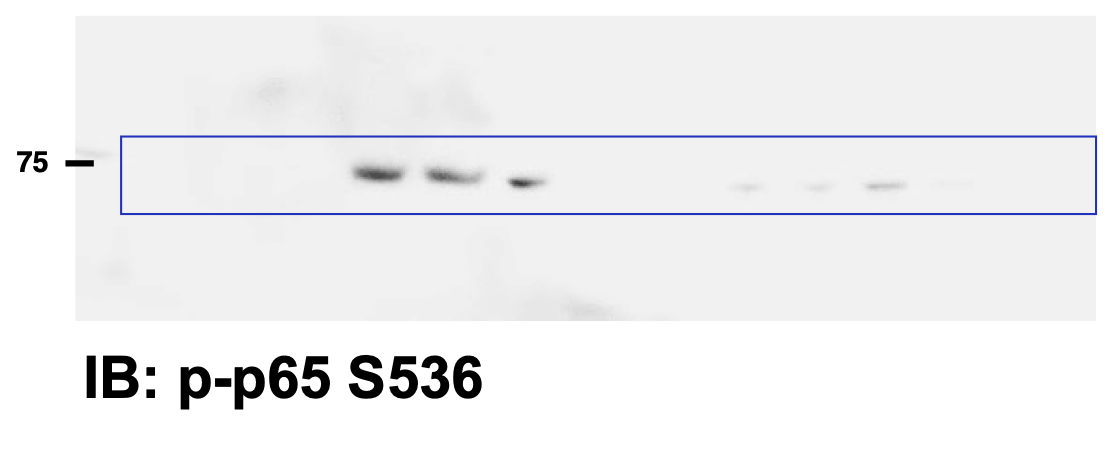

Supplement: Supplementary file 4 — Source data Fig. 3 [file 44319_2024_354_MOESM4_ESM.zip › Figure 3/3F/p-p65.tif]

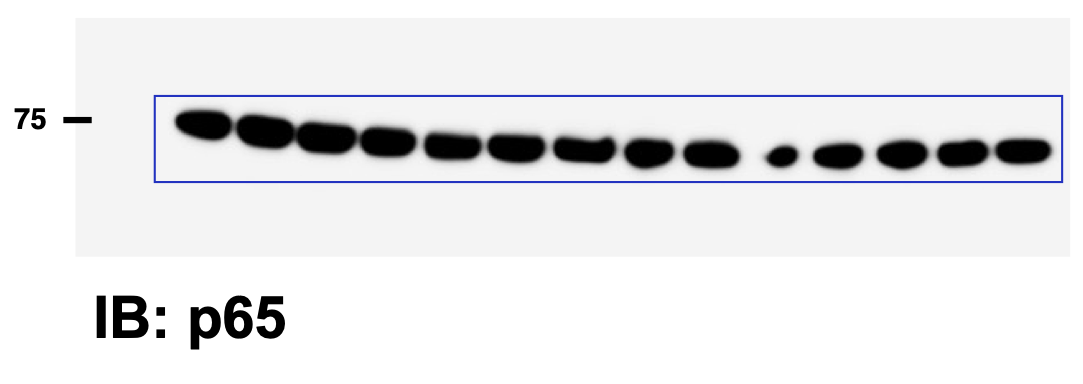

Supplement: Supplementary file 4 — Source data Fig. 3 [file 44319_2024_354_MOESM4_ESM.zip › Figure 3/3F/p65.tif]

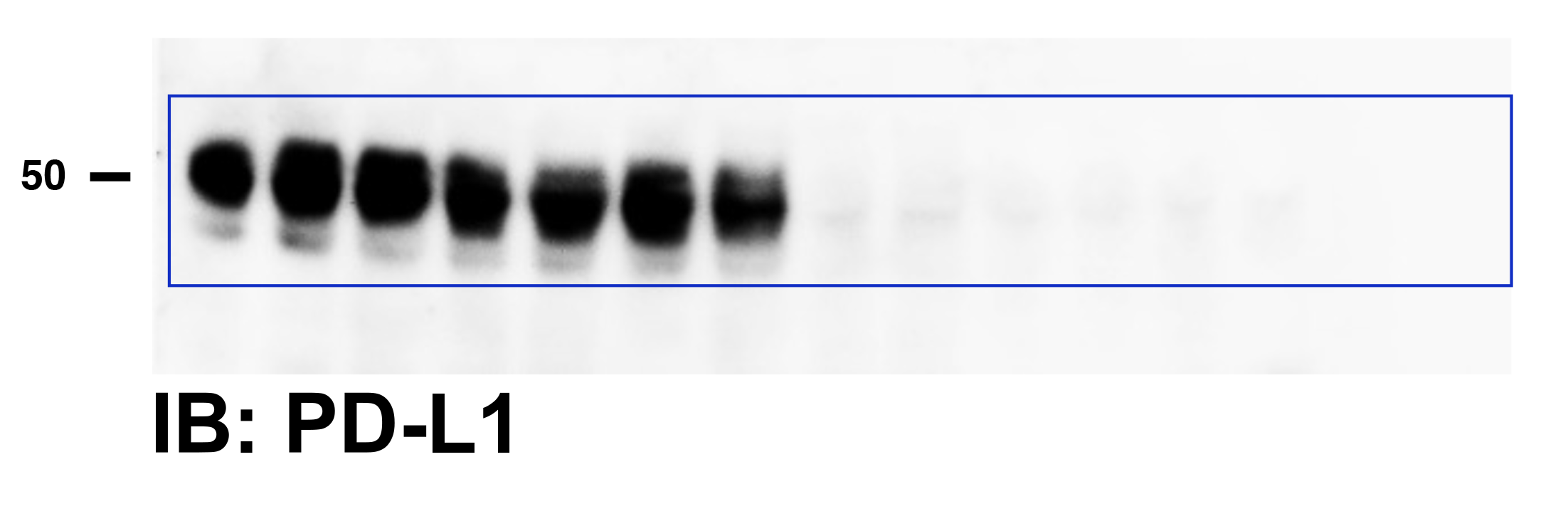

Supplement: Supplementary file 4 — Source data Fig. 3 [file 44319_2024_354_MOESM4_ESM.zip › Figure 3/3F/PD-L1.tif]

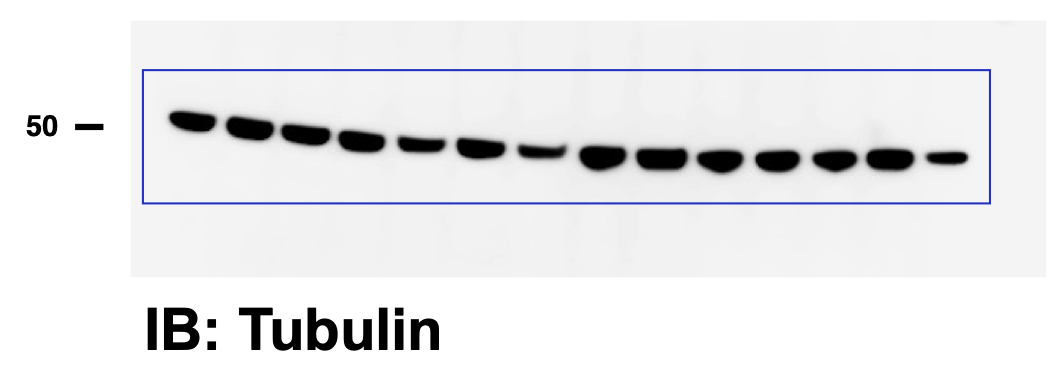

Supplement: Supplementary file 4 — Source data Fig. 3 [file 44319_2024_354_MOESM4_ESM.zip › Figure 3/3F/Tubulin.tif]

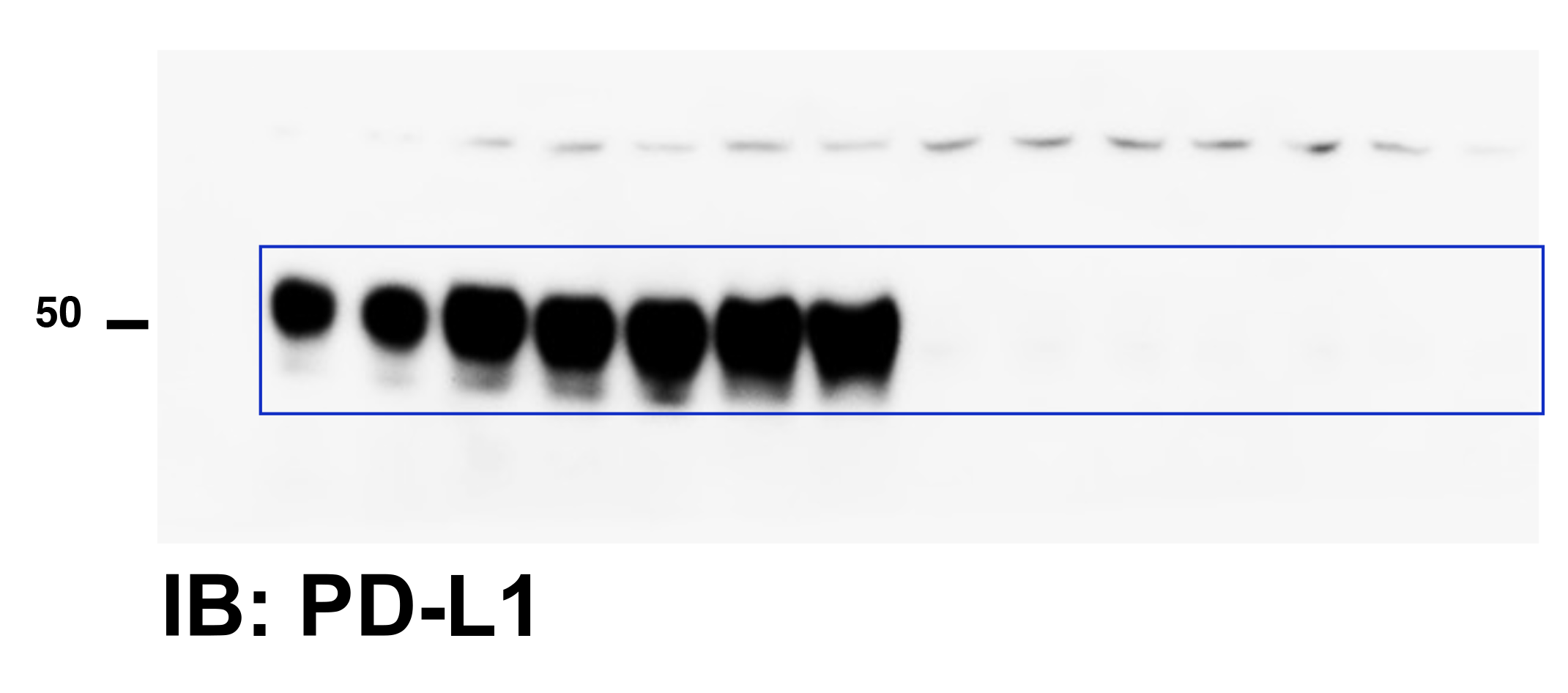

Supplement: Supplementary file 5 — Source data Fig. 4 [file 44319_2024_354_MOESM5_ESM.zip › Figure 4/4A/PD-L1.tif]

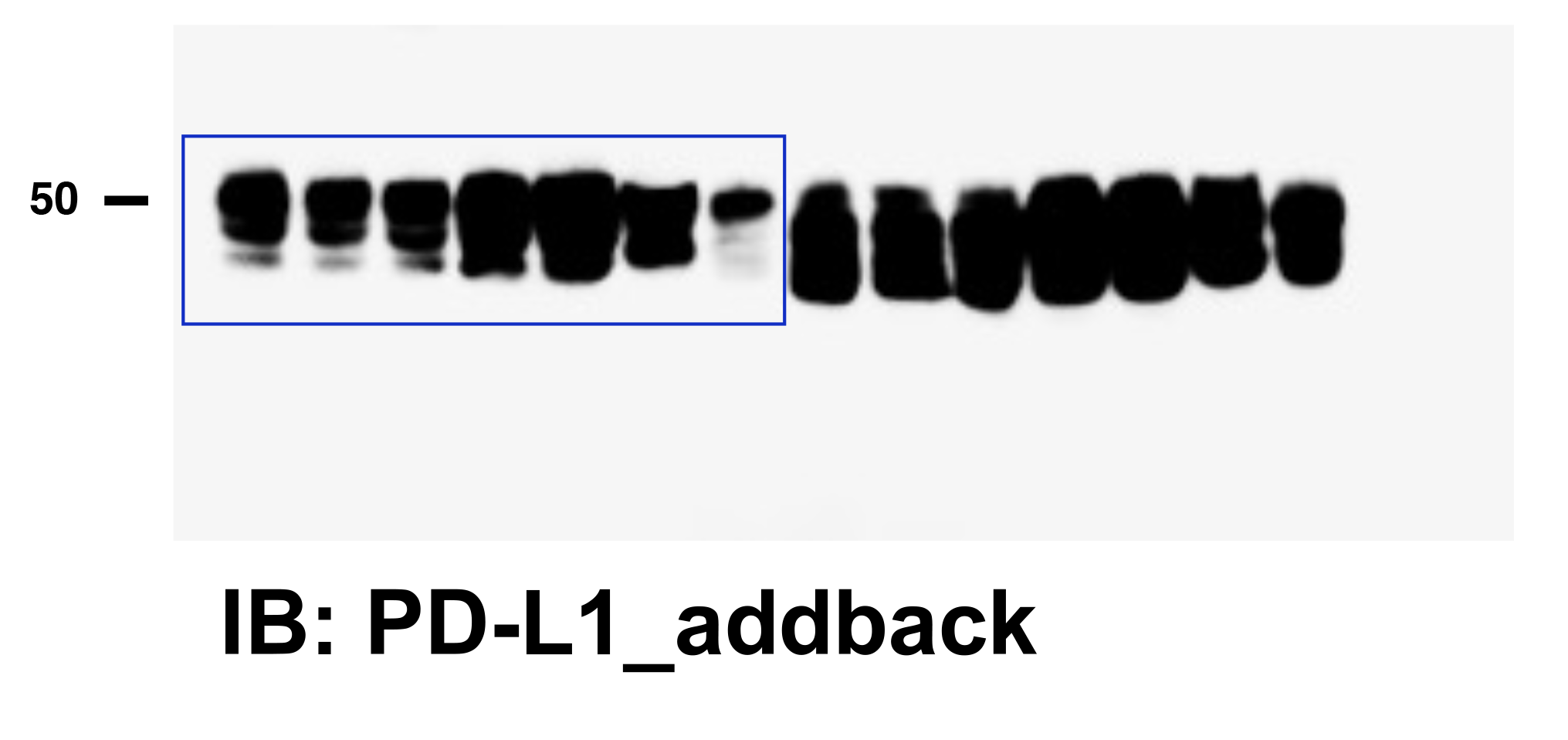

Supplement: Supplementary file 5 — Source data Fig. 4 [file 44319_2024_354_MOESM5_ESM.zip › Figure 4/4A/PD-L1_addback.tif]

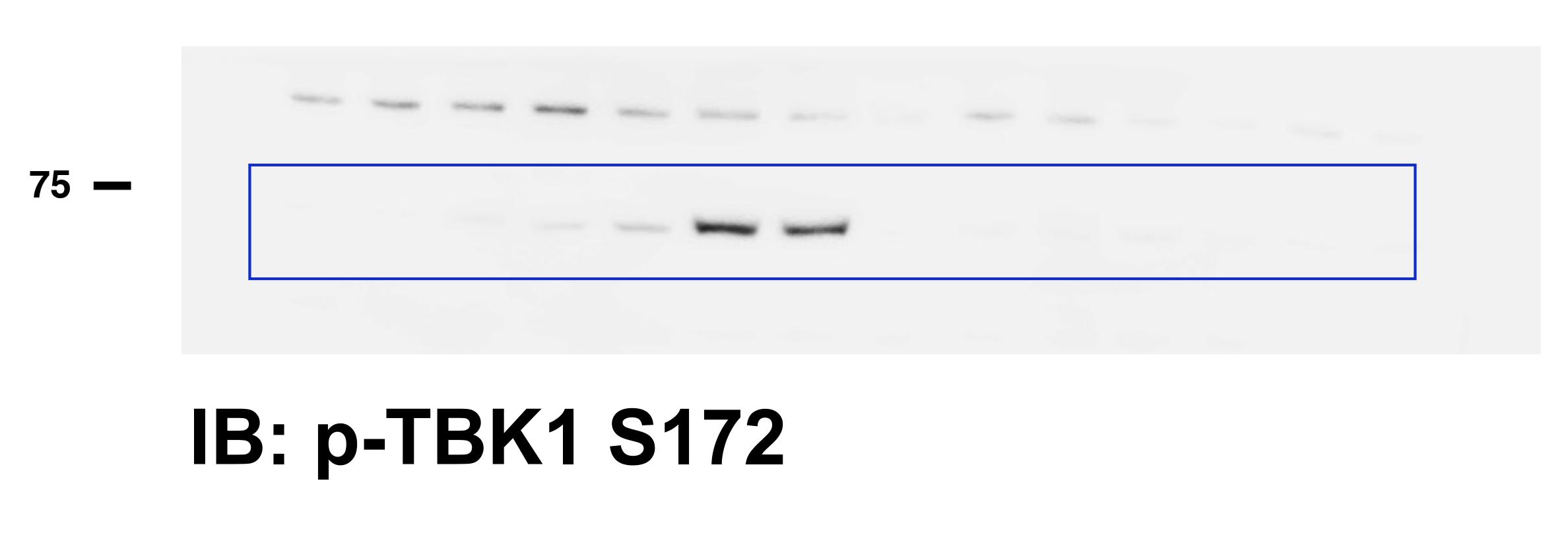

Supplement: Supplementary file 5 — Source data Fig. 4 [file 44319_2024_354_MOESM5_ESM.zip › Figure 4/4A/pTBK1.tif]

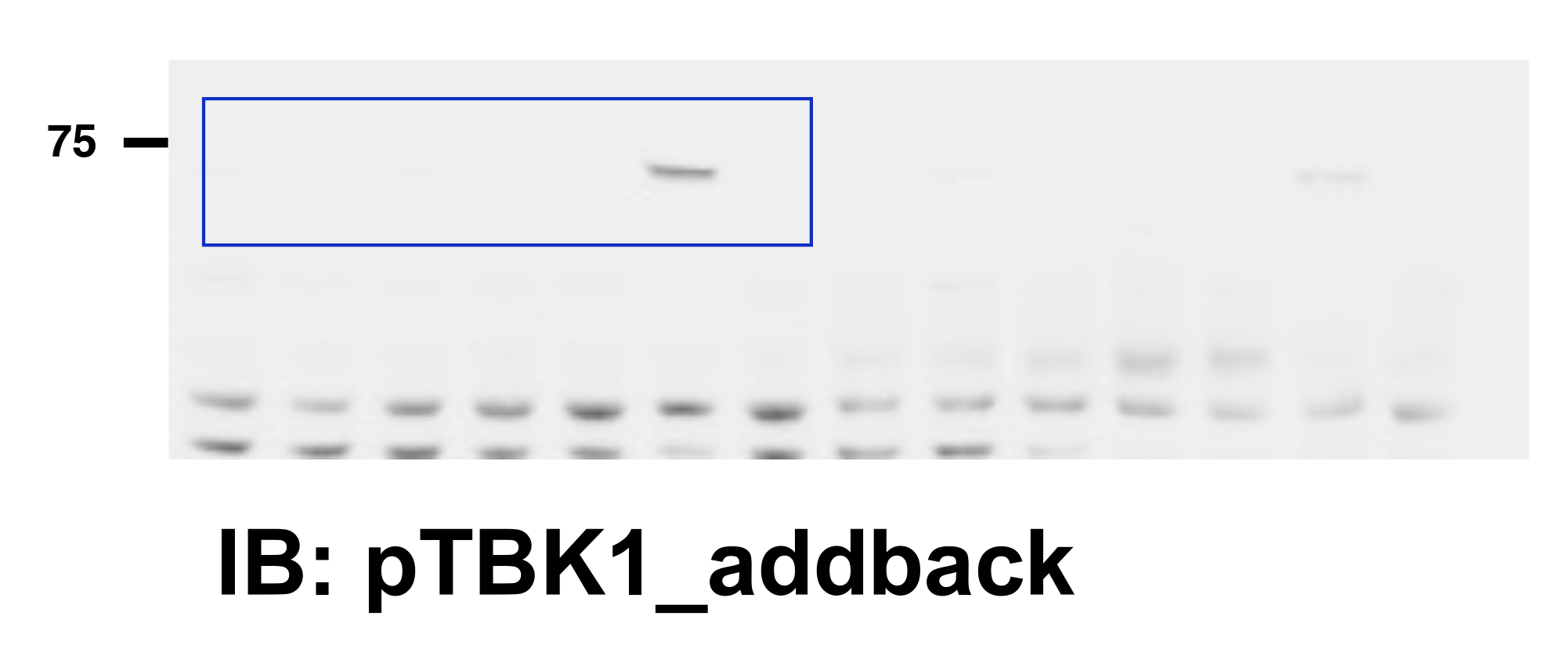

Supplement: Supplementary file 5 — Source data Fig. 4 [file 44319_2024_354_MOESM5_ESM.zip › Figure 4/4A/pTBK1_addback.tif]

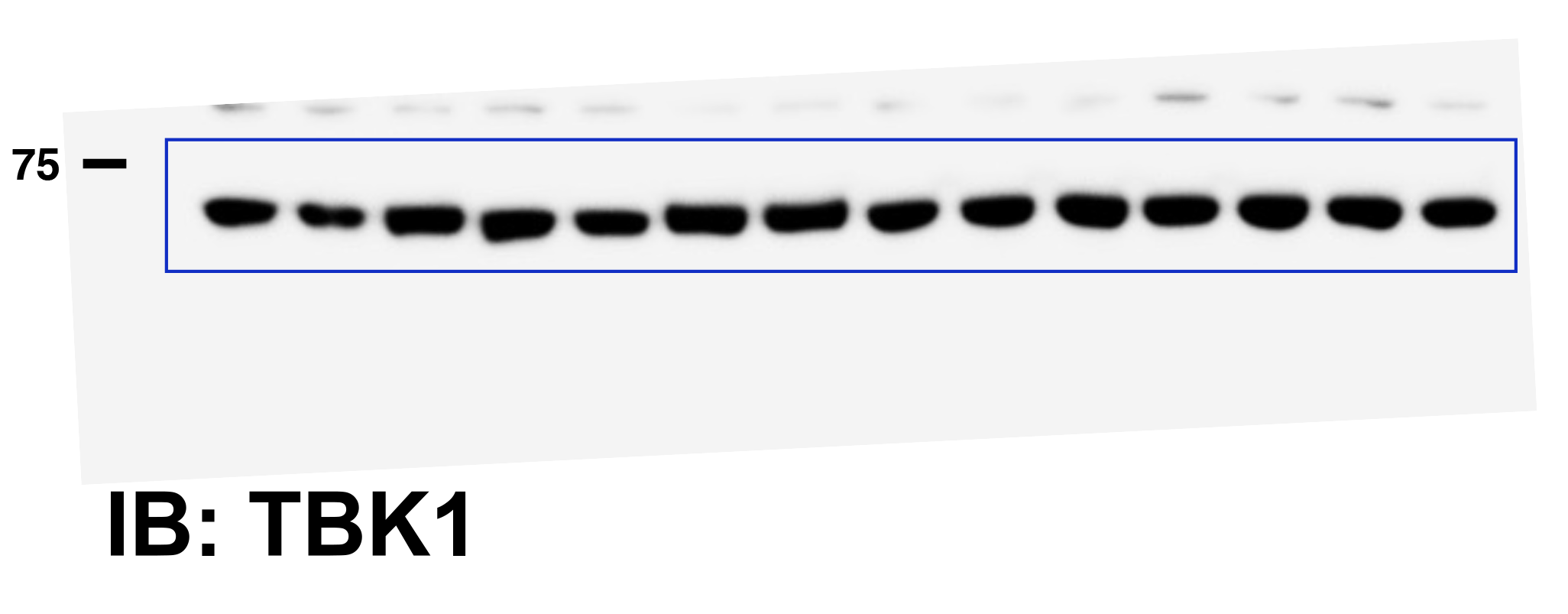

Supplement: Supplementary file 5 — Source data Fig. 4 [file 44319_2024_354_MOESM5_ESM.zip › Figure 4/4A/TBK1.tif]

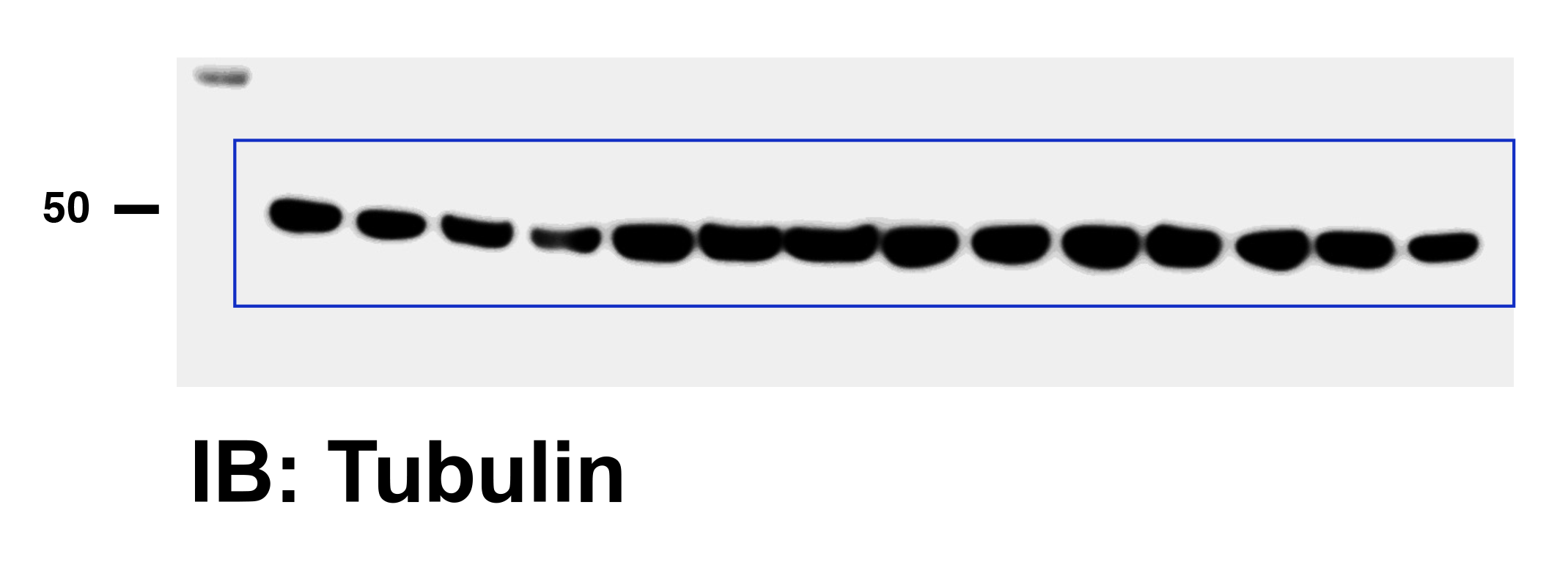

Supplement: Supplementary file 5 — Source data Fig. 4 [file 44319_2024_354_MOESM5_ESM.zip › Figure 4/4A/Tubulin.tif]

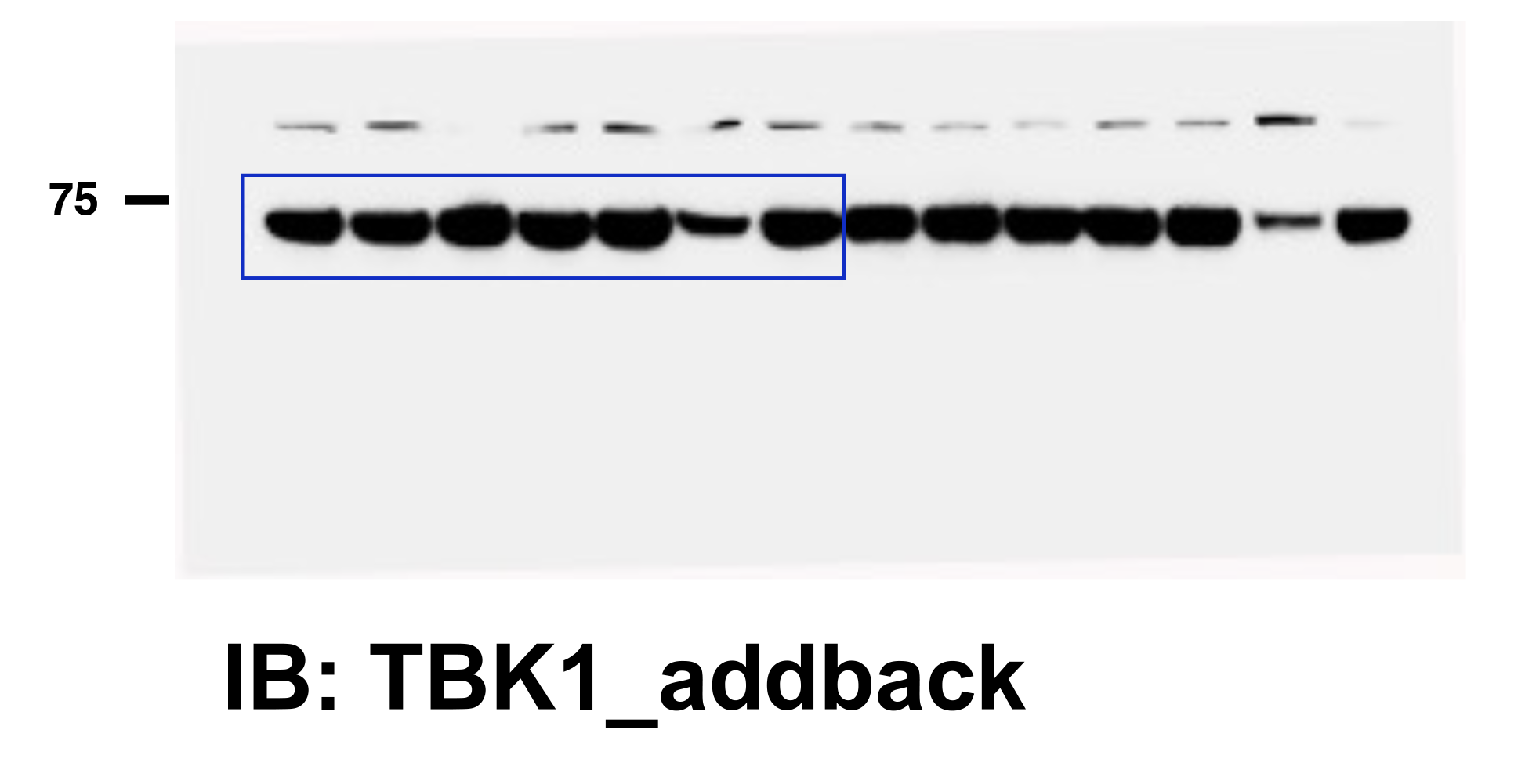

Supplement: Supplementary file 5 — Source data Fig. 4 [file 44319_2024_354_MOESM5_ESM.zip › Figure 4/4A/TBK1_addback.tif]

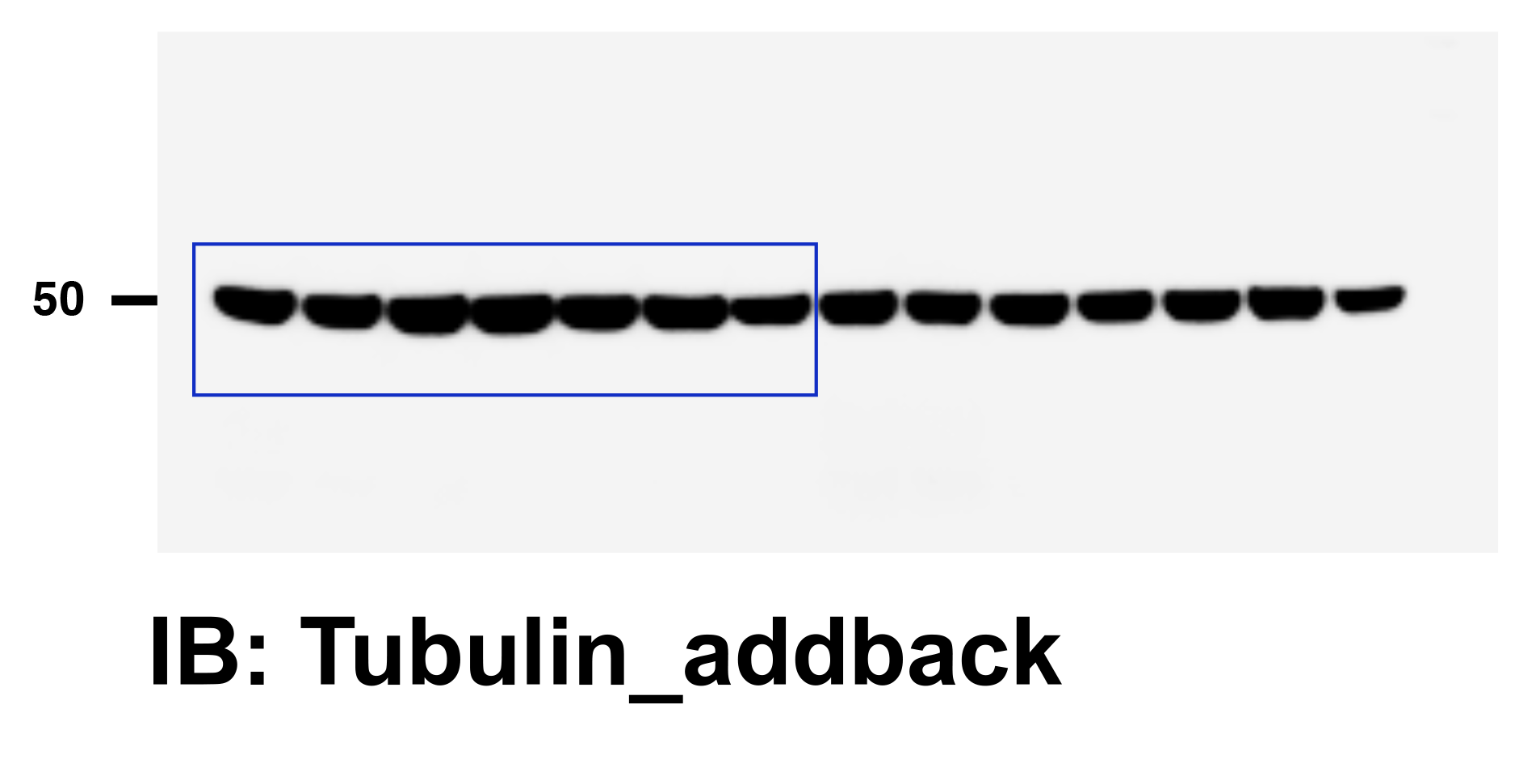

Supplement: Supplementary file 5 — Source data Fig. 4 [file 44319_2024_354_MOESM5_ESM.zip › Figure 4/4A/Tubulin_addback.tif]

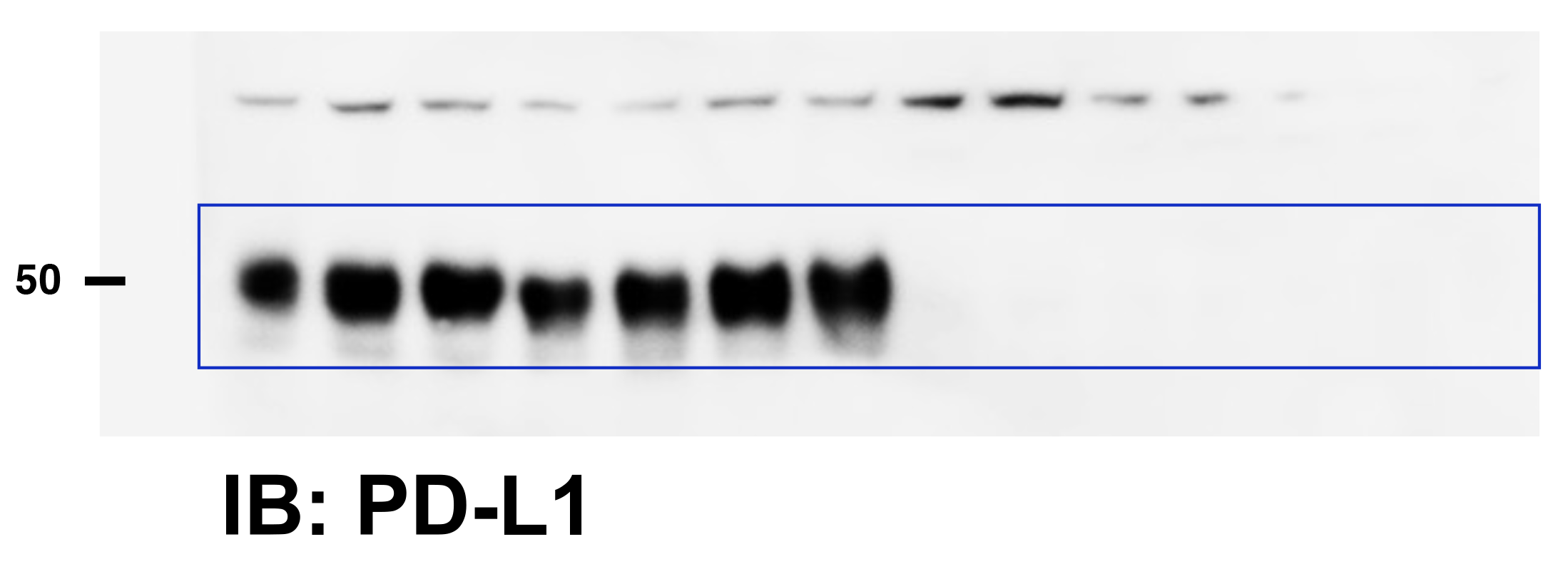

Supplement: Supplementary file 5 — Source data Fig. 4 [file 44319_2024_354_MOESM5_ESM.zip › Figure 4/4B/PD-L1.tif]

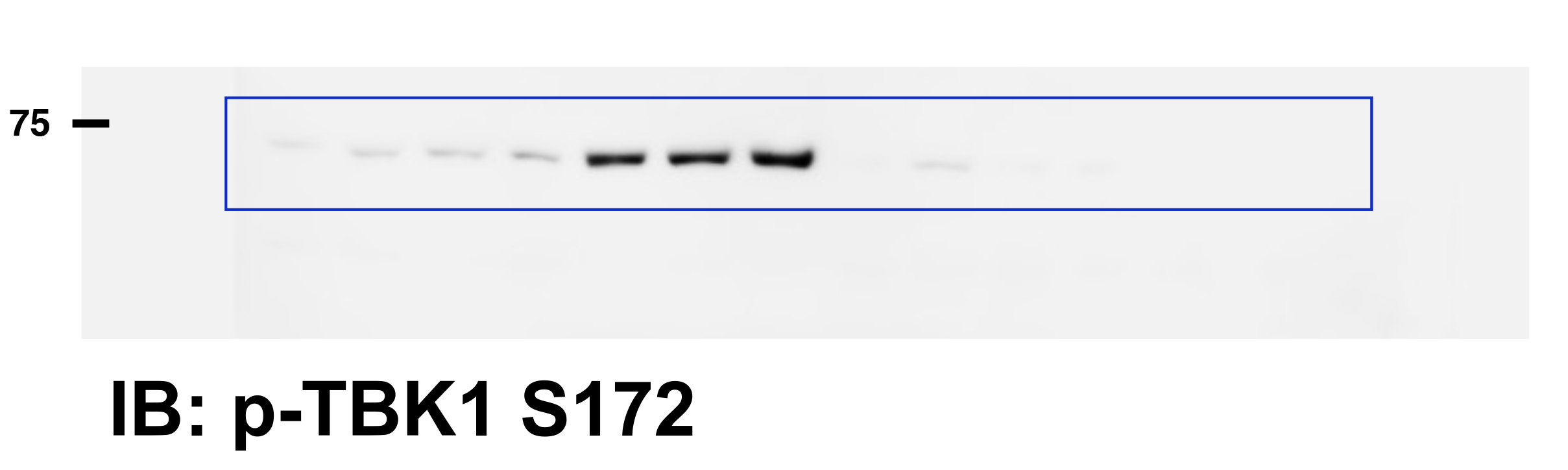

Supplement: Supplementary file 5 — Source data Fig. 4 [file 44319_2024_354_MOESM5_ESM.zip › Figure 4/4B/pTBK1.tif]

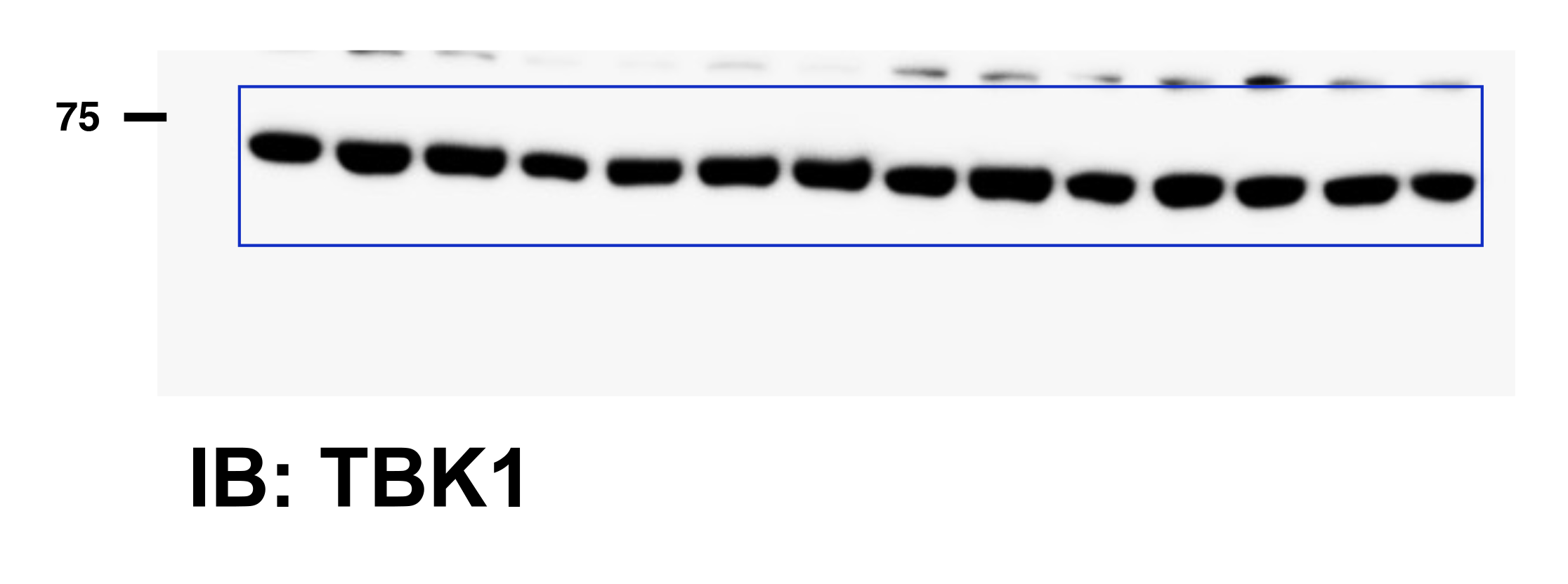

Supplement: Supplementary file 5 — Source data Fig. 4 [file 44319_2024_354_MOESM5_ESM.zip › Figure 4/4B/TBK1.tif]

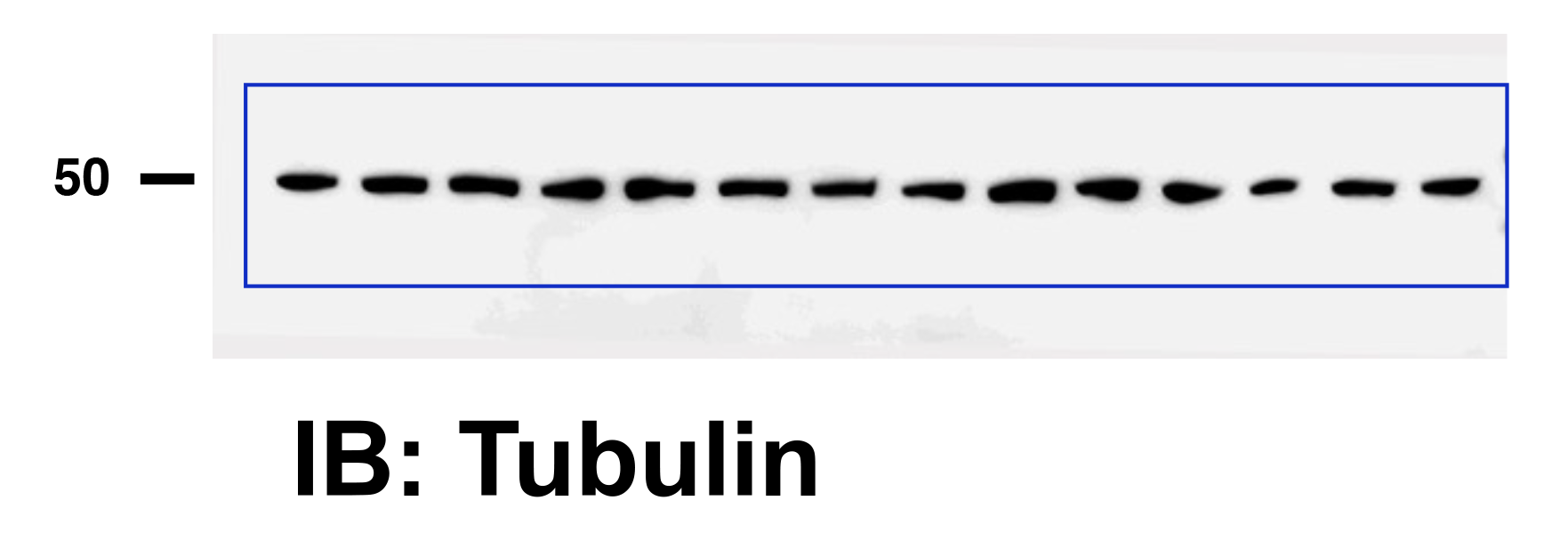

Supplement: Supplementary file 5 — Source data Fig. 4 [file 44319_2024_354_MOESM5_ESM.zip › Figure 4/4B/Tubulin.tif]

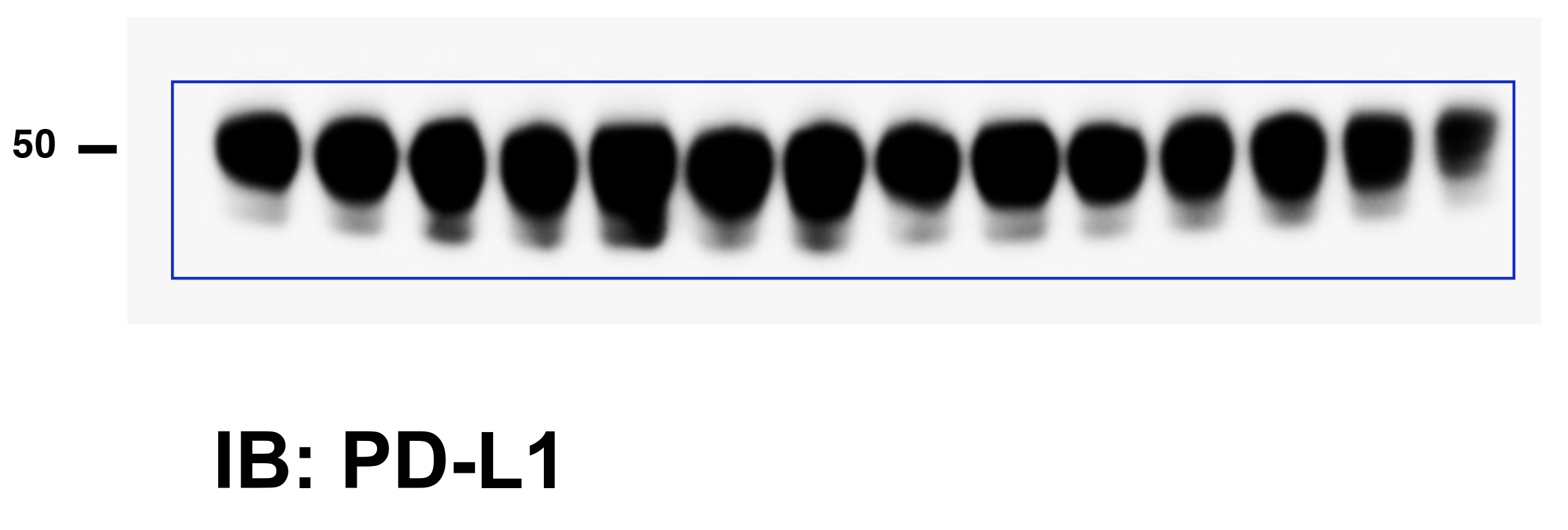

Supplement: Supplementary file 5 — Source data Fig. 4 [file 44319_2024_354_MOESM5_ESM.zip › Figure 4/4C/PD-L1.tif]

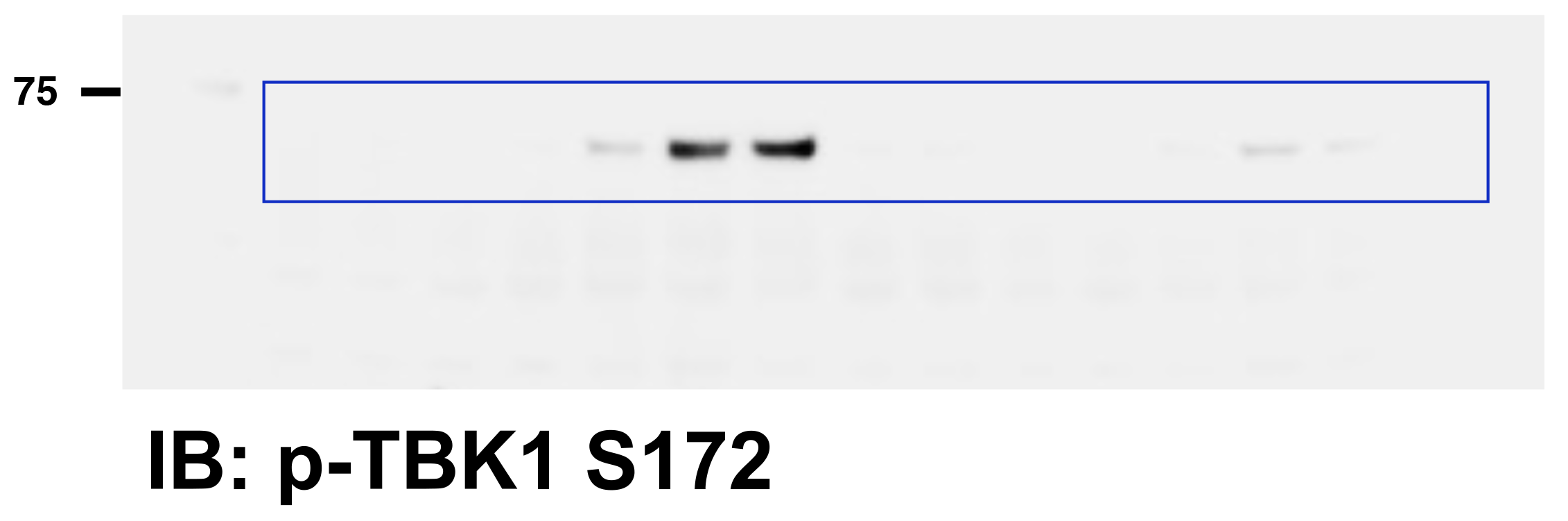

Supplement: Supplementary file 5 — Source data Fig. 4 [file 44319_2024_354_MOESM5_ESM.zip › Figure 4/4C/pTBK1.tif]

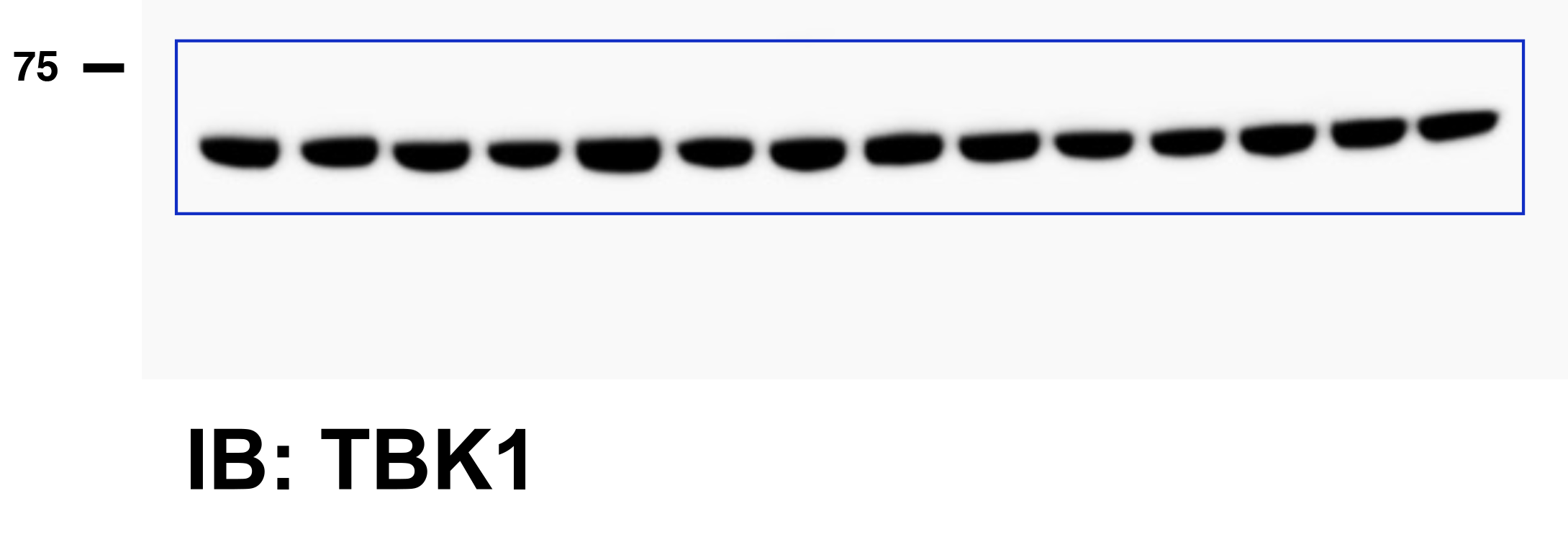

Supplement: Supplementary file 5 — Source data Fig. 4 [file 44319_2024_354_MOESM5_ESM.zip › Figure 4/4C/TBK1.tif]

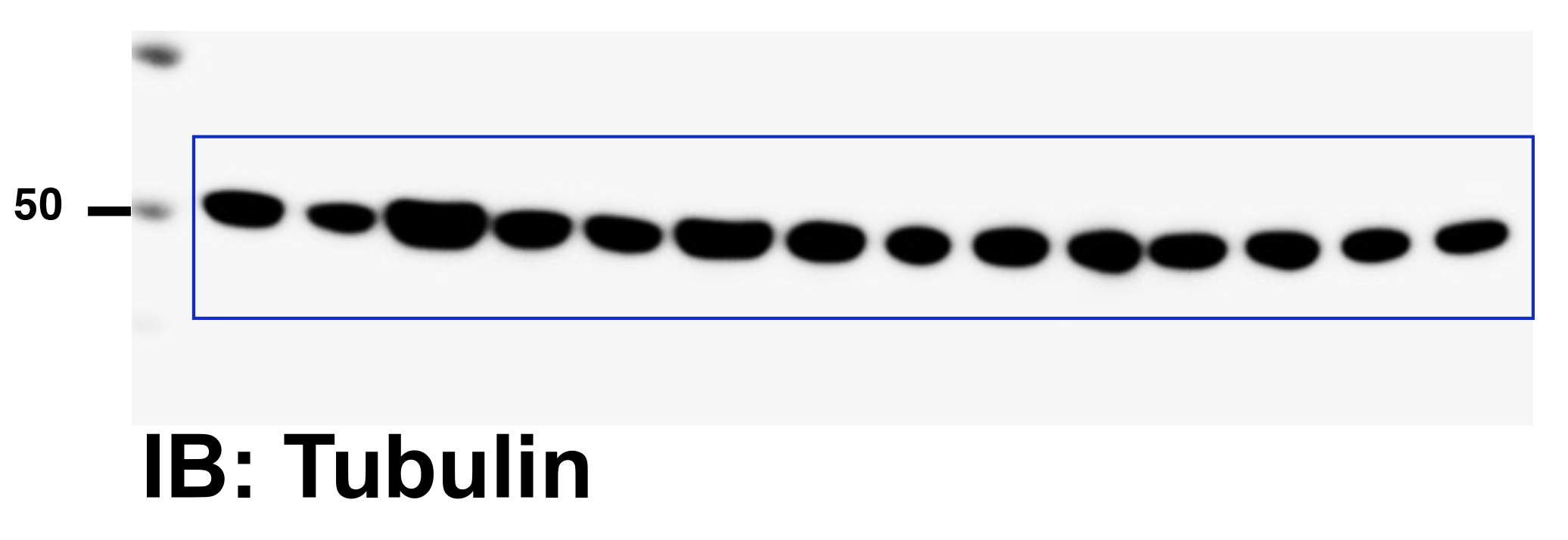

Supplement: Supplementary file 5 — Source data Fig. 4 [file 44319_2024_354_MOESM5_ESM.zip › Figure 4/4C/Tubulin.tif]

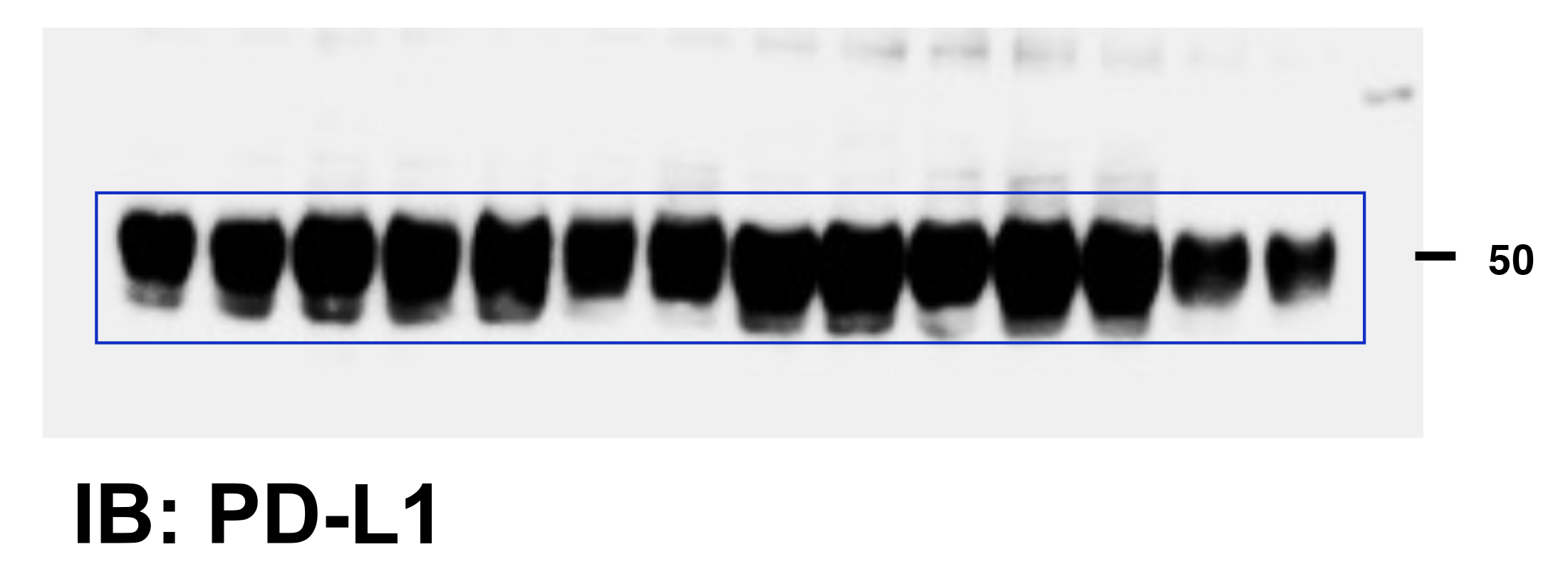

Supplement: Supplementary file 5 — Source data Fig. 4 [file 44319_2024_354_MOESM5_ESM.zip › Figure 4/4D/PD-L1.tif]

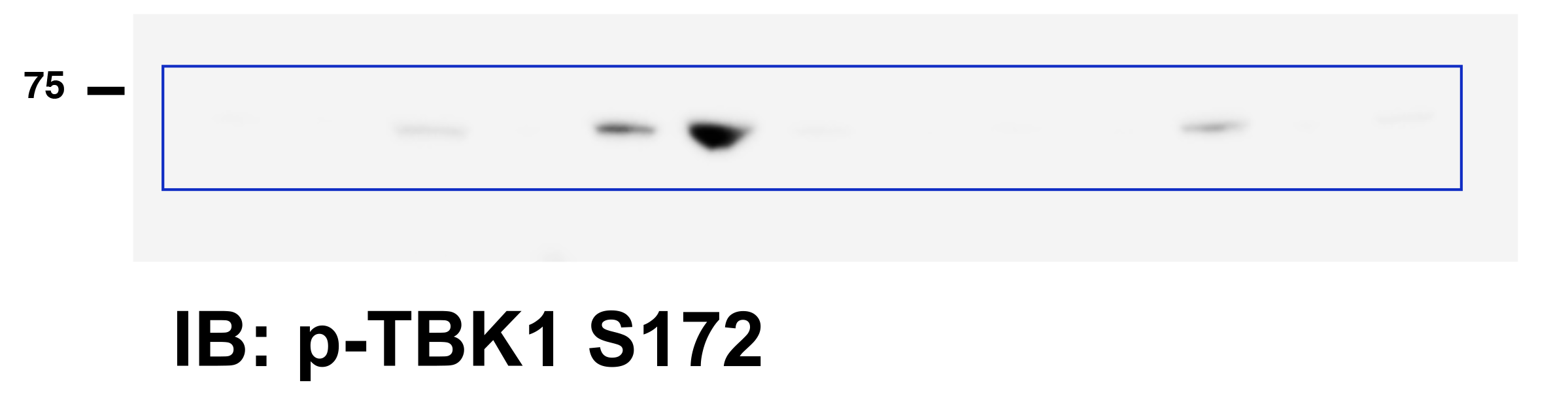

Supplement: Supplementary file 5 — Source data Fig. 4 [file 44319_2024_354_MOESM5_ESM.zip › Figure 4/4D/pTBK1.tif]

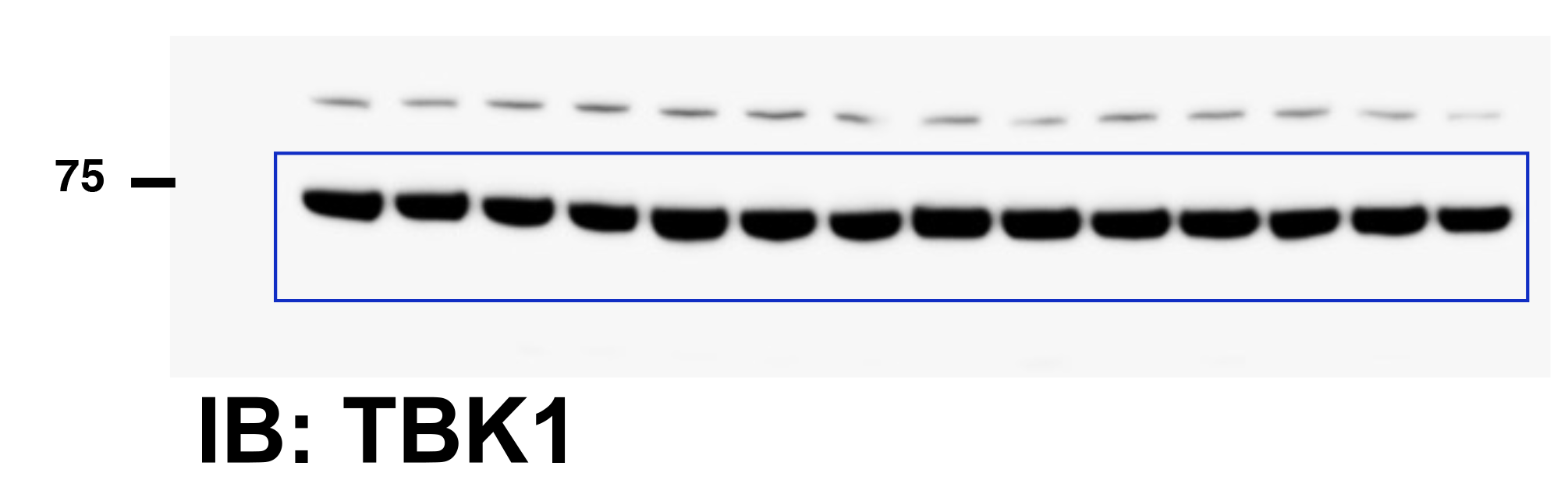

Supplement: Supplementary file 5 — Source data Fig. 4 [file 44319_2024_354_MOESM5_ESM.zip › Figure 4/4D/TBK1.tif]

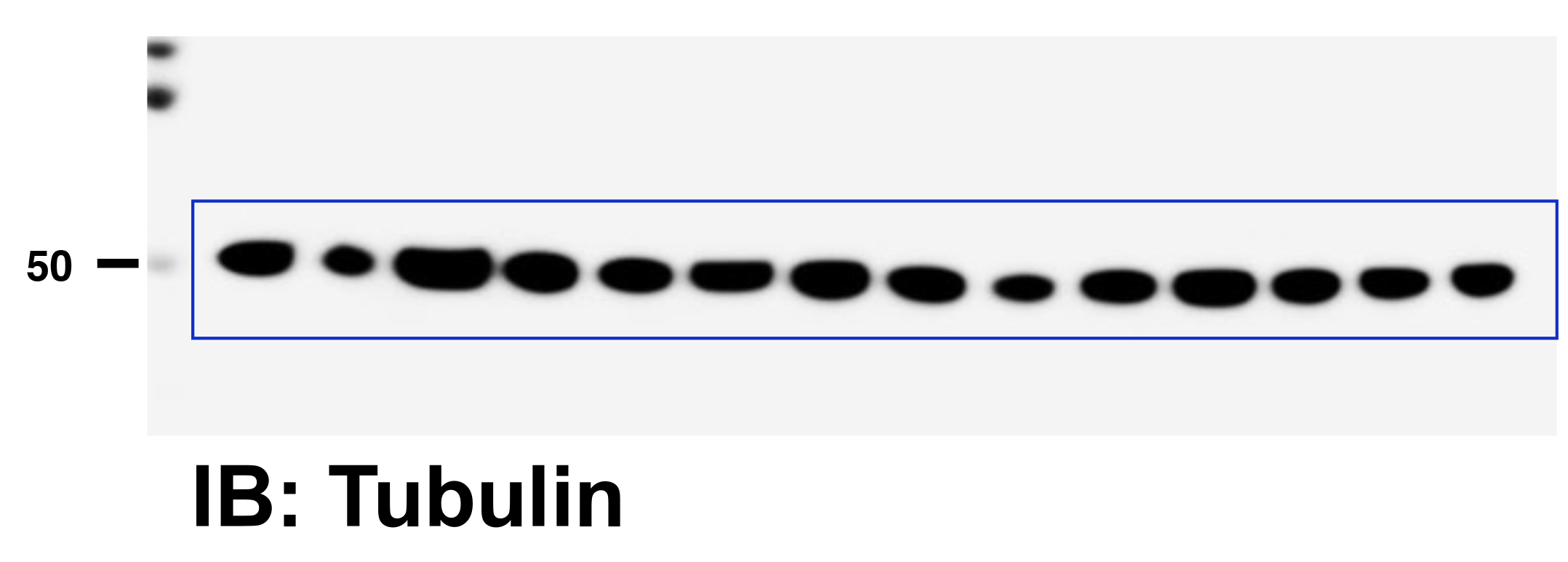

Supplement: Supplementary file 5 — Source data Fig. 4 [file 44319_2024_354_MOESM5_ESM.zip › Figure 4/4D/Tubulin.tif]

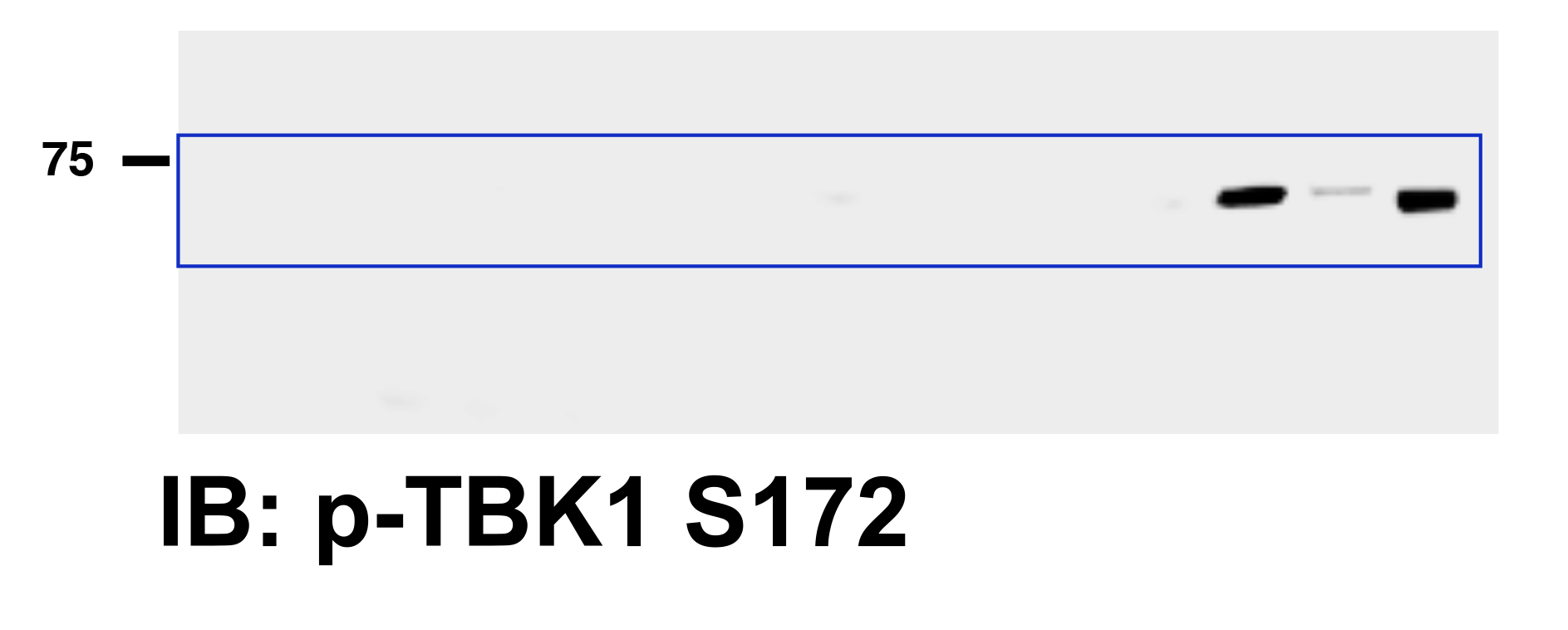

Supplement: Supplementary file 5 — Source data Fig. 4 [file 44319_2024_354_MOESM5_ESM.zip › Figure 4/4E/pTBK1.tif]

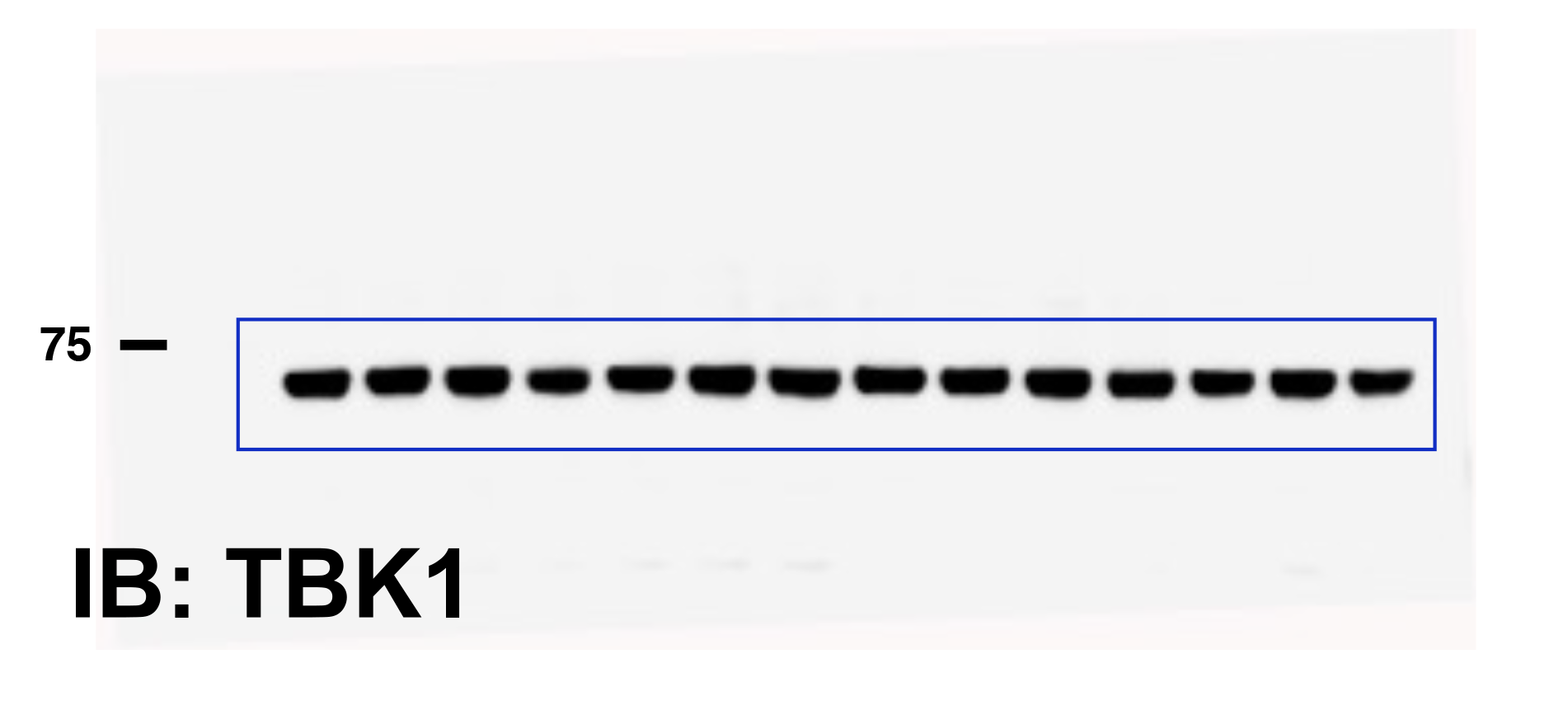

Supplement: Supplementary file 5 — Source data Fig. 4 [file 44319_2024_354_MOESM5_ESM.zip › Figure 4/4E/TBK1.tif]

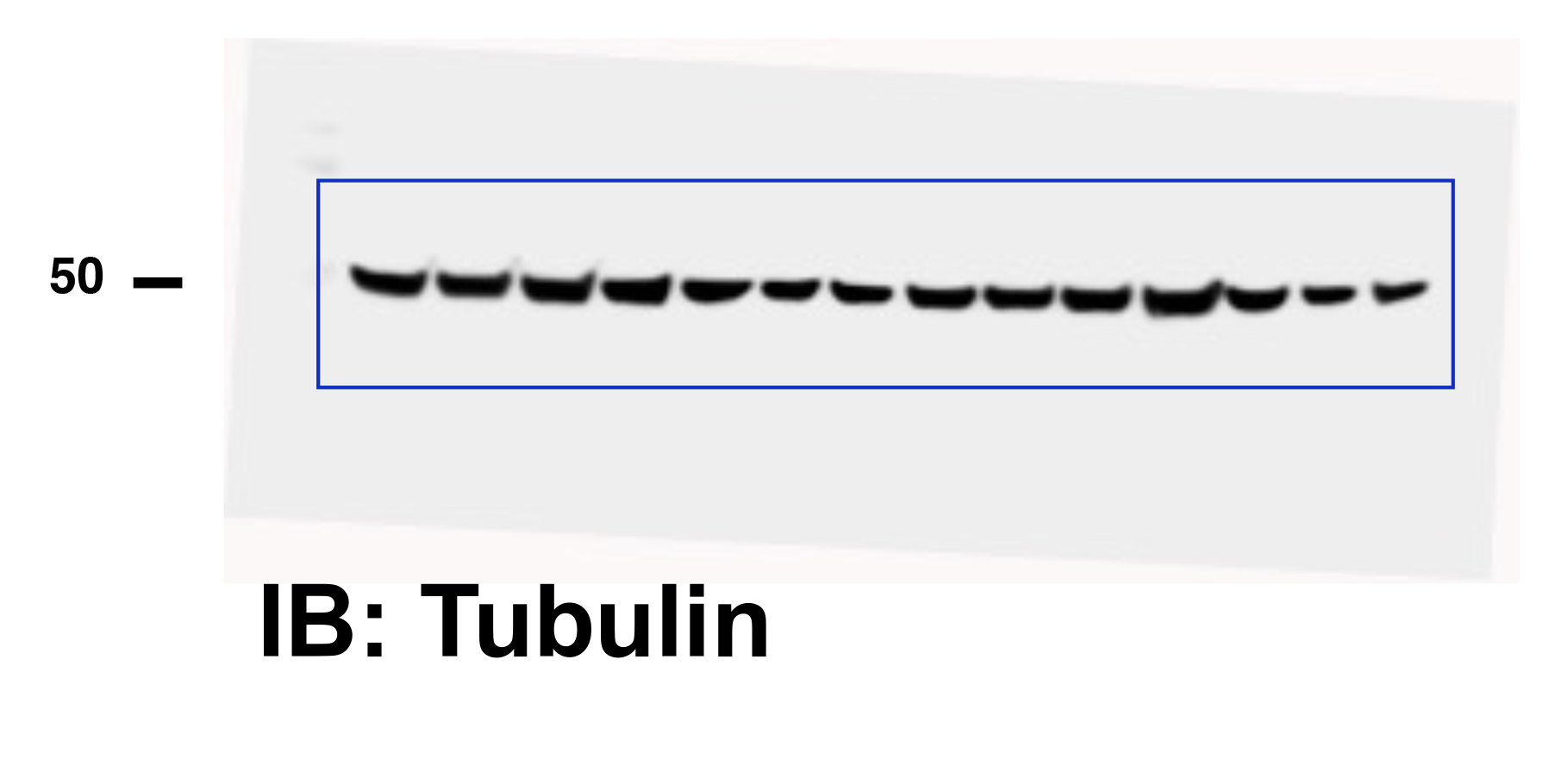

Supplement: Supplementary file 5 — Source data Fig. 4 [file 44319_2024_354_MOESM5_ESM.zip › Figure 4/4E/Tubulin.tif]

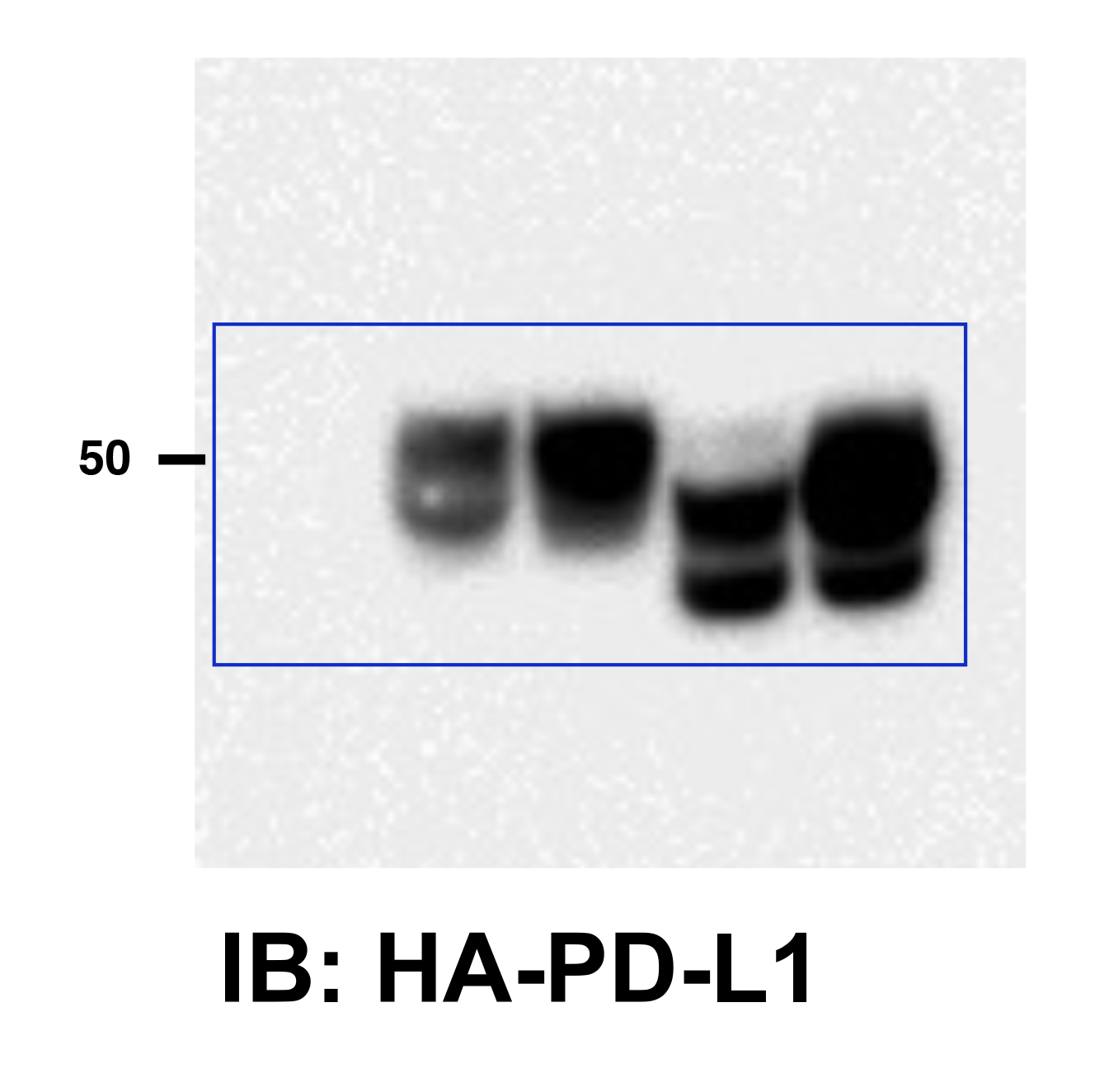

Supplement: Supplementary file 7 — Source data Fig. 6 [file 44319_2024_354_MOESM7_ESM.zip › Figure 6/6A/HA-PD-L1.tif]

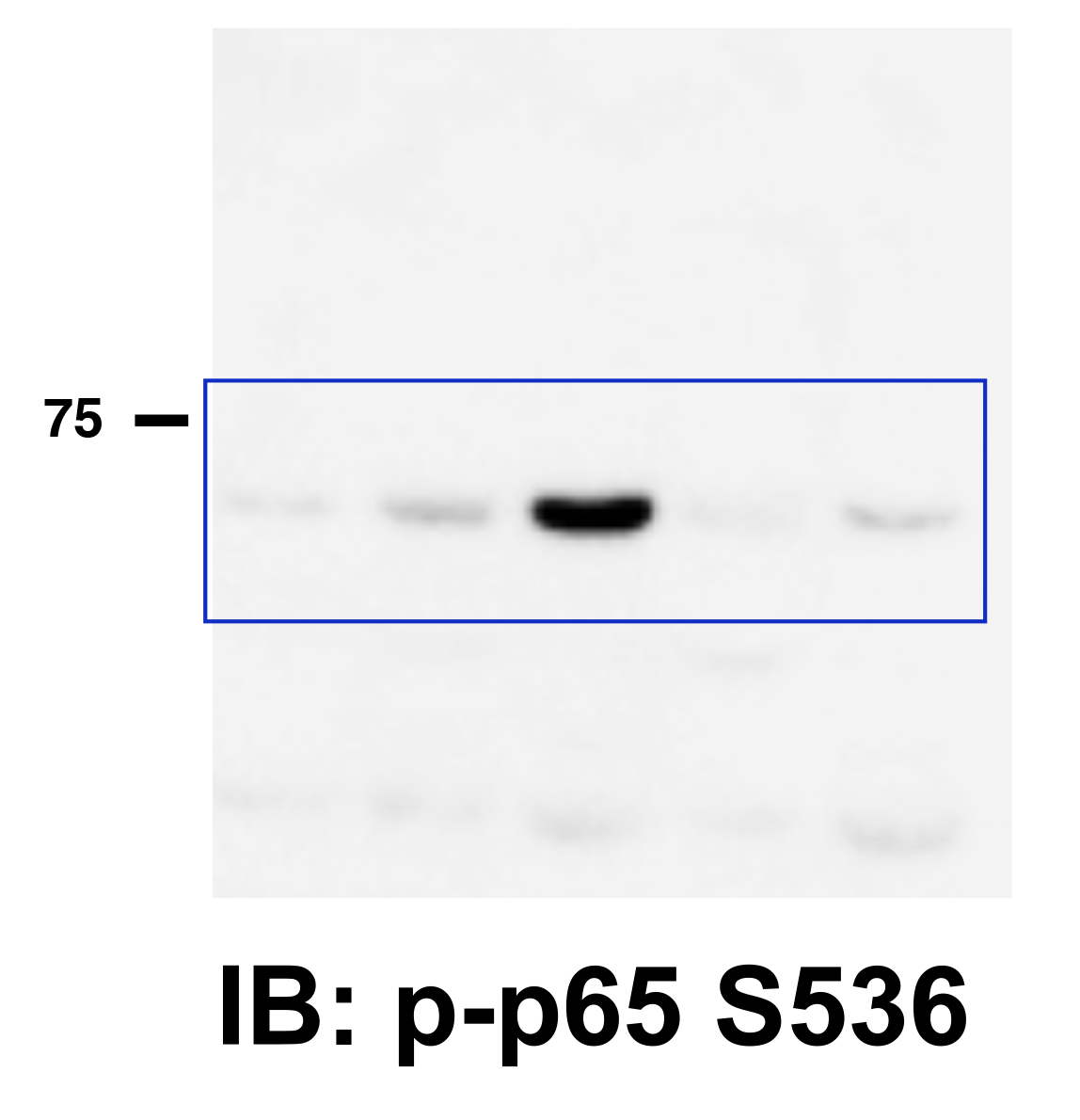

Supplement: Supplementary file 7 — Source data Fig. 6 [file 44319_2024_354_MOESM7_ESM.zip › Figure 6/6A/p-p65.tif]

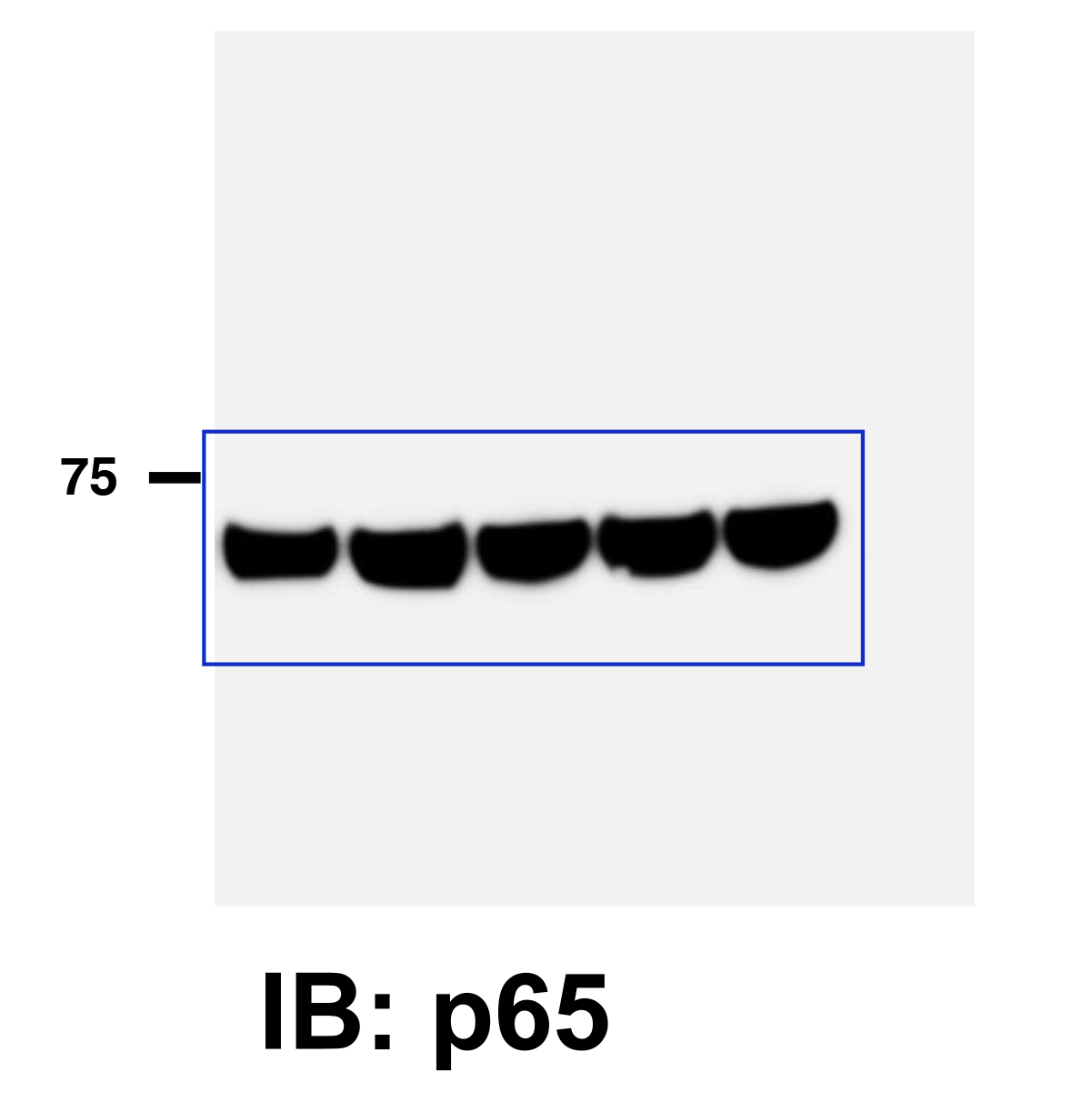

Supplement: Supplementary file 7 — Source data Fig. 6 [file 44319_2024_354_MOESM7_ESM.zip › Figure 6/6A/p65.tif]

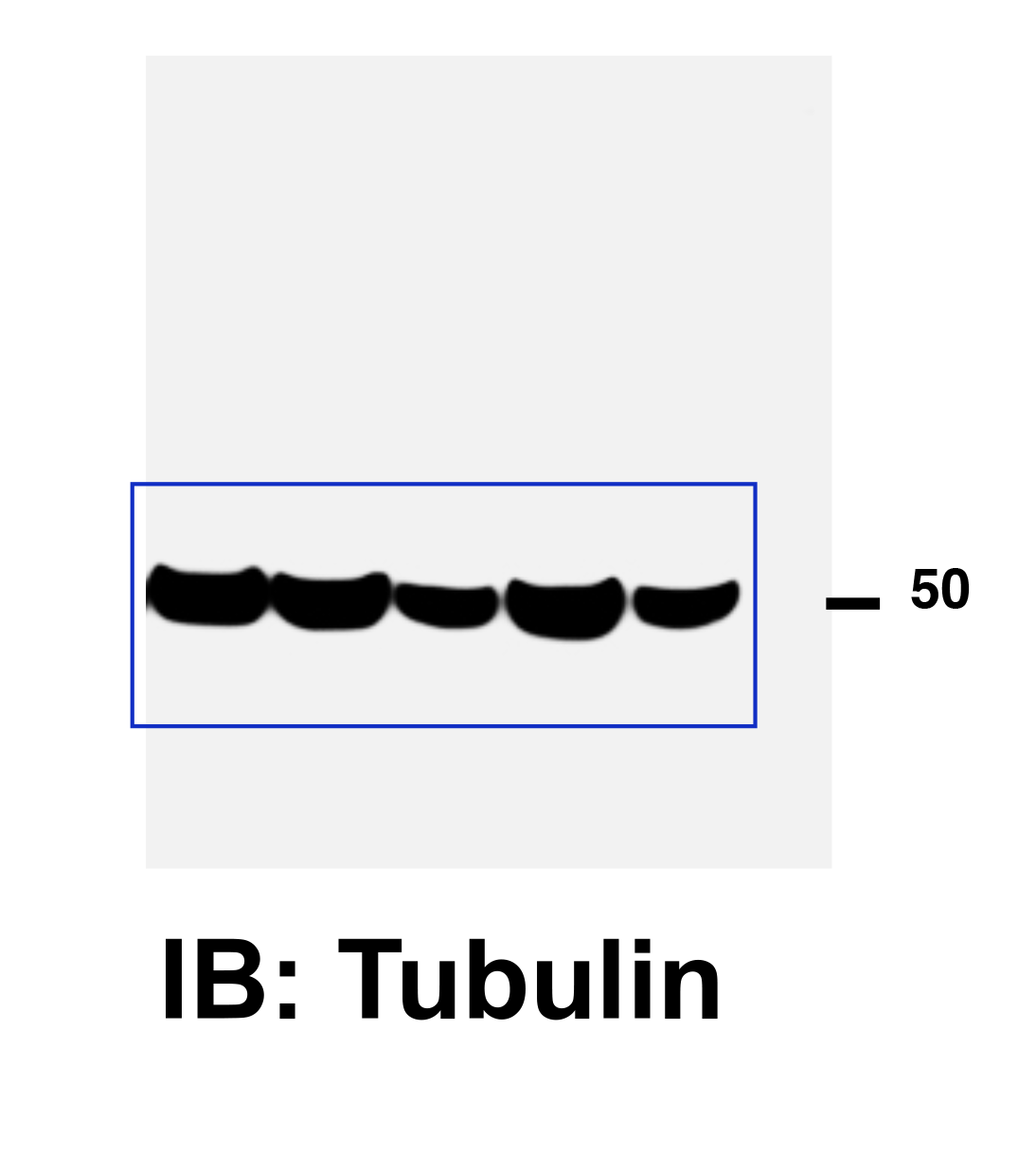

Supplement: Supplementary file 7 — Source data Fig. 6 [file 44319_2024_354_MOESM7_ESM.zip › Figure 6/6A/Tubulin.tif]

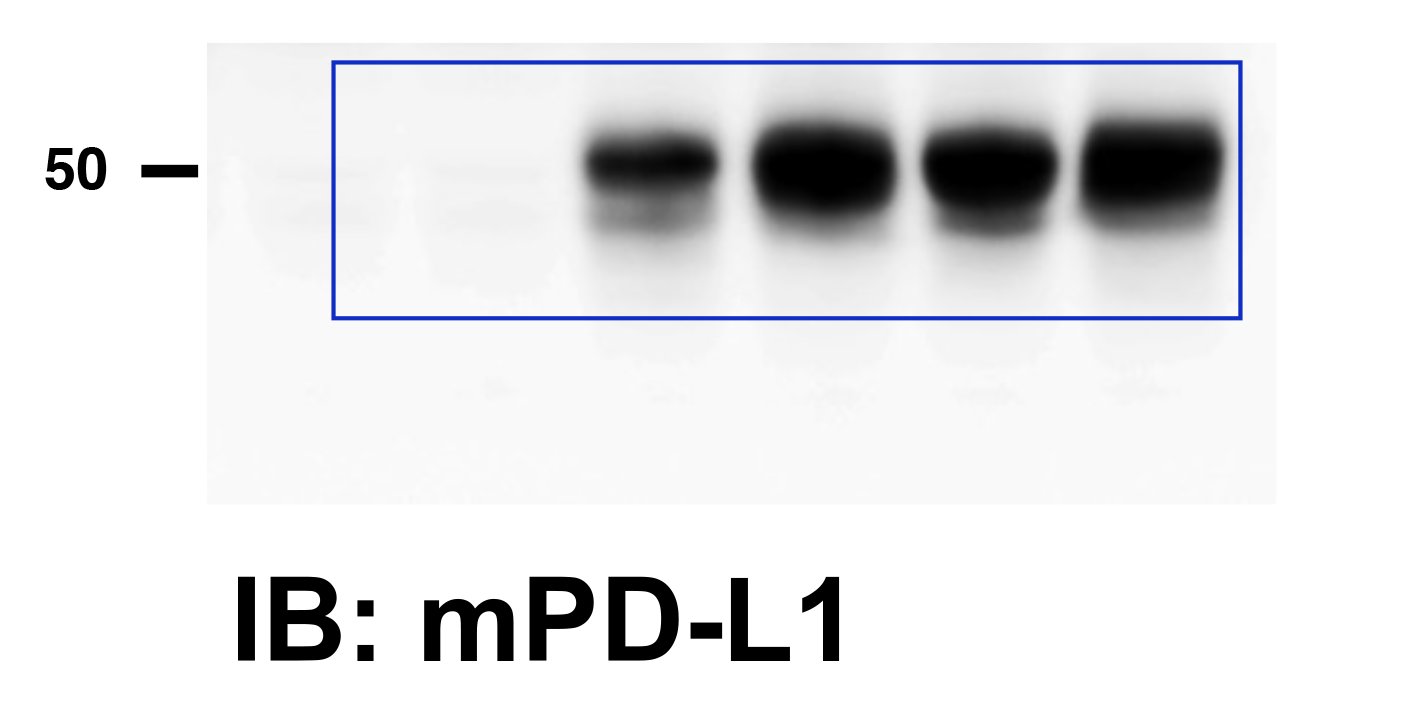

Supplement: Supplementary file 7 — Source data Fig. 6 [file 44319_2024_354_MOESM7_ESM.zip › Figure 6/6C/mPD-L1.tif]

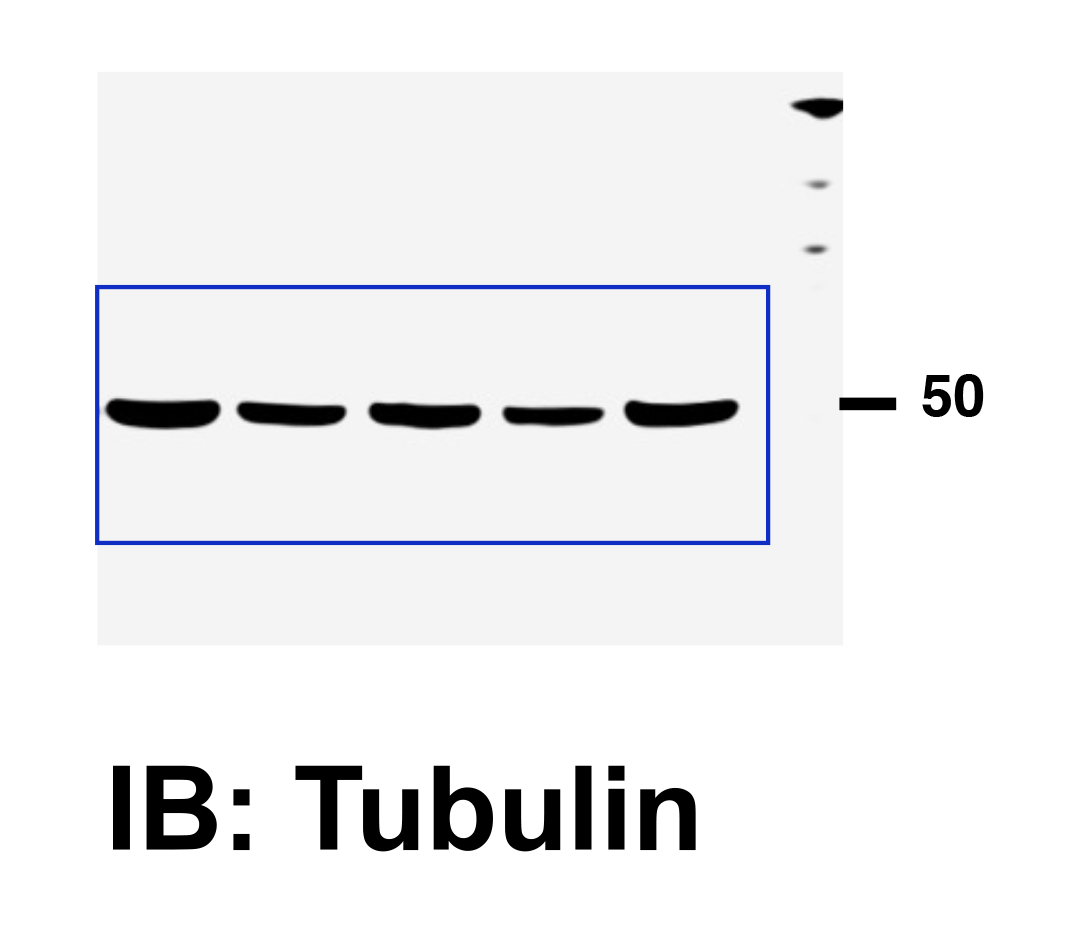

Supplement: Supplementary file 7 — Source data Fig. 6 [file 44319_2024_354_MOESM7_ESM.zip › Figure 6/6C/Tubulin.tif]

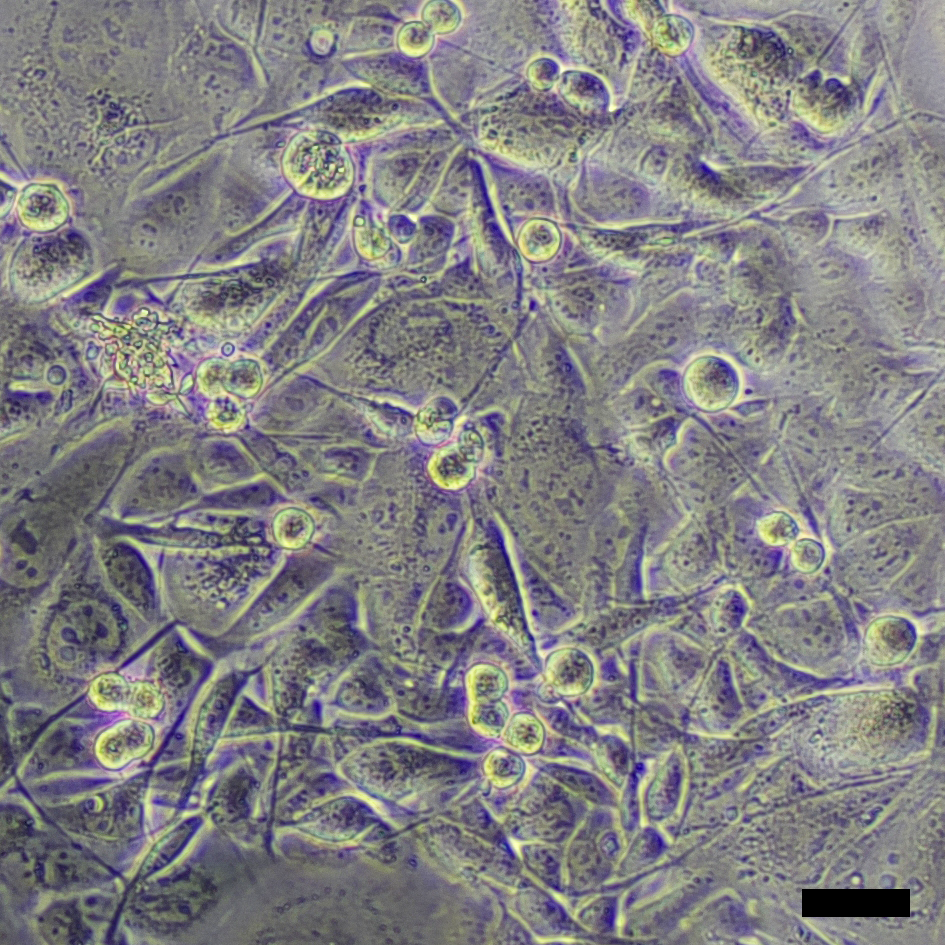

Supplement: Supplementary file 8 — Figure EV1-5 Source Data [file 44319_2024_354_MOESM8_ESM.zip › Figure EV1-5/Figure EV3/EV3A/WT_IR day 3.tif]

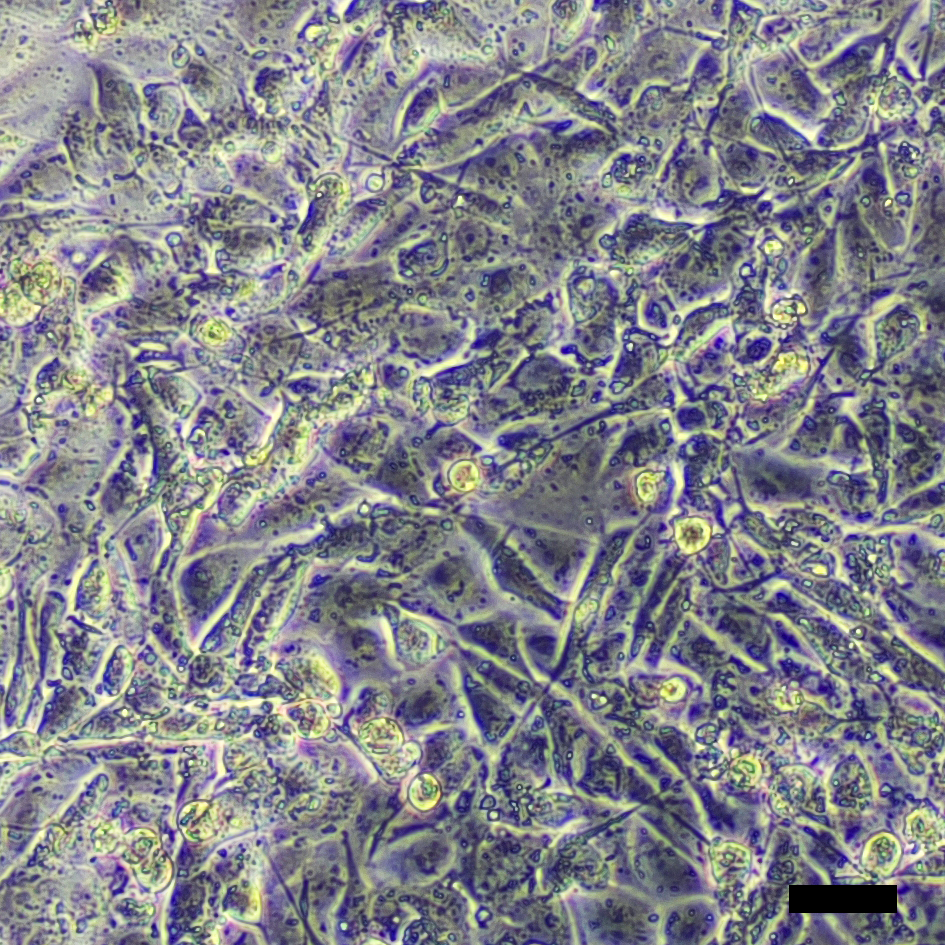

Supplement: Supplementary file 8 — Figure EV1-5 Source Data [file 44319_2024_354_MOESM8_ESM.zip › Figure EV1-5/Figure EV3/EV3A/KO_IR day6.tif]

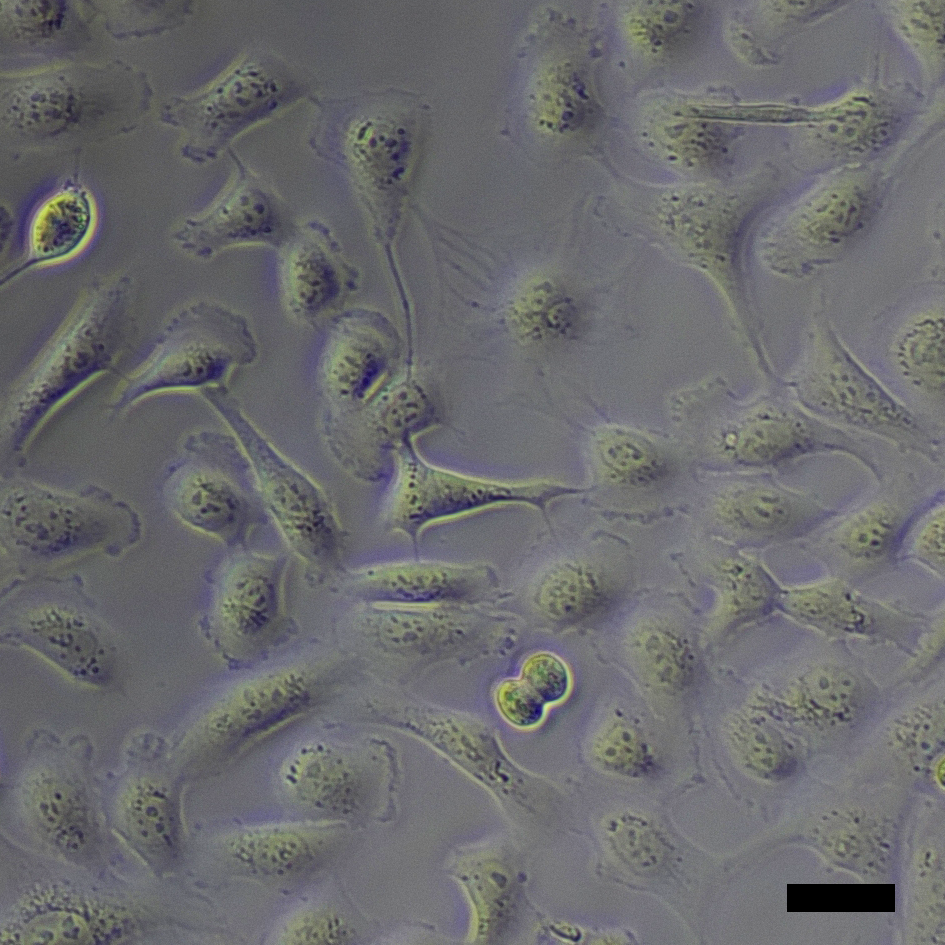

Supplement: Supplementary file 8 — Figure EV1-5 Source Data [file 44319_2024_354_MOESM8_ESM.zip › Figure EV1-5/Figure EV3/EV3A/KO day0.tif]

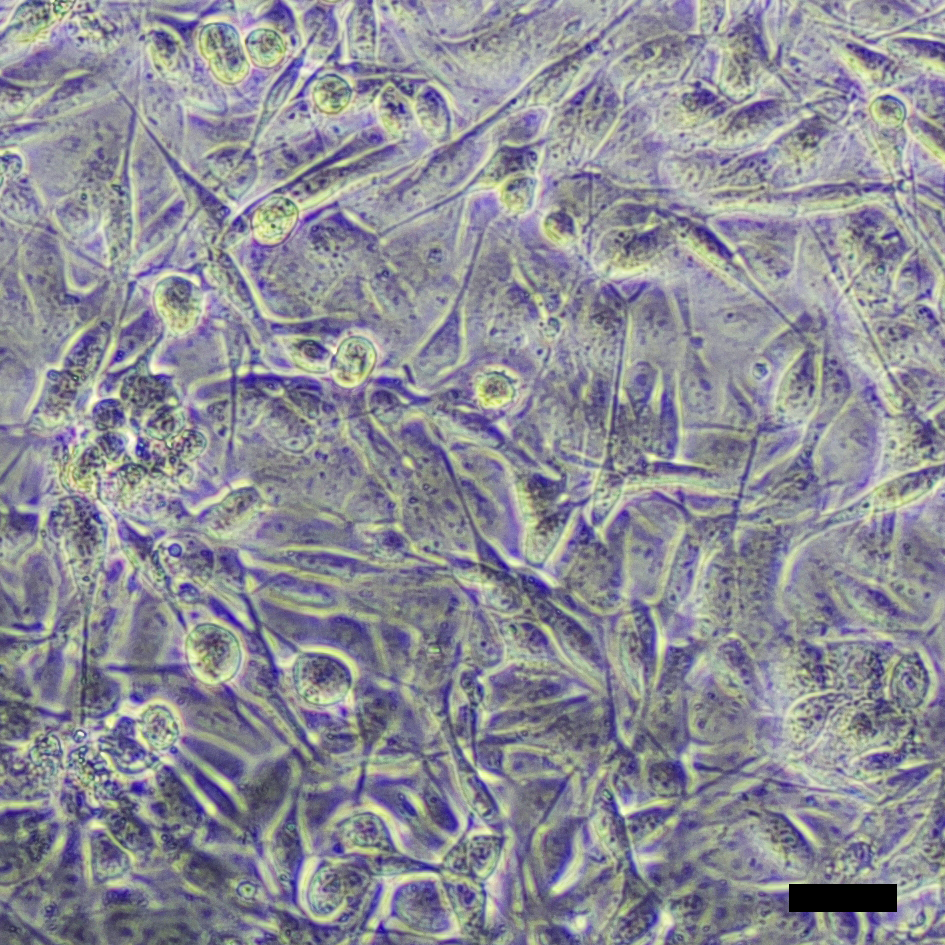

Supplement: Supplementary file 8 — Figure EV1-5 Source Data [file 44319_2024_354_MOESM8_ESM.zip › Figure EV1-5/Figure EV3/EV3A/KO_IR day3.tif]

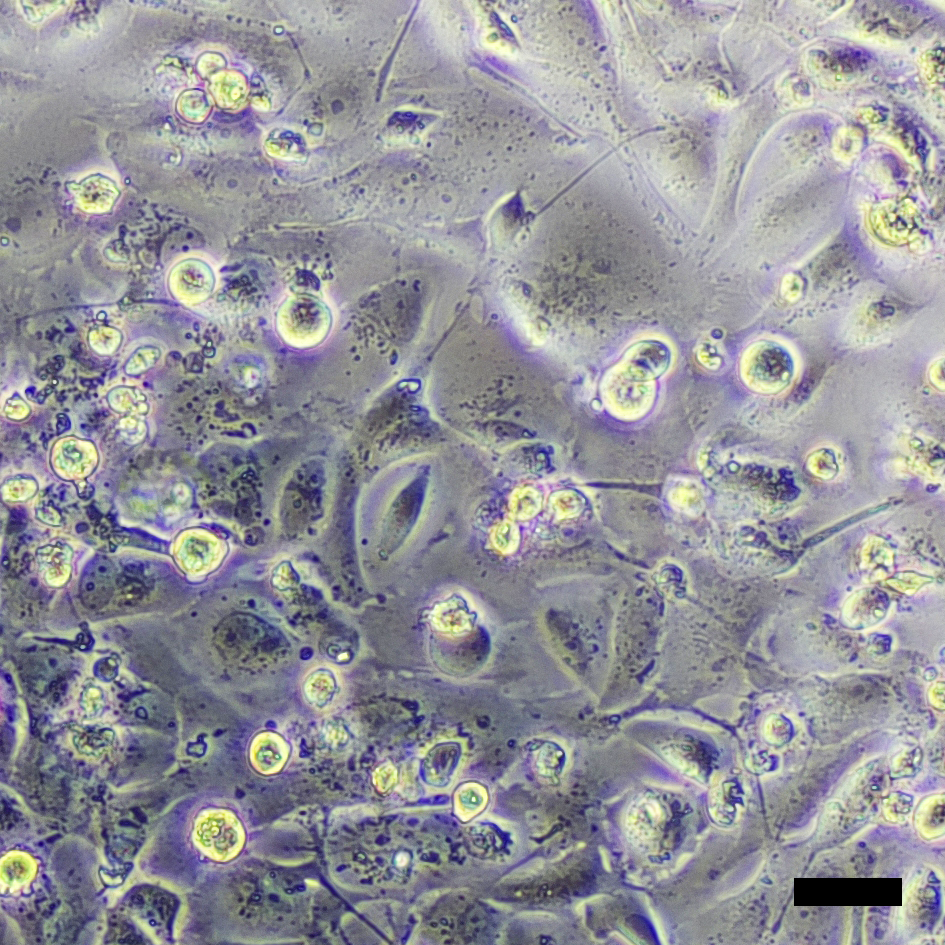

Supplement: Supplementary file 8 — Figure EV1-5 Source Data [file 44319_2024_354_MOESM8_ESM.zip › Figure EV1-5/Figure EV3/EV3A/WT_IR day6.tif]

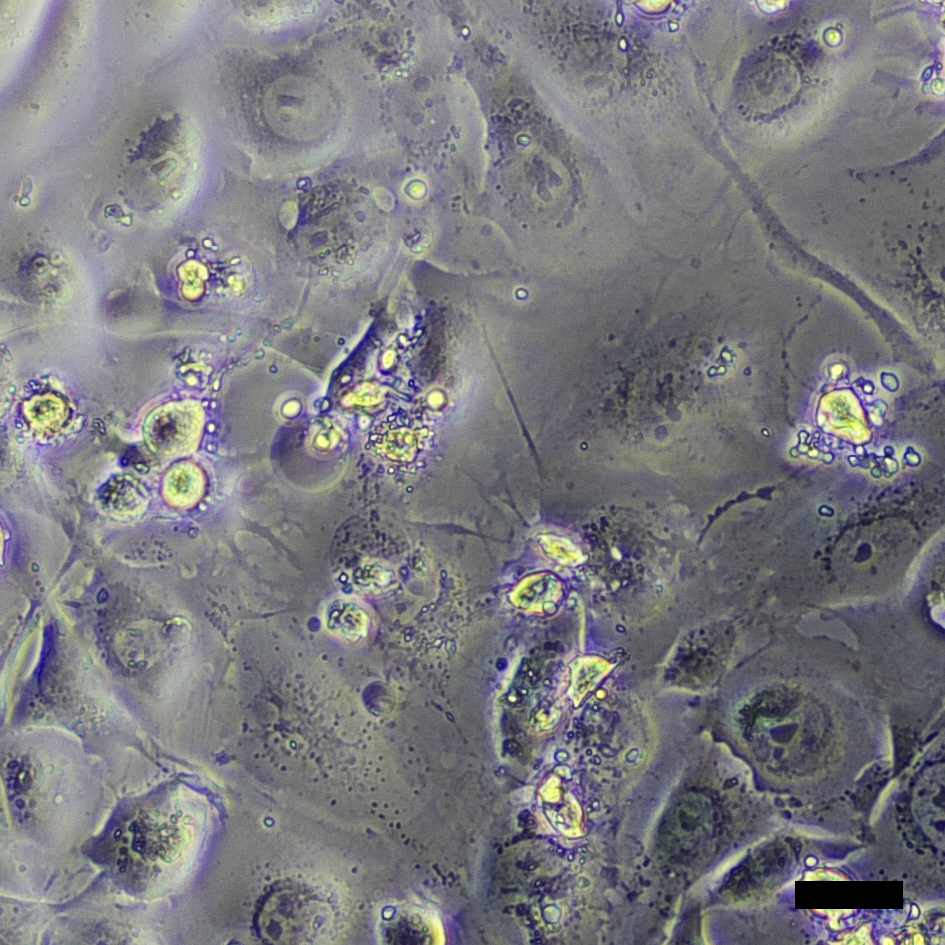

Supplement: Supplementary file 8 — Figure EV1-5 Source Data [file 44319_2024_354_MOESM8_ESM.zip › Figure EV1-5/Figure EV3/EV3A/WT_NCS day 3.tif]

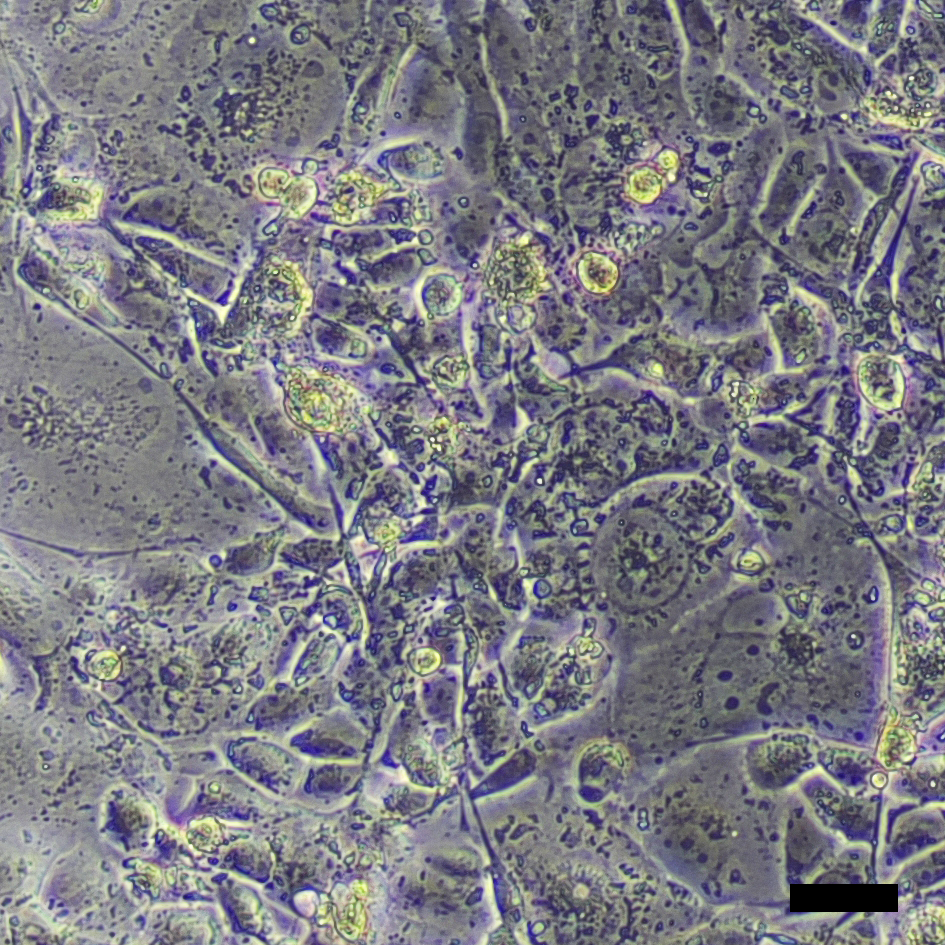

Supplement: Supplementary file 8 — Figure EV1-5 Source Data [file 44319_2024_354_MOESM8_ESM.zip › Figure EV1-5/Figure EV3/EV3A/KO_NCS day6.tif]

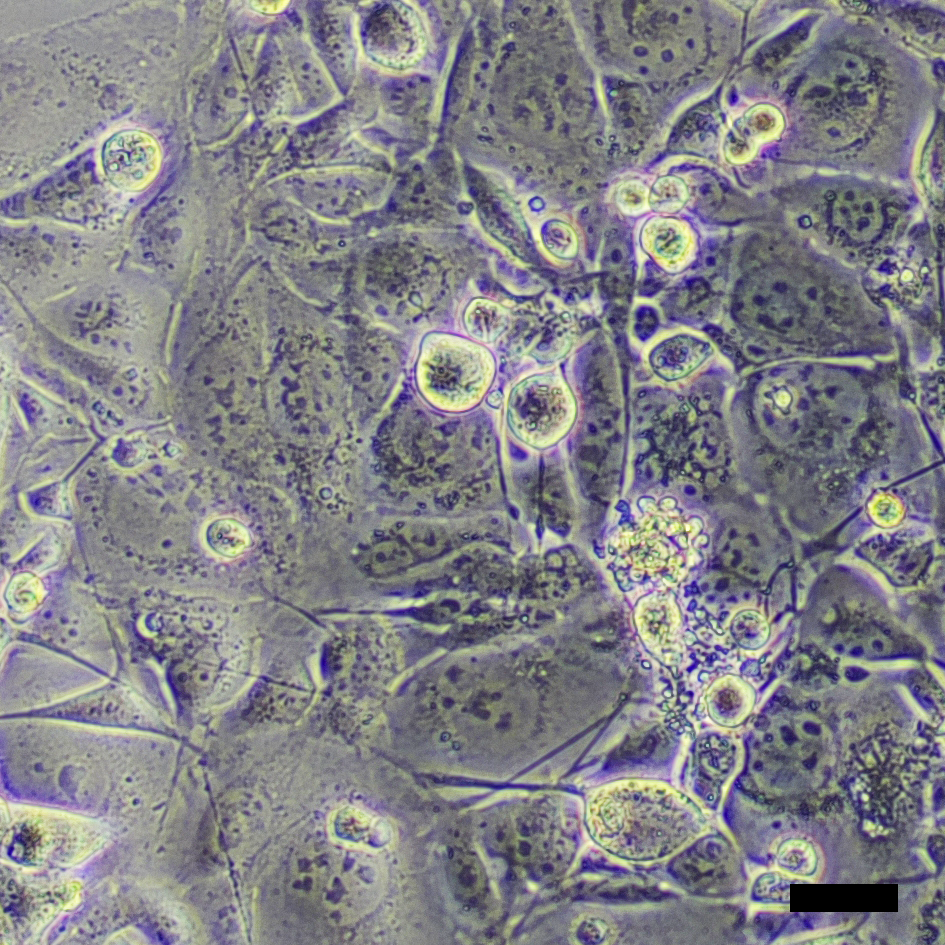

Supplement: Supplementary file 8 — Figure EV1-5 Source Data [file 44319_2024_354_MOESM8_ESM.zip › Figure EV1-5/Figure EV3/EV3A/KO_NCS day3.tif]

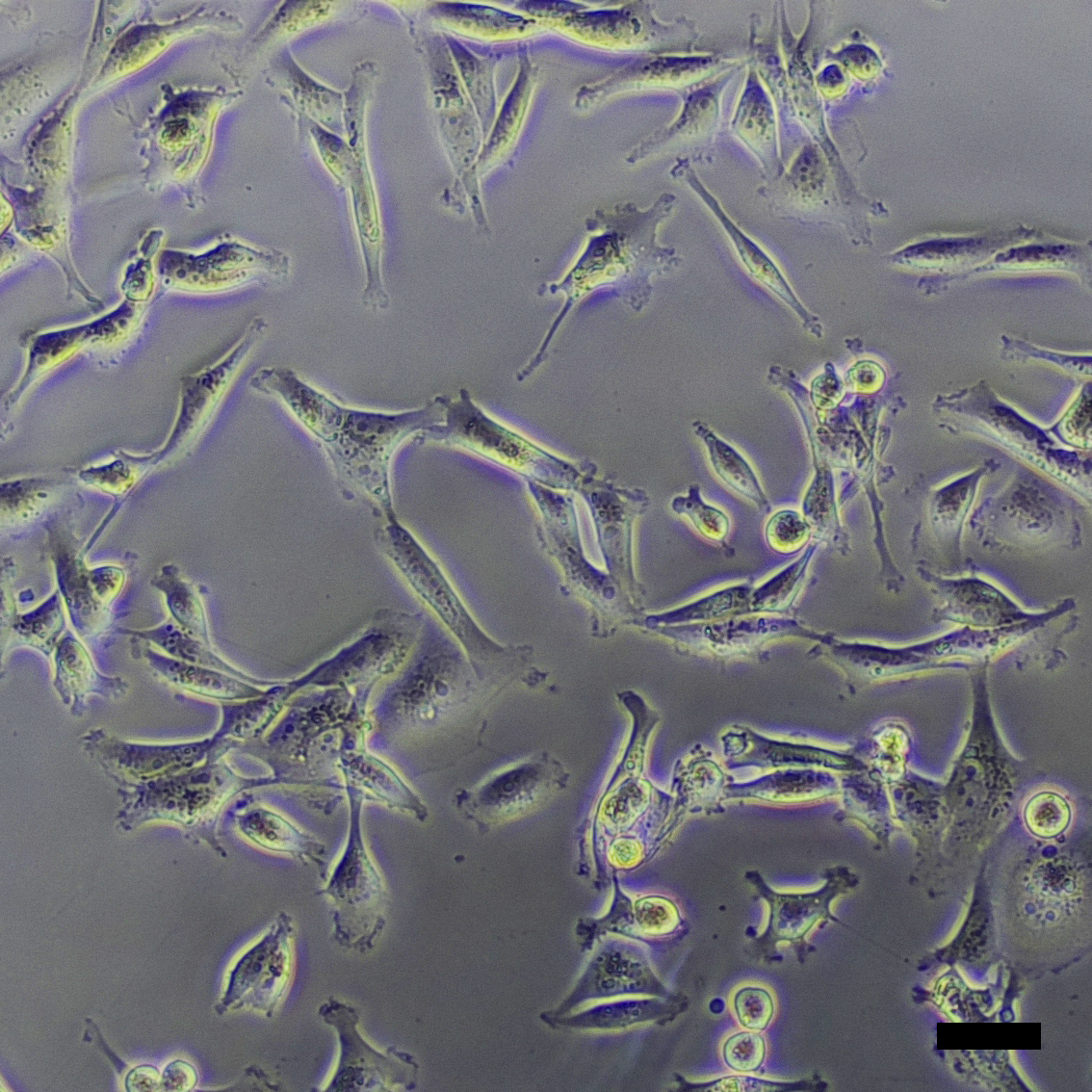

Supplement: Supplementary file 8 — Figure EV1-5 Source Data [file 44319_2024_354_MOESM8_ESM.zip › Figure EV1-5/Figure EV3/EV3A/WT day 0.tif]

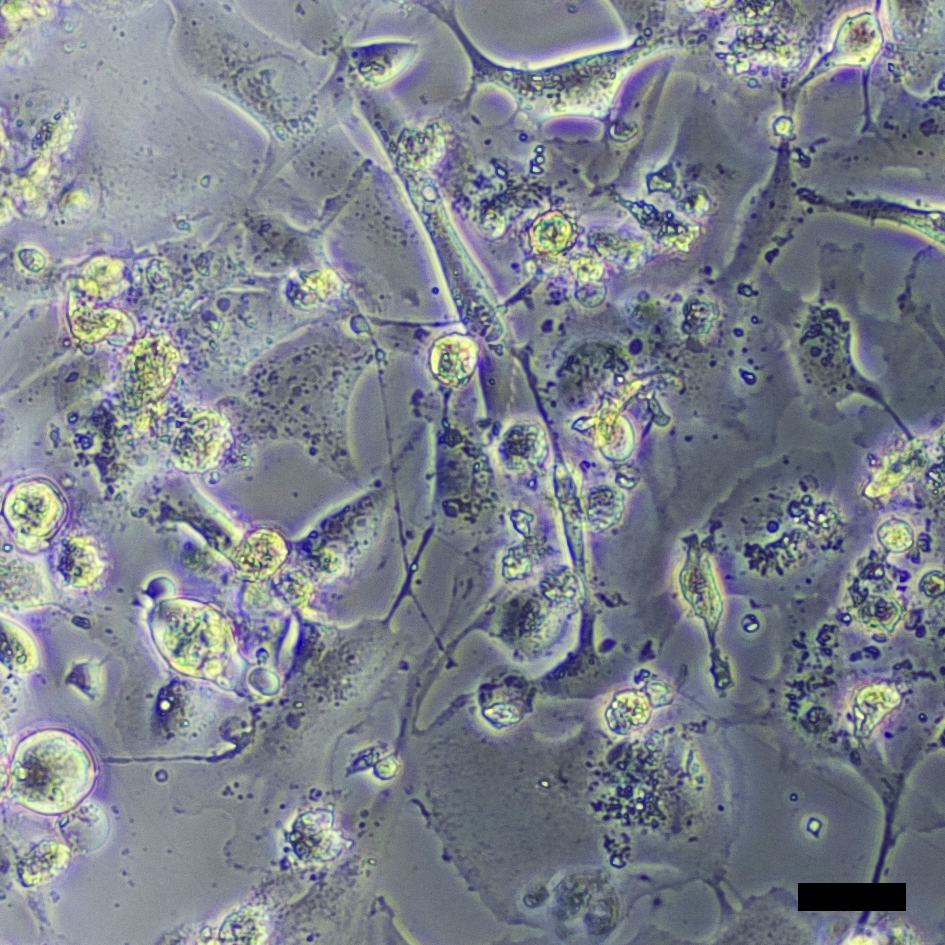

Supplement: Supplementary file 8 — Figure EV1-5 Source Data [file 44319_2024_354_MOESM8_ESM.zip › Figure EV1-5/Figure EV3/EV3A/WT_NCS day6.tif]

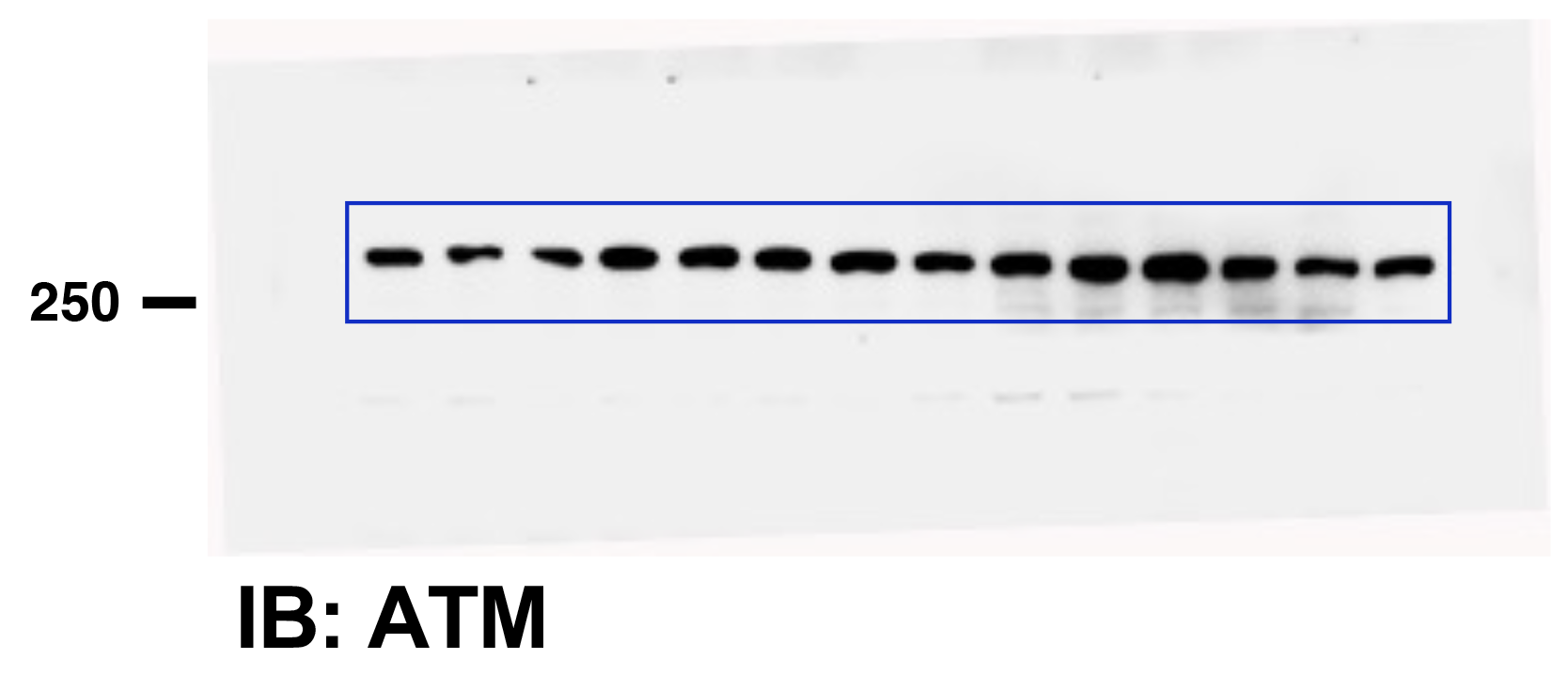

Supplement: Supplementary file 8 — Figure EV1-5 Source Data [file 44319_2024_354_MOESM8_ESM.zip › Figure EV1-5/Figure EV3/EV3D/ATM.tif]

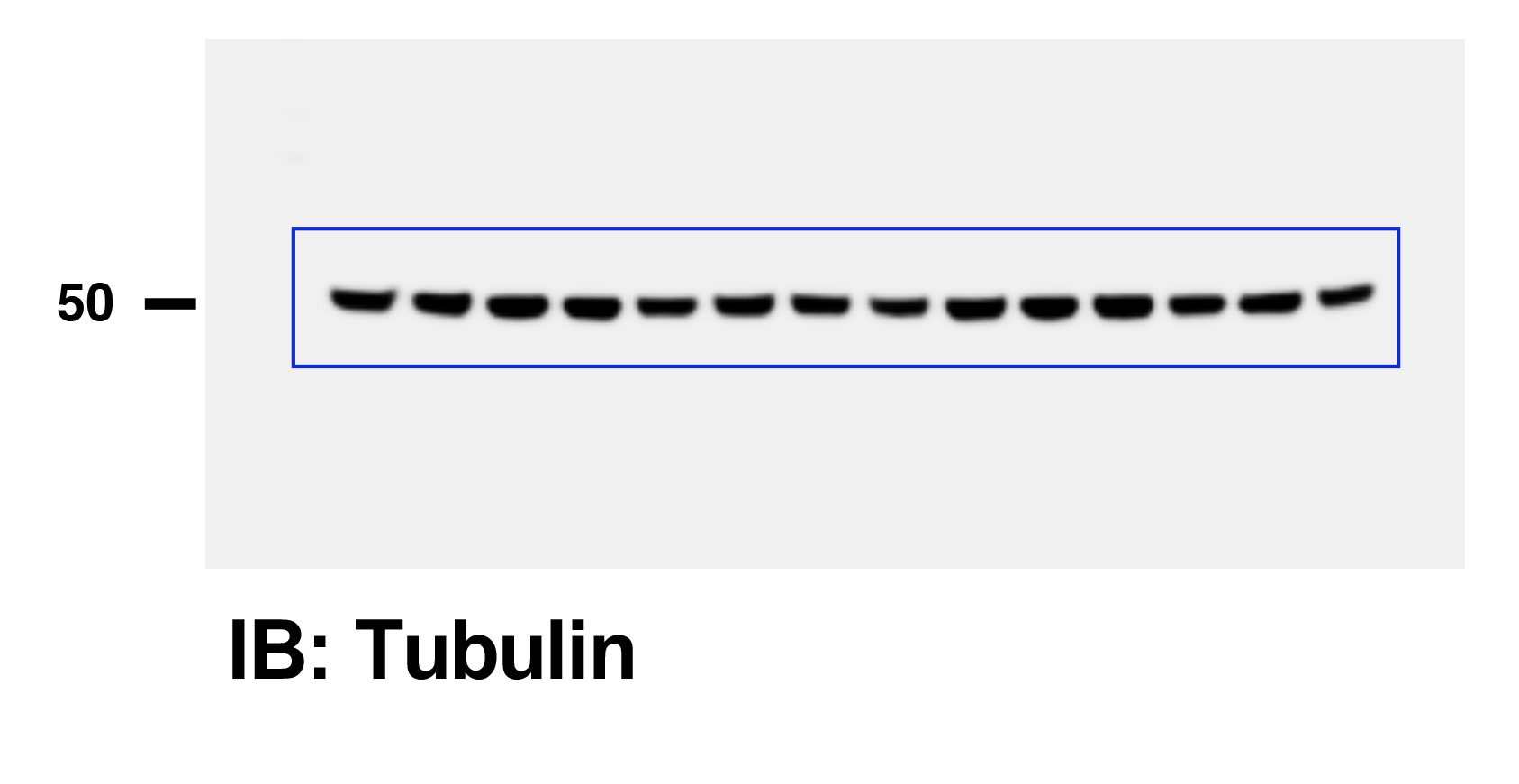

Supplement: Supplementary file 8 — Figure EV1-5 Source Data [file 44319_2024_354_MOESM8_ESM.zip › Figure EV1-5/Figure EV3/EV3D/Tubulin.tif]

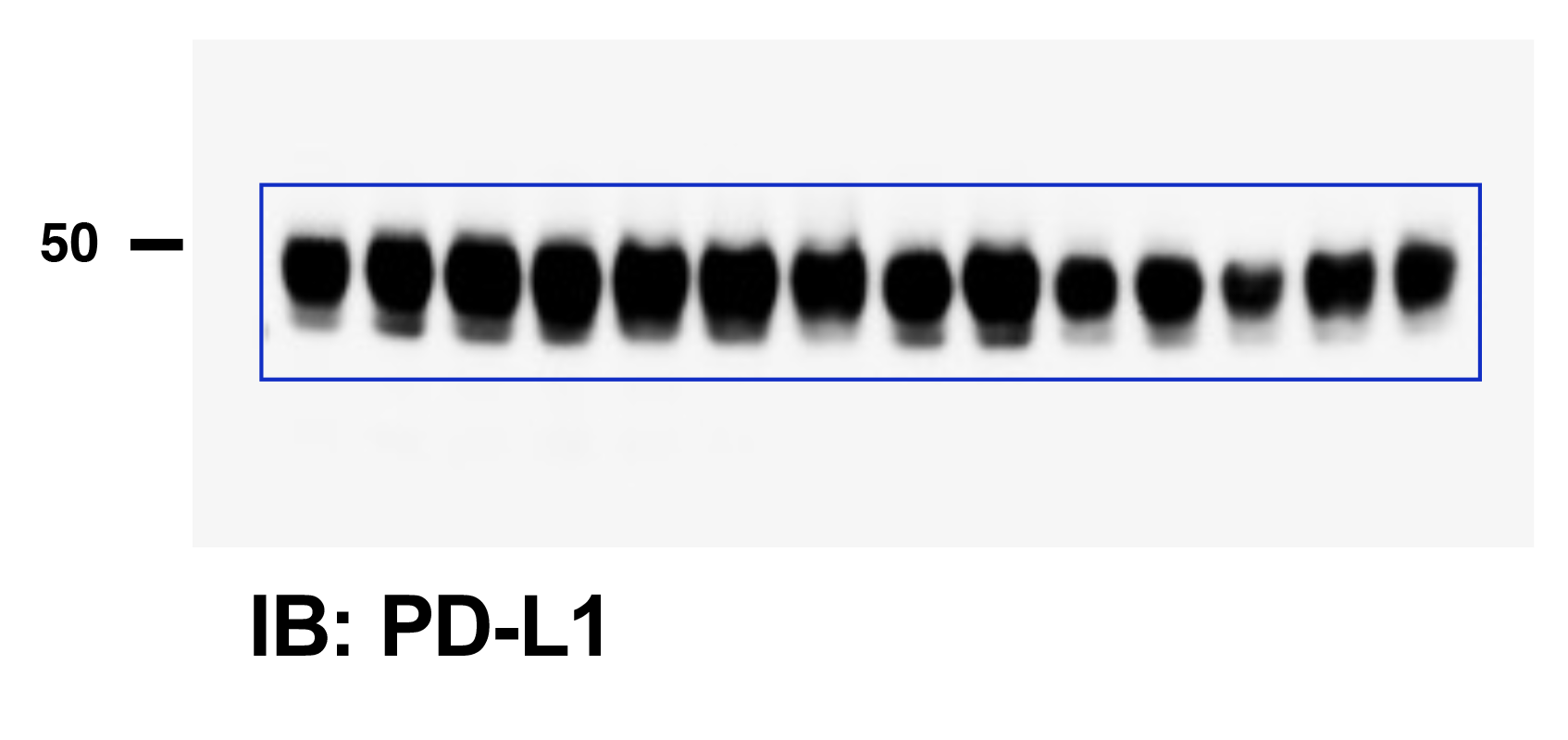

Supplement: Supplementary file 8 — Figure EV1-5 Source Data [file 44319_2024_354_MOESM8_ESM.zip › Figure EV1-5/Figure EV3/EV3D/PD-L1.tif]
