# Supplementary figures and images for: Nuclear PD-L1 triggers tumour-associated inflammation upon DNA damage (part 3 of 3)
Source: EMBO Rep. 2025 Jan 2;26(3):635–55. doi: 10.1038/s44319-024-00354-9 (PMC11811057; doi:10.1038/s44319-024-00354-9)

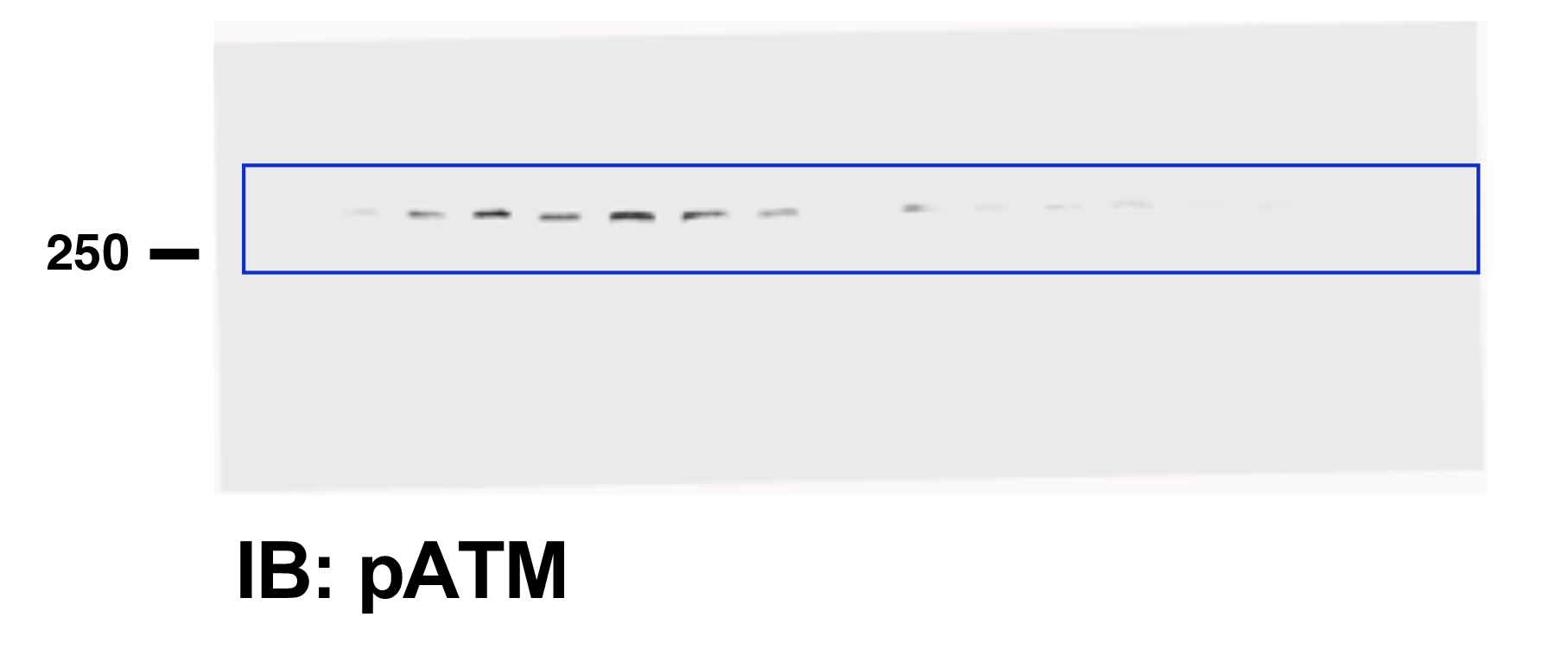

Supplement: Supplementary file 8 — Figure EV1-5 Source Data [file 44319_2024_354_MOESM8_ESM.zip › Figure EV1-5/Figure EV3/EV3D/pATM.tif]

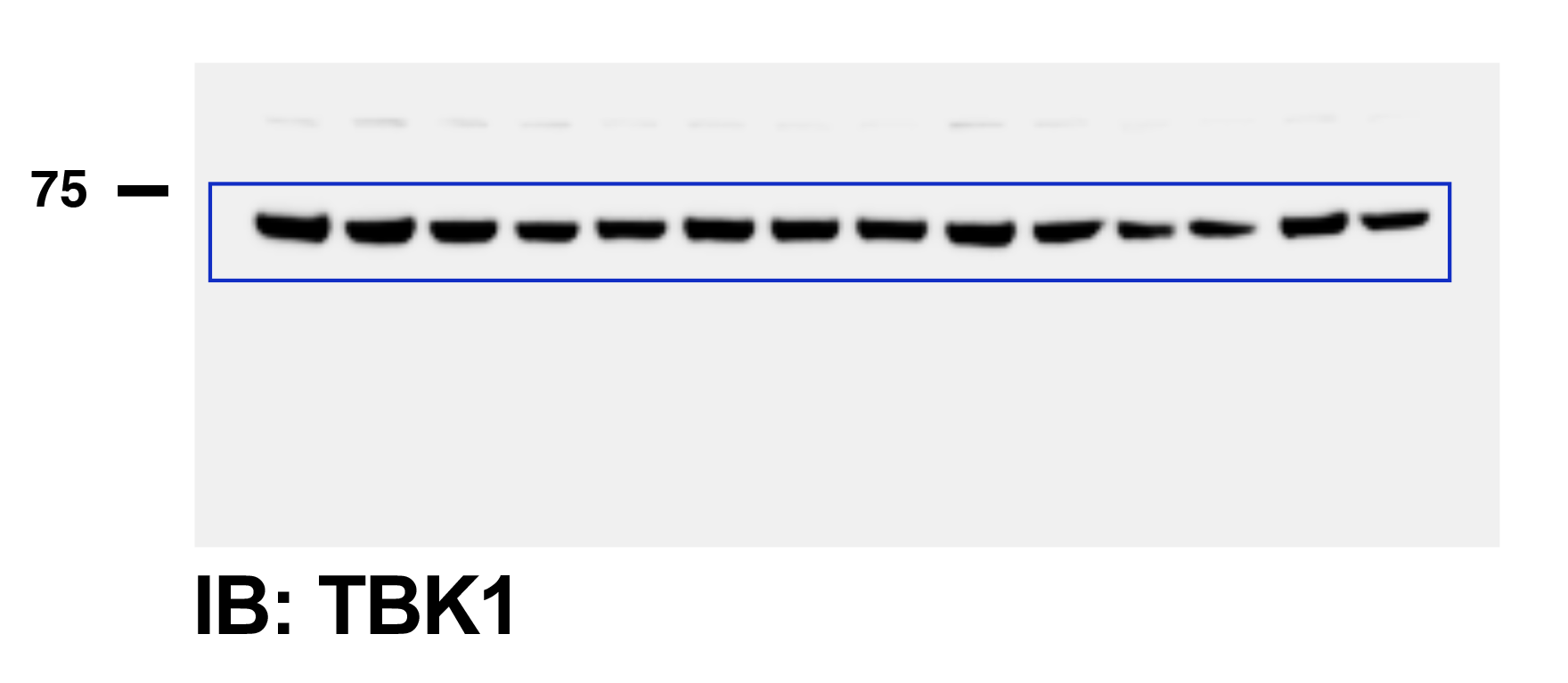

Supplement: Supplementary file 8 — Figure EV1-5 Source Data [file 44319_2024_354_MOESM8_ESM.zip › Figure EV1-5/Figure EV3/EV3D/TBK1.tif]

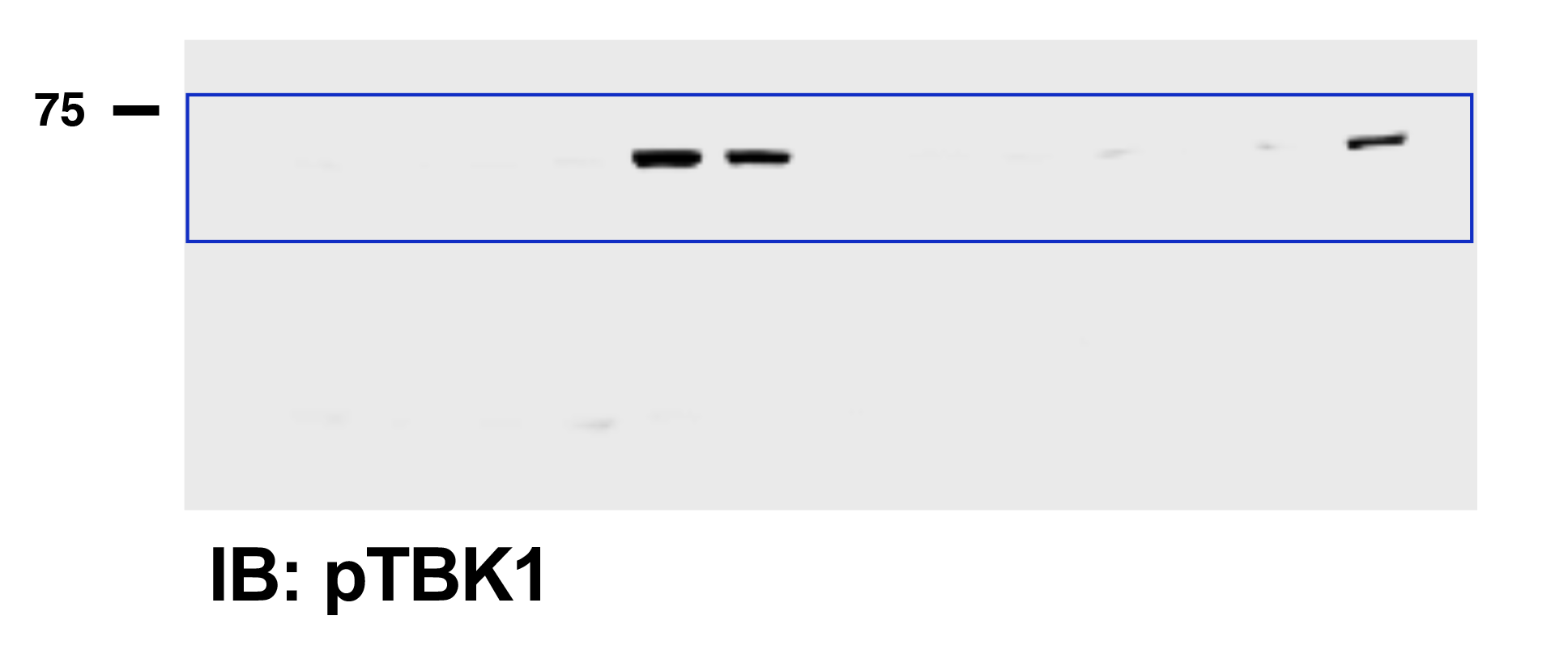

Supplement: Supplementary file 8 — Figure EV1-5 Source Data [file 44319_2024_354_MOESM8_ESM.zip › Figure EV1-5/Figure EV3/EV3D/pTBK1.tif]

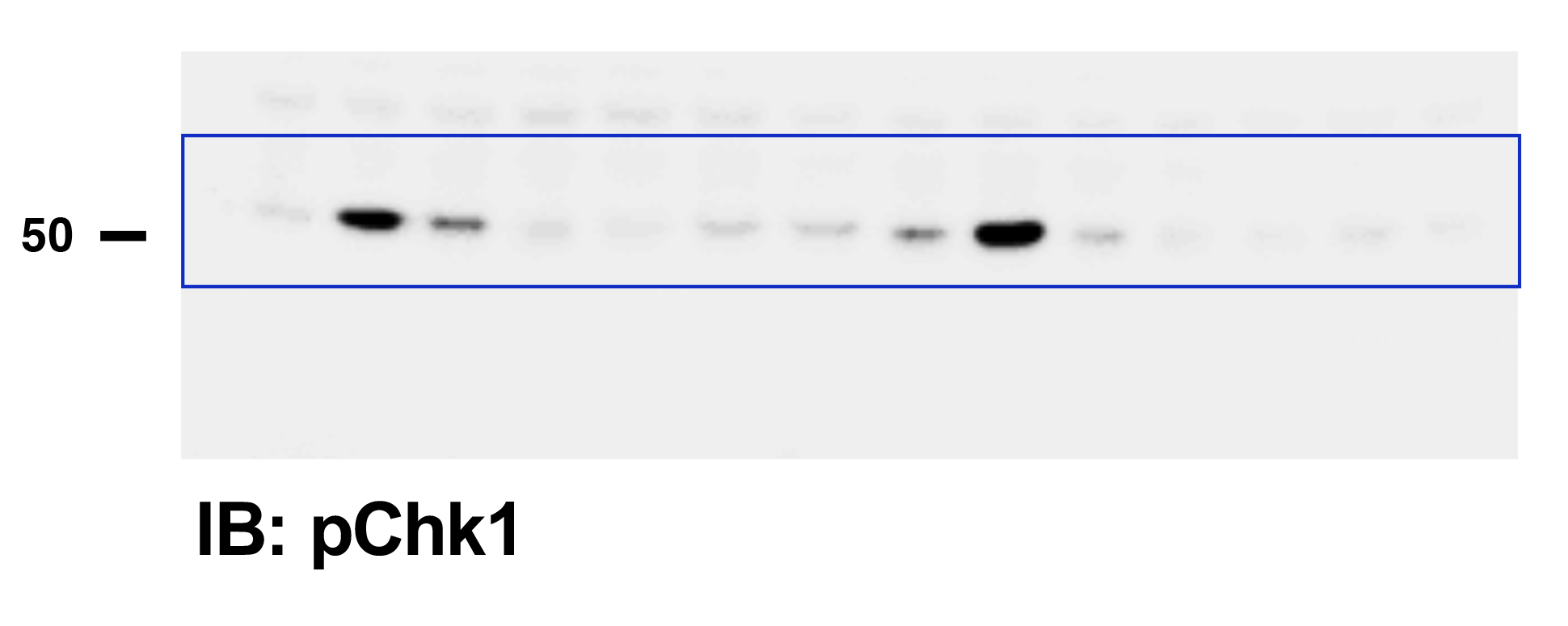

Supplement: Supplementary file 8 — Figure EV1-5 Source Data [file 44319_2024_354_MOESM8_ESM.zip › Figure EV1-5/Figure EV3/EV3D/pChk1.tif]

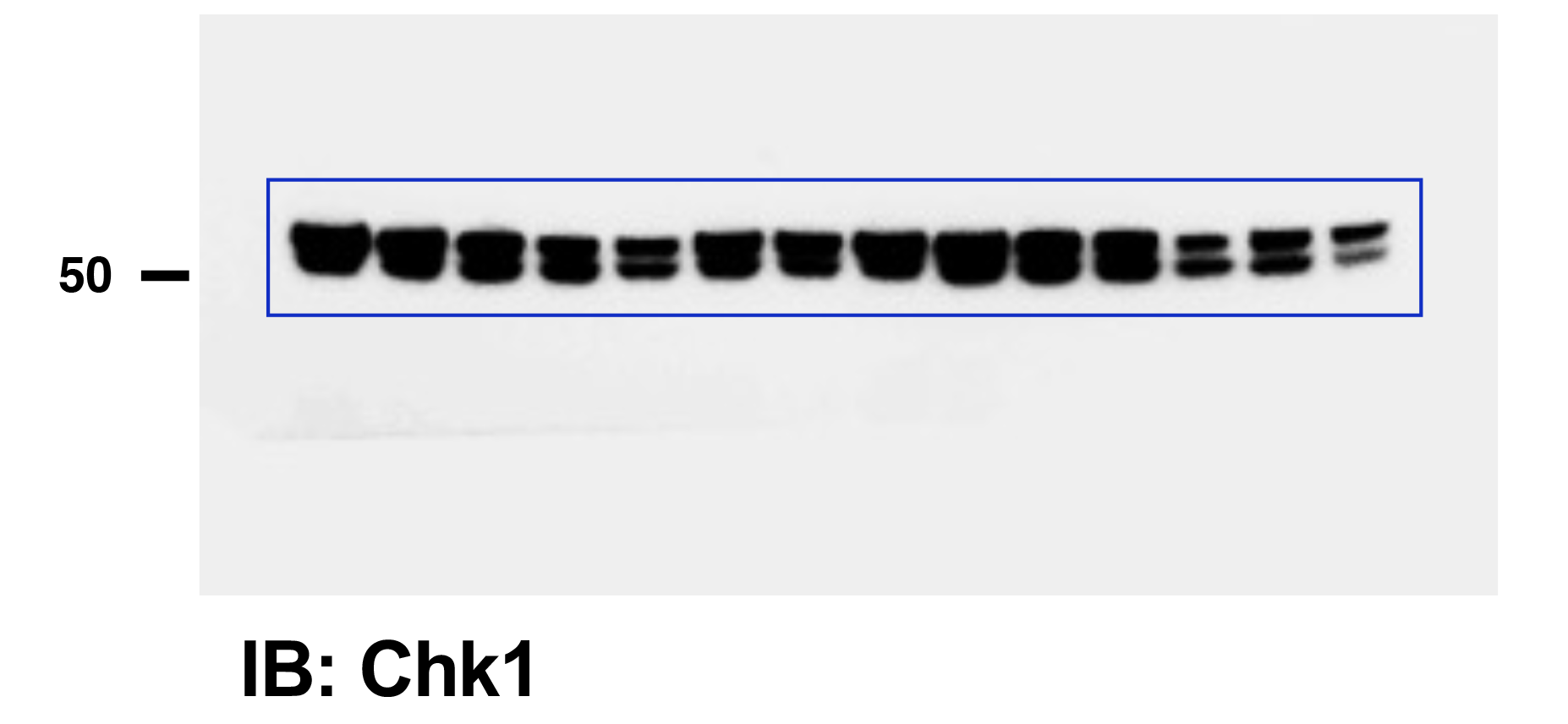

Supplement: Supplementary file 8 — Figure EV1-5 Source Data [file 44319_2024_354_MOESM8_ESM.zip › Figure EV1-5/Figure EV3/EV3D/Chk1.tif]

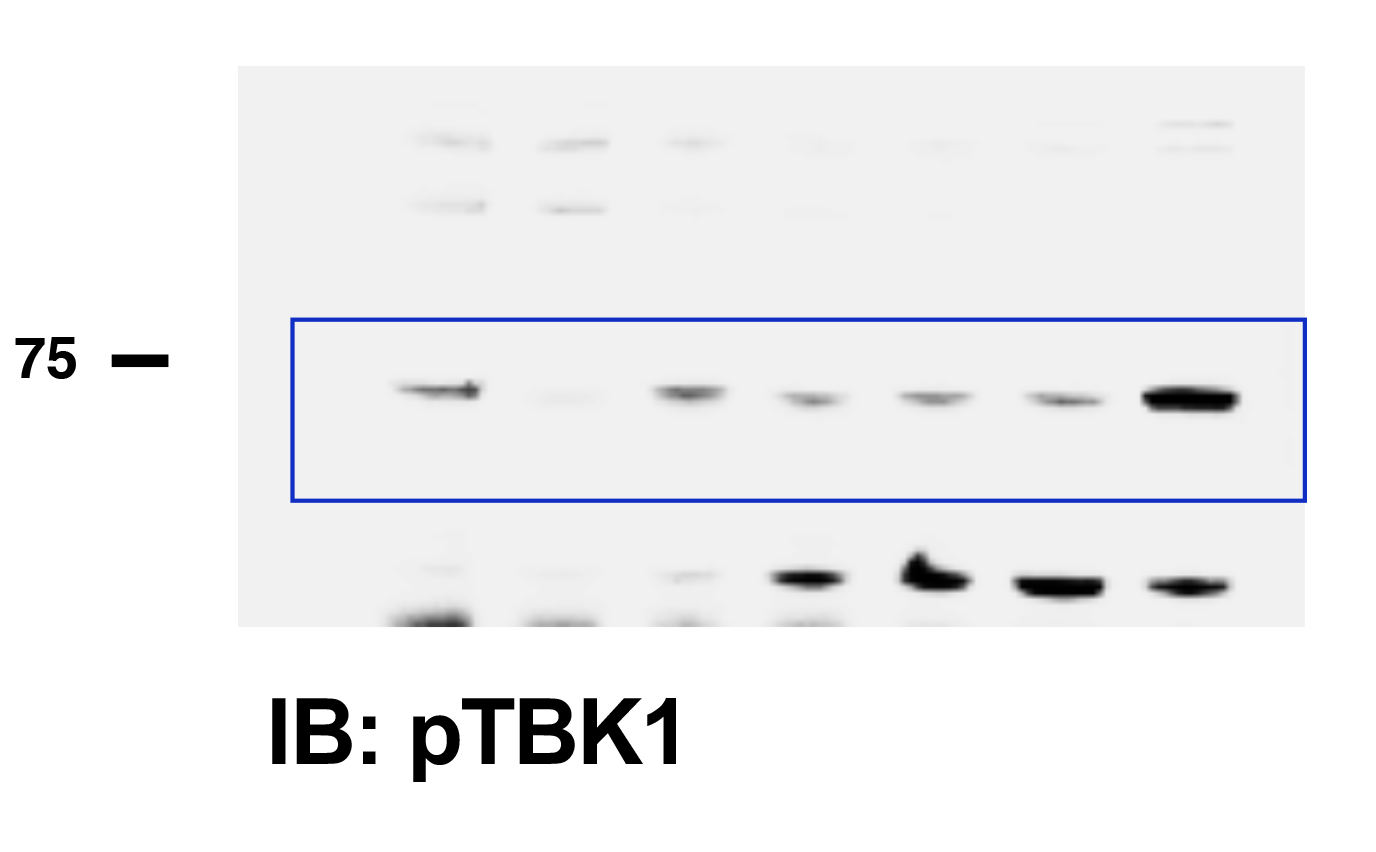

Supplement: Supplementary file 8 — Figure EV1-5 Source Data [file 44319_2024_354_MOESM8_ESM.zip › Figure EV1-5/Figure EV3/EV3C/pTBK1_addback.tif]

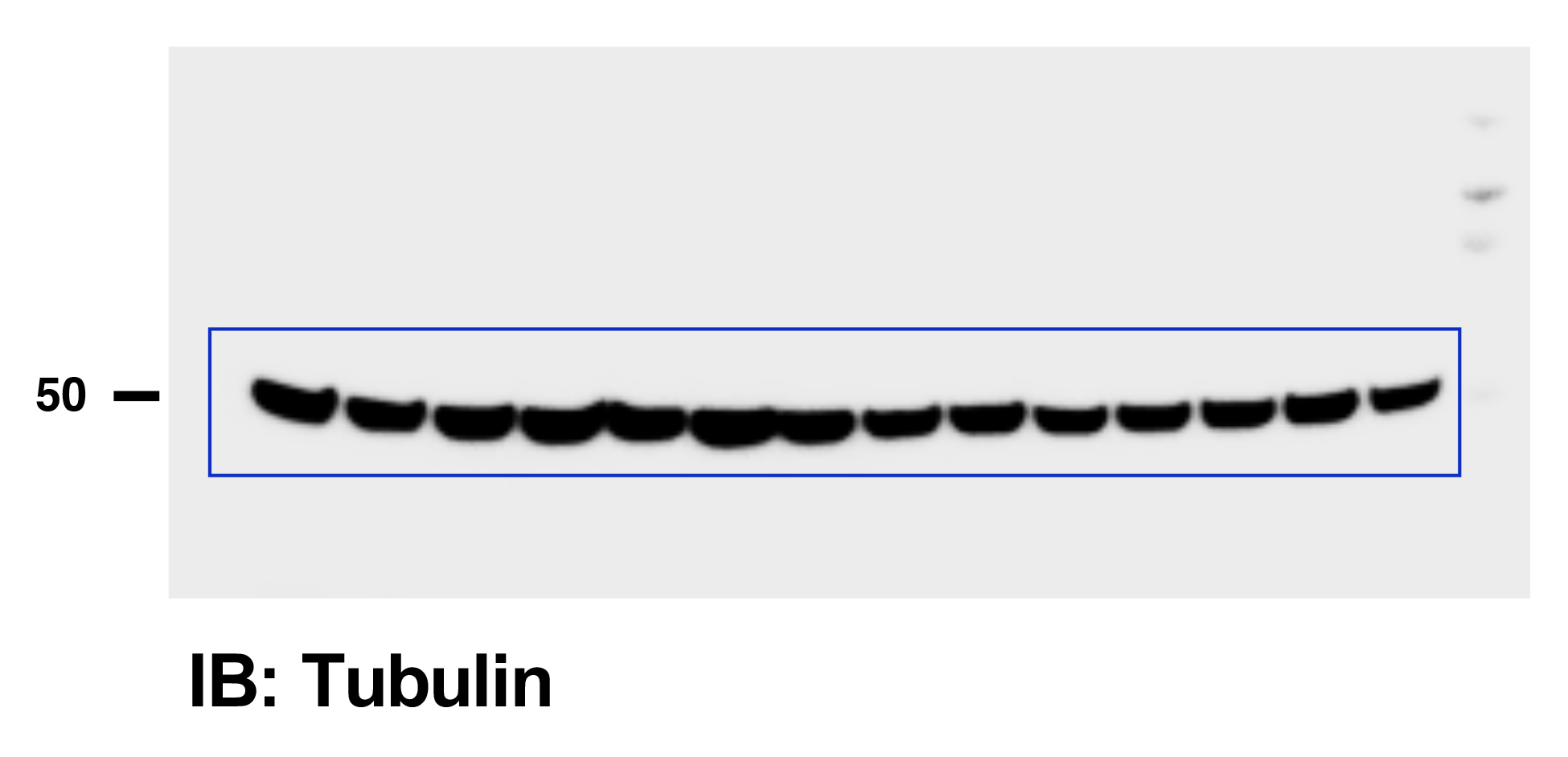

Supplement: Supplementary file 8 — Figure EV1-5 Source Data [file 44319_2024_354_MOESM8_ESM.zip › Figure EV1-5/Figure EV3/EV3C/Tubulin.tif]

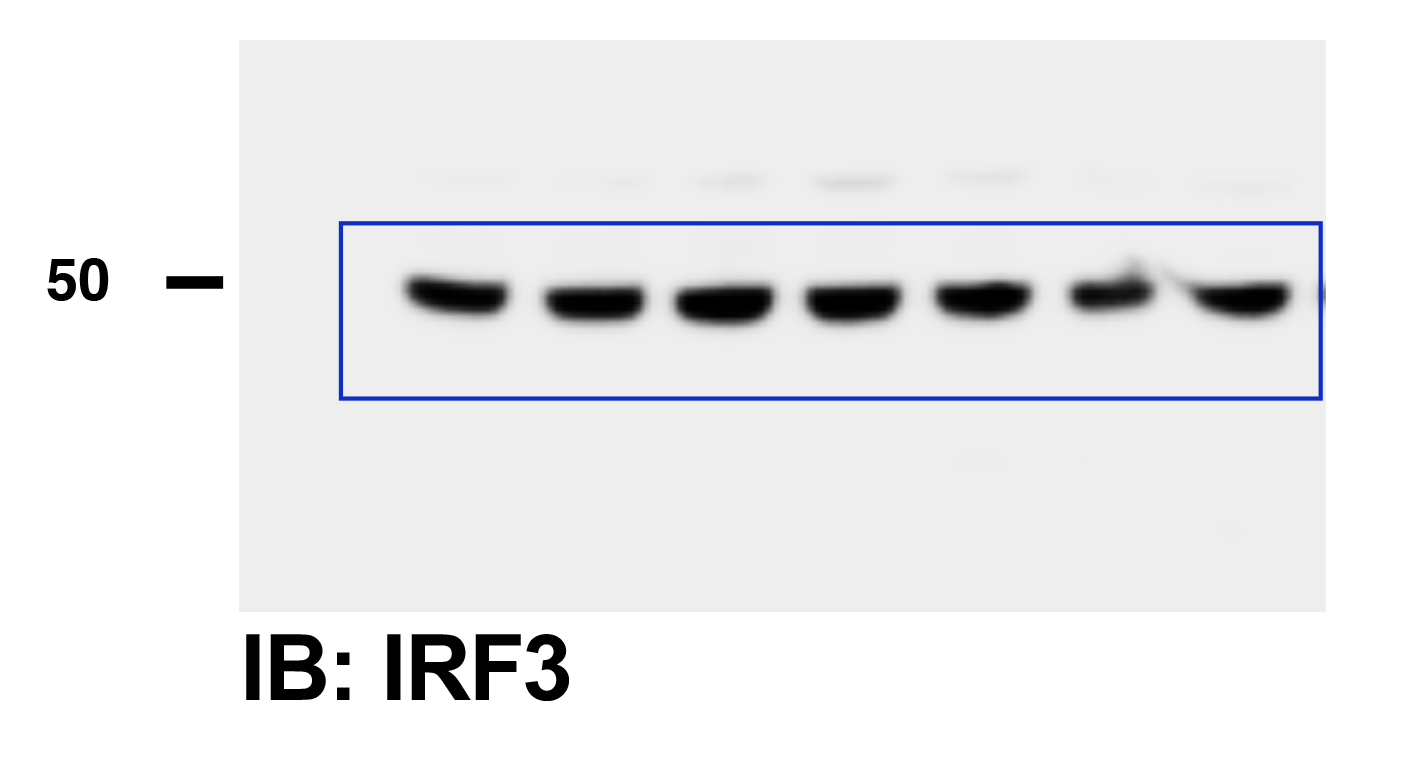

Supplement: Supplementary file 8 — Figure EV1-5 Source Data [file 44319_2024_354_MOESM8_ESM.zip › Figure EV1-5/Figure EV3/EV3C/IRF3_addback.tif]

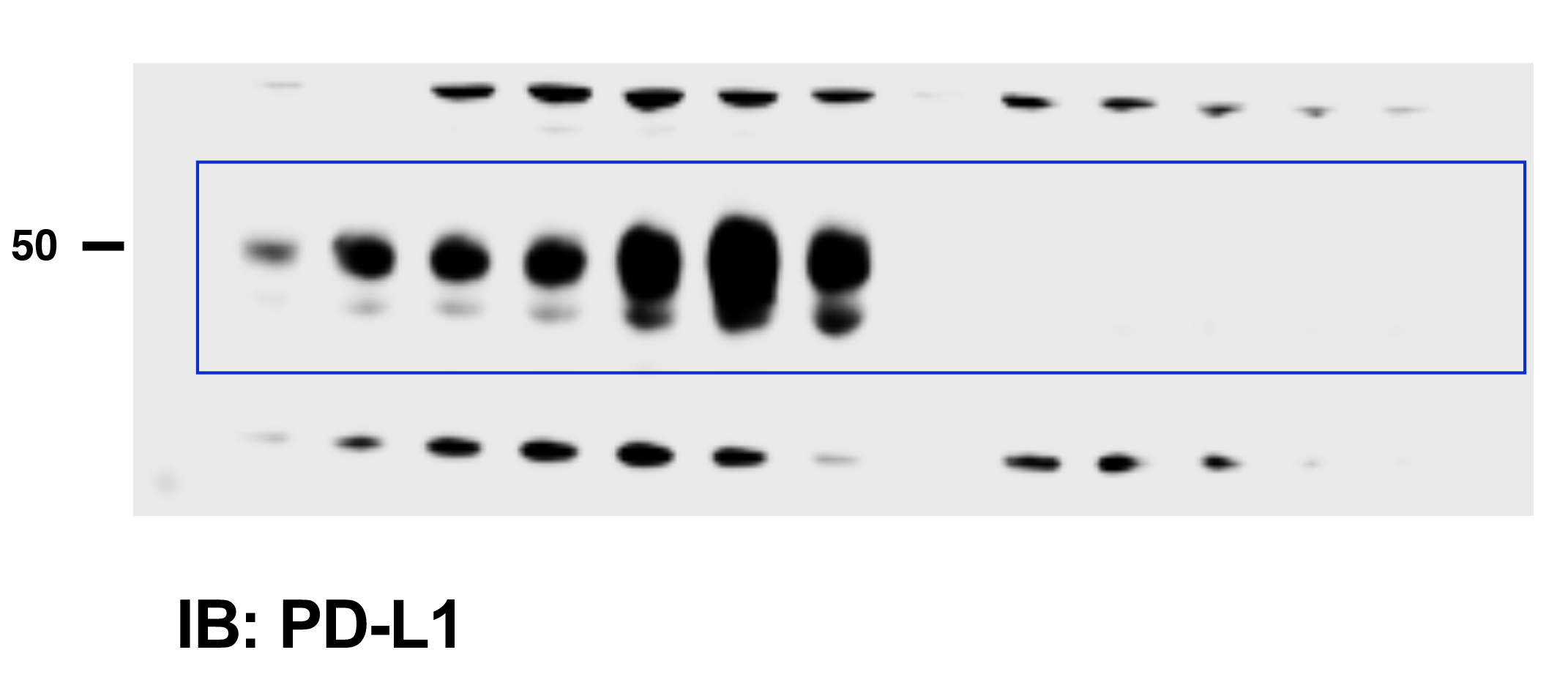

Supplement: Supplementary file 8 — Figure EV1-5 Source Data [file 44319_2024_354_MOESM8_ESM.zip › Figure EV1-5/Figure EV3/EV3C/PD-L1.tif]

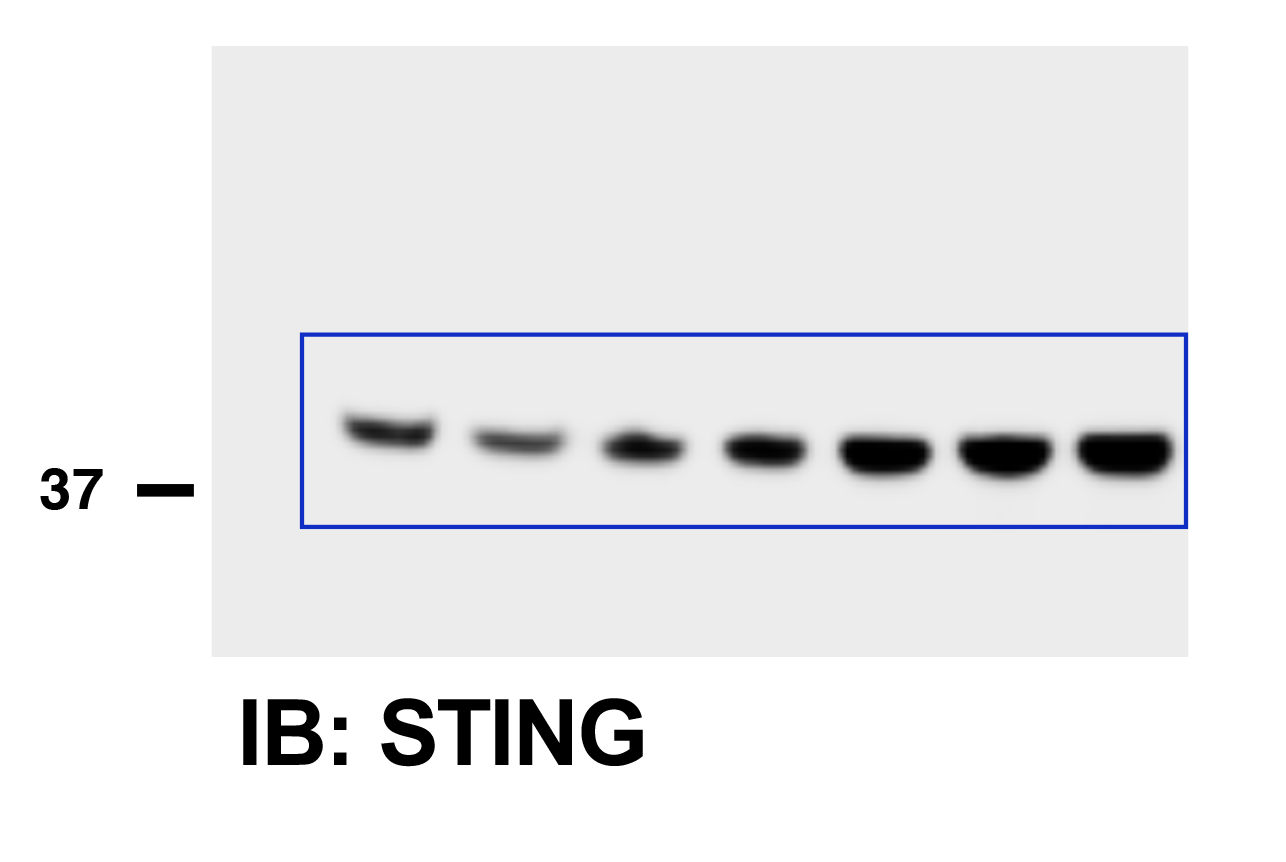

Supplement: Supplementary file 8 — Figure EV1-5 Source Data [file 44319_2024_354_MOESM8_ESM.zip › Figure EV1-5/Figure EV3/EV3C/STING_addback.tif]

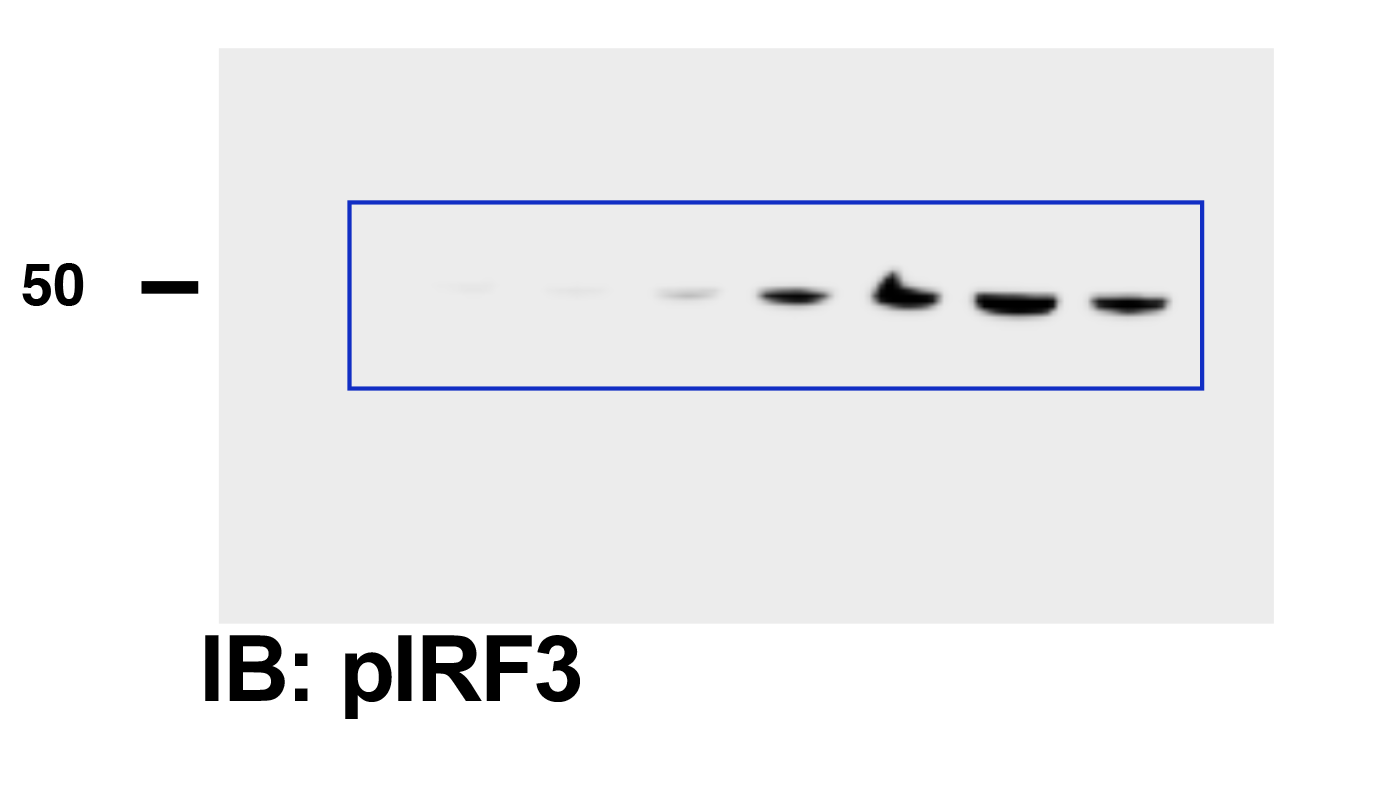

Supplement: Supplementary file 8 — Figure EV1-5 Source Data [file 44319_2024_354_MOESM8_ESM.zip › Figure EV1-5/Figure EV3/EV3C/pIRF3_addback.tif]

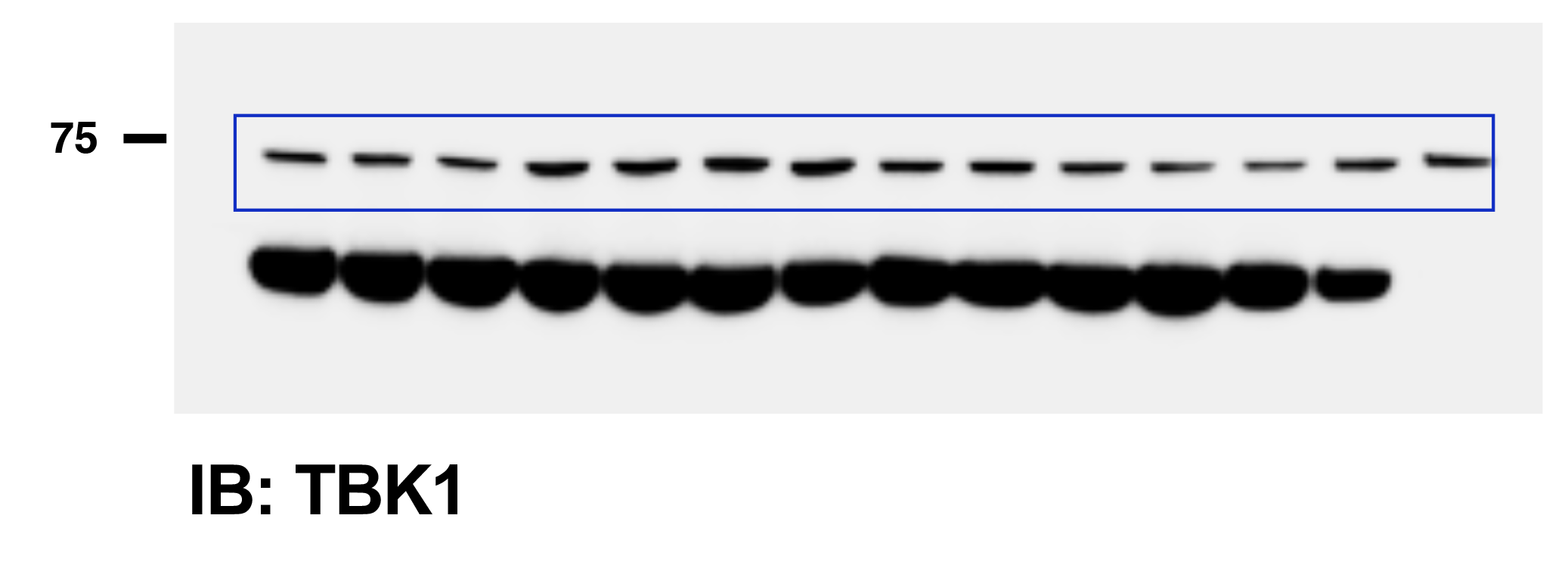

Supplement: Supplementary file 8 — Figure EV1-5 Source Data [file 44319_2024_354_MOESM8_ESM.zip › Figure EV1-5/Figure EV3/EV3C/TBK1.tif]

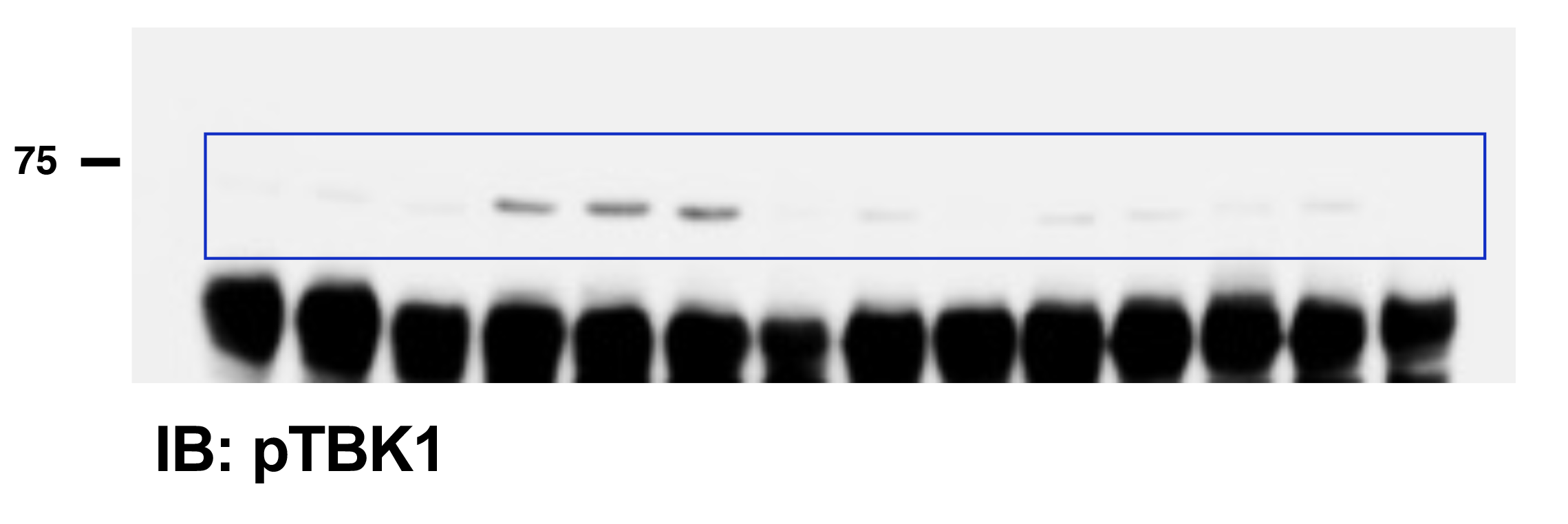

Supplement: Supplementary file 8 — Figure EV1-5 Source Data [file 44319_2024_354_MOESM8_ESM.zip › Figure EV1-5/Figure EV3/EV3C/pTBK1.tif]

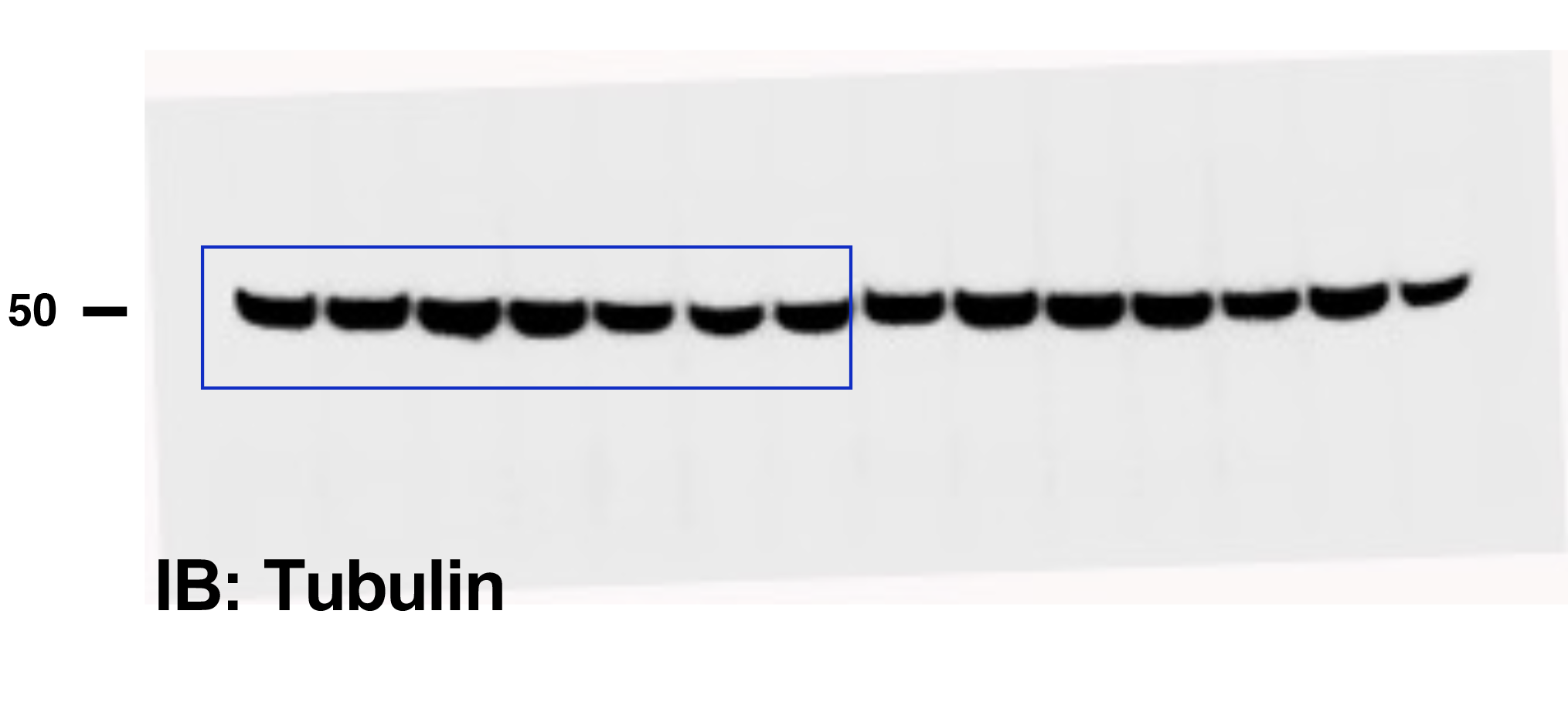

Supplement: Supplementary file 8 — Figure EV1-5 Source Data [file 44319_2024_354_MOESM8_ESM.zip › Figure EV1-5/Figure EV3/EV3C/Tubulin_addback.tif]

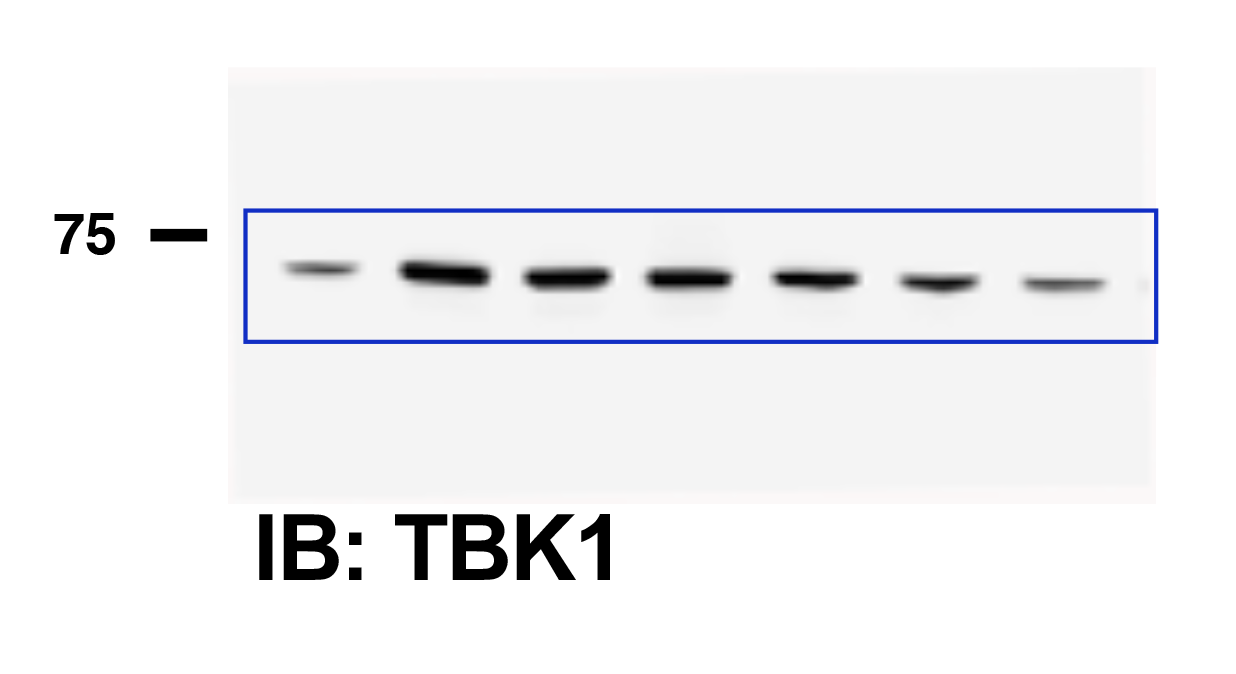

Supplement: Supplementary file 8 — Figure EV1-5 Source Data [file 44319_2024_354_MOESM8_ESM.zip › Figure EV1-5/Figure EV3/EV3C/TBK1_addback.tif]

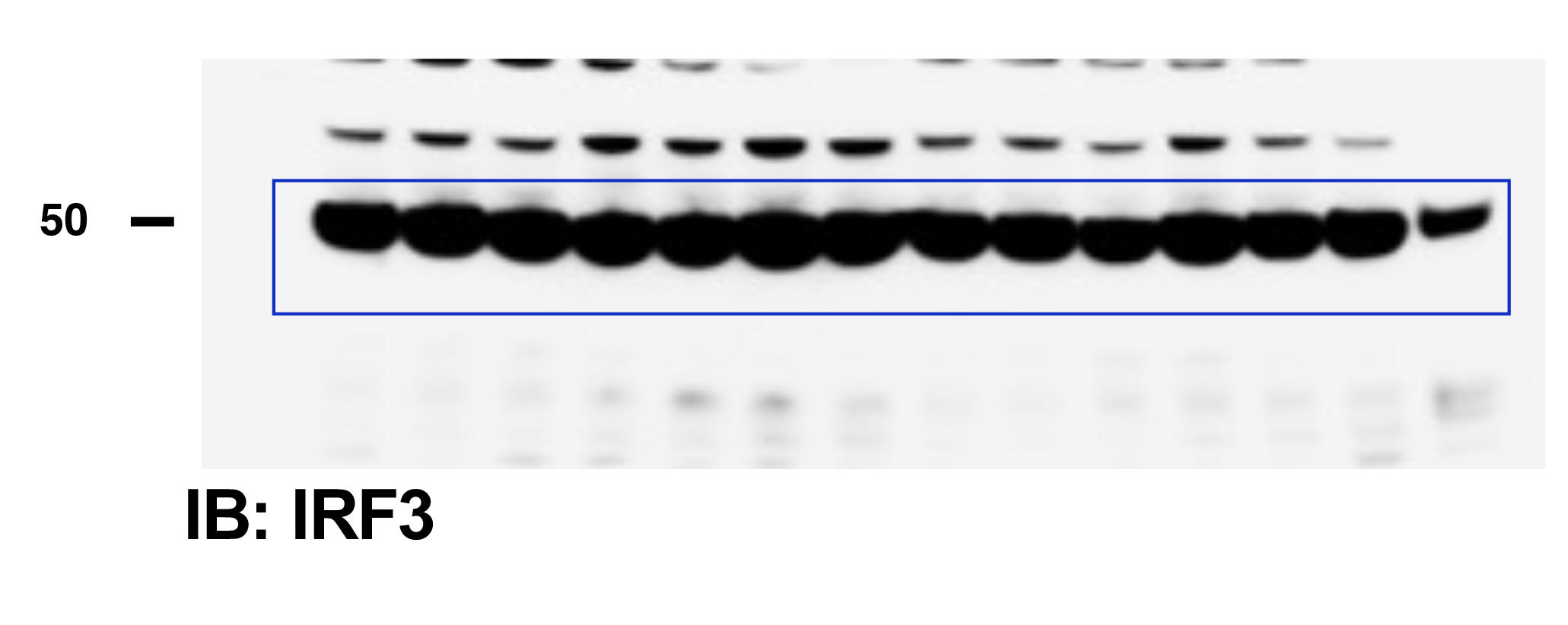

Supplement: Supplementary file 8 — Figure EV1-5 Source Data [file 44319_2024_354_MOESM8_ESM.zip › Figure EV1-5/Figure EV3/EV3C/IRF3.tif]

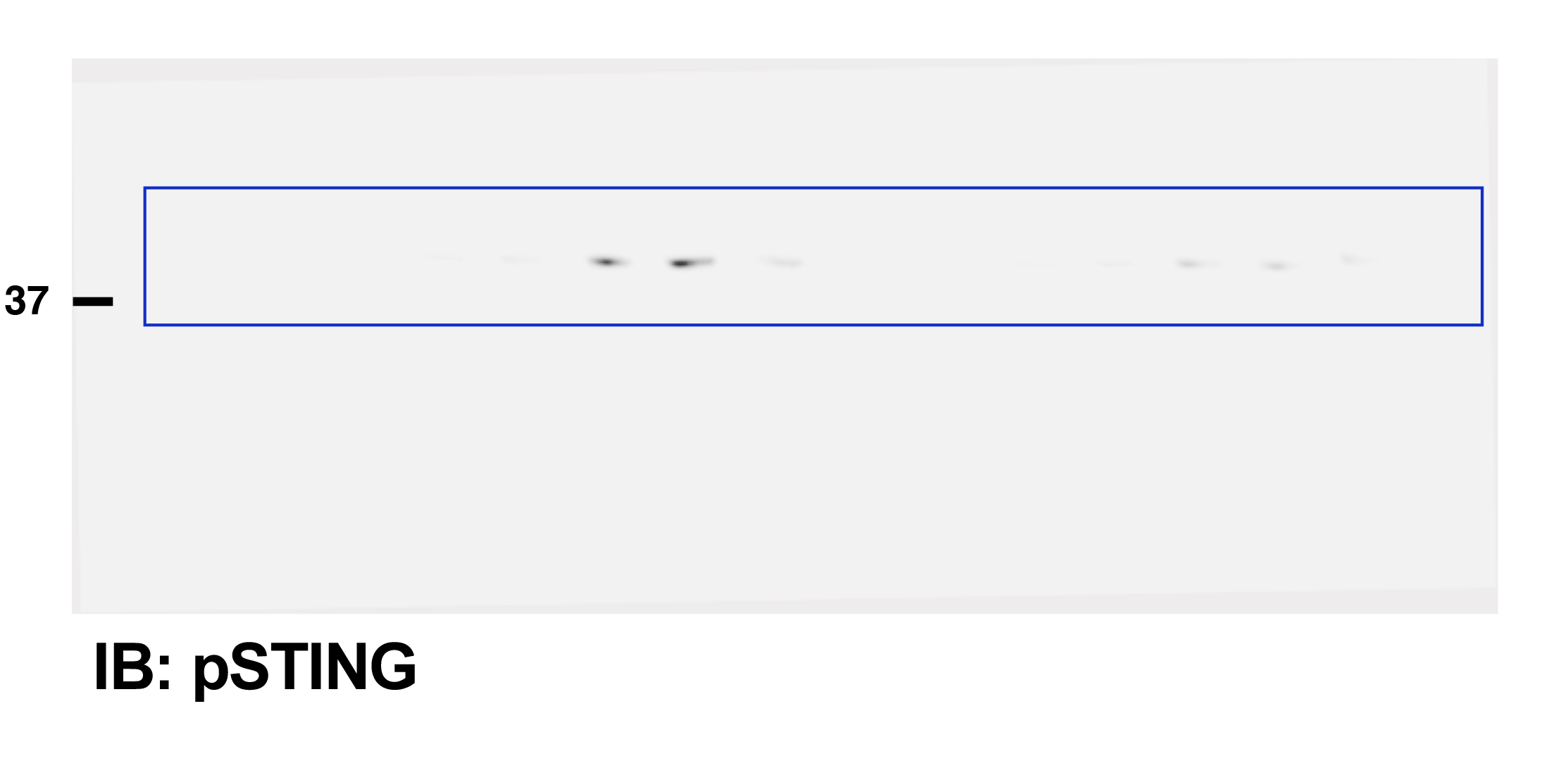

Supplement: Supplementary file 8 — Figure EV1-5 Source Data [file 44319_2024_354_MOESM8_ESM.zip › Figure EV1-5/Figure EV3/EV3C/pSTING.tif]

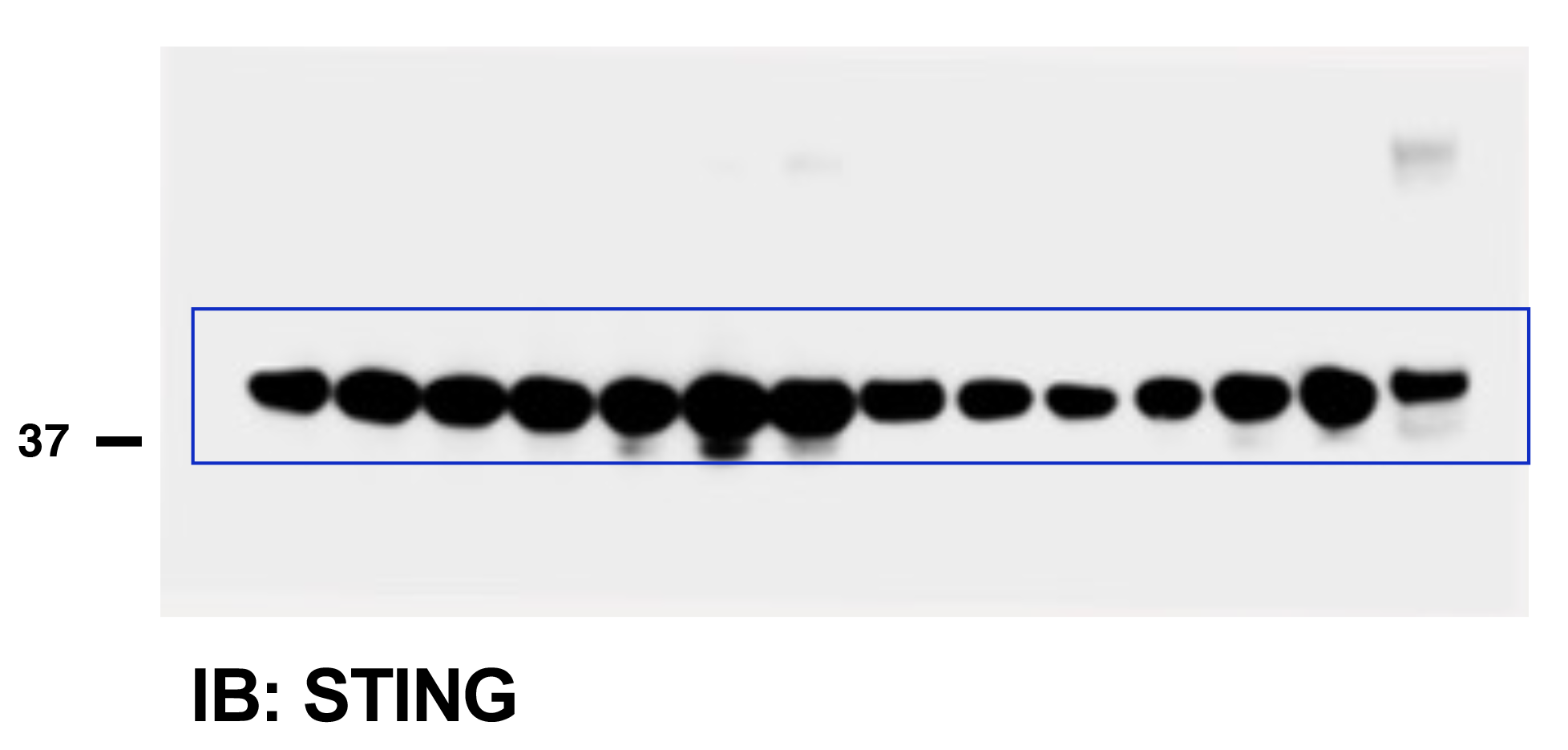

Supplement: Supplementary file 8 — Figure EV1-5 Source Data [file 44319_2024_354_MOESM8_ESM.zip › Figure EV1-5/Figure EV3/EV3C/STING.tif]

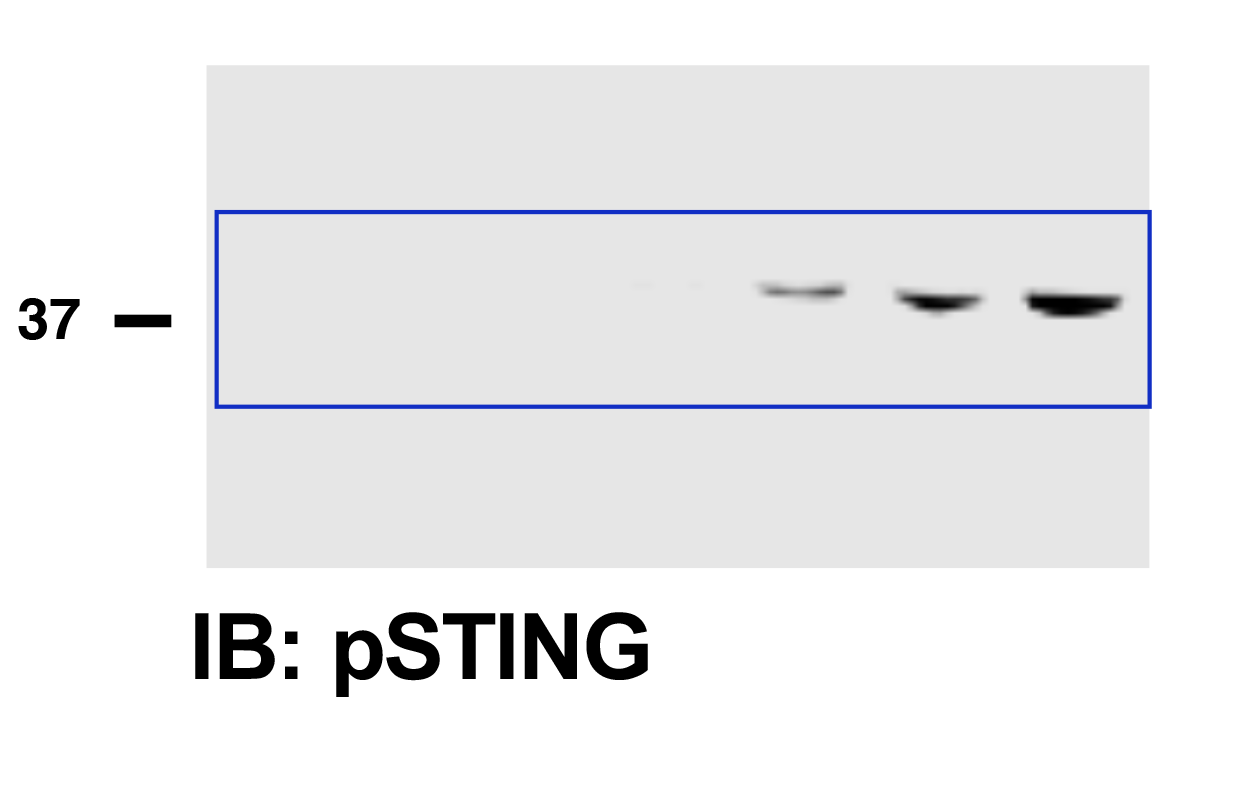

Supplement: Supplementary file 8 — Figure EV1-5 Source Data [file 44319_2024_354_MOESM8_ESM.zip › Figure EV1-5/Figure EV3/EV3C/pSTING_addback.tif]

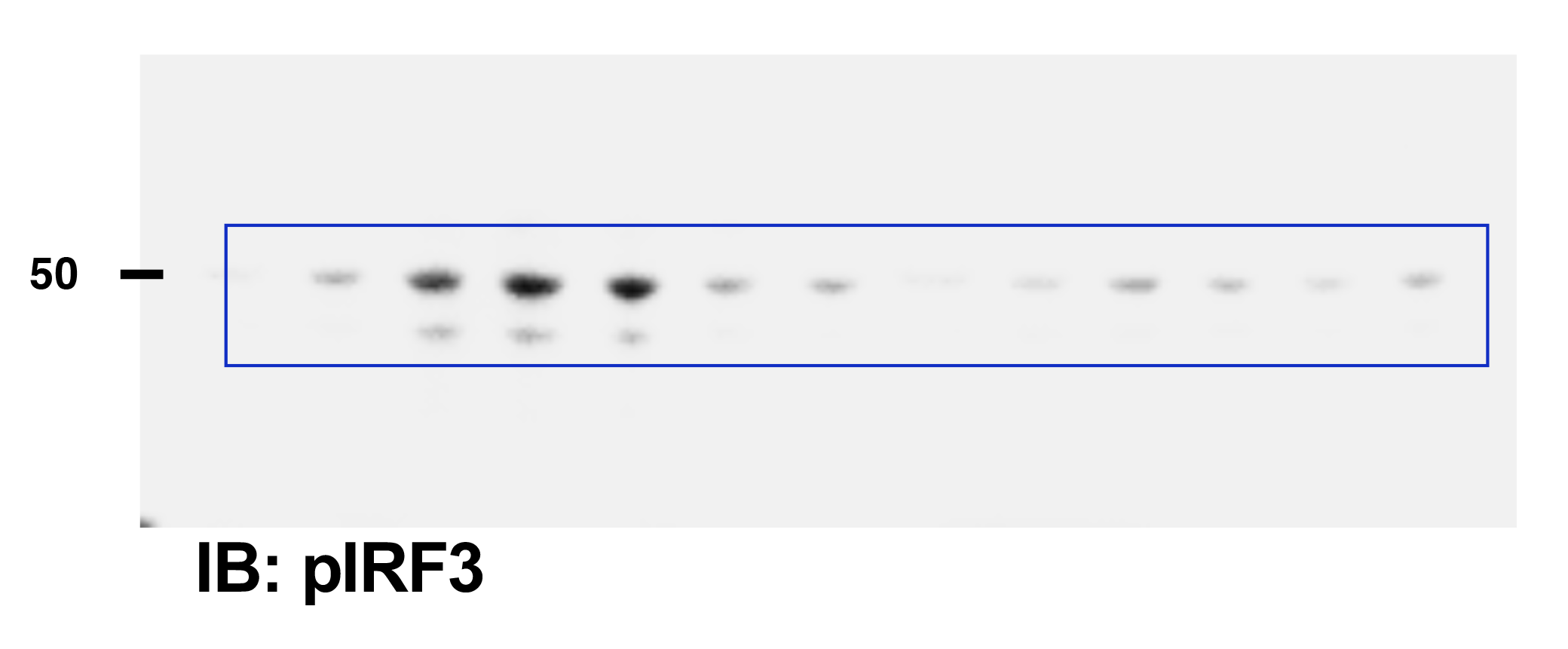

Supplement: Supplementary file 8 — Figure EV1-5 Source Data [file 44319_2024_354_MOESM8_ESM.zip › Figure EV1-5/Figure EV3/EV3C/pIRF3.tif]

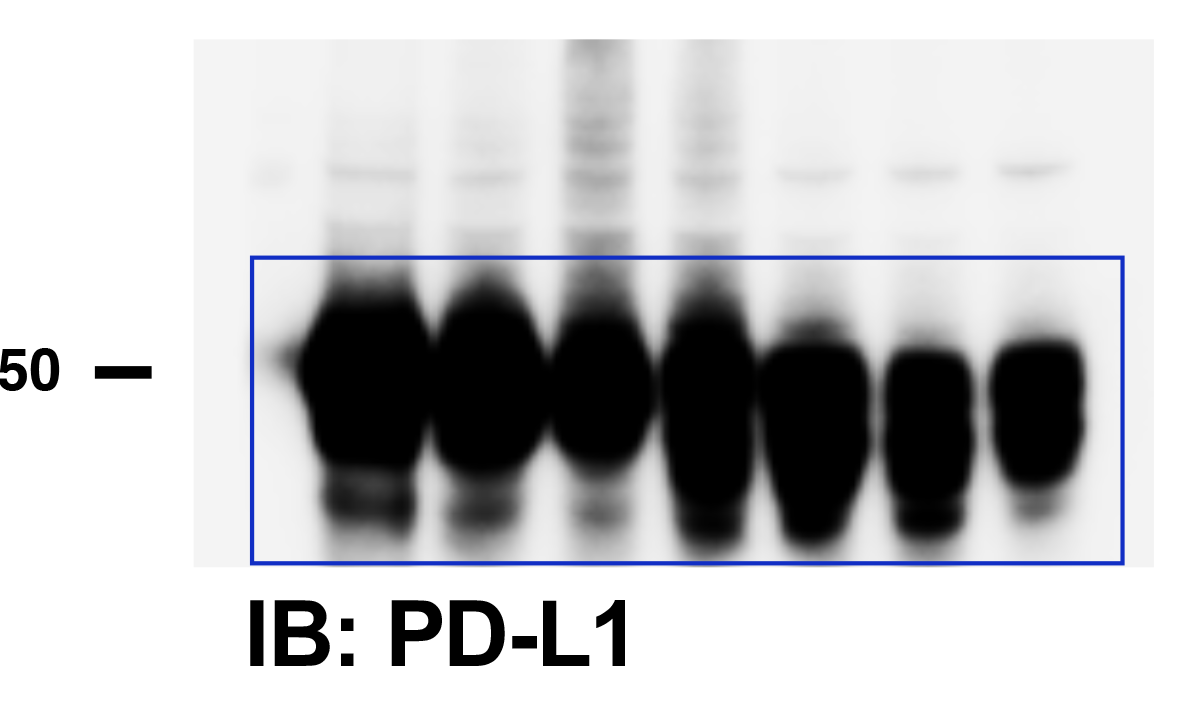

Supplement: Supplementary file 8 — Figure EV1-5 Source Data [file 44319_2024_354_MOESM8_ESM.zip › Figure EV1-5/Figure EV3/EV3C/HA-PD-L1.tif]

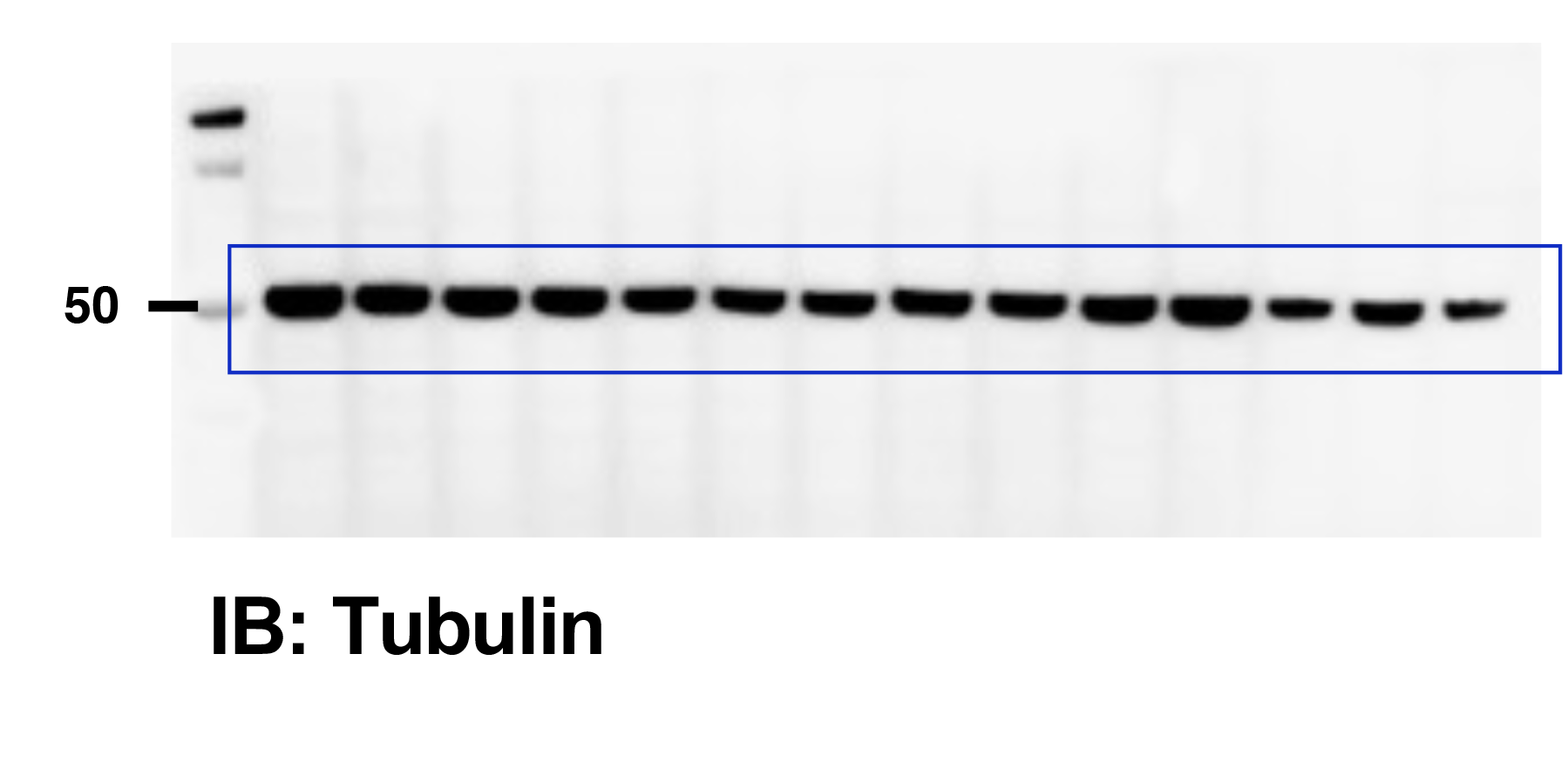

Supplement: Supplementary file 8 — Figure EV1-5 Source Data [file 44319_2024_354_MOESM8_ESM.zip › Figure EV1-5/Figure EV3/EV3B/Tubulin.tif]

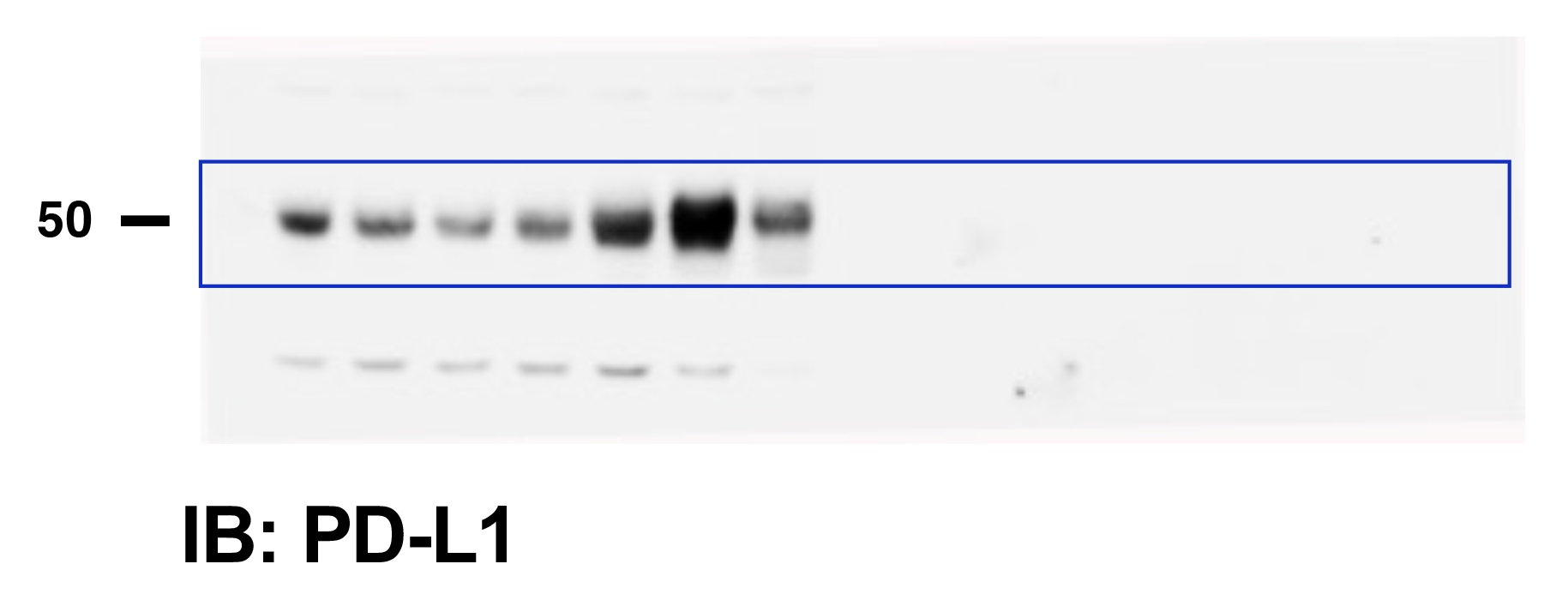

Supplement: Supplementary file 8 — Figure EV1-5 Source Data [file 44319_2024_354_MOESM8_ESM.zip › Figure EV1-5/Figure EV3/EV3B/PD-L1.tif]

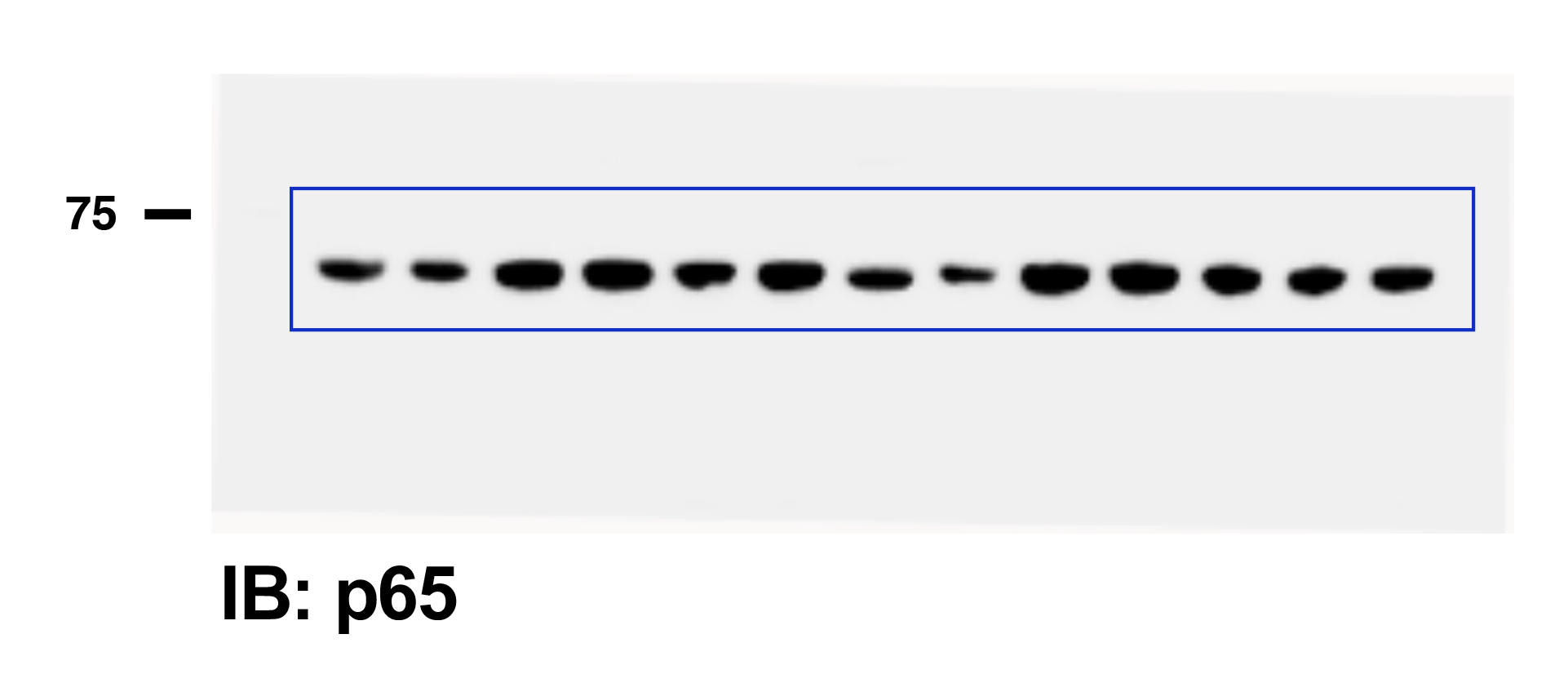

Supplement: Supplementary file 8 — Figure EV1-5 Source Data [file 44319_2024_354_MOESM8_ESM.zip › Figure EV1-5/Figure EV3/EV3B/p65.tif]

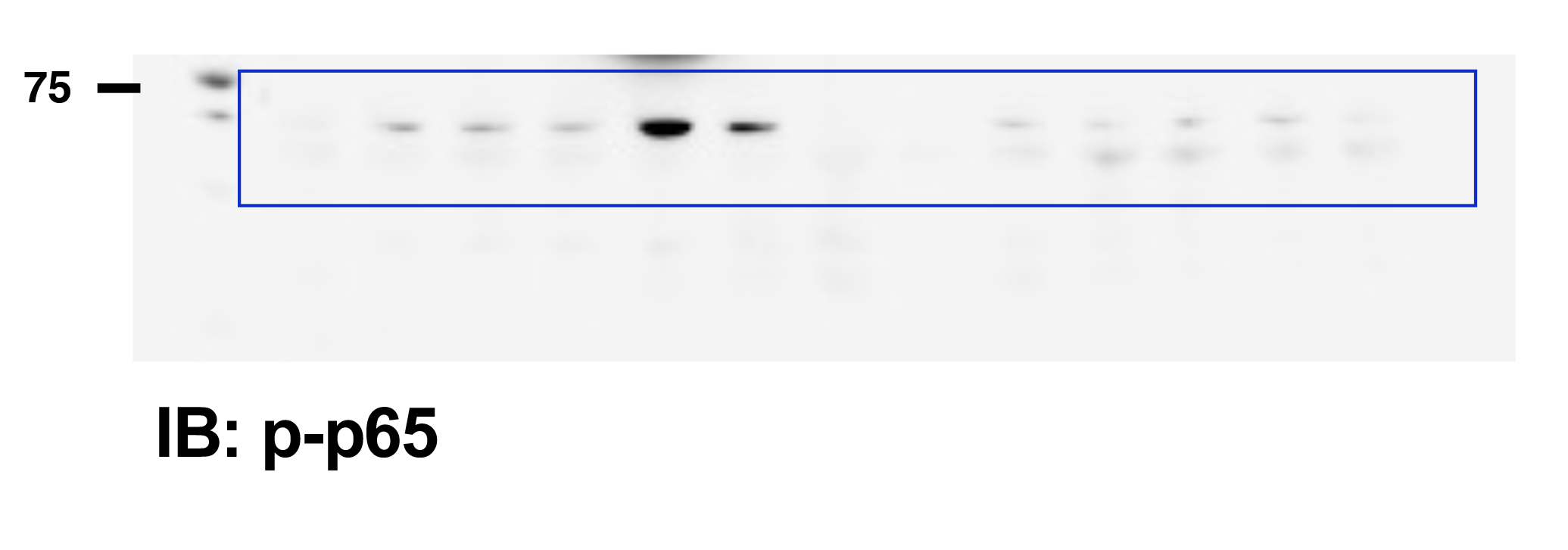

Supplement: Supplementary file 8 — Figure EV1-5 Source Data [file 44319_2024_354_MOESM8_ESM.zip › Figure EV1-5/Figure EV3/EV3B/p-p65.tif]

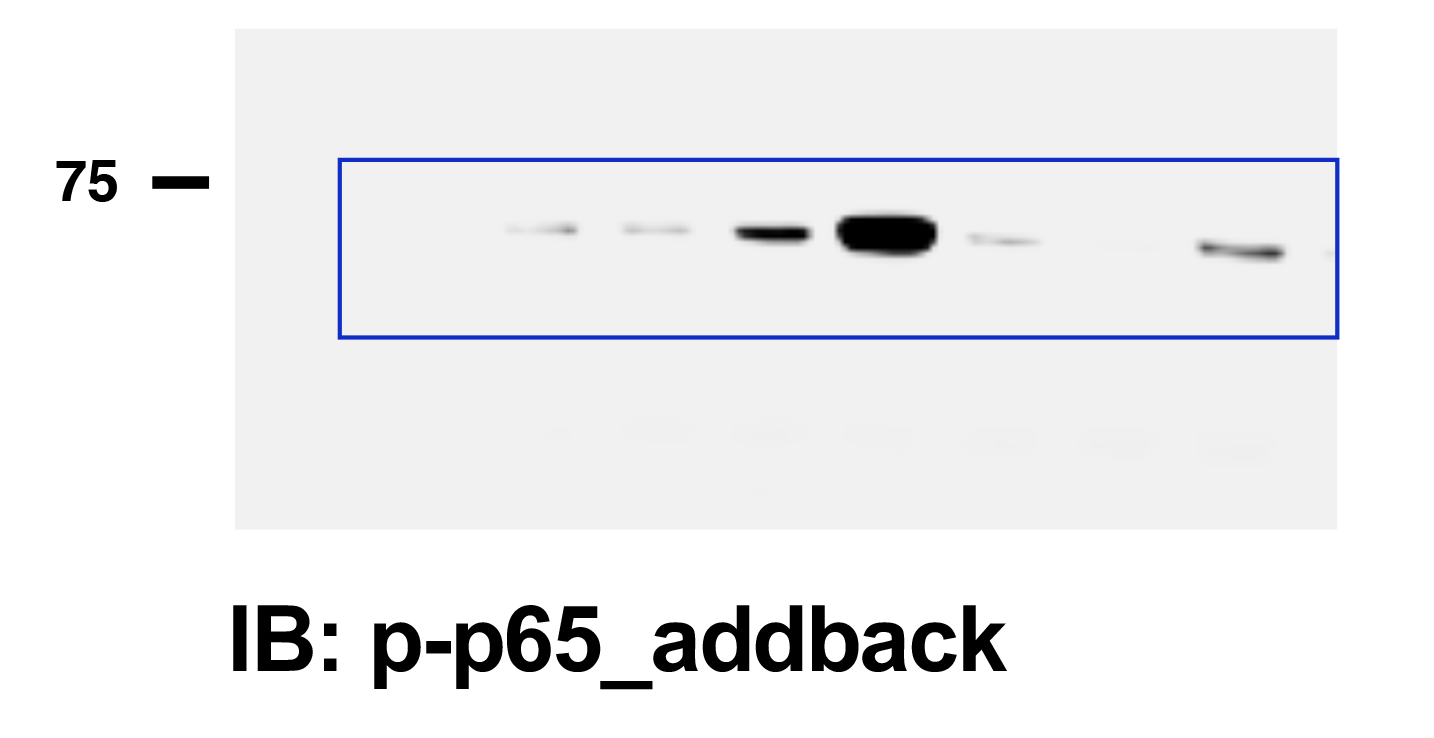

Supplement: Supplementary file 8 — Figure EV1-5 Source Data [file 44319_2024_354_MOESM8_ESM.zip › Figure EV1-5/Figure EV3/EV3B/p-p65_addback.tif]

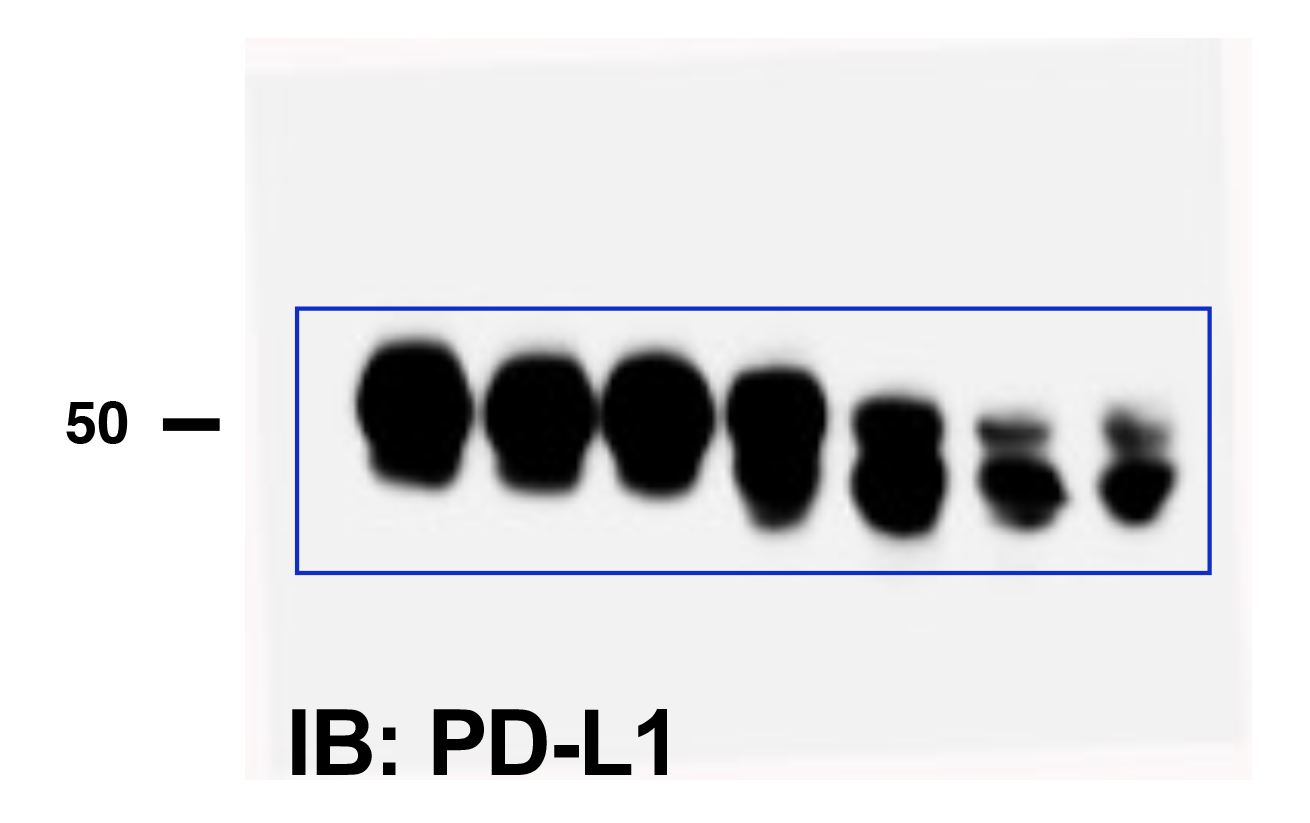

Supplement: Supplementary file 8 — Figure EV1-5 Source Data [file 44319_2024_354_MOESM8_ESM.zip › Figure EV1-5/Figure EV3/EV3B/PD-L1_addback.tif]

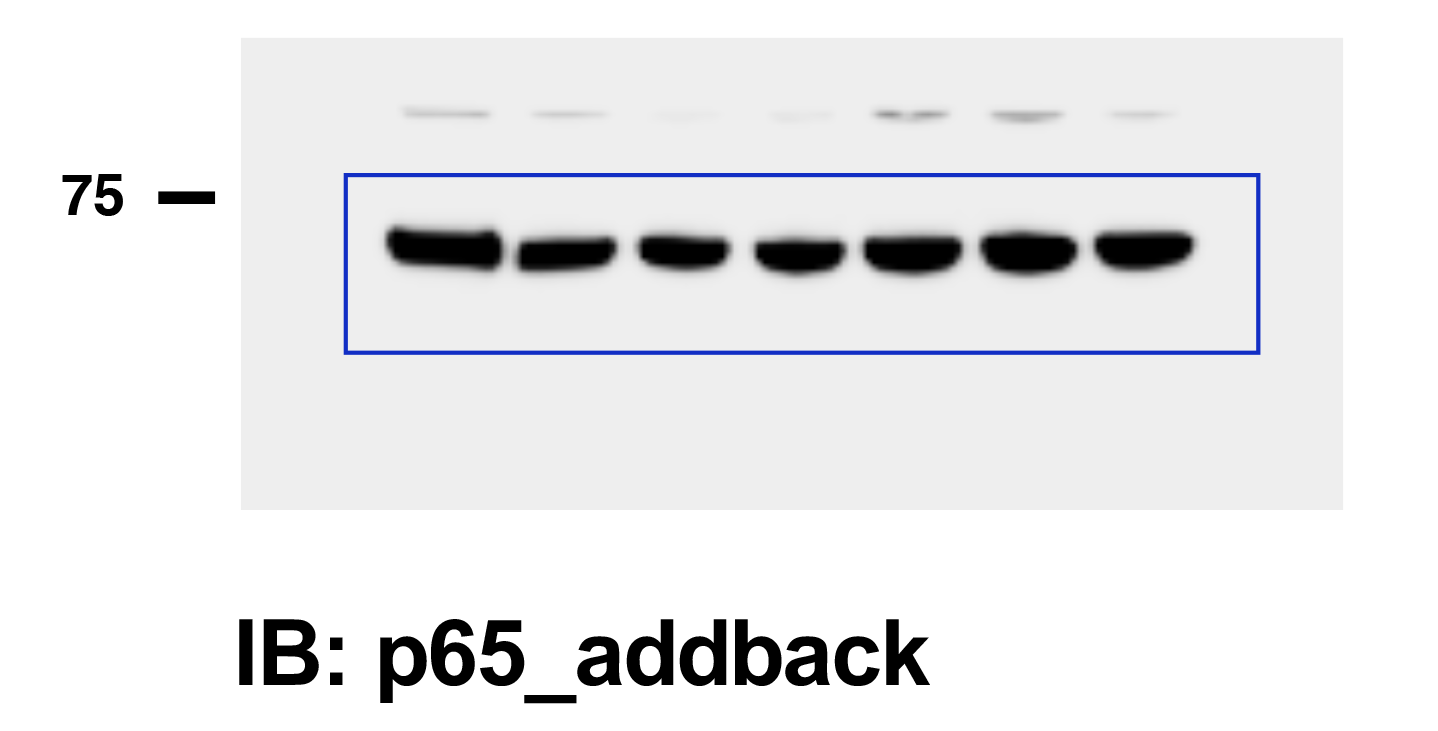

Supplement: Supplementary file 8 — Figure EV1-5 Source Data [file 44319_2024_354_MOESM8_ESM.zip › Figure EV1-5/Figure EV3/EV3B/p65_addback.tif]

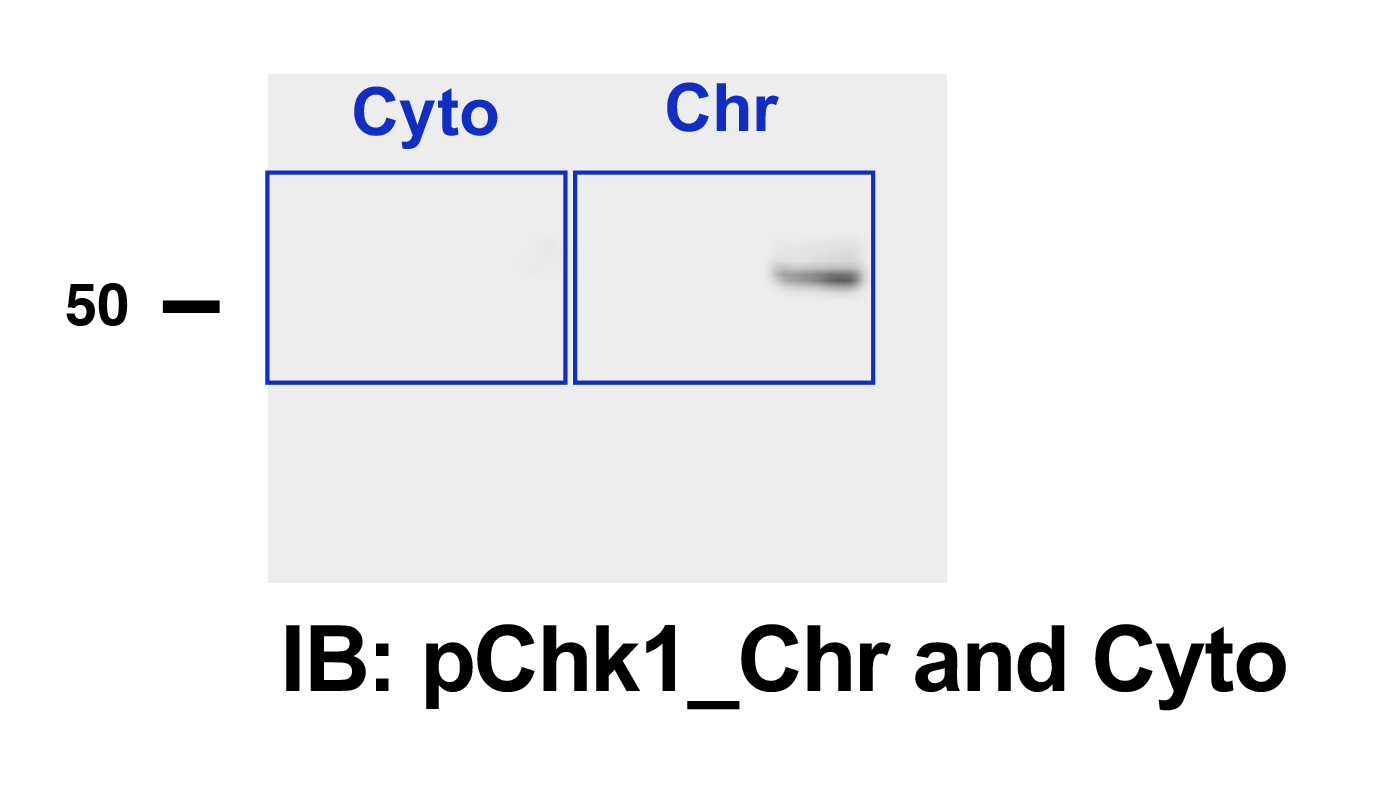

Supplement: Supplementary file 8 — Figure EV1-5 Source Data [file 44319_2024_354_MOESM8_ESM.zip › Figure EV1-5/Figure EV3/EV3E/pChk1_chromatin and cytoplasm.tif]

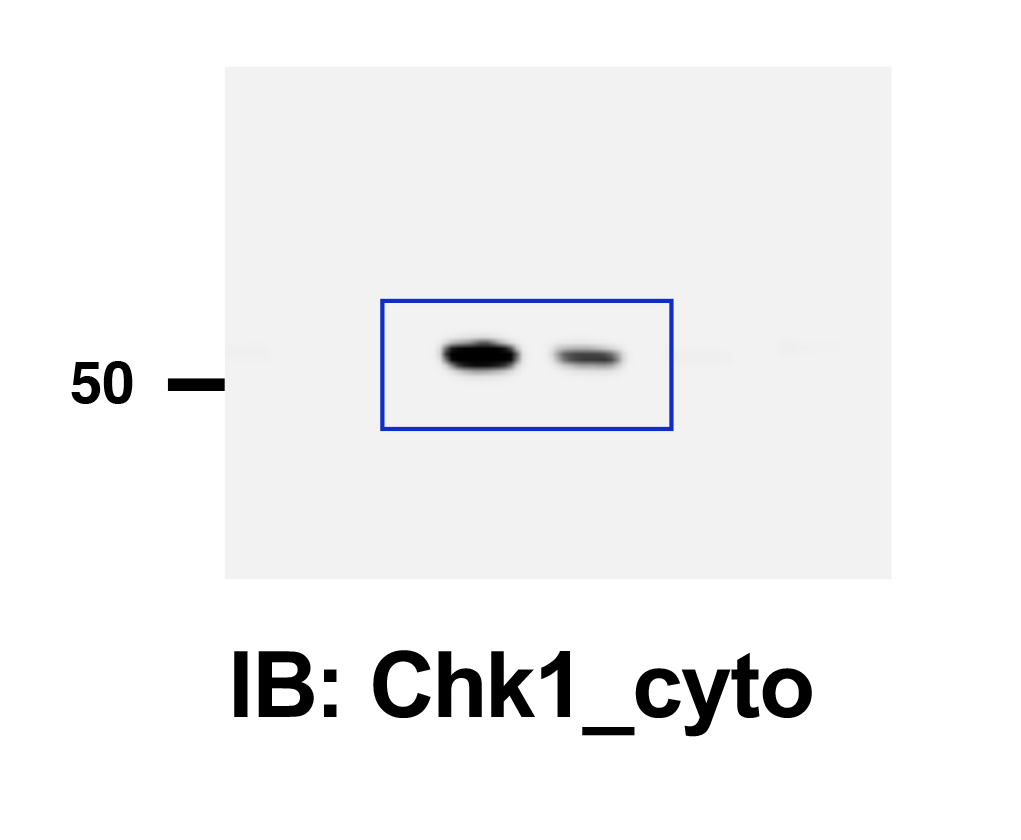

Supplement: Supplementary file 8 — Figure EV1-5 Source Data [file 44319_2024_354_MOESM8_ESM.zip › Figure EV1-5/Figure EV3/EV3E/Chk1_cytoplasm.tif]

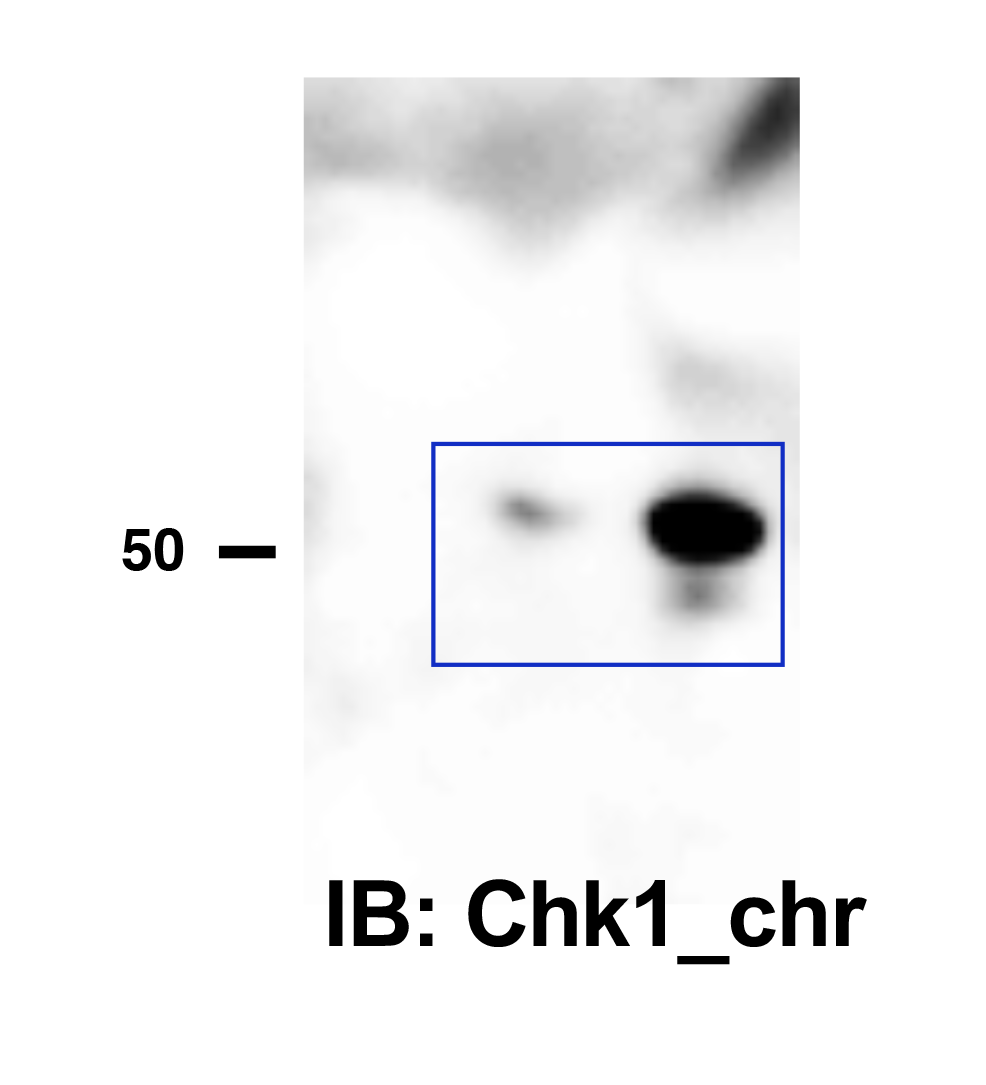

Supplement: Supplementary file 8 — Figure EV1-5 Source Data [file 44319_2024_354_MOESM8_ESM.zip › Figure EV1-5/Figure EV3/EV3E/Chk1_chromatin.tif]

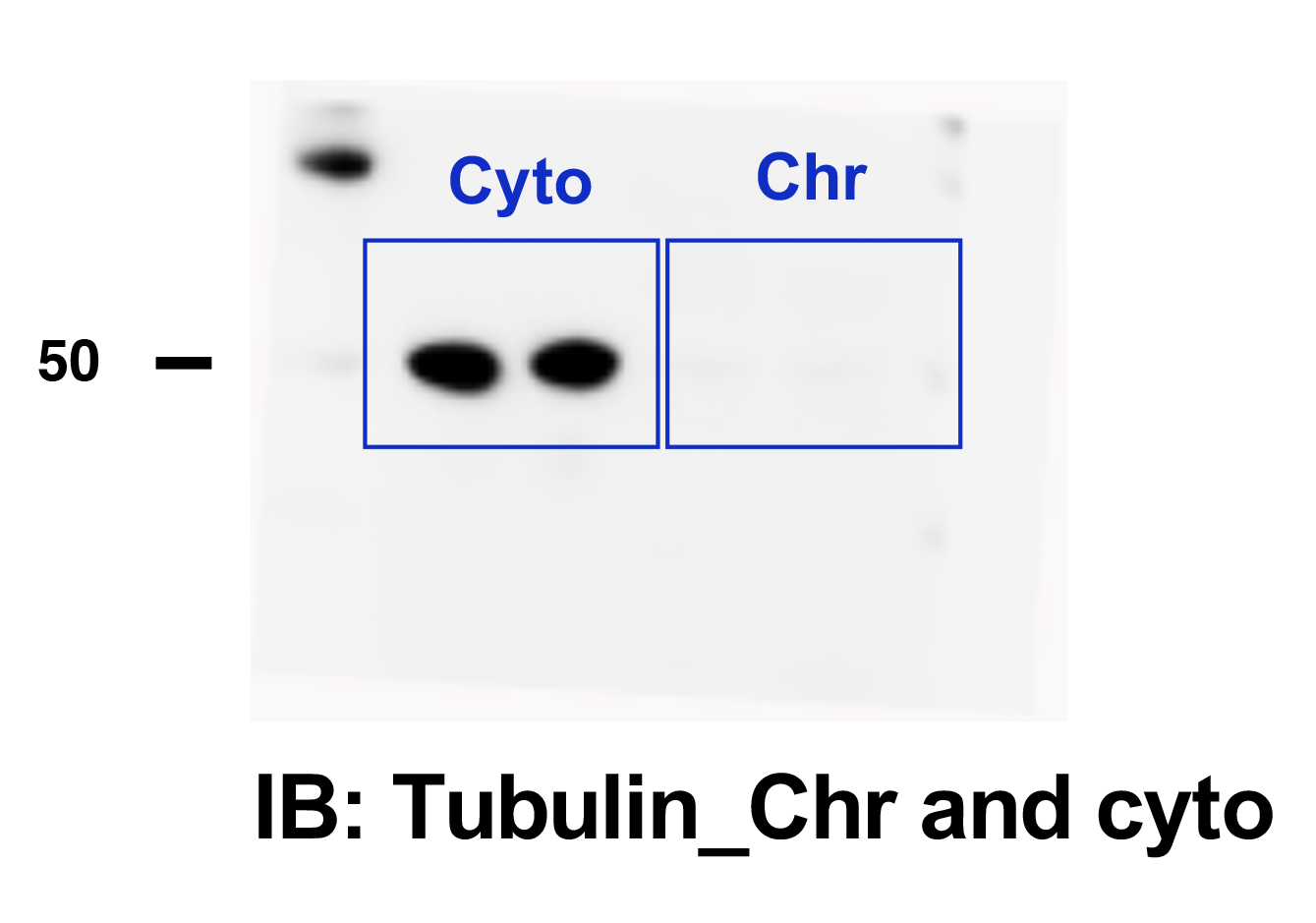

Supplement: Supplementary file 8 — Figure EV1-5 Source Data [file 44319_2024_354_MOESM8_ESM.zip › Figure EV1-5/Figure EV3/EV3E/Tubulin_chromatin and cytoplasm.tif]

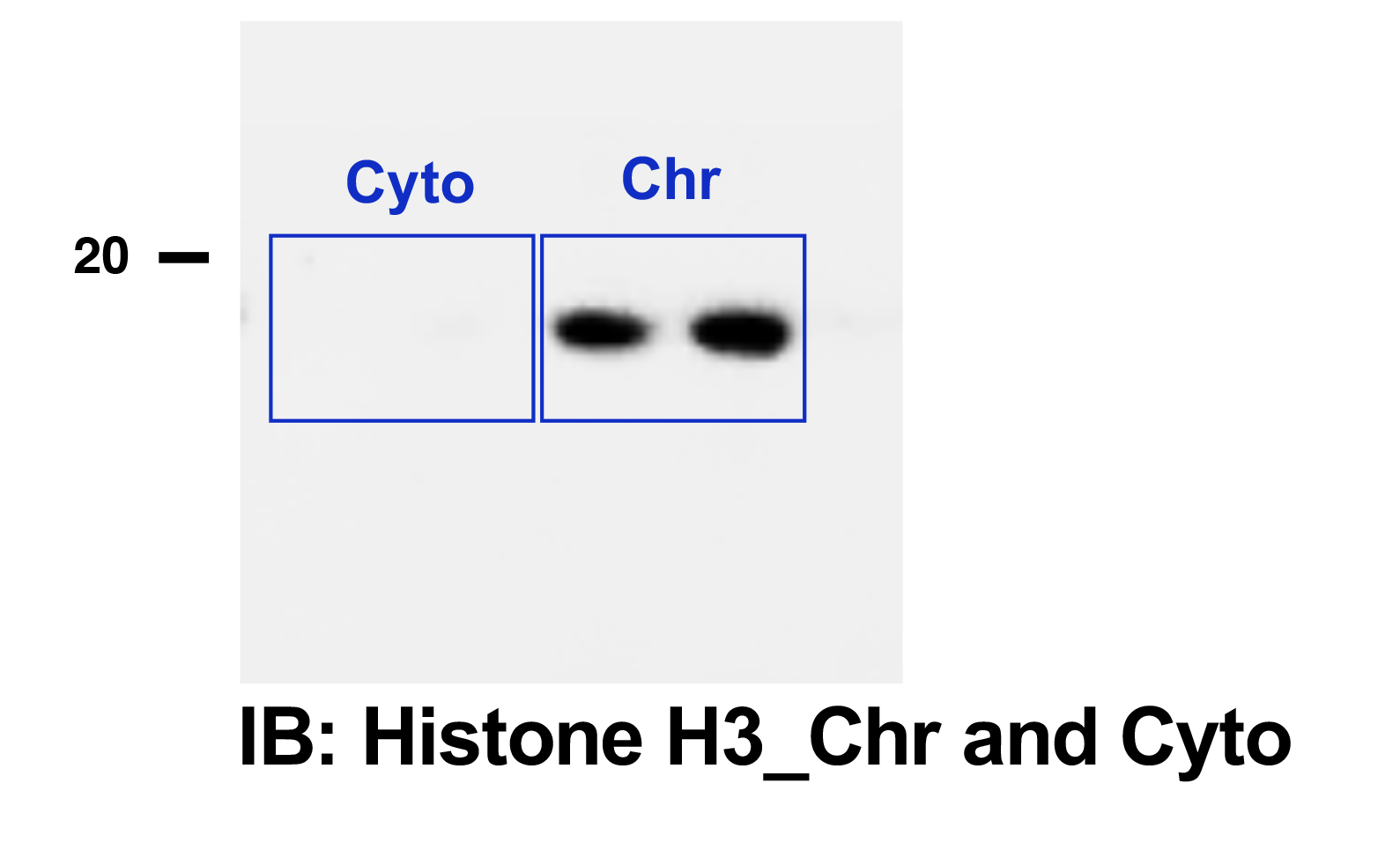

Supplement: Supplementary file 8 — Figure EV1-5 Source Data [file 44319_2024_354_MOESM8_ESM.zip › Figure EV1-5/Figure EV3/EV3E/Histone H3_chromatin and cytoplasm.tif]

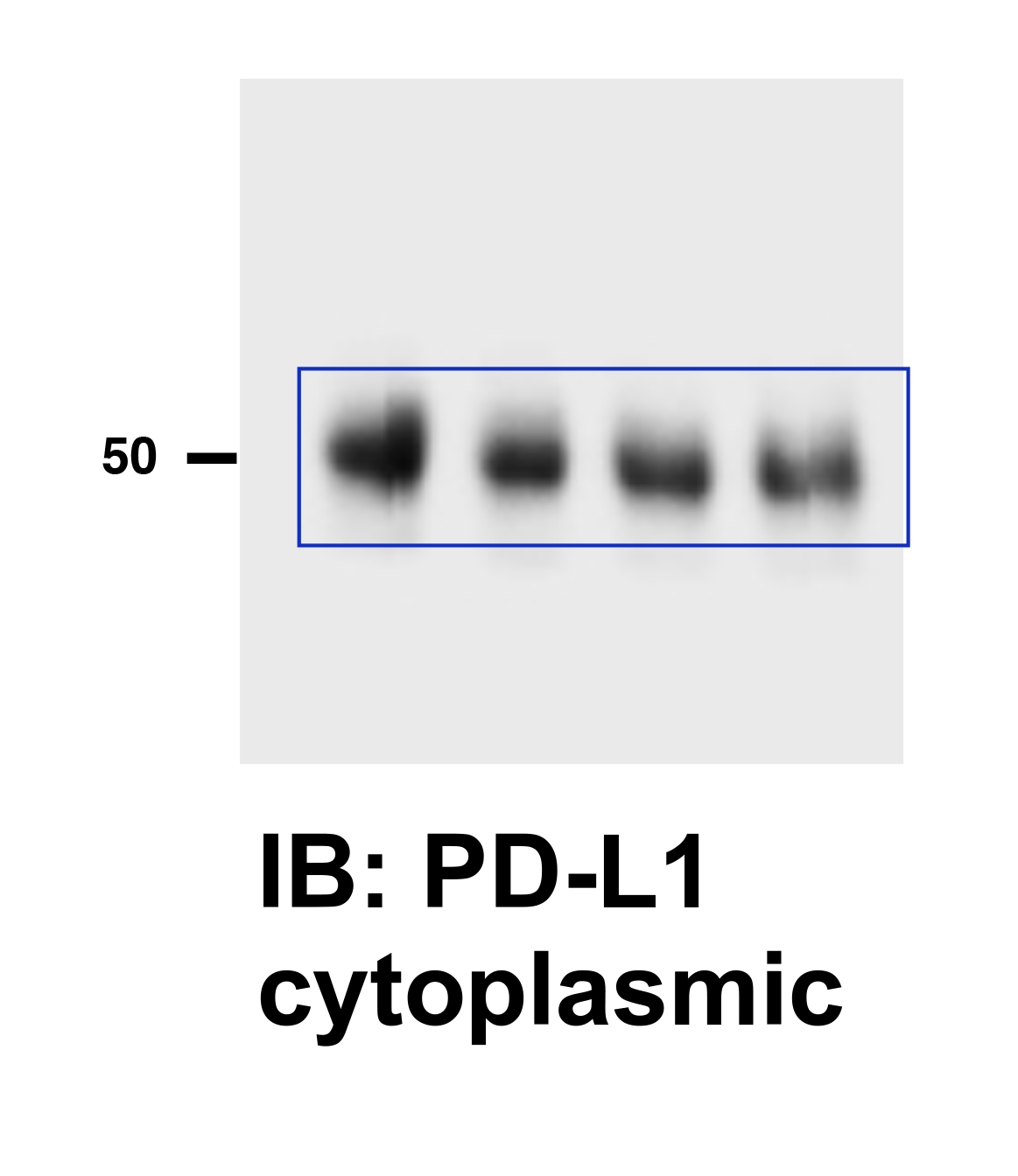

Supplement: Supplementary file 8 — Figure EV1-5 Source Data [file 44319_2024_354_MOESM8_ESM.zip › Figure EV1-5/Figure EV5/EV5C/PD-L1_cytoplasm.tif]

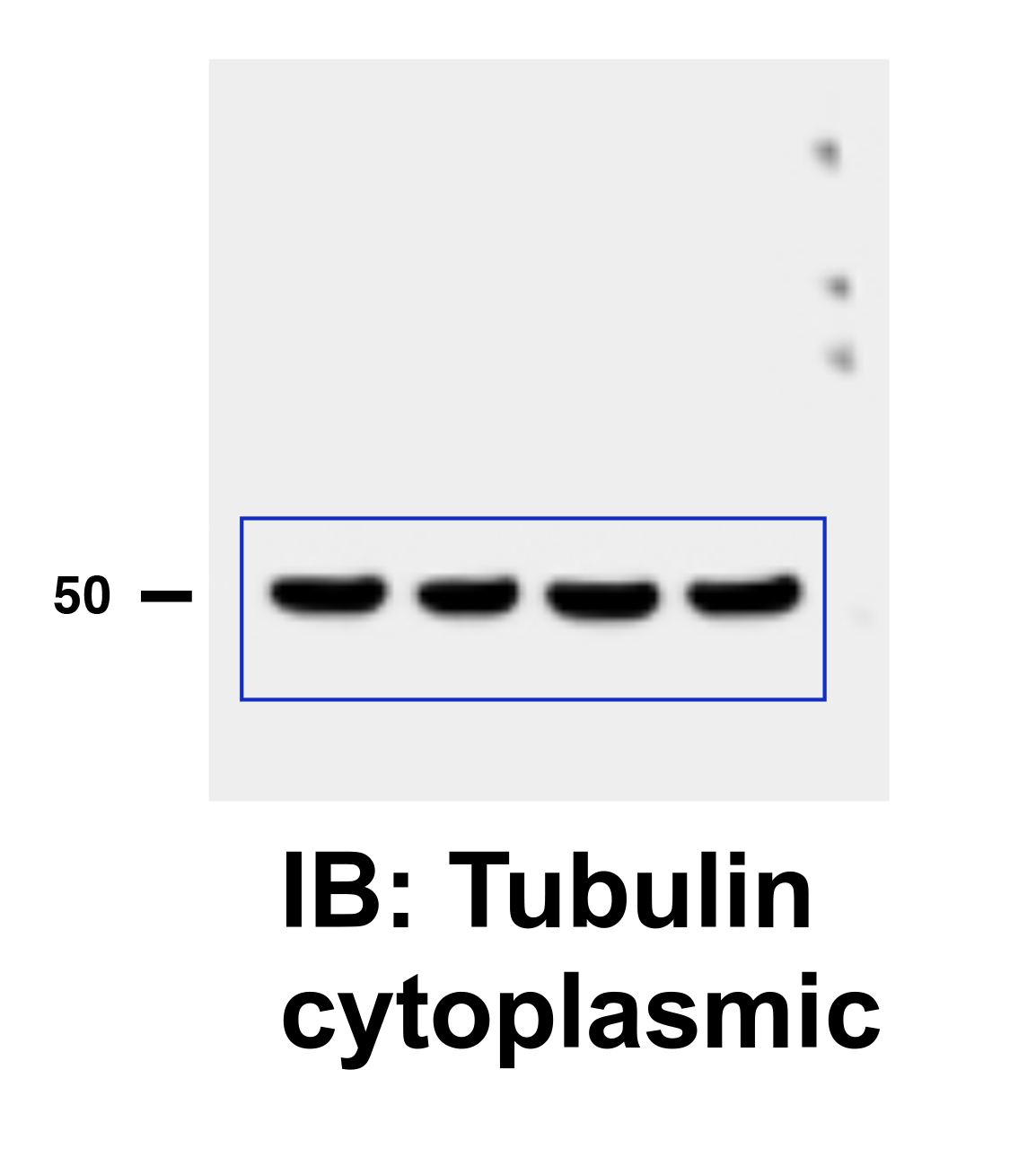

Supplement: Supplementary file 8 — Figure EV1-5 Source Data [file 44319_2024_354_MOESM8_ESM.zip › Figure EV1-5/Figure EV5/EV5C/Tubulin_cytoplasm.tif]

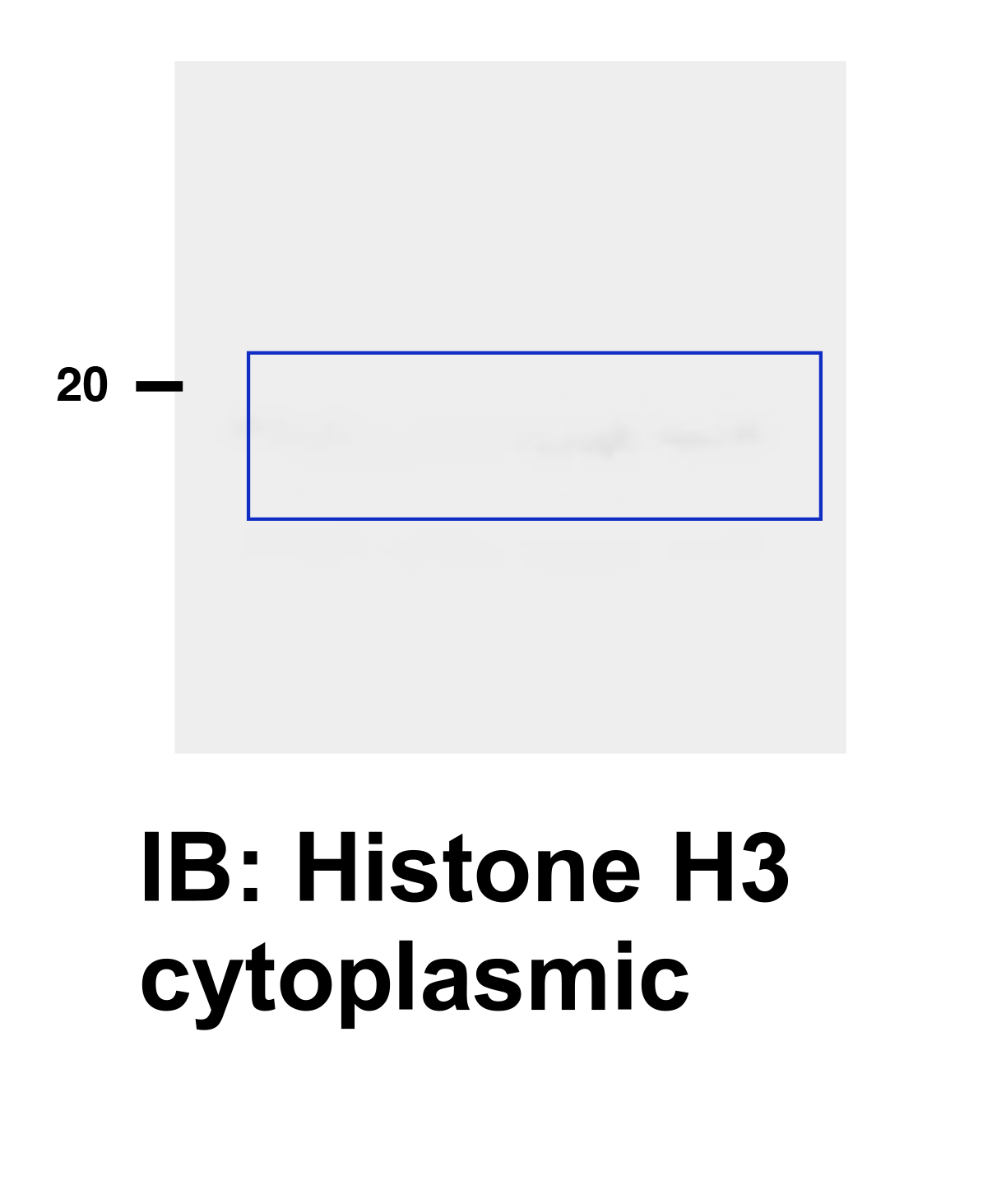

Supplement: Supplementary file 8 — Figure EV1-5 Source Data [file 44319_2024_354_MOESM8_ESM.zip › Figure EV1-5/Figure EV5/EV5C/Histone H3_cytolasm.tif]

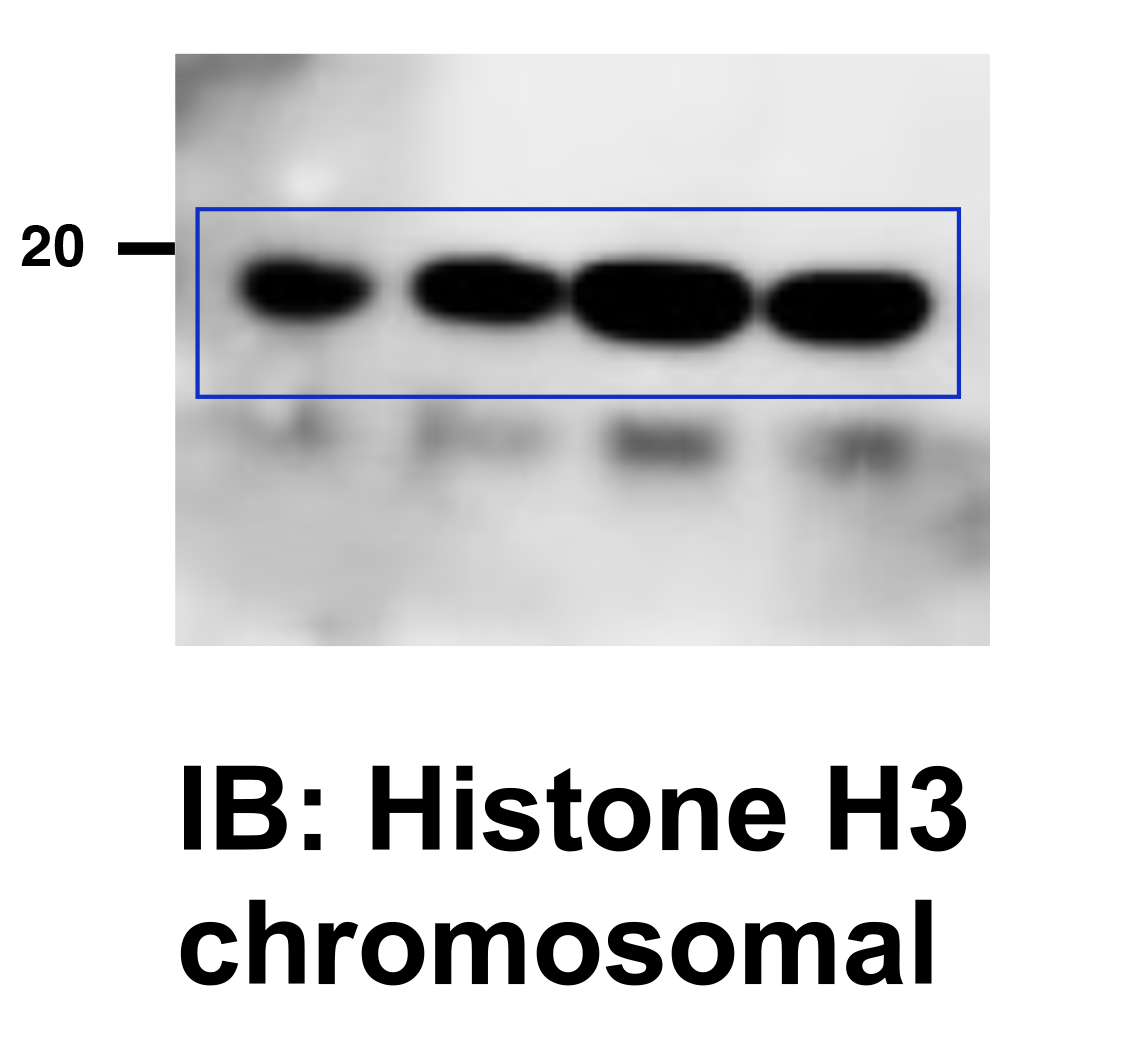

Supplement: Supplementary file 8 — Figure EV1-5 Source Data [file 44319_2024_354_MOESM8_ESM.zip › Figure EV1-5/Figure EV5/EV5C/Histone H3_chromatin.tif]

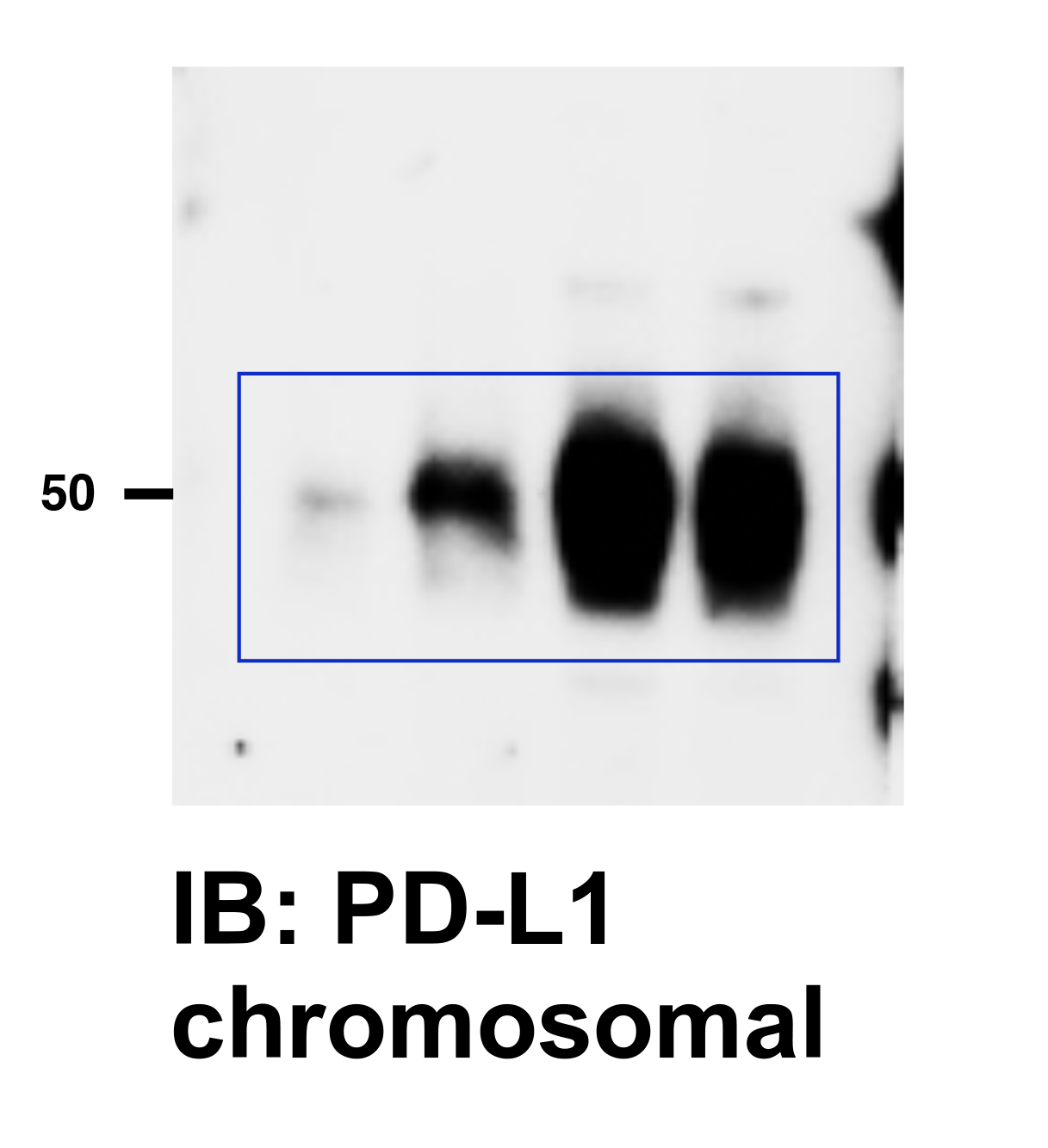

Supplement: Supplementary file 8 — Figure EV1-5 Source Data [file 44319_2024_354_MOESM8_ESM.zip › Figure EV1-5/Figure EV5/EV5C/PD-L1_chromatin.tif]

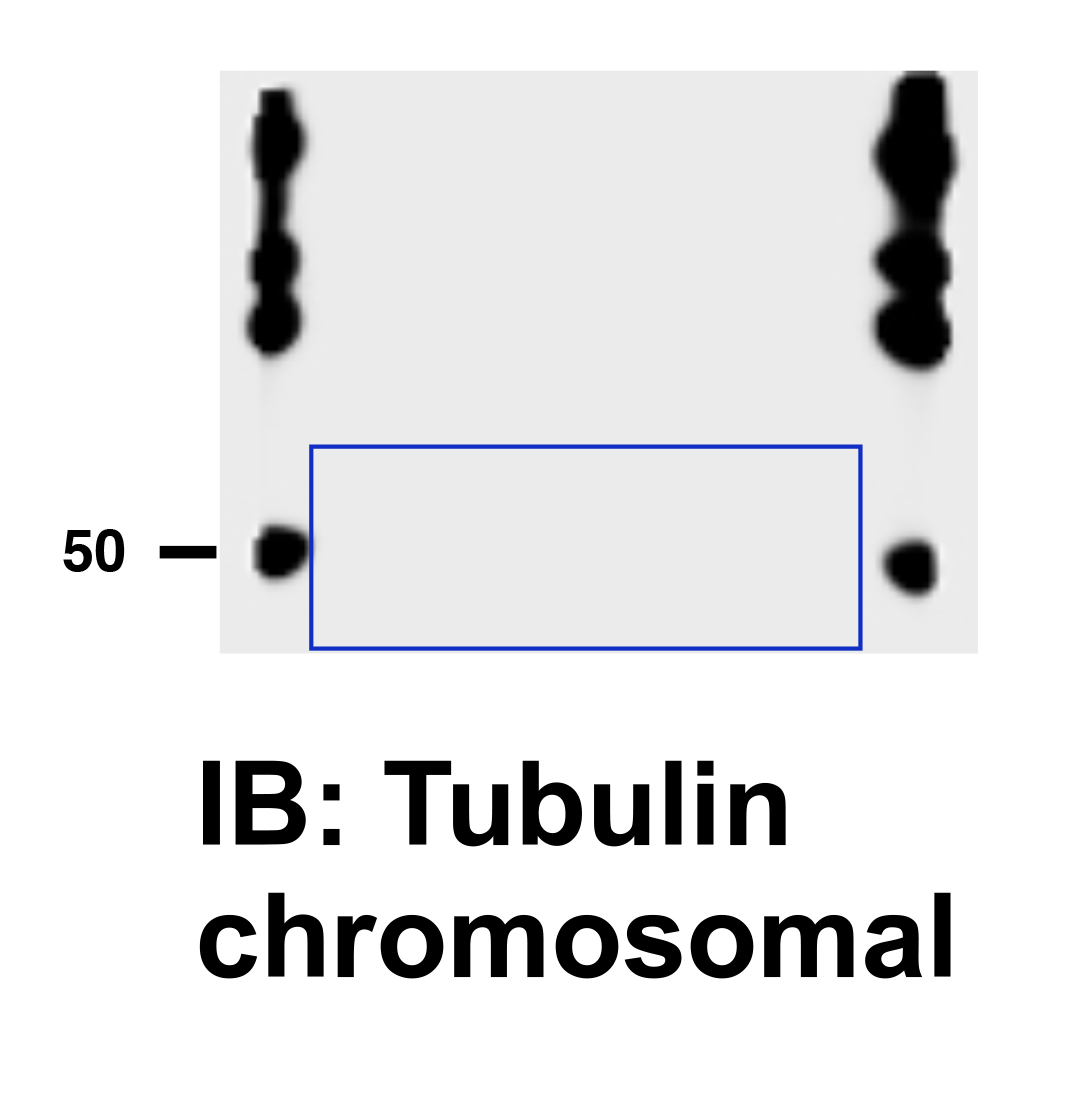

Supplement: Supplementary file 8 — Figure EV1-5 Source Data [file 44319_2024_354_MOESM8_ESM.zip › Figure EV1-5/Figure EV5/EV5C/Tubulin_chromatin.tif]

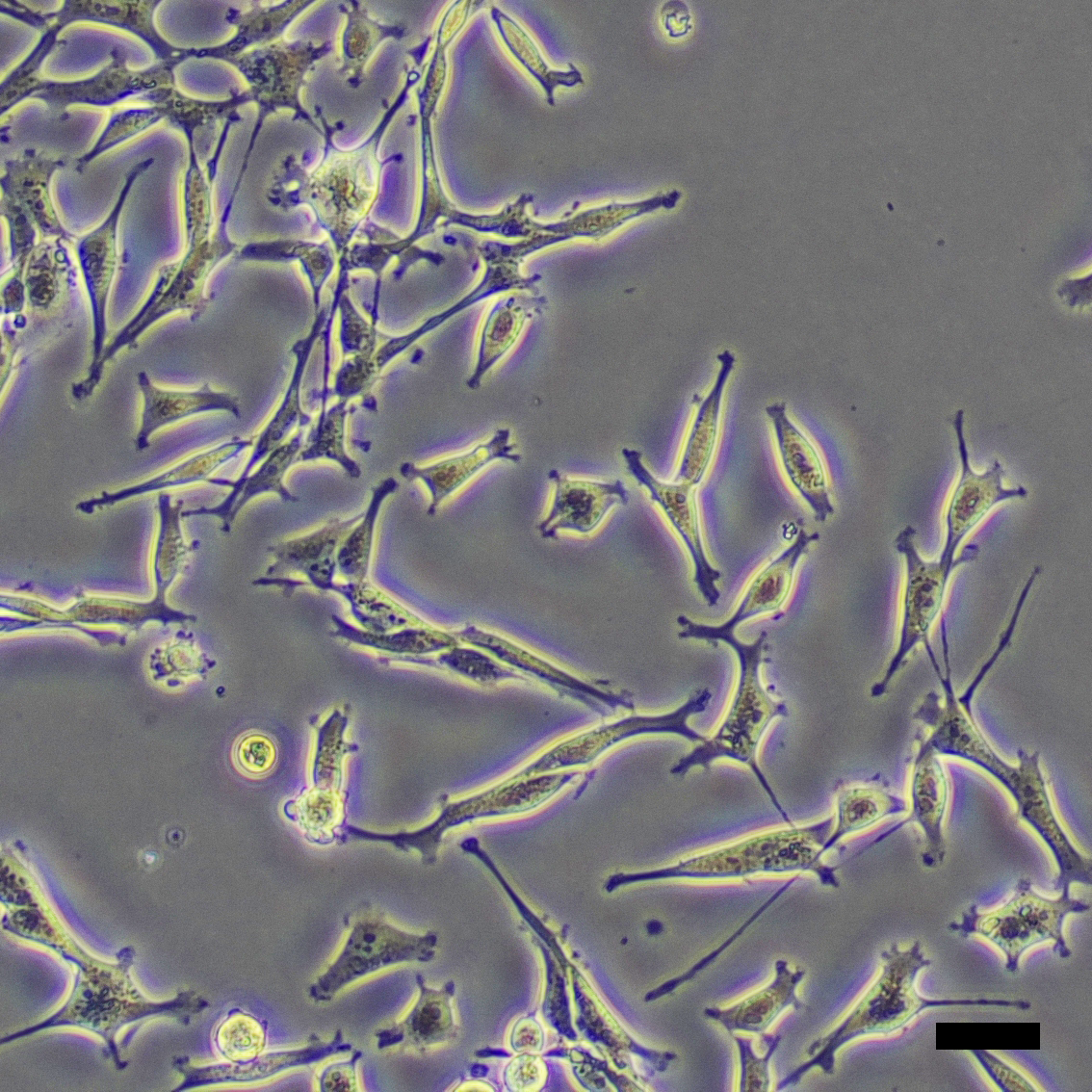

Supplement: Supplementary file 8 — Figure EV1-5 Source Data [file 44319_2024_354_MOESM8_ESM.zip › Figure EV1-5/Figure EV1/EV1C/WT_2h.tif]

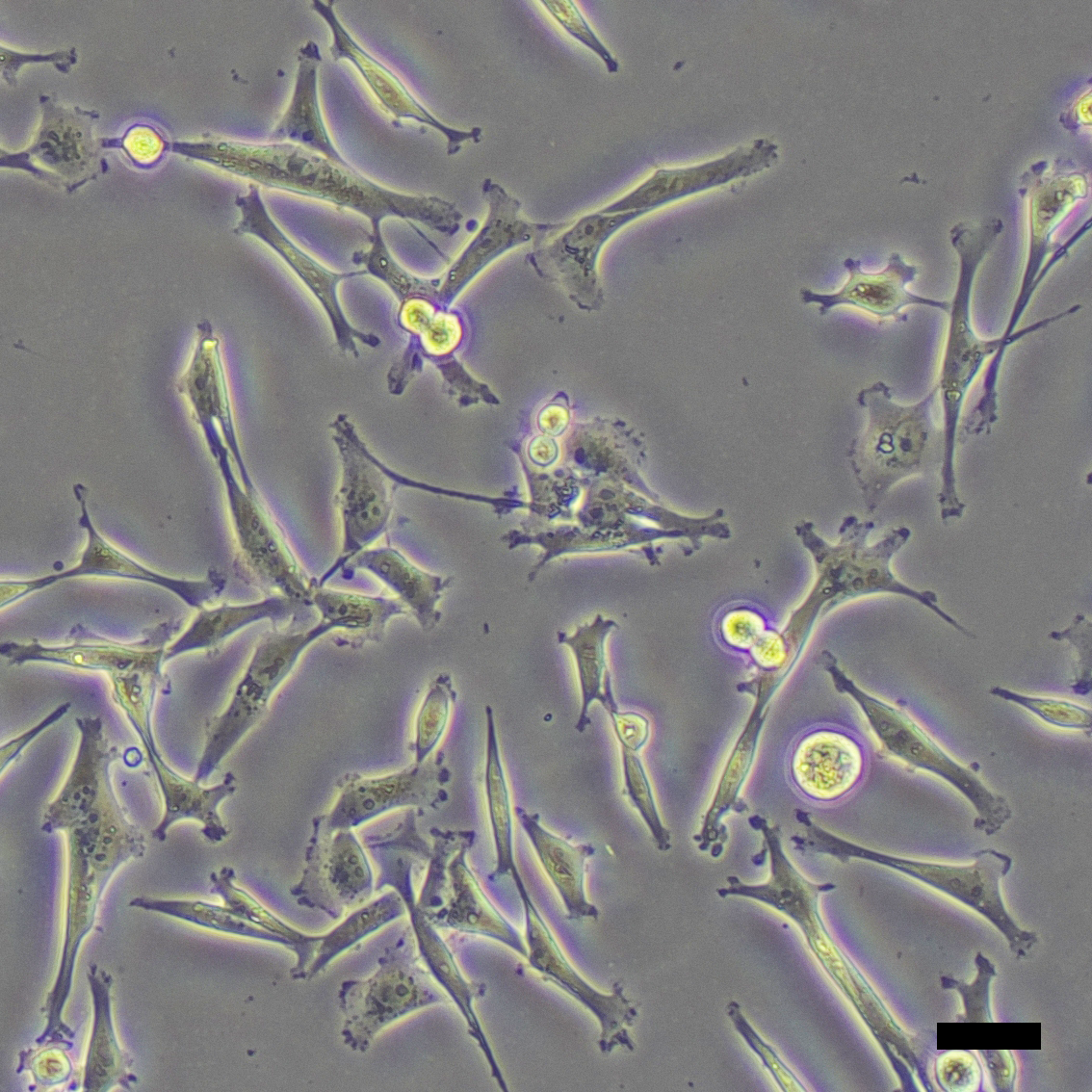

Supplement: Supplementary file 8 — Figure EV1-5 Source Data [file 44319_2024_354_MOESM8_ESM.zip › Figure EV1-5/Figure EV1/EV1C/WT_Control.tif]

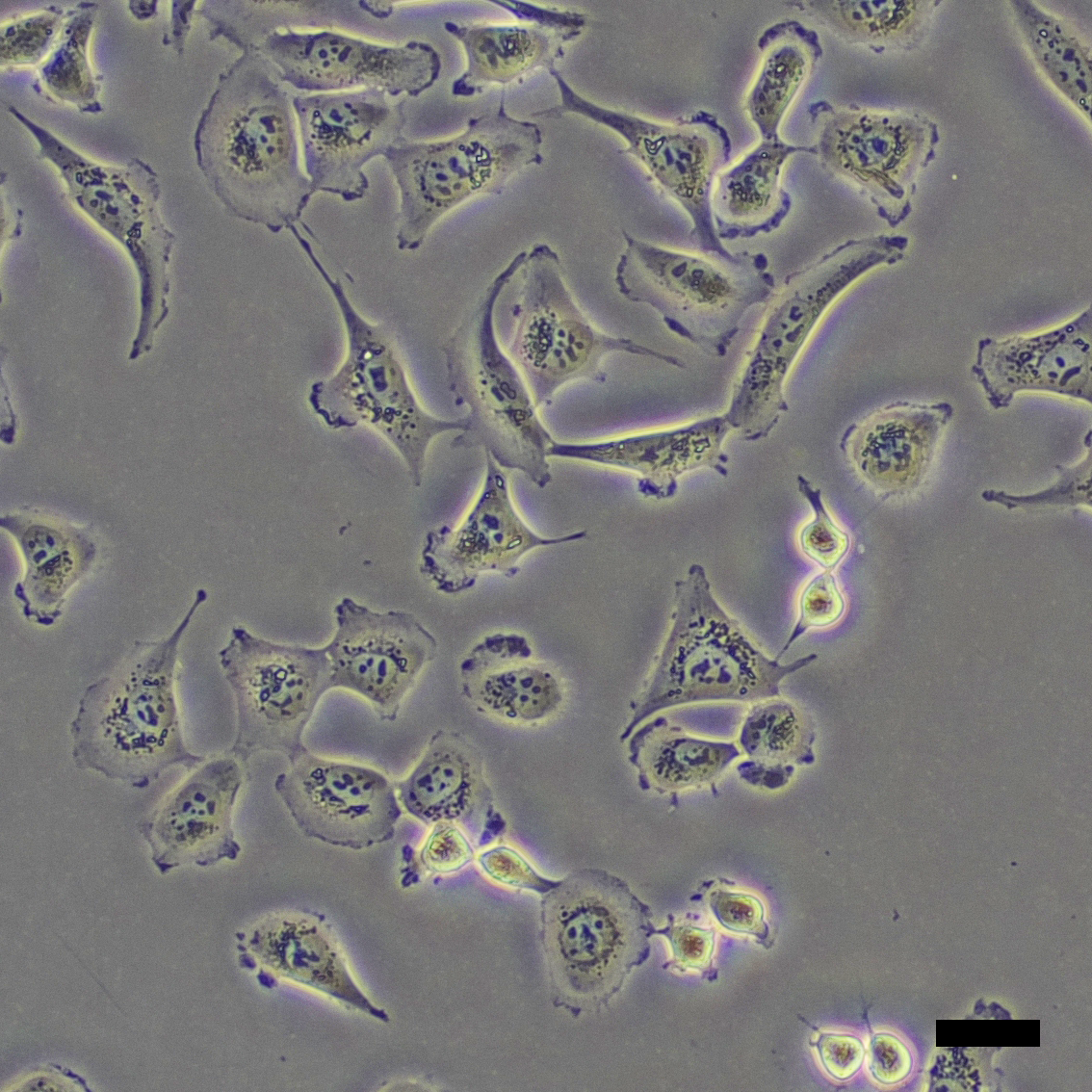

Supplement: Supplementary file 8 — Figure EV1-5 Source Data [file 44319_2024_354_MOESM8_ESM.zip › Figure EV1-5/Figure EV1/EV1C/KO 1h.tif]

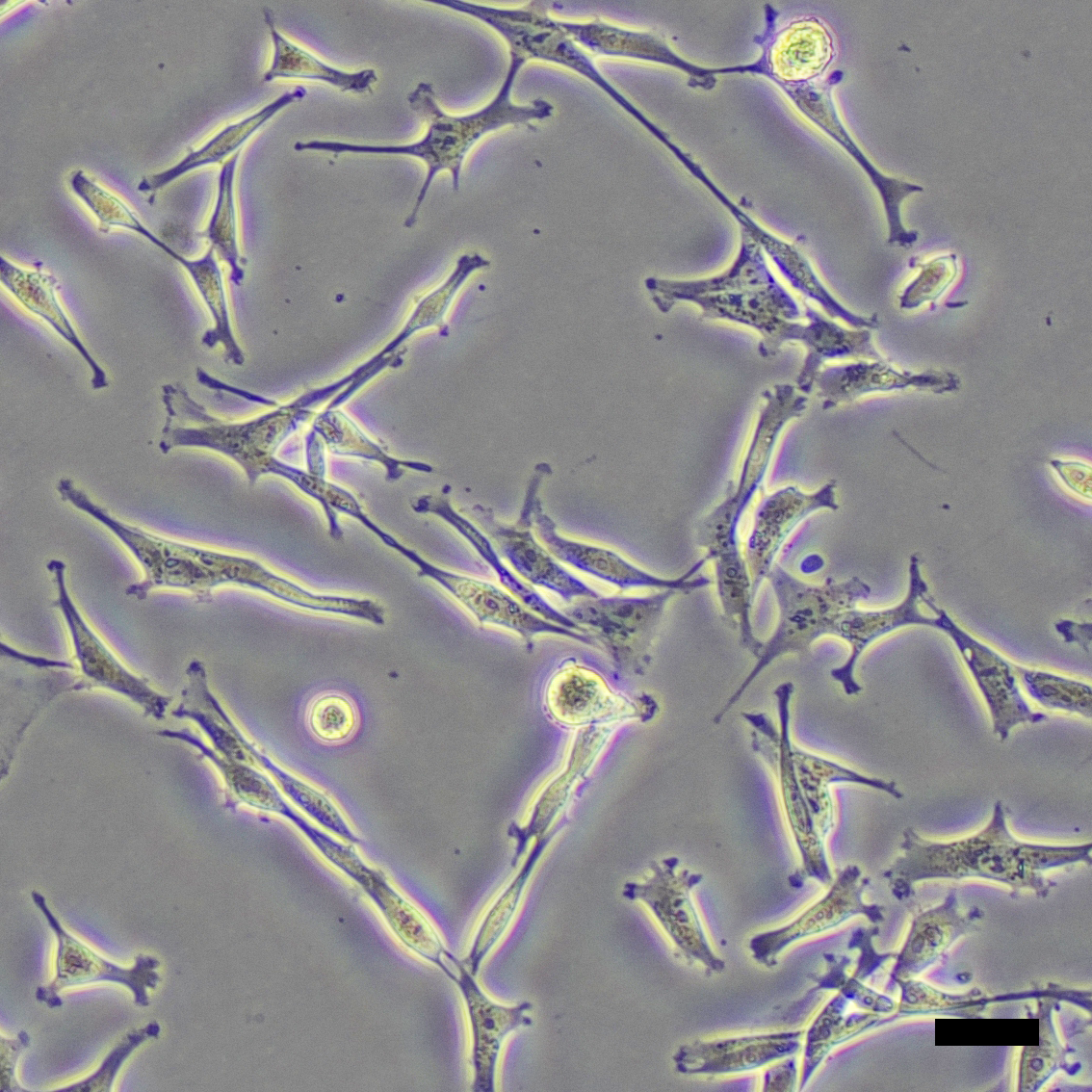

Supplement: Supplementary file 8 — Figure EV1-5 Source Data [file 44319_2024_354_MOESM8_ESM.zip › Figure EV1-5/Figure EV1/EV1C/WT_4h.tif]

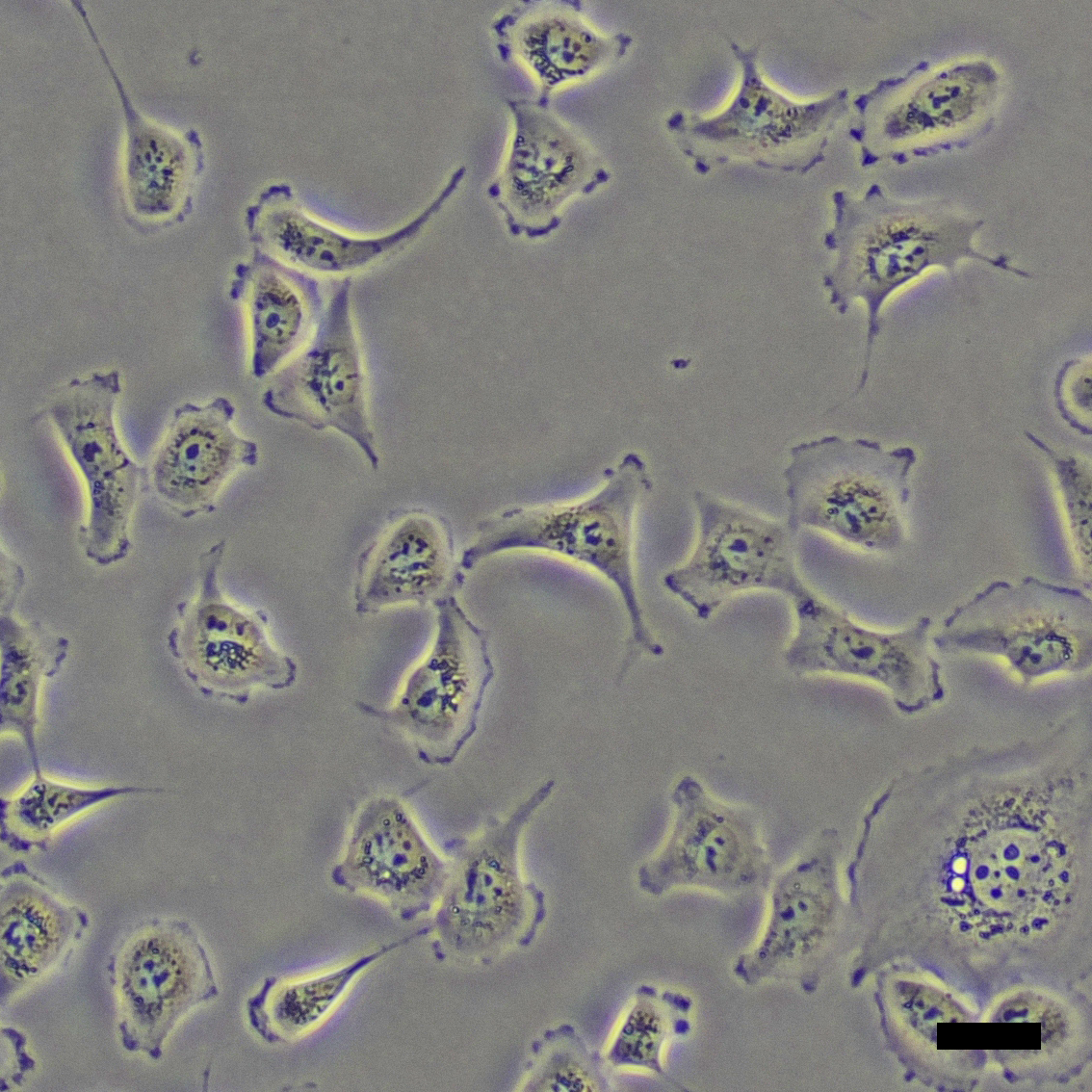

Supplement: Supplementary file 8 — Figure EV1-5 Source Data [file 44319_2024_354_MOESM8_ESM.zip › Figure EV1-5/Figure EV1/EV1C/KO 2h.tif]

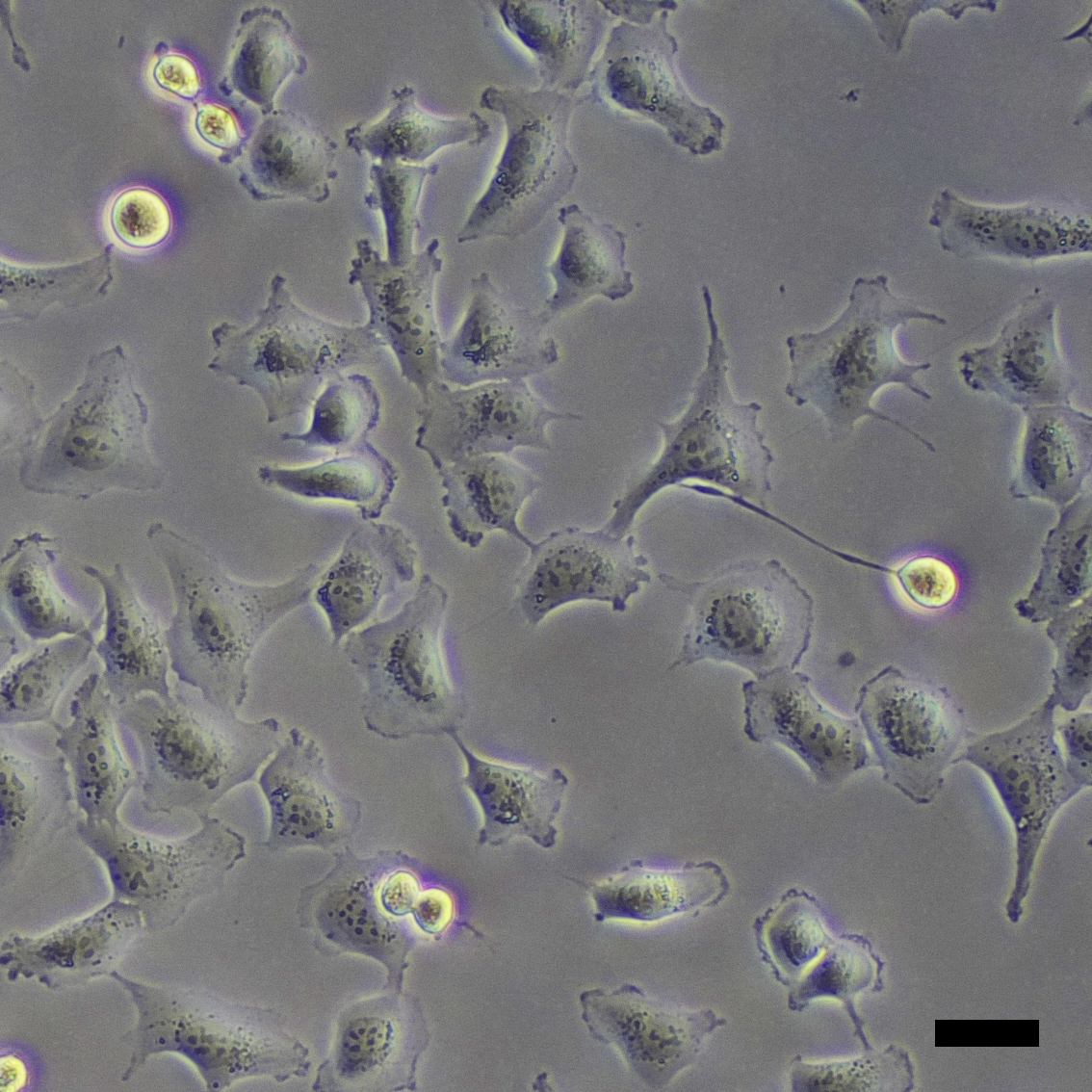

Supplement: Supplementary file 8 — Figure EV1-5 Source Data [file 44319_2024_354_MOESM8_ESM.zip › Figure EV1-5/Figure EV1/EV1C/KO control.tif]

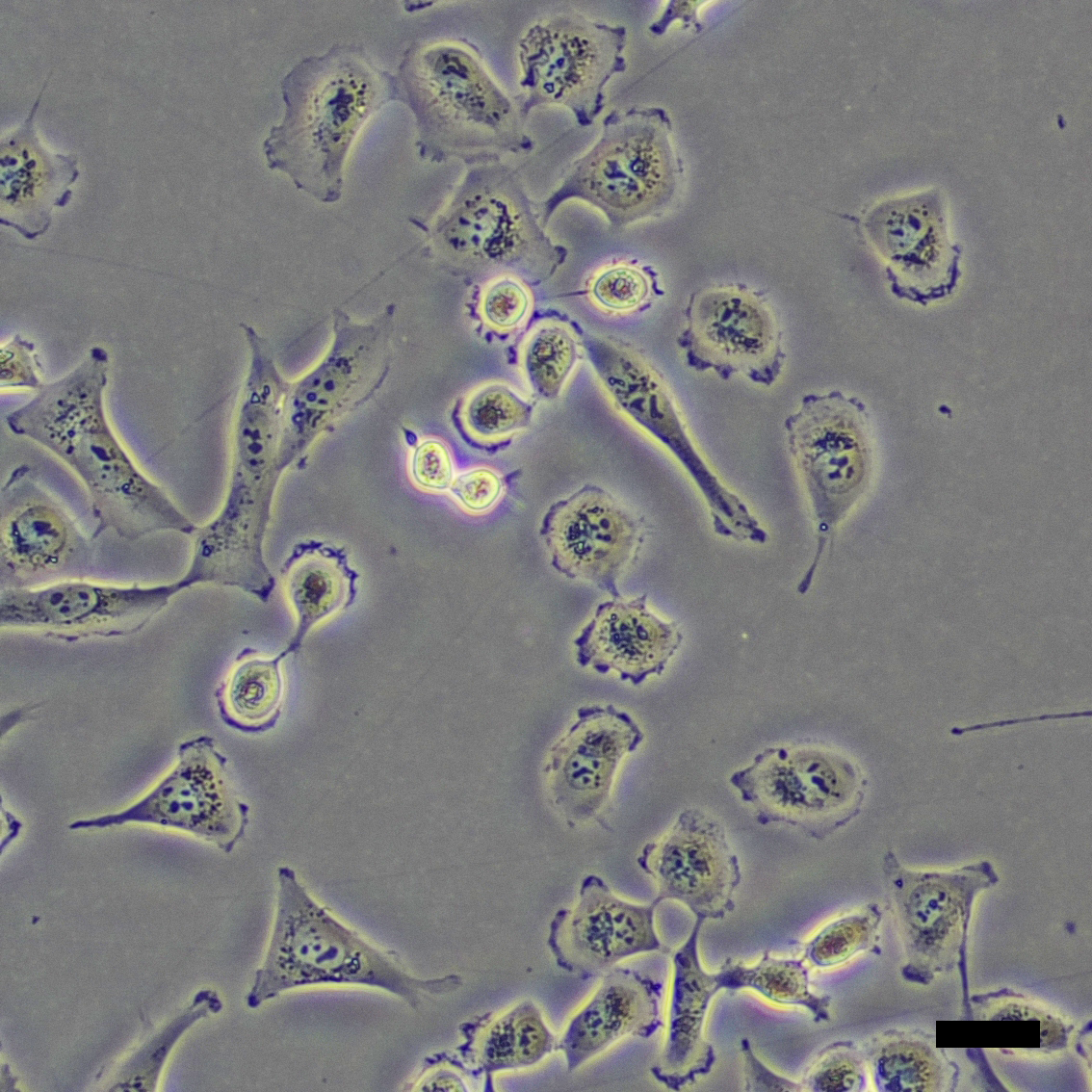

Supplement: Supplementary file 8 — Figure EV1-5 Source Data [file 44319_2024_354_MOESM8_ESM.zip › Figure EV1-5/Figure EV1/EV1C/KO 0.5h.tif]

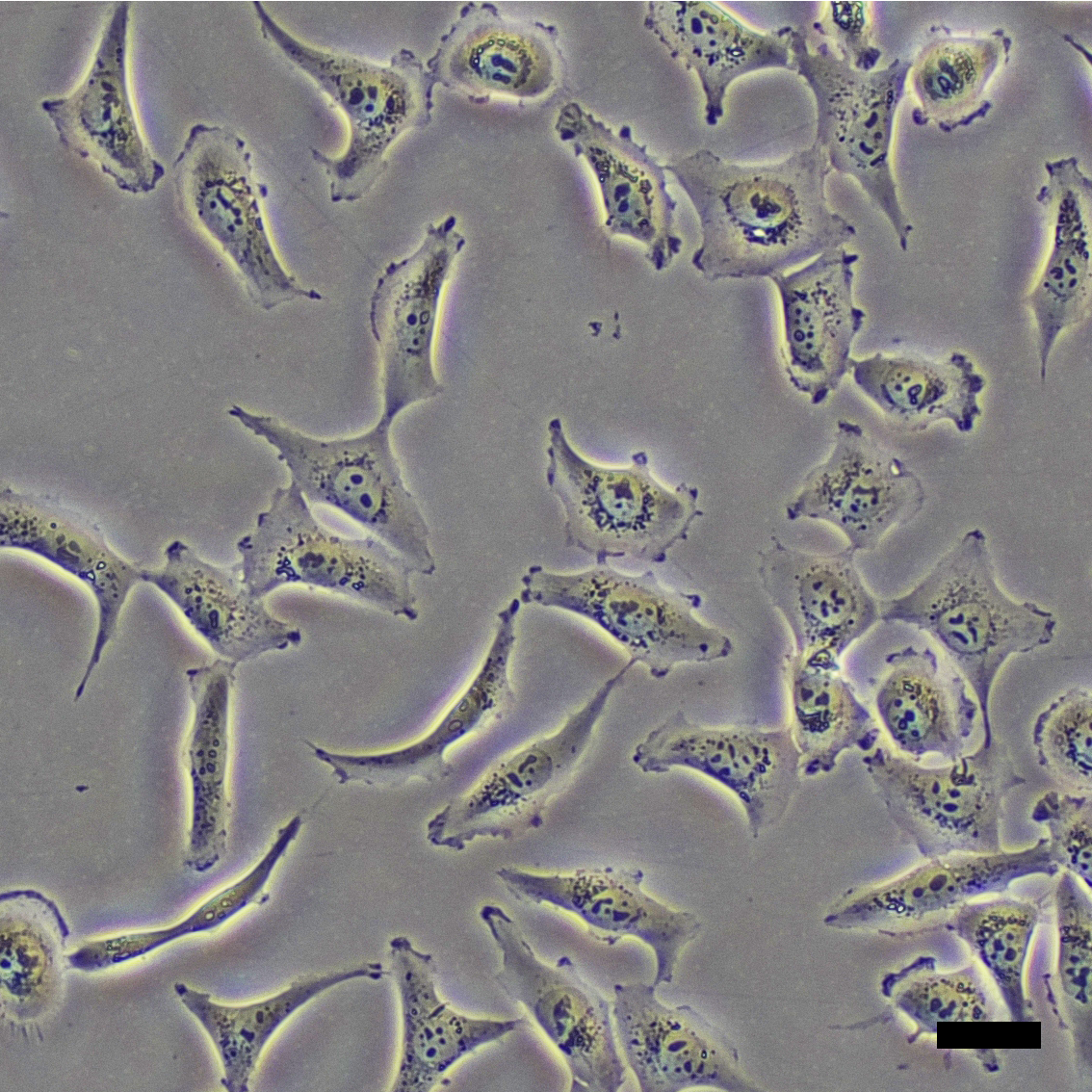

Supplement: Supplementary file 8 — Figure EV1-5 Source Data [file 44319_2024_354_MOESM8_ESM.zip › Figure EV1-5/Figure EV1/EV1C/KO 4h.tif]

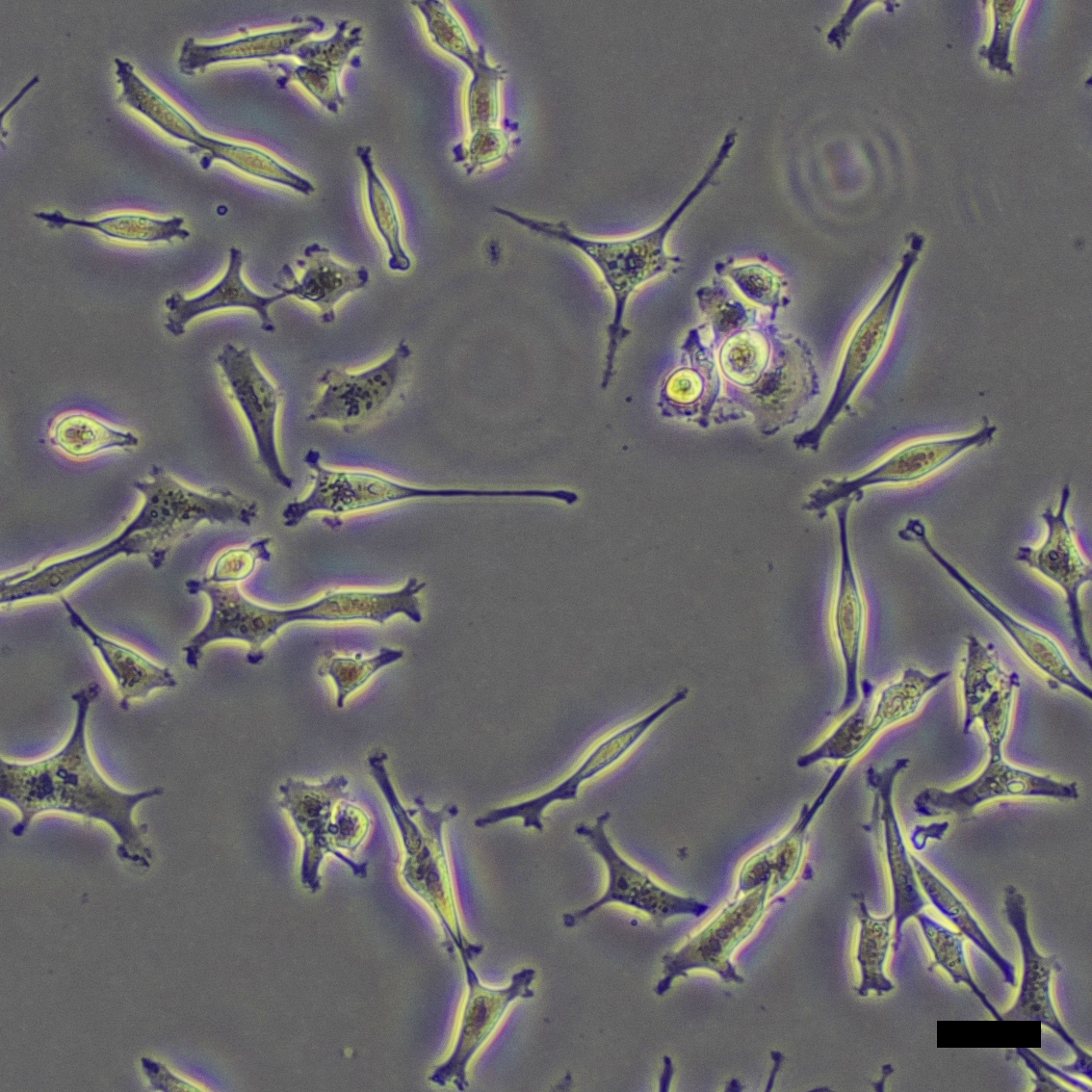

Supplement: Supplementary file 8 — Figure EV1-5 Source Data [file 44319_2024_354_MOESM8_ESM.zip › Figure EV1-5/Figure EV1/EV1C/WT_1h.tif]

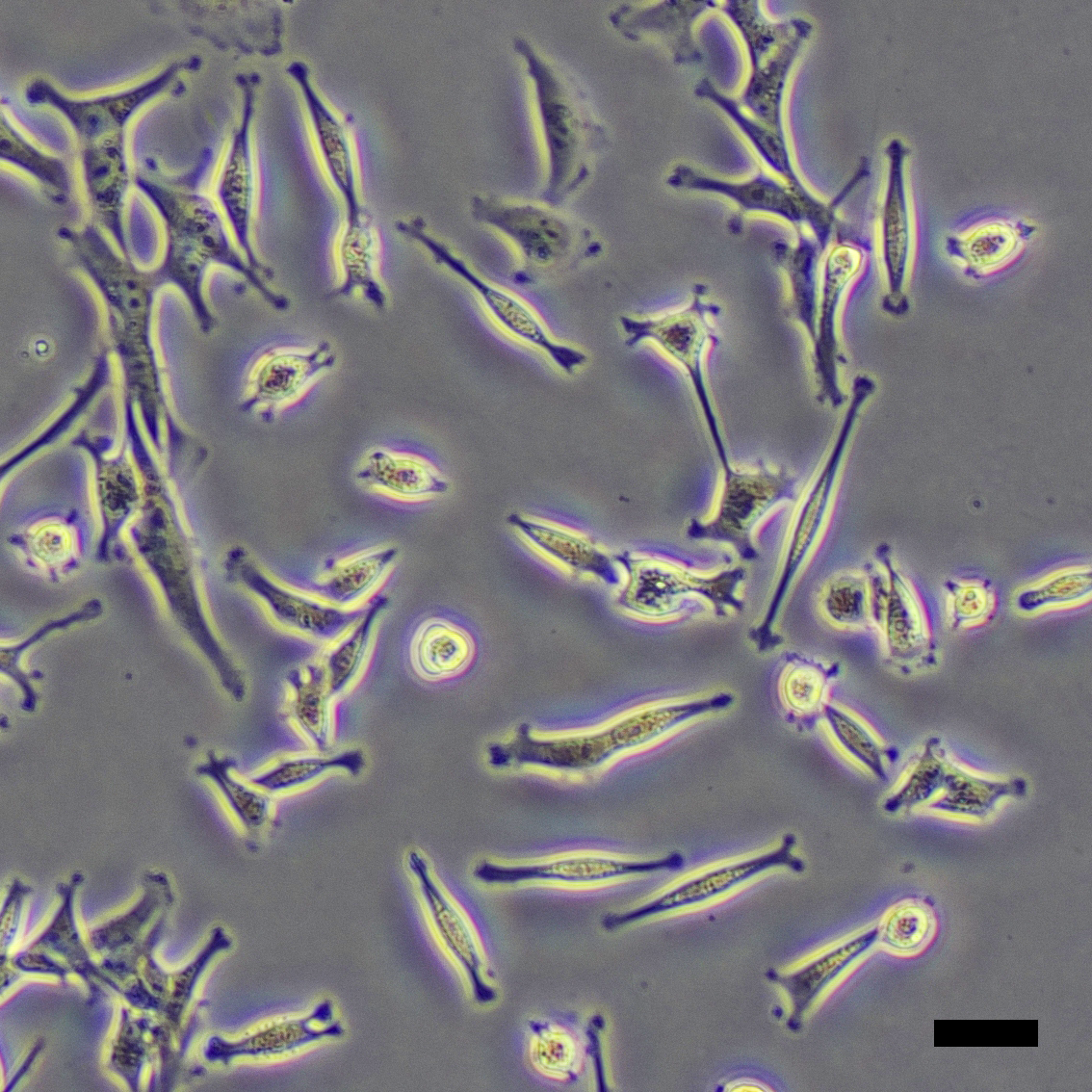

Supplement: Supplementary file 8 — Figure EV1-5 Source Data [file 44319_2024_354_MOESM8_ESM.zip › Figure EV1-5/Figure EV1/EV1C/WT_0.5h.tif]

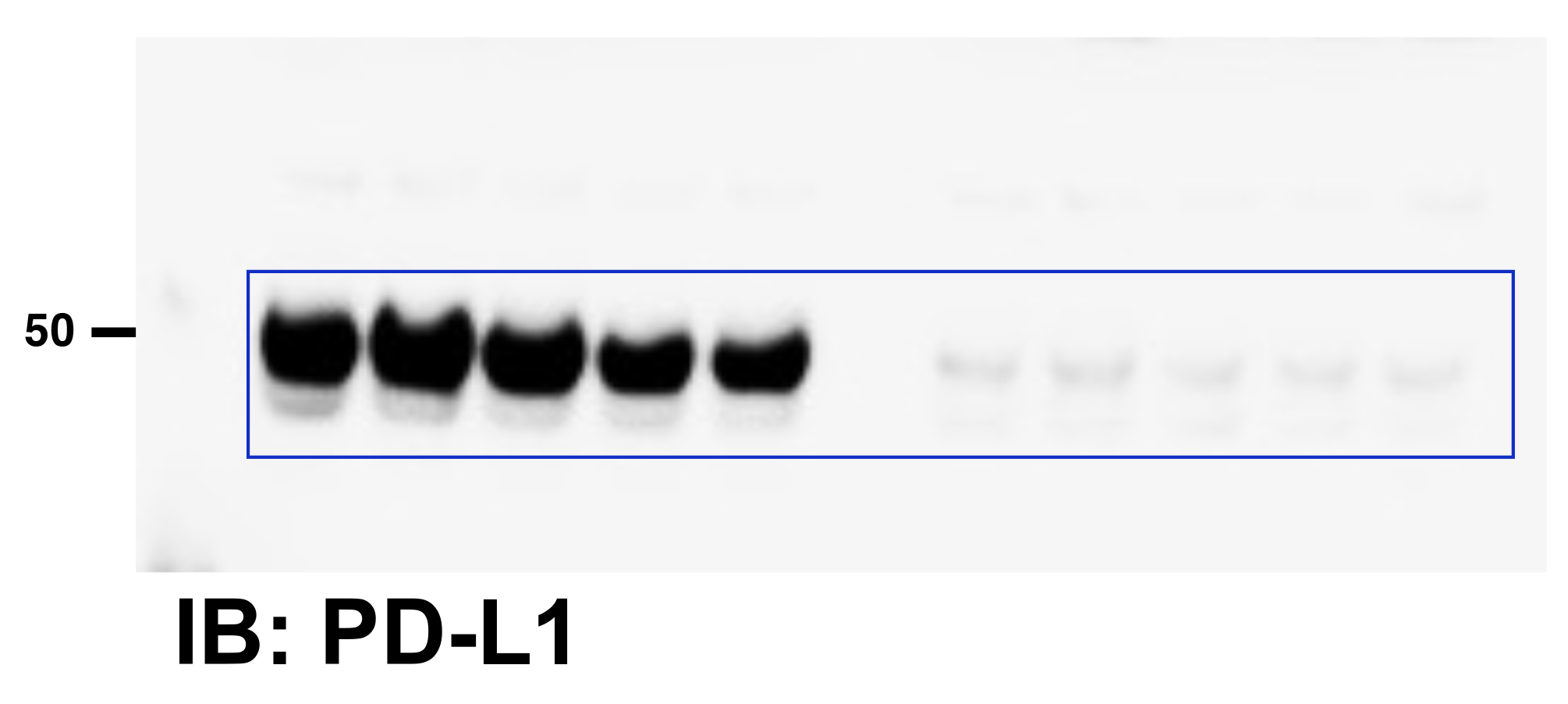

Supplement: Supplementary file 8 — Figure EV1-5 Source Data [file 44319_2024_354_MOESM8_ESM.zip › Figure EV1-5/Figure EV1/EV1B/PD-L1.tif]

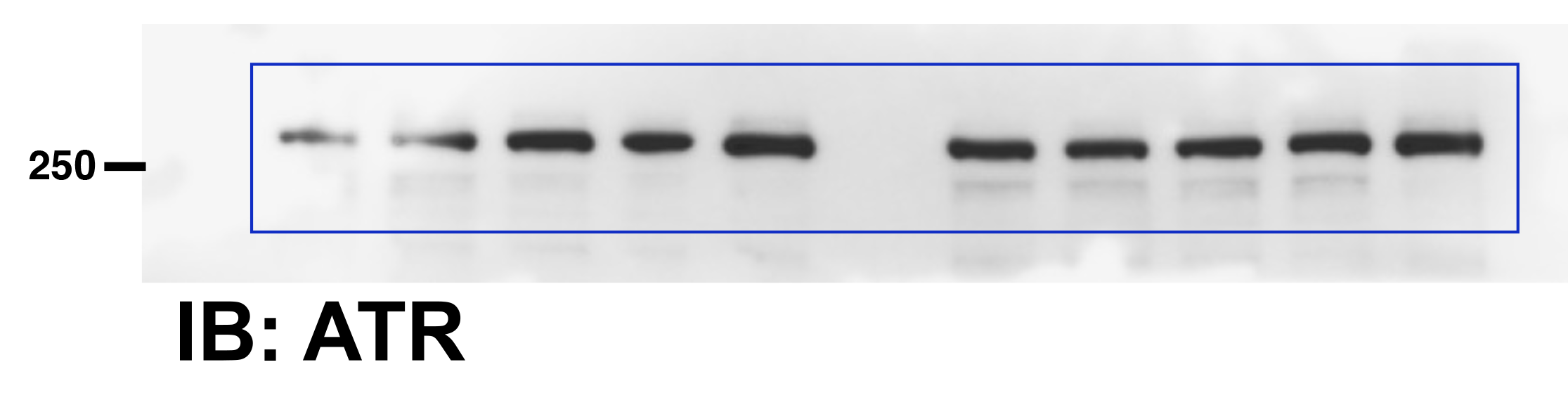

Supplement: Supplementary file 8 — Figure EV1-5 Source Data [file 44319_2024_354_MOESM8_ESM.zip › Figure EV1-5/Figure EV1/EV1B/ATR.tif]

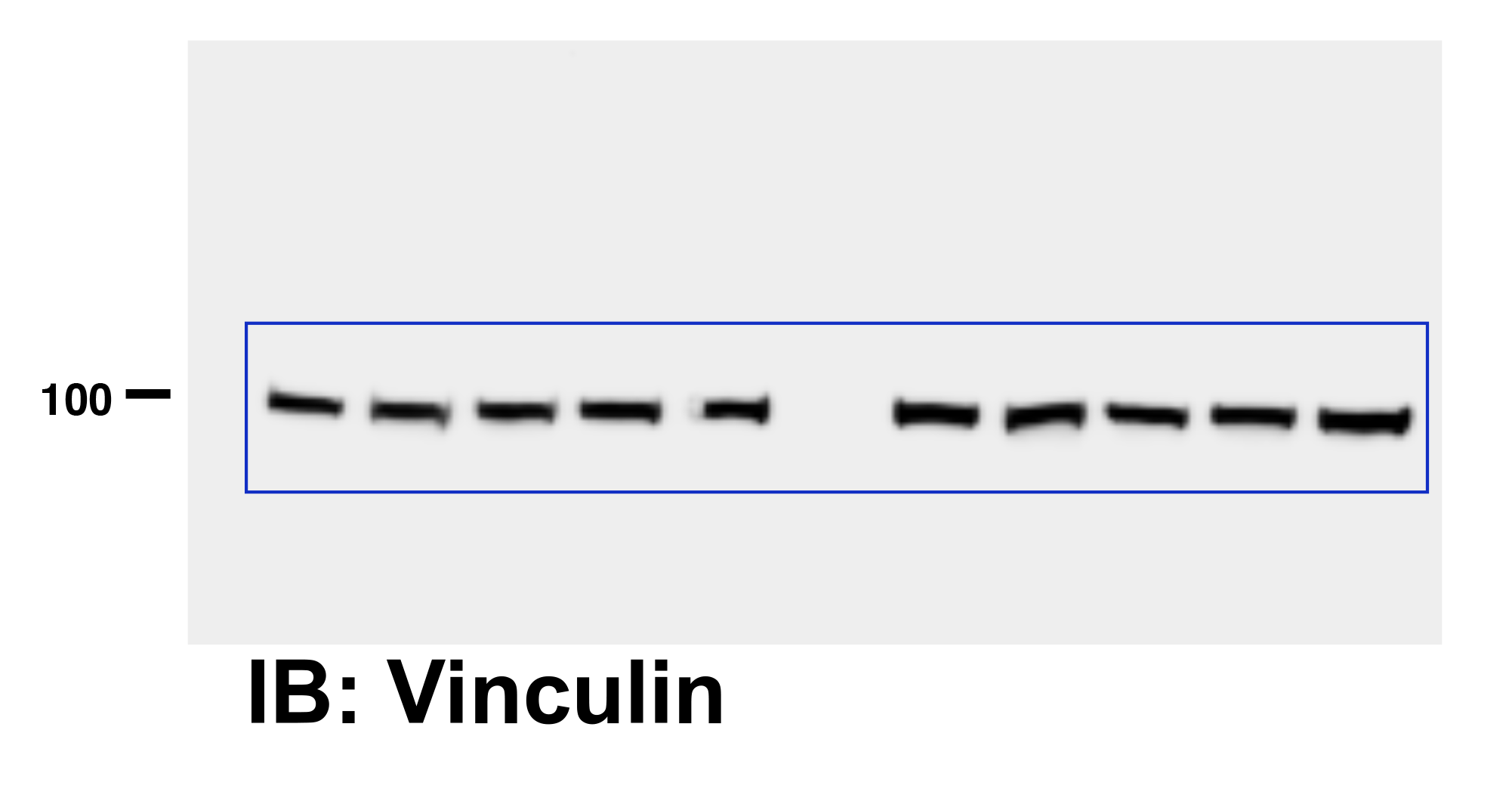

Supplement: Supplementary file 8 — Figure EV1-5 Source Data [file 44319_2024_354_MOESM8_ESM.zip › Figure EV1-5/Figure EV1/EV1B/Vinculin.tif]

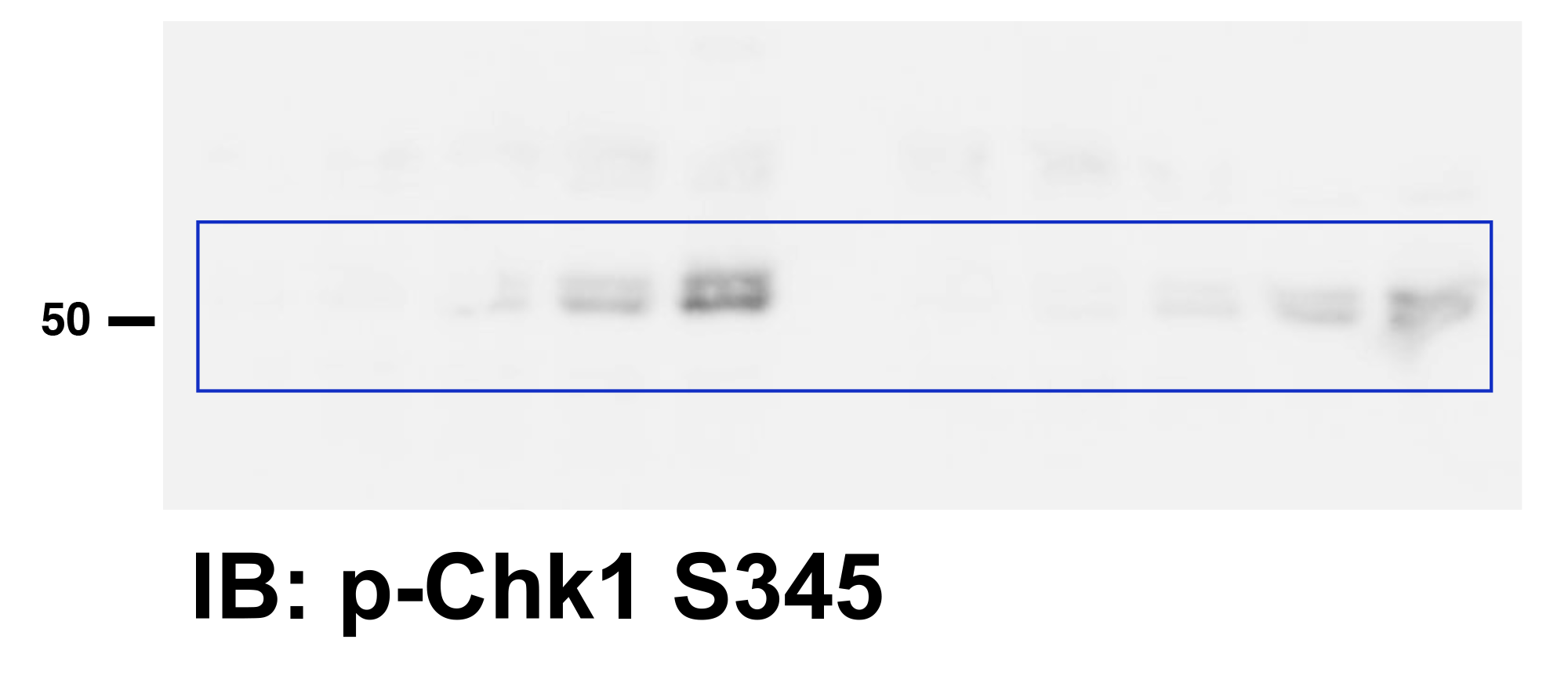

Supplement: Supplementary file 8 — Figure EV1-5 Source Data [file 44319_2024_354_MOESM8_ESM.zip › Figure EV1-5/Figure EV1/EV1B/pChk1.tif]

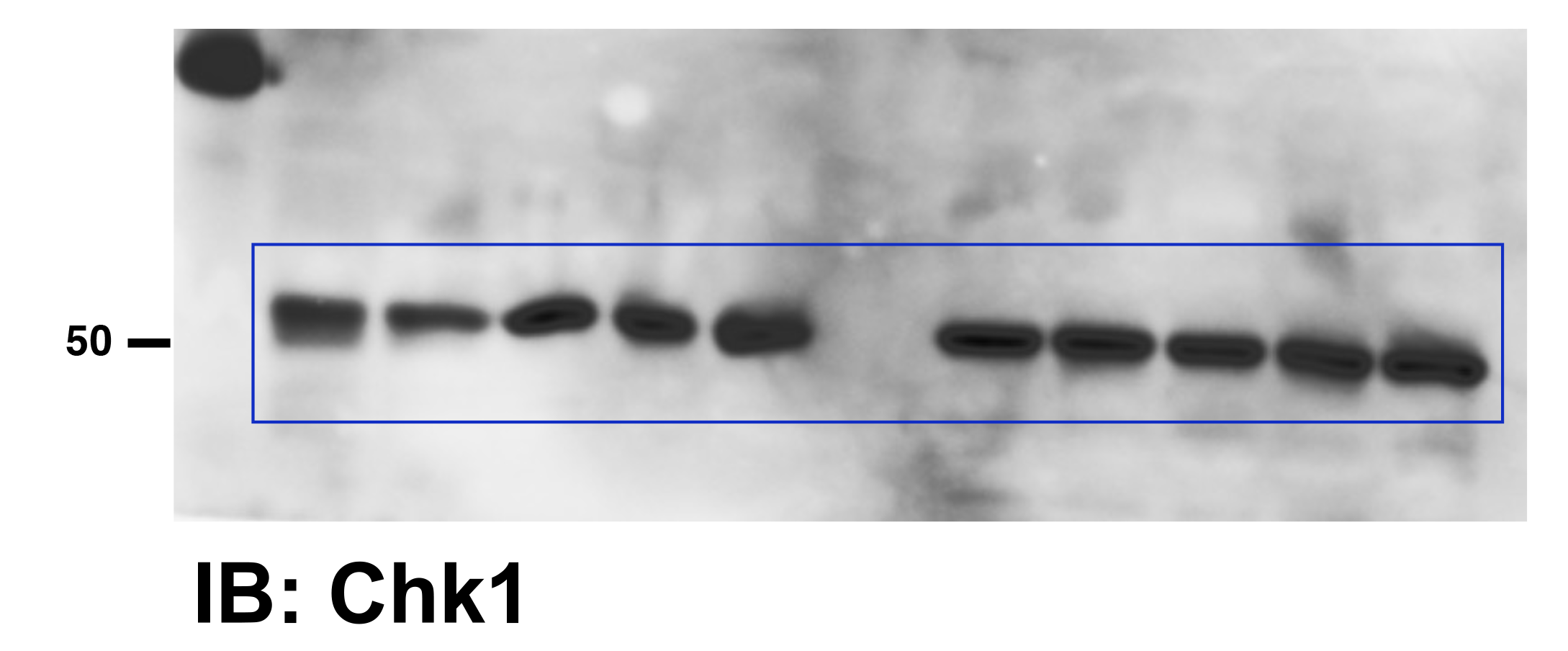

Supplement: Supplementary file 8 — Figure EV1-5 Source Data [file 44319_2024_354_MOESM8_ESM.zip › Figure EV1-5/Figure EV1/EV1B/Chk1.tif]

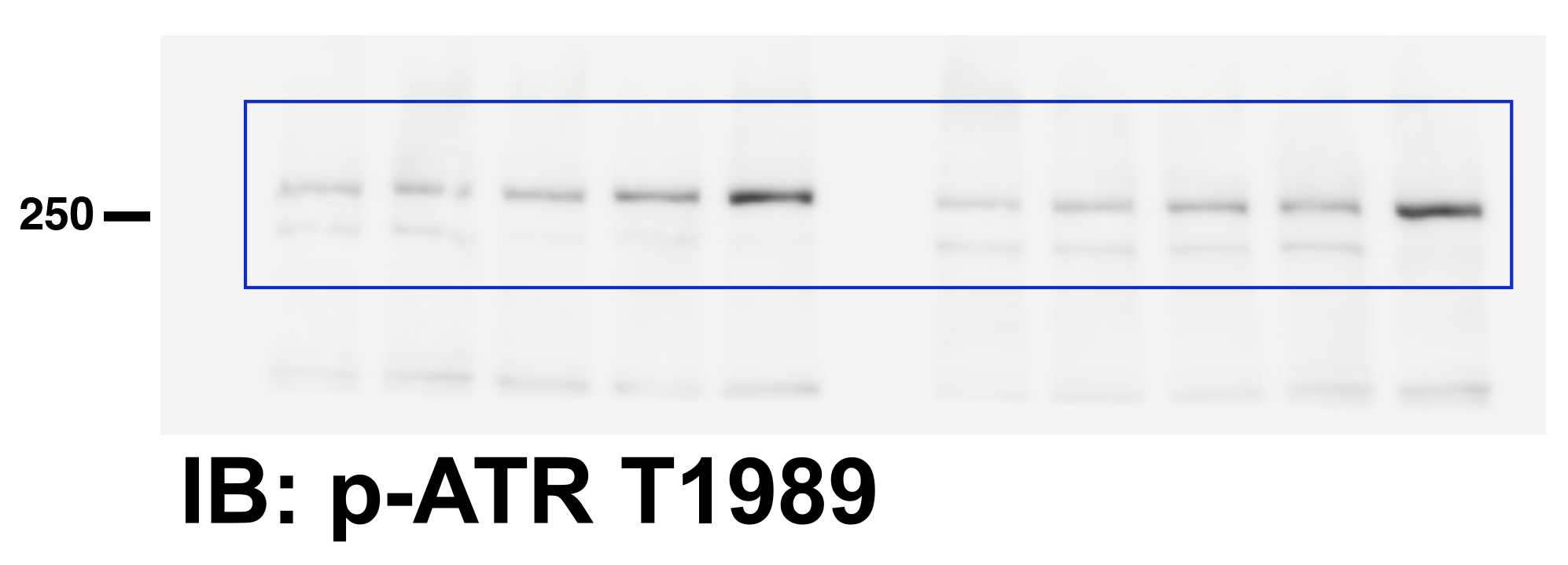

Supplement: Supplementary file 8 — Figure EV1-5 Source Data [file 44319_2024_354_MOESM8_ESM.zip › Figure EV1-5/Figure EV1/EV1B/pATR.tif]

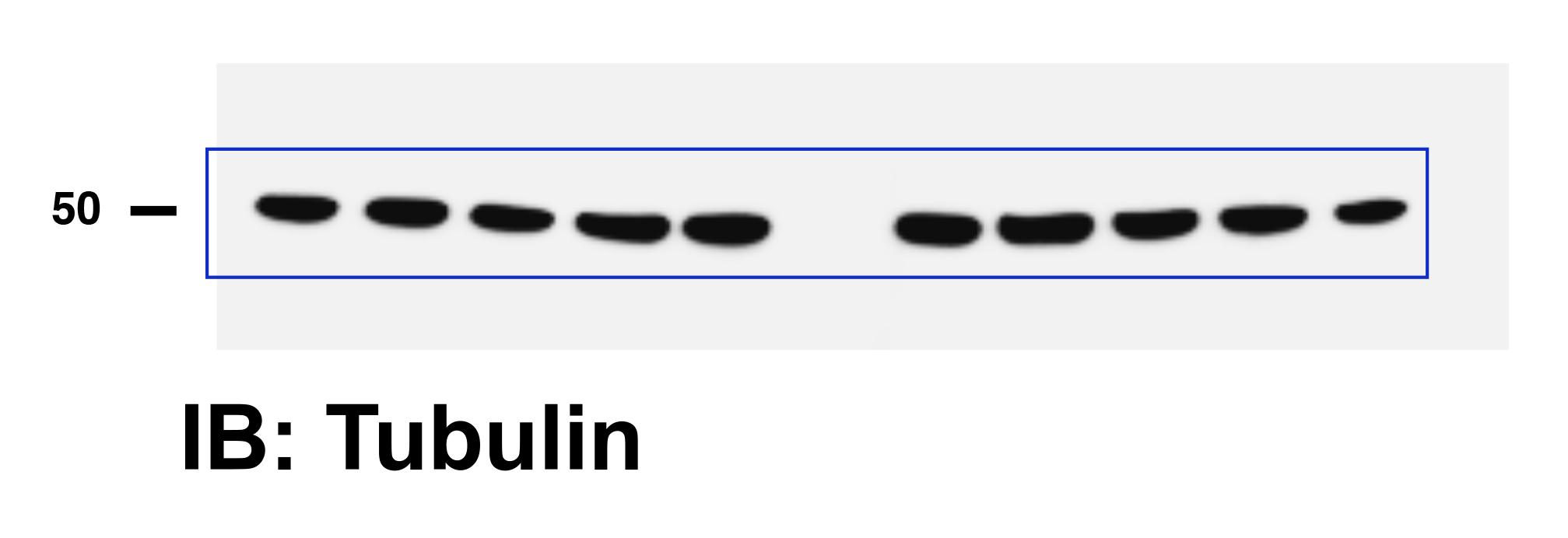

Supplement: Supplementary file 8 — Figure EV1-5 Source Data [file 44319_2024_354_MOESM8_ESM.zip › Figure EV1-5/Figure EV1/EV1A/Tubulin.tif]

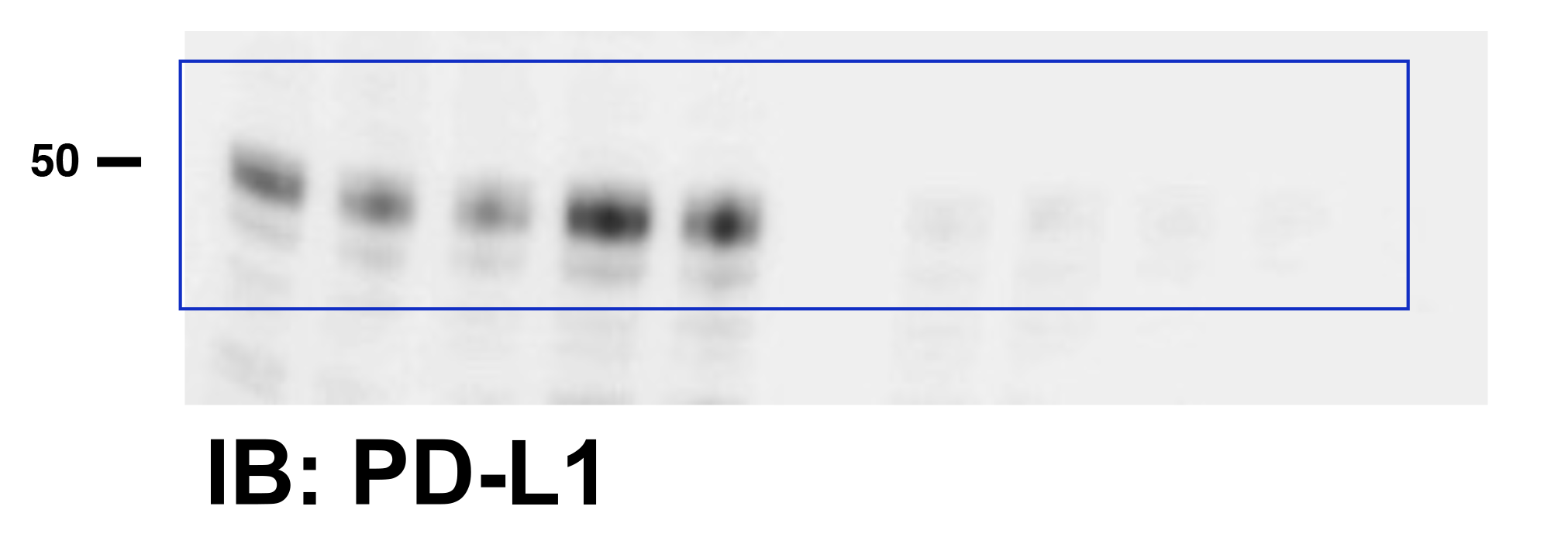

Supplement: Supplementary file 8 — Figure EV1-5 Source Data [file 44319_2024_354_MOESM8_ESM.zip › Figure EV1-5/Figure EV1/EV1A/PD-L1.tif]

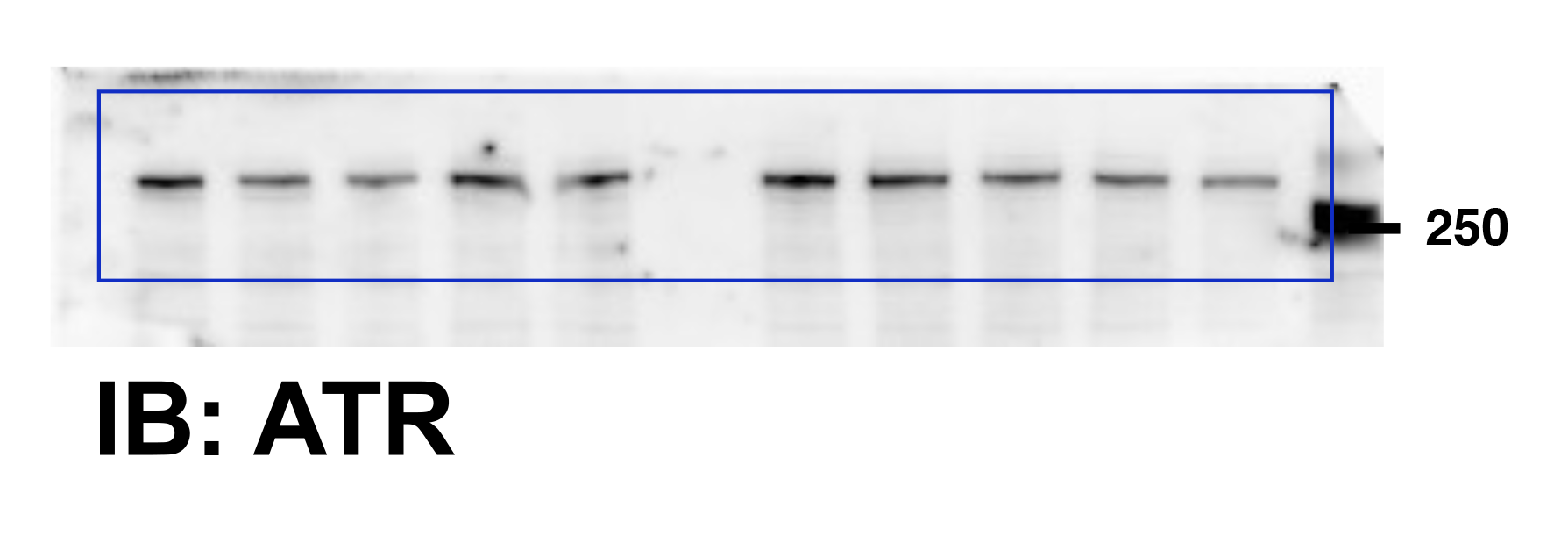

Supplement: Supplementary file 8 — Figure EV1-5 Source Data [file 44319_2024_354_MOESM8_ESM.zip › Figure EV1-5/Figure EV1/EV1A/ATR.tif]

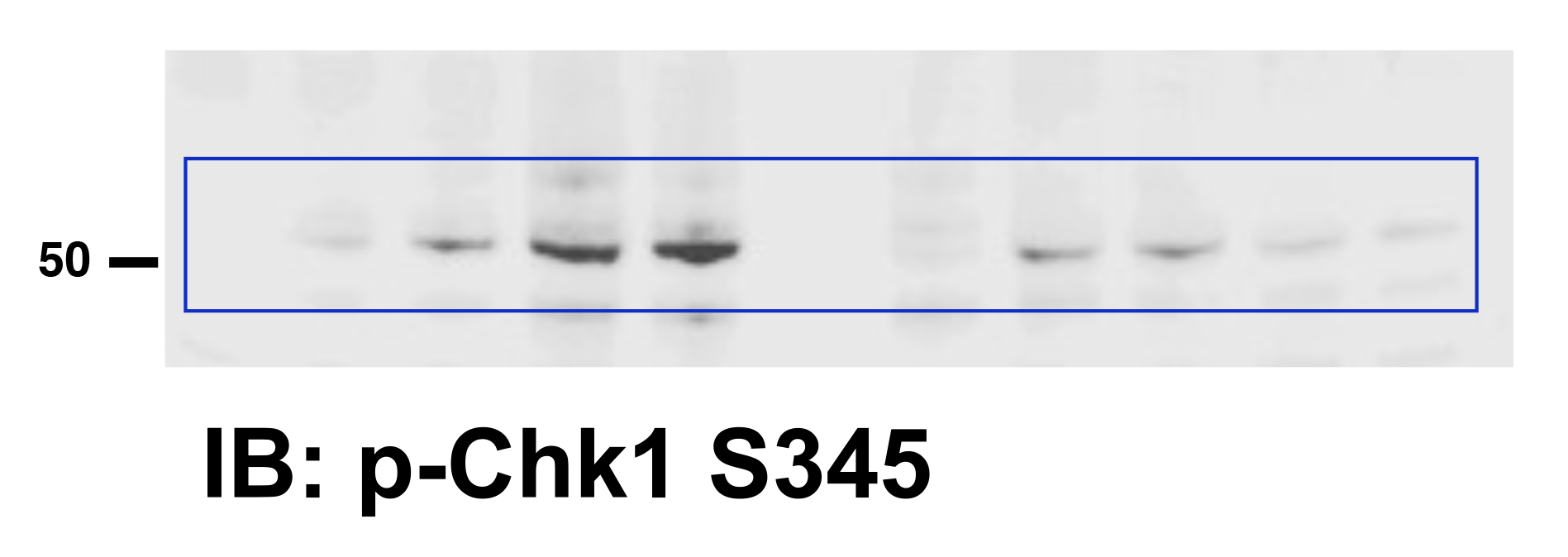

Supplement: Supplementary file 8 — Figure EV1-5 Source Data [file 44319_2024_354_MOESM8_ESM.zip › Figure EV1-5/Figure EV1/EV1A/pChk1.tif]

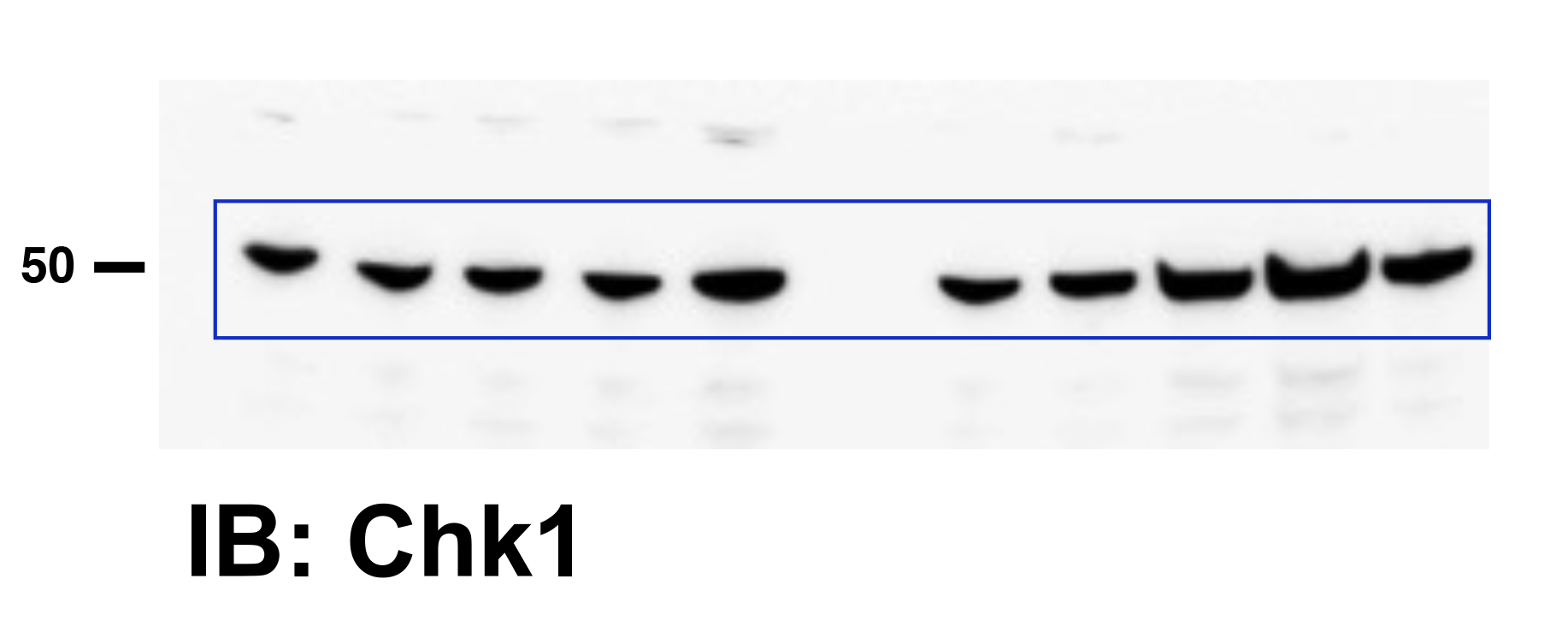

Supplement: Supplementary file 8 — Figure EV1-5 Source Data [file 44319_2024_354_MOESM8_ESM.zip › Figure EV1-5/Figure EV1/EV1A/Chk1.tif]

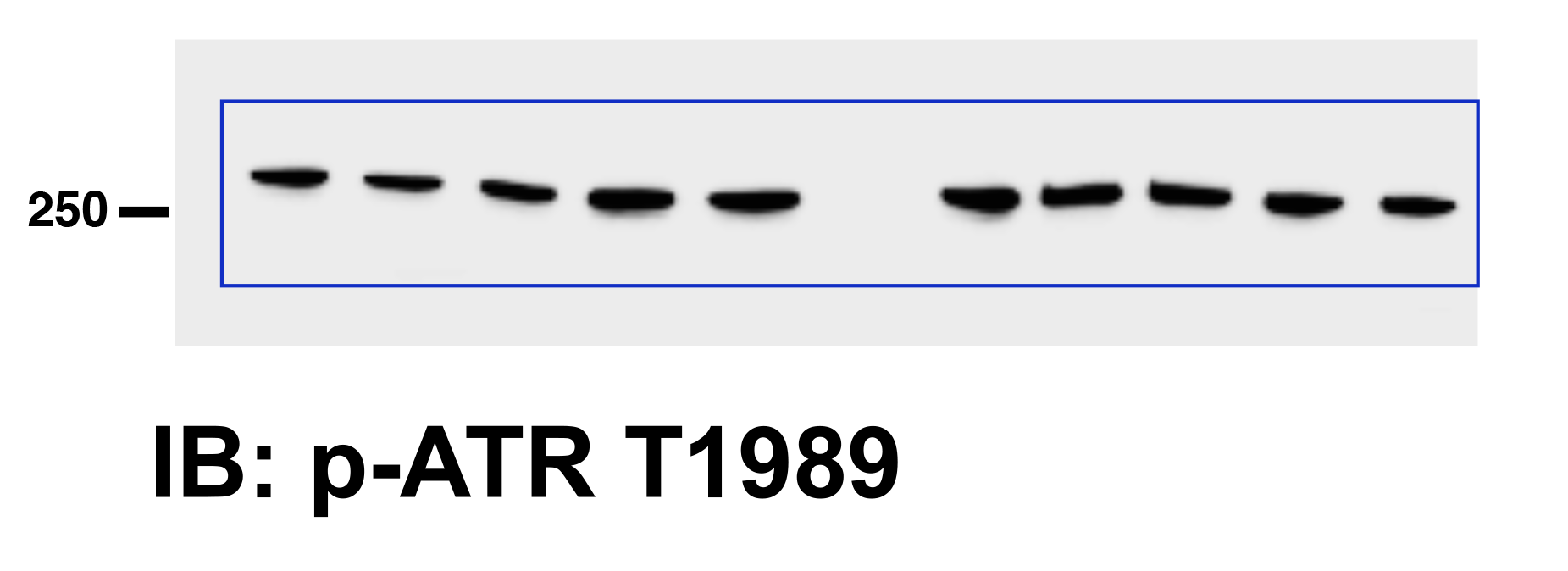

Supplement: Supplementary file 8 — Figure EV1-5 Source Data [file 44319_2024_354_MOESM8_ESM.zip › Figure EV1-5/Figure EV1/EV1A/pATR.tif]

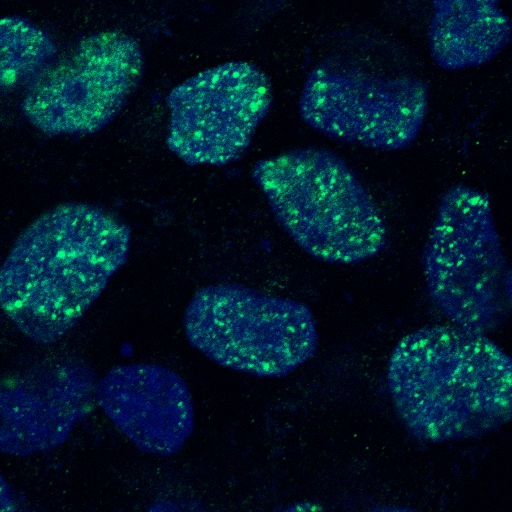

Supplement: Supplementary file 8 — Figure EV1-5 Source Data [file 44319_2024_354_MOESM8_ESM.zip › Figure EV1-5/Figure EV2/EV2A/KO-NCS-6hr-H2AX(g)-BARD1(r)-1/KO-NCS-6hr-H2AX(g)-BARD1(r)-1_c1+2.jpg]

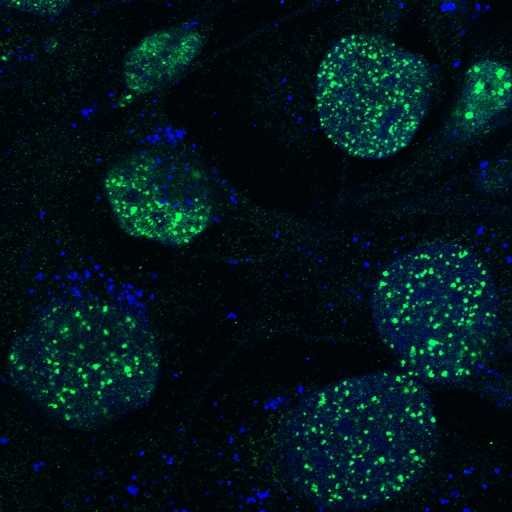

Supplement: Supplementary file 8 — Figure EV1-5 Source Data [file 44319_2024_354_MOESM8_ESM.zip › Figure EV1-5/Figure EV2/EV2A/KO-NCS-24hr-H2AX(g)-BARD1(r)-6/KO-NCS-24hr-H2AX(g)-BARD1(r)-6_c1+2.jpg]

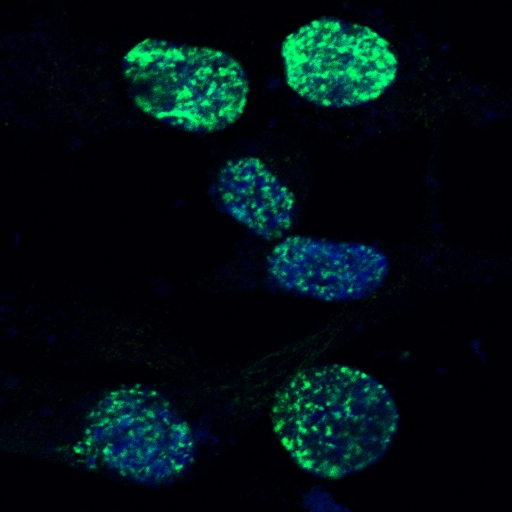

Supplement: Supplementary file 8 — Figure EV1-5 Source Data [file 44319_2024_354_MOESM8_ESM.zip › Figure EV1-5/Figure EV2/EV2A/WT_NCS-30min-H2AX(g)-BARD1(r)-5/WT_NCS-30min-H2AX(g)-BARD1(r)-5_c1+2.jpg]

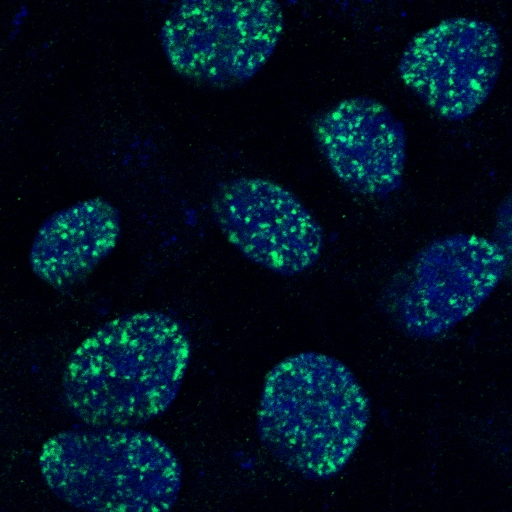

Supplement: Supplementary file 8 — Figure EV1-5 Source Data [file 44319_2024_354_MOESM8_ESM.zip › Figure EV1-5/Figure EV2/EV2A/KO-NCS-30min-H2AX(g)-BARD1(r)-3/KO-NCS-30min-H2AX(g)-BARD1(r)-3_c1+2.jpg]

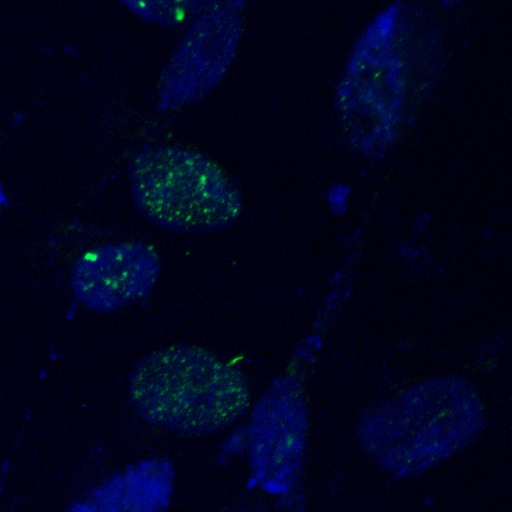

Supplement: Supplementary file 8 — Figure EV1-5 Source Data [file 44319_2024_354_MOESM8_ESM.zip › Figure EV1-5/Figure EV2/EV2A/WT_non-NCS H2AX(g)-BARD1(r)-6/WT_non-treatment H2AX(g)-BARD1(r)-6_c2+3.jpg]

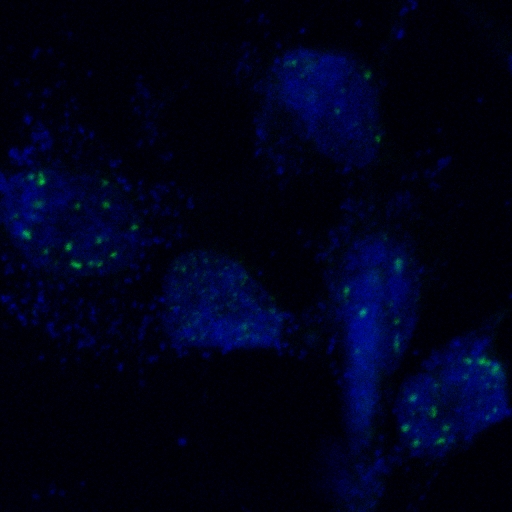

Supplement: Supplementary file 8 — Figure EV1-5 Source Data [file 44319_2024_354_MOESM8_ESM.zip › Figure EV1-5/Figure EV2/EV2A/WT_24hr NCS H2AX(g)-BARD1(r)-21/WT_24hr NCS H2AX(g)-BARD1(r)-21_c2+3.jpg]

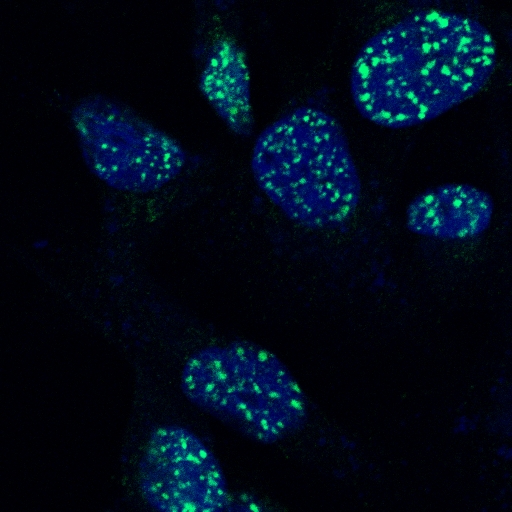

Supplement: Supplementary file 8 — Figure EV1-5 Source Data [file 44319_2024_354_MOESM8_ESM.zip › Figure EV1-5/Figure EV2/EV2A/WT_NCS-6hr-H2AX(g)-BARD1(r)-7/WT_NCS-6hr-H2AX(g)-BARD1(r)-7_c1+2.jpg]

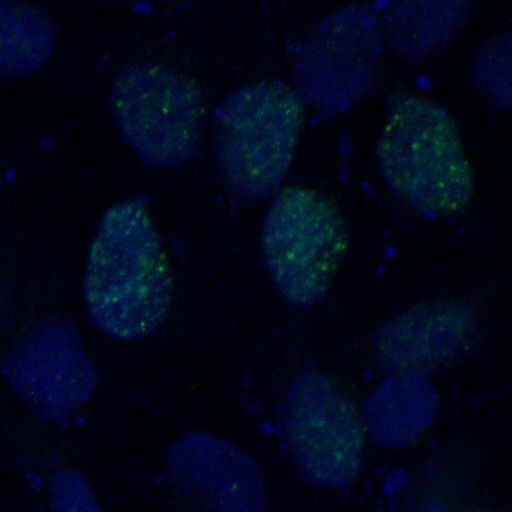

Supplement: Supplementary file 8 — Figure EV1-5 Source Data [file 44319_2024_354_MOESM8_ESM.zip › Figure EV1-5/Figure EV2/EV2A/KO-non-NCS H2AX(g)-BARD1(r)-18/KO-non-NCS H2AX(g)-BARD1(r)-18_c2+3.jpg]

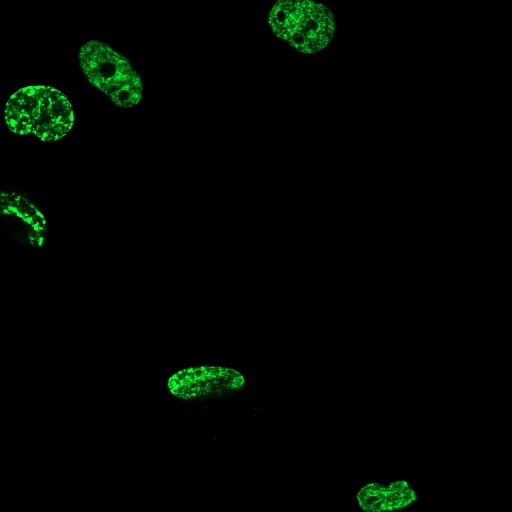

Supplement: Supplementary file 8 — Figure EV1-5 Source Data [file 44319_2024_354_MOESM8_ESM.zip › Figure EV1-5/Figure EV1/EV1D/WT_non-treatment/EdU.jpg]

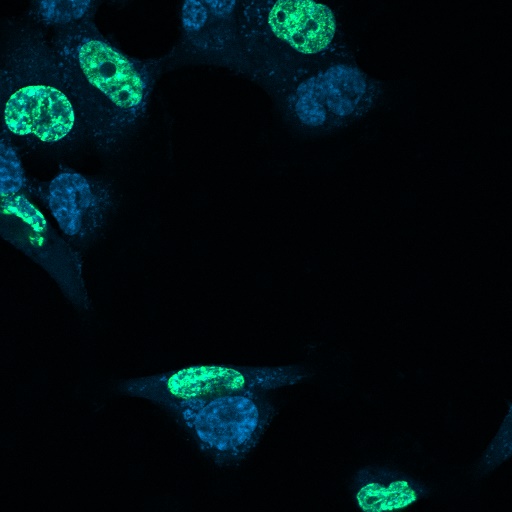

Supplement: Supplementary file 8 — Figure EV1-5 Source Data [file 44319_2024_354_MOESM8_ESM.zip › Figure EV1-5/Figure EV1/EV1D/WT_non-treatment/Merge.jpg]

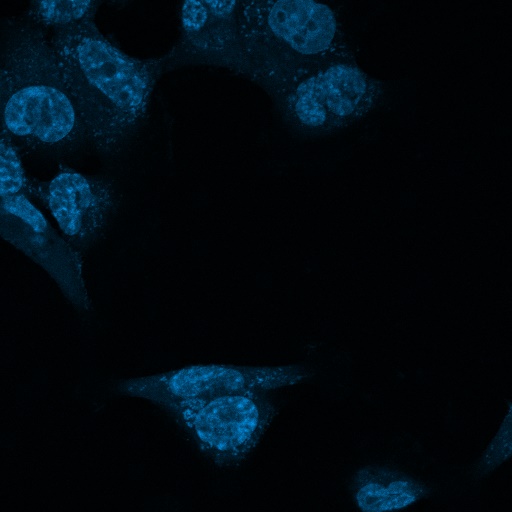

Supplement: Supplementary file 8 — Figure EV1-5 Source Data [file 44319_2024_354_MOESM8_ESM.zip › Figure EV1-5/Figure EV1/EV1D/WT_non-treatment/DAPI.jpg]

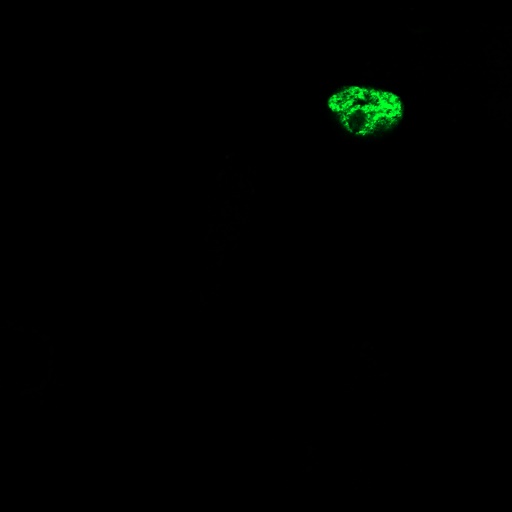

Supplement: Supplementary file 8 — Figure EV1-5 Source Data [file 44319_2024_354_MOESM8_ESM.zip › Figure EV1-5/Figure EV1/EV1D/WT_IR/EdU.jpg]

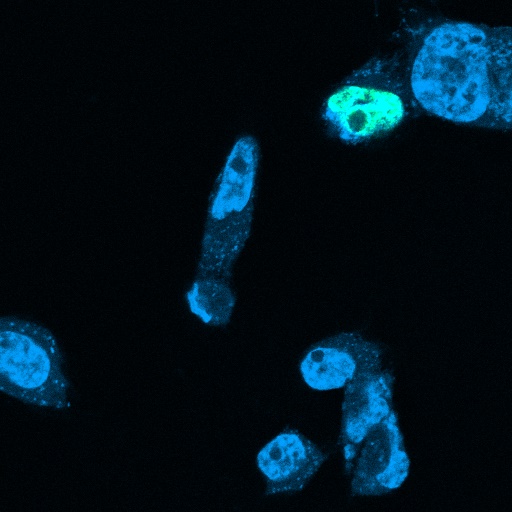

Supplement: Supplementary file 8 — Figure EV1-5 Source Data [file 44319_2024_354_MOESM8_ESM.zip › Figure EV1-5/Figure EV1/EV1D/WT_IR/Merge.jpg]

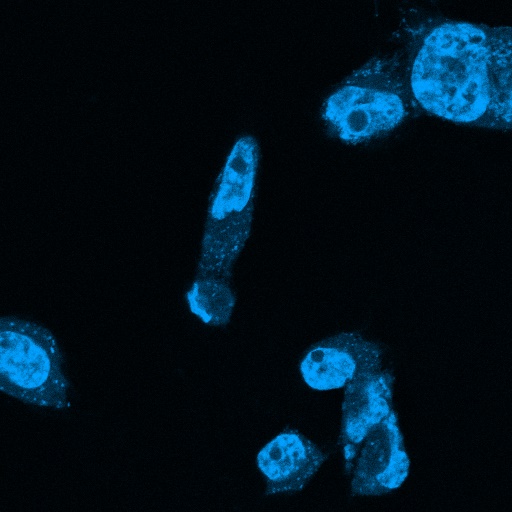

Supplement: Supplementary file 8 — Figure EV1-5 Source Data [file 44319_2024_354_MOESM8_ESM.zip › Figure EV1-5/Figure EV1/EV1D/WT_IR/DAPI.jpg]

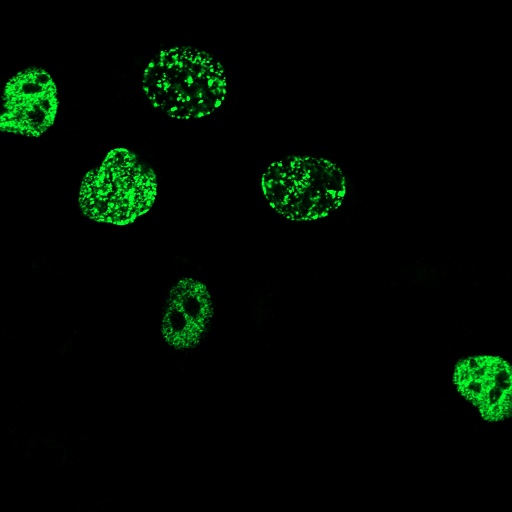

Supplement: Supplementary file 8 — Figure EV1-5 Source Data [file 44319_2024_354_MOESM8_ESM.zip › Figure EV1-5/Figure EV1/EV1D/KO_IR/EdU.jpg]

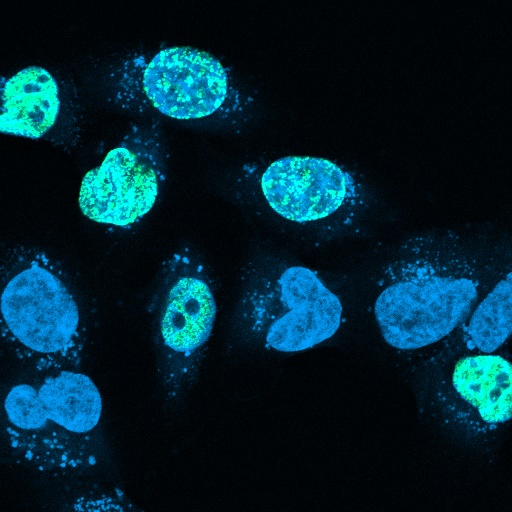

Supplement: Supplementary file 8 — Figure EV1-5 Source Data [file 44319_2024_354_MOESM8_ESM.zip › Figure EV1-5/Figure EV1/EV1D/KO_IR/Merge.jpg]

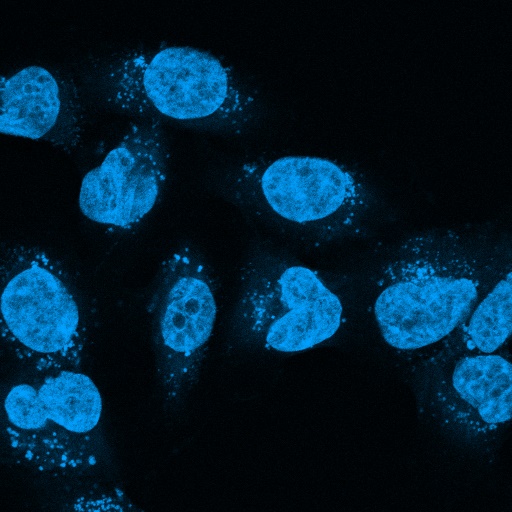

Supplement: Supplementary file 8 — Figure EV1-5 Source Data [file 44319_2024_354_MOESM8_ESM.zip › Figure EV1-5/Figure EV1/EV1D/KO_IR/DAPI.jpg]

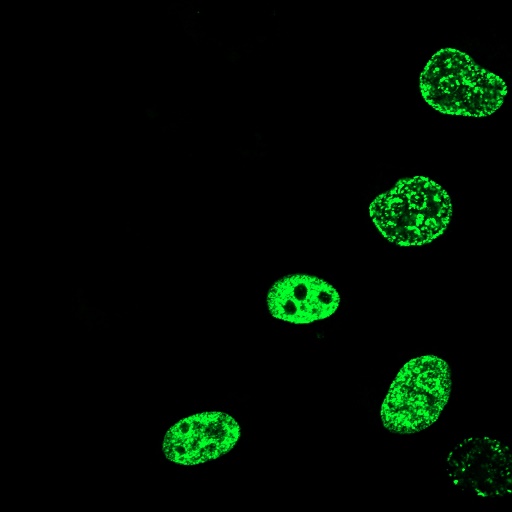

Supplement: Supplementary file 8 — Figure EV1-5 Source Data [file 44319_2024_354_MOESM8_ESM.zip › Figure EV1-5/Figure EV1/EV1D/KO_non-treatment/EdU.jpg]

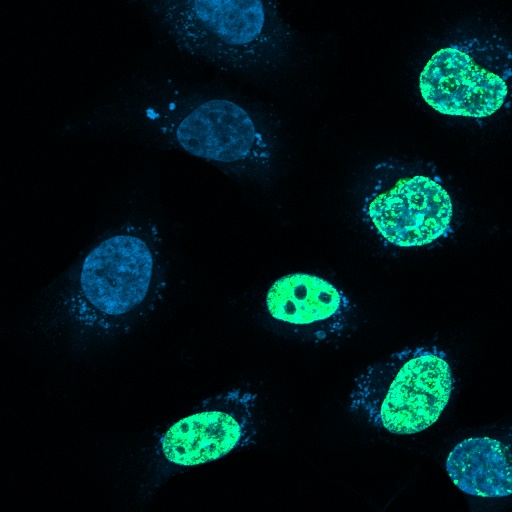

Supplement: Supplementary file 8 — Figure EV1-5 Source Data [file 44319_2024_354_MOESM8_ESM.zip › Figure EV1-5/Figure EV1/EV1D/KO_non-treatment/Merge.jpg]

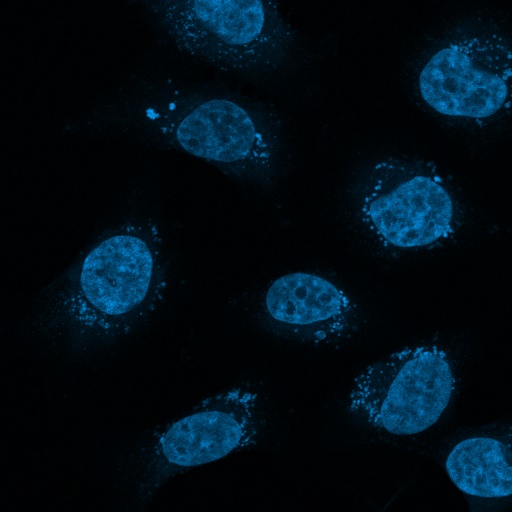

Supplement: Supplementary file 8 — Figure EV1-5 Source Data [file 44319_2024_354_MOESM8_ESM.zip › Figure EV1-5/Figure EV1/EV1D/KO_non-treatment/DAPI.jpg]
