# Supplementary material for: Nuclear PD-L1 triggers tumour-associated inflammation upon DNA damage
Source: EMBO Rep. 2025 Jan 2;26(3):635–55. doi: 10.1038/s44319-024-00354-9 (PMC11811057; doi:10.1038/s44319-024-00354-9)
Supplement: Supplementary file 9 — Expanded View Figures [file 44319_2024_354_MOESM9_ESM.pdf]

Expanded View Figures

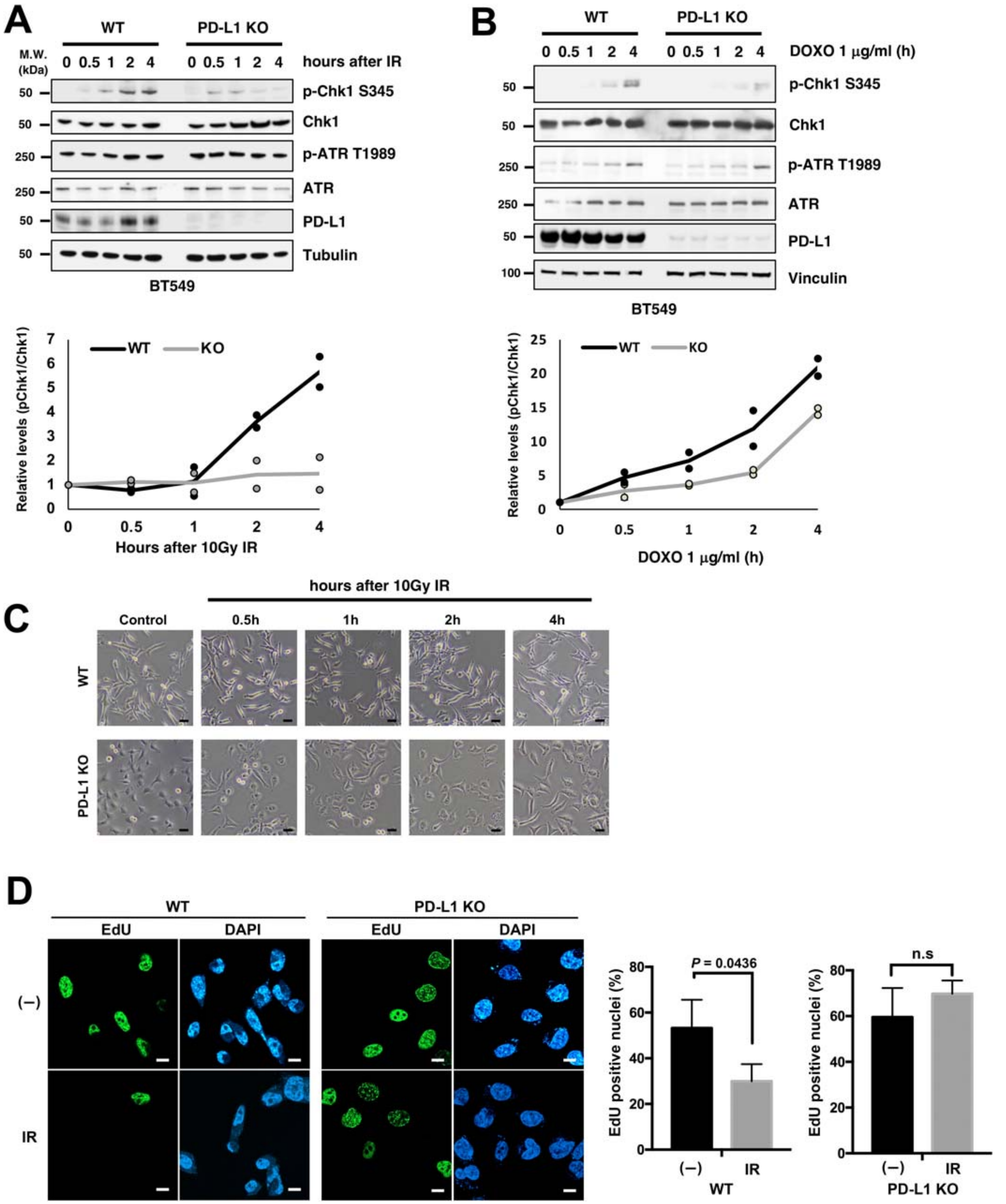

 © The Author(s)

Figure EV1. PD-L1 is involved in ATR-Chk1 signalling pathway.

(**A**, **B**) WT or PD-L1 KO BT549 cells exposed to 10 Gy IR and incubated for the indicated times (**A**) or treated with DOXO for the indicated times (**B**) were subjected to western blotting. The lower panels depict the ratio of phosphorylated Chk1 to total Chk1, as quantified from digital images derived from $n = 2$ biological replicates using the ImageJ software. (**C**) Phase contrast findings of WT or PD-L1 KO MDA-MD-231 cells exposed to 10 Gy IR and incubated for the indicated times. Scale bars: 20 µm. (**D**) WT or PD-L1 KO MDA-MB-231 cells were exposed or not to 5 Gy IR and incubated with EdU for 4 h. Cells that incorporated EdU were examined under a confocal laser scanning microscope. Scale bars: 10 µm. The right panels depict the quantification of the EdU-positive nuclei. The number of EdU-positive cell is shown below the images. Data information: In (**A**, **B**), data are presented as mean with actual values from $n = 2$ biological replicates. In (**D**), data are presented as mean ± SD from $n = 3$ biological replicates, each based on more than 50 cells. *P* values were determined by Student's *t* test (n.s. indicates no significance). Source data are available online for this figure.

© The Author(s)

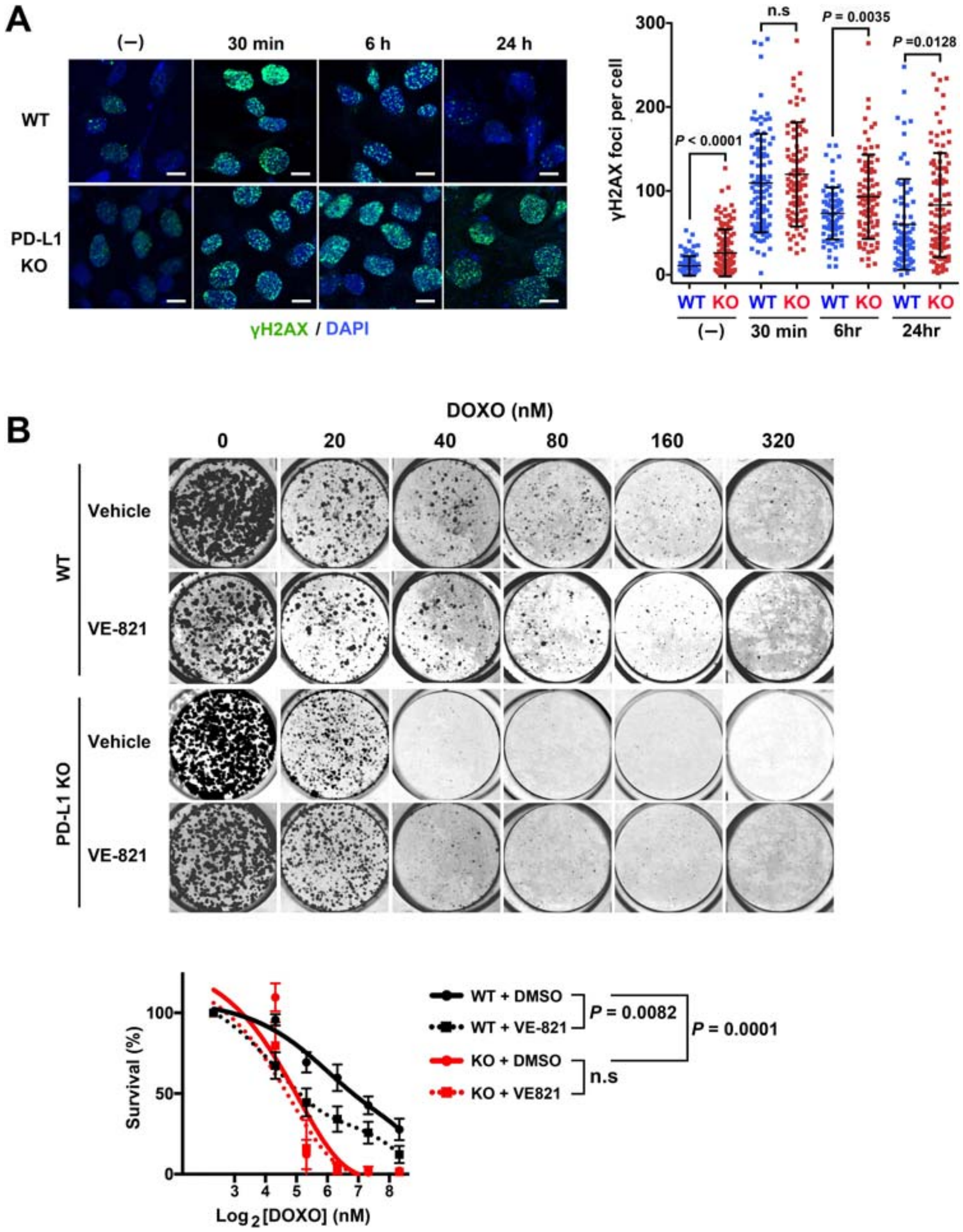

Figure EV2. PD-L1 depletion increases DNA damage and sensitizes cells to DSB agents.

(**A**) WT or PD-L1 KO MDA-MB-231 cells were treated with or without NCS for 15 min, washed, cultured for the indicated time, and subjected to immunofluorescence using the anti-γH2AX antibody. Nuclei were counterstained with DAPI. The number of nuclear γH2AX foci per cell is shown in the right panel. Scale bars: 10 μm. (**B**) WT and PD-L1 KO MDA-MB-231 cells were treated with the indicated doses of DOXO in the presence of vehicle DMSO or VE-821 for 24 h, then analyzed for clonogenic survival. The relative survival data are shown below the images. Data information: In (**A**), data are representative of $n = 2$ biological replicates and are shown as mean ± SD with actual values. P values were determined by Student's t test (n.s. indicates no significance). In (**B**), data are presented as mean ± SD from $n = 3$ biological replicates. Statistical significance was calculated using a two-way ANOVA (n.s. indicates no significance). Source data are available online for this figure.

 © The Author(s)

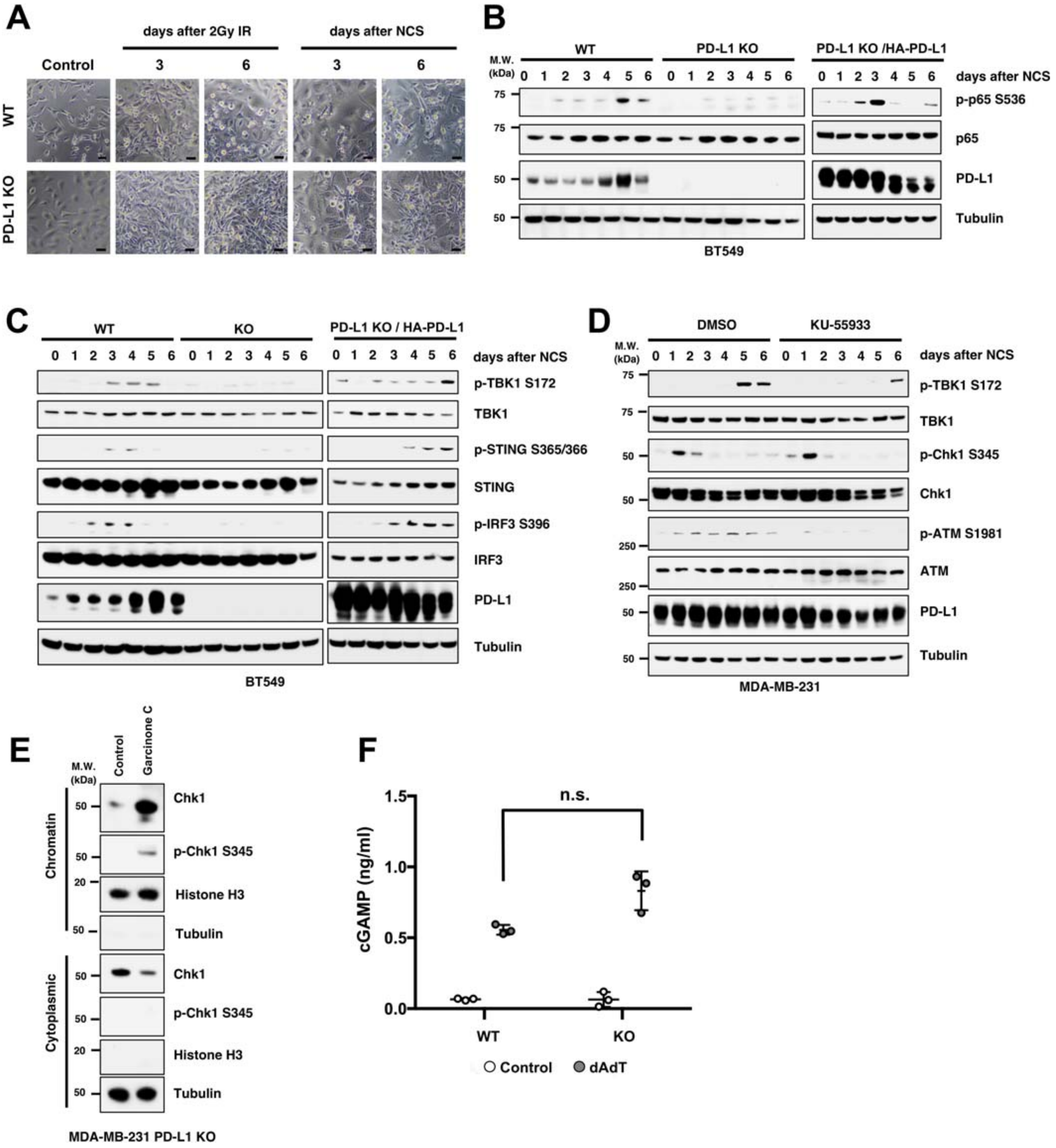

Figure EV3. PD-L1 promotes NF-κB and cGAS-STING activation following genotoxic stress.

(**A**) Phase contrast findings of WT or PD-L1 KO MDA-MD-231 cells exposed to 2 Gy IR and incubated for the indicated times. Scale bars: 20 μm. (**B**, **C**) WT and PD-L1 KO BT549 cells, or PD-L1 KO cells re-expressing HA-PD-L1 were untreated (day 0) or treated with NCS, incubated for the indicated time, and subjected to western blotting. (**D**) MDA-MB-231 cells untreated (day 0) or treated with NCS were cultured with vehicle DMSO or KU-55933 for the indicated time and subjected to western blotting. (**E**) PD-L1 KO MDA-MB-231 cells were treated with DMSO (Control) or Garcinone C for 24 h. Cells were fractionated into chromatin and cytoplasmic fractions and analyzed by western blotting. (**F**) WT and PD-L1 KO BT549 cells were transfected with or without oligo DNA dAdT and incubated for 24 h, and the concentrations of cellular cGAMP were analyzed using ELISA. Data information: All western blot data are representative of at least $n = 2$ biological replicates. In (**F**), data are representative of $n = 2$ biological replicates and are shown as mean ± SD from $n = 3$ technical replicates. *P* value was determined by Student's *t* test (n.s. indicates no significance). Source data are available online for this figure.

© The Author(s)

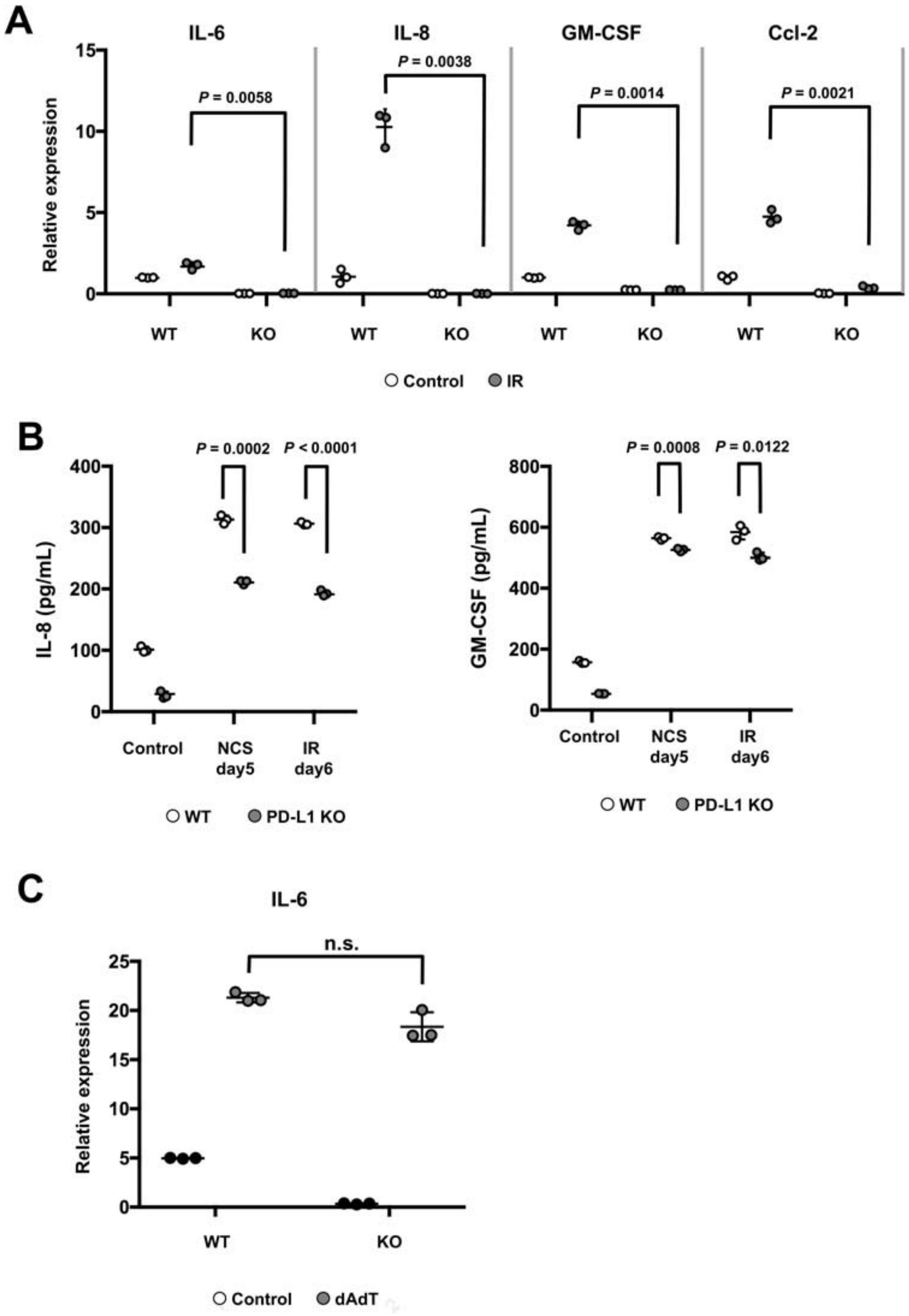

Figure EV4. PD-L1 depletion suppresses proinflammatory chemocytokines in the late phase following genotoxic stress.

(**A**) WT or PD-L1 KO MDA-MB-231 cells exposed to 2 Gy IR and cultured for 6 days were analyzed for mRNA expression levels of IL-6, IL-8, GM-CSF and Ccl-2 by qRT-PCR. The scores were normalized to untreated WT cells. (**B**) WT and PD-L1 KO MDA-MB-231 cells were untreated (control), or treated with NCS or exposed to IR and incubated for 5 or 6 days, respectively, and the concentrations of secreted IL-8 and GM-CSF in culture medium were analysed using ELISA. (**C**) WT and PD-L1 KO BT549 cells were non-transfected or transfected with oligo DNA dAdT, incubated for 24 h, and analyzed for the mRNA expression levels of IL-6 using qRT-PCR. The scores were normalized to those of untreated WT cells. Data information: All data are representative of $n = 2$ biological replicates and are shown as mean ± SD from $n = 3$ technical replicates. *P* values were determined by Student's *t* test (n.s. indicates no significance). Source data are available online for this figure.

 © The Author(s)

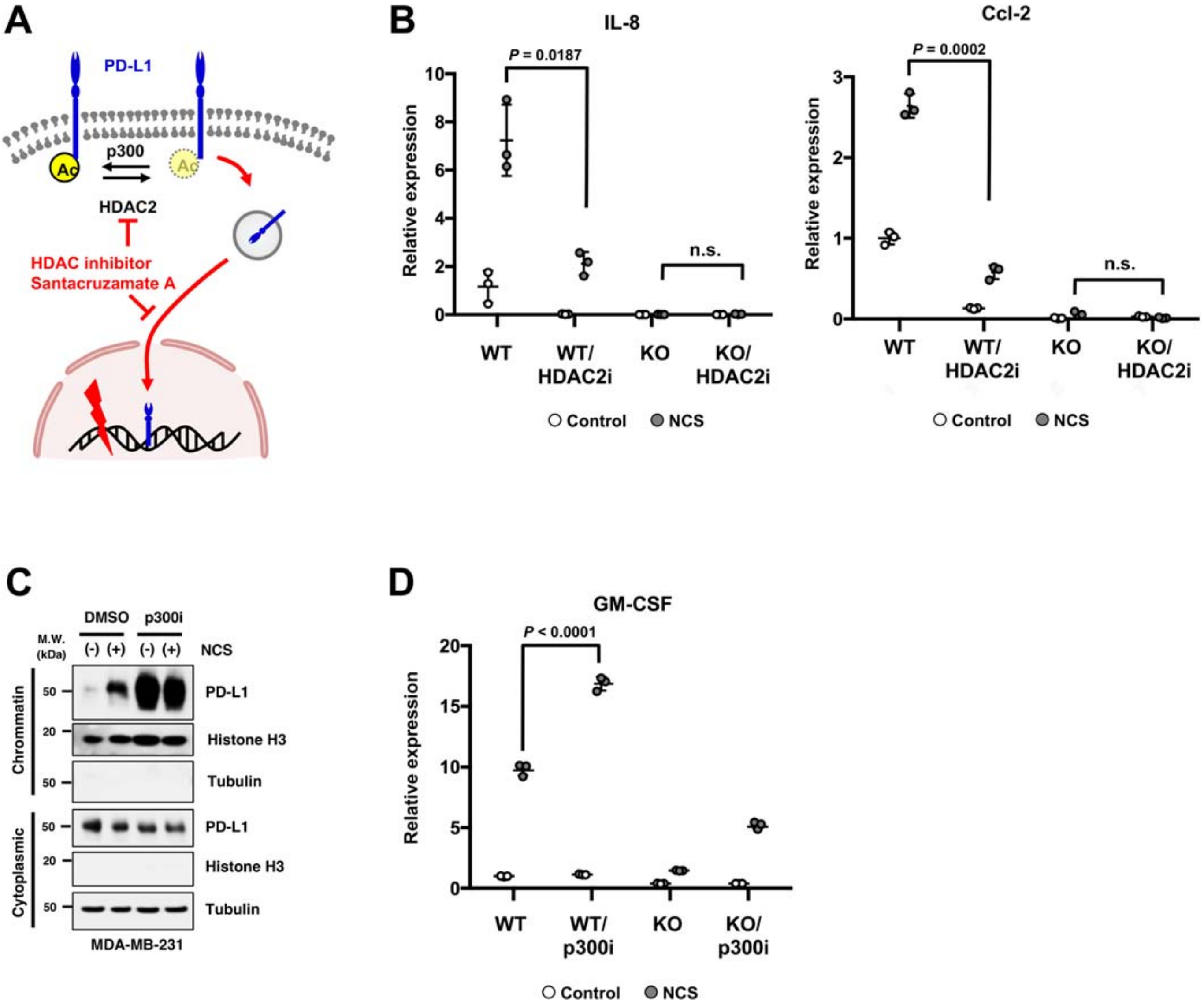

Figure EV5. Nuclear translocation of PD-L1 is mediated by HDAC2.

(**A**) The diagram depicts a role of HDAC2 in nuclear translocation of PD-L1 (Gao et al, 2020). PD-L1 expressing on the plasma membrane is constitutively acetylated by p300 on its C-terminus. HDAC2-mediated deacetylation of PD-L1 triggers its translocation into the nucleus from the membrane. HDAC2-specific inhibitor Santacruzamate A blocks this nuclear translocation. (**B**) WT and PD-L1 KO MDA-MB-231 cells untreated (control) or treated with NCS and incubated for 5 days in the presence or absence of the HDAC2 inhibitor Santacruzamate A were subjected to qRT-PCR for the indicated chemocytokines. (**C**) MDA-MB-231 cells were untreated (−) or treated with NCS (+) and incubated for 5 days in the presence or absence of the p300 inhibitor C646. Cell lysates were fractionated into chromosomal and cytoplasmic fractions and analyzed by western blotting. Data are representative of $n = 2$ biological replicates. (**D**) WT and PD-L1 KO MDA-MB-231 cells untreated (control) or treated with NCS and incubated for 5 days in the presence or absence of the p300 inhibitor C646 were subjected to qRT-PCR for the indicated chemocytokines. Data information: In (**B**, **D**), data are representative of $n = 2$ biological replicates and are shown as mean ± SD from $n = 3$ technical replicates. *P* values were determined by Student's *t* test (n.s. indicates no significance). Source data are available online for this figure.
